# LnDOTA Releasing Probes for Luminescence and Magnetic Resonance Imaging

**DOI:** 10.1021/acs.inorgchem.5c00199

**Published:** 2025-03-24

**Authors:** Ceri A. Foster, Deborah Sneddon, Lina Hacker, Euan T. Sarson, Max Robertson, Daria Sokolova, Louise A. W. Martin, Matthew F. Allen, Alexandr Khrapichev, Kylie A. Vincent, Ester M. Hammond, Stuart J. Conway, Stephen Faulkner

**Affiliations:** † Department of Chemistry, Chemistry Research Laboratory, University of Oxford, Mansfield Road, Oxford OX1 3TA, United Kingdom; ‡ Department of Chemistry, School of Life Sciences, University of Sussex, Falmer, Brighton BN1 9QJ, United Kingdom; § Department of Oncology, University of Oxford, Old Road Campus Research Building, Roosevelt Drive, Oxford OX3 7DA, United Kingdom; ∥ Department of Chemistry and Biochemistry, University of California Los Angeles, 607 Charles E. Young Drive East, Los Angeles, California 90095-1569, United Kingdom

## Abstract

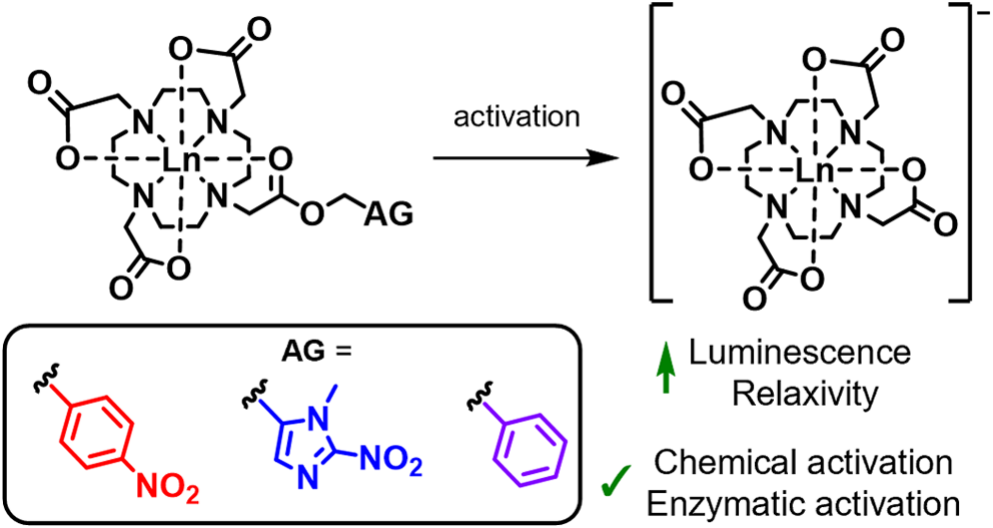

Lanthanide complexes of DOTA monoesters bearing nitrobenzyl and nitroimidazole groups are shown to be converted to the corresponding DOTA complexes under chemical and enzymatic conditions, giving rise to favorable changes in the luminescence properties of the europium and terbium complexes and relaxometric properties of the gadolinium complexes. The nitroimidazole complexes are converted more rapidly than their nitrobenzyl and benzyl analogues. We propose that activation of these complexes may occur by ester cleavage rather than nitro reduction and fragmentation since complexes bearing a simple benzyl group may also be cleaved under the same conditions, albeit more slowly.

## Abstract 2

Lanthanide complexes of DOTA monoesters bearing nitrobenzyl and nitroimidazole groups are shown to be converted to the corresponding DOTA complexes and cause a change in the spectroscopic and relaxometric properties observed. We propose that activation under chemical and enzymatic conditions may occur via an ester cleavage pathway, with faster rates observed for the nitroimidazole analogues.

## Introduction

Redox balance is crucial in the body, as reductive and oxidative stress can negatively impact cell proliferation and survival. Reductive stress is generally less well characterized than oxidative stress; therefore, recent research has focused on investigating reductive stress, particularly its role in cancer progression and treatment.^1,2^ Many species and reductive enzymes are upregulated during reductive stress, including NAD(P)H, GSH, and NAD(P)H-dependent enzymes, such as nitroreductases.^3,4^ A prodrug or profluorophore approach has been extensively used in this area and others to develop chemical probes that are selectively activated under reducing conditions. For example, nitroreductase enzymes have been used to selectively activate profluorophores in hypoxic (low oxygen) cancer cells^5^ and for the detection of bacterial infection.^6,7^

In general, these prodrugs and profluorophores employ bioactivatable moieties such as N-oxide, nitroaryl,^6,8−15^ indolequinone,^16^ azo,^17−19^ and azide^5,20^ functional groups, which cleave to release known anticancer drugs or imaging agents. Within the nitroaryl bioreductive groups, the 2-nitroimidazole and 4-nitrobenzyl moieties are among the most commonly used groups in the literature.^21^ The 2-nitroimidazole group is often favored over the nitrobenzyl group due to its higher one-electron reduction potential (around −250 mV for 1-methyl-2-nitro-1H-imidazole-5-carbaldehyde, compared to −425 mV, vs NHE, for 4-nitrobenzoic acid);^8,22^ however, both reduction potentials fall within the range of physiological reducing environments; for instance, hypoxic A549 cells were previously measured to have a reduction potential in the range of approximately −330 to −440 mV vs NHE (potential measured at corrected pH),^23^ while the midpoint potential of NAD(P)H at pH 7 is −320 mV vs SHE. This suggests that it is more thermodynamically favorable to activate nitroimidazole-containing prodrugs than their nitrobenzyl analogues, as evidenced by the trend in activation observed by Calder and co-workers.^9^

Luminescent lanthanide complexes may offer alternatives to organic fluorophores due to their advantageous sharp emission bands and long emission lifetimes, enabling the removal of background biological autofluorescence and scattered light by using time-gated techniques.^24,25^ For *in* vivo imaging, magnetic resonance imaging (MRI) is a noninvasive clinical imaging technique that offers high spatial resolution^26^ without requiring ionizing radiation, unlike positron emission tomography, computed tomography, and single-photon emission computed tomography.^27,28^ Gadolinium analogues of luminescent lanthanide complexes may be used as MRI contrast agents that modify the relaxation rates of water.

MRI signal intensity can be varied by changing the physical parameters of the complexes, including the number of inner-sphere water molecules (*q*), the rate of molecular tumbling (τ_r_), and the rate of water exchange (τ_m_), among other parameters, such as electron spin relaxation.^29,30^ Furthermore, luminescence emission is inversely proportional to *q*, as OH oscillators from inner-sphere water molecules quench luminescence by vibrational energy transfer.^31^ Therefore, responsive probes may be designed where a change in luminescence and MRI contrast can be monitored using the same scaffold.

**Figure 1:**
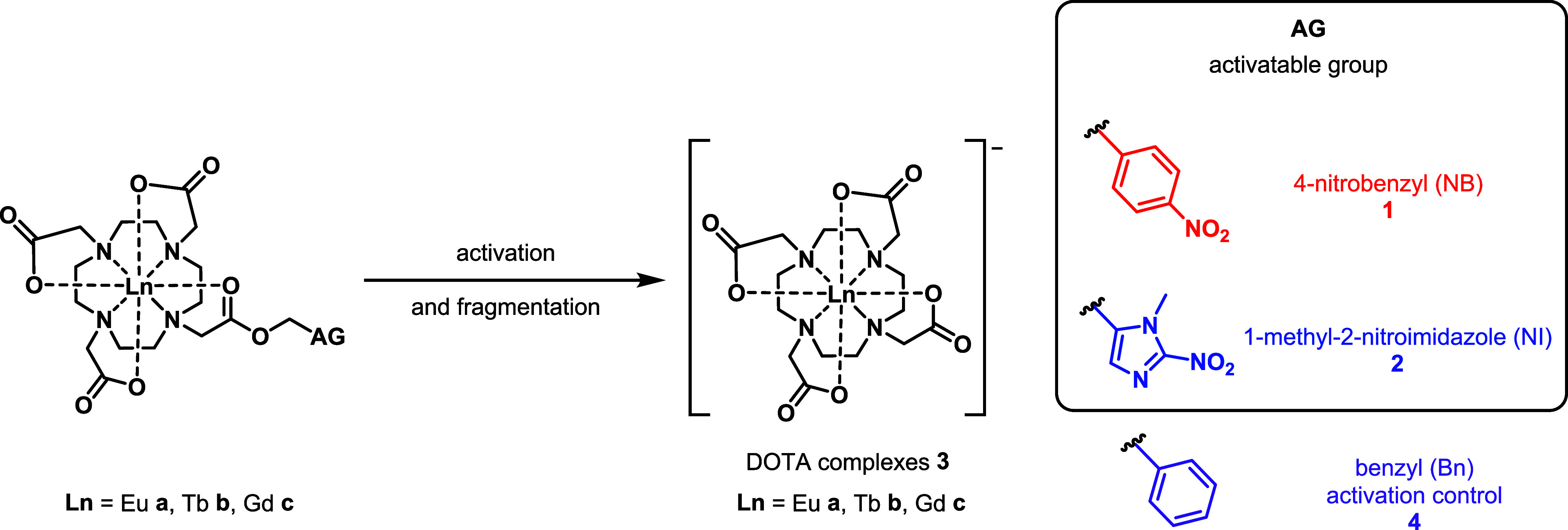
Structures of the activatable lanthanide complexes and the benzyl control, discussed here, and the corresponding activated DOTA complexes.

The use of bioreductive groups has been explored in the development of several MRI probes,^32−37^ including several self-immolative responsive MRI probes, including examples where the coordination sphere of the lanthanide changes upon activation.^38−41^ Nazaré and co-workers reported a nitrobenzyl gadolinium complex that, in the presence of nitroreductases, may be activated to give gadolinium DOTA, a commercial contrast agent, causing a change in MRI contrast.^7^ Our work expands on this approach by utilizing the europium, terbium, and gadolinium analogues of both 4-nitrobenzyl (NB) and 1-methyl-2-nitroimidazole (NI) DOTA esters (Figure 1) to develop lanthanide complexes whose activation to the known DOTA complexes (by the mechanisms proposed in Figure S1) may be monitored by luminescence for optical imaging and ^1^H NMR, in addition to changes in relaxivity for MRI.

## Results and Discussion

### Synthesis of Lanthanide Complexes

**Figure 2:**
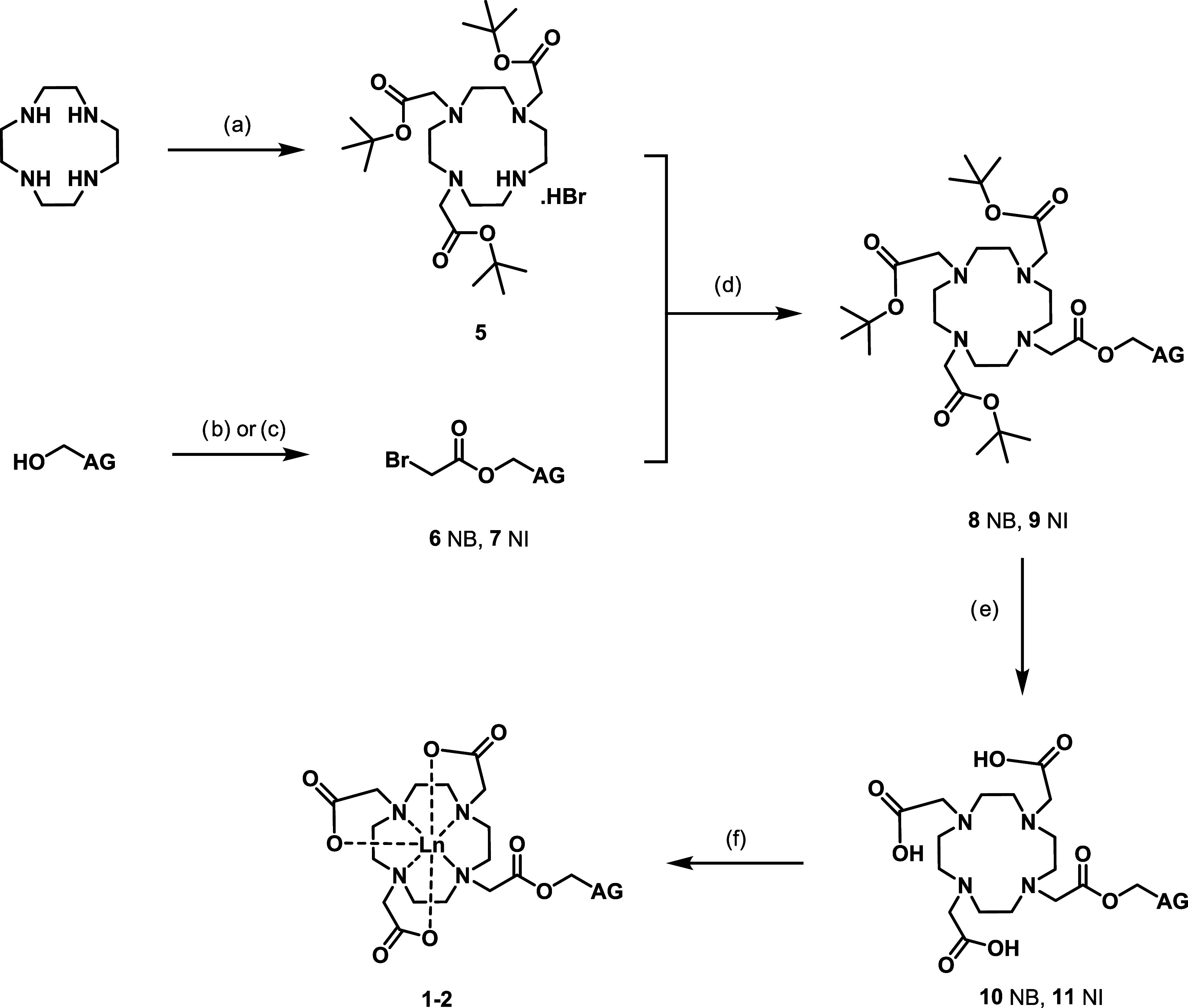
Synthetic route for the lanthanide complexes **1–2**, where AG = activatable group, 4-nitrobenzyl (NB) or 1-methyl-2-nitroimidazole (NI). Reagents and conditions: (a) NaHCO_3_, *tert*-butylbromoacetate, MeCN, 0 °C to rt, 44 h, 40%; (b) bromoacetyl bromide, NaHCO_3_, MeCN, 45 °C, 18 h, 55%; or (c) bromoacetyl bromide, 2,6-ditertbutylpyridine, DMF:CH_2_Cl_2_ 1:1, rt, 15 h, 43–58%; (d) NaHCO_3_, MeCN, 80 °C, 39–42 h, 72–95%; (e) TFA, CH_2_Cl_2_, rt, 20–43 h, 55–95%; (f) Ln(OTf)_3_, MES buffer (1 M, pH 6.0), rt, 1–1.5 h, 27–66%.

The target nitrobenzyl (NB, **1a**–**1c**) and nitroimidazole (NI, **2a**–**2c**) complexes were synthesized according to the general synthetic scheme shown in Figure 2, with full experimental details and characterization reported in the SI. The nitroimidazole alcohol precursor was synthesized using adapted literature procedures,^42^ as detailed in Scheme S1. Compounds **8** and **9** were obtained by reacting bromoacetyl compounds **6** and **7** with the tris *tert*-butyl ester of DO3A, **5**. Cleavage of the protecting *tert*-butyl groups with trifluoroacetic acid gave ligands **10** and **11**. Complexation was carried out using the corresponding lanthanide triflate salt in MES buffer (pH 6.0), and the resulting complexes were purified by semipreparative HPLC. The europium and terbium analogues of the positive control DOTA complexes (**3a** and **3b**, respectively) were synthesized following adapted literature procedures^43,44^ (Scheme S2)—for the gadolinium complexes, the commercial contrast agent Dotarem was used as a positive control. Benzyl-only analogues of **1**, EuBn (**4a**) and TbBn (**4b**), were synthesized as controls for chemical and enzymatic reduction assays and electrochemical measurements (Scheme S3).

### Changes in Luminescence Properties of Lanthanide Complexes

The photophysical properties of the europium and terbium analogues of the NB and NI complexes were characterized by UV–visible absorbance spectroscopy (Figures S2–S3), steady-state emission (Figures S4–S6, 3a, and S9) and excitation spectroscopy (Figures S7–S8), time-gated emission spectroscopy (Figures S10–S13), and luminescence lifetimes (Tables S3–S6 and Figures S14–S17), in water. These photophysical properties were compared to those of the parent DOTA complexes to determine whether these complexes can be used as effective profluorophores, with an increase in luminescence observed upon activation.

**Figure 3:**
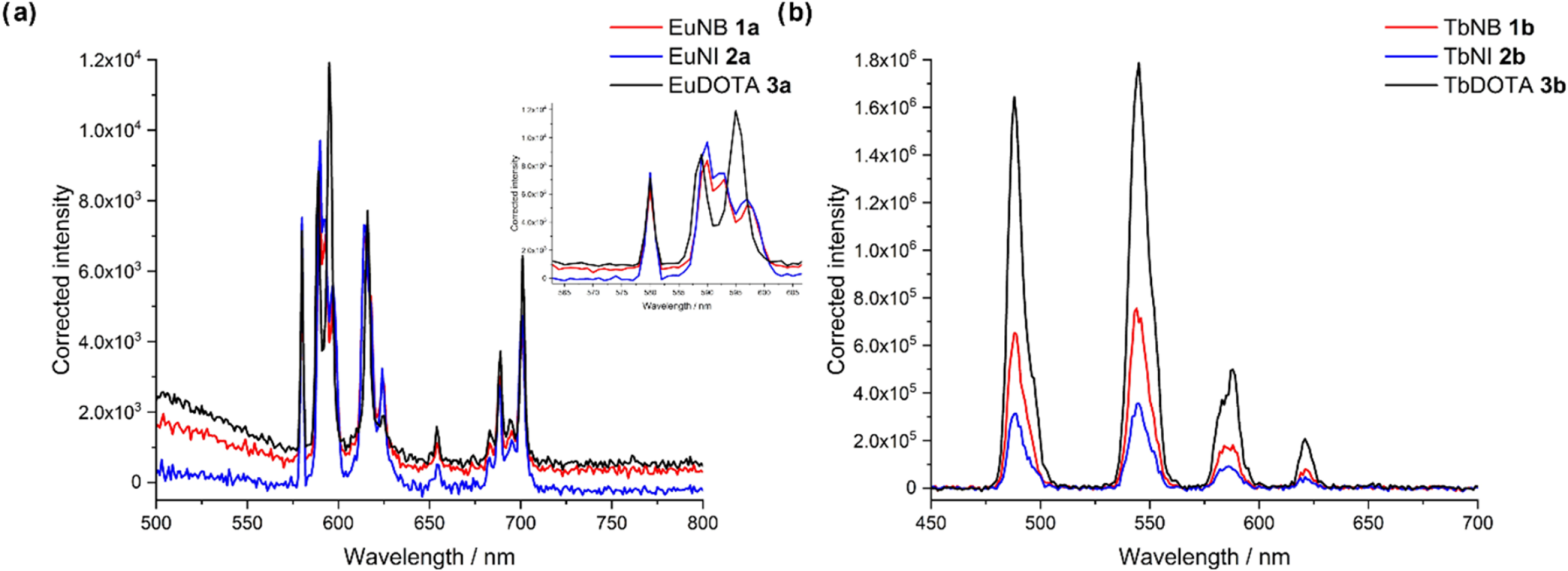
(a) Steady-state emission upon direct metal excitation (λ_ex_ 397 nm) of EuNB (**1a**, red, Φ = 0.09–0.13) and EuNI (**2a**, blue, Φ = 0.11–0.16) in comparison to EuDOTA (**3a**, black, Φ = 0.08–0.11), with inset zoomed on the *J* = 0 and *J* = 1 bands to highlight the change in symmetry observed. (b) Time-gated emission upon direct metal excitation (λ_ex_ 366 nm) of TbNB (**1b**, red) and TbNI (**2b**, blue) in comparison to TbDOTA (**3b**, black) demonstrating a change in luminescence emission. Further details on quantum yield measurements are available in the SI.

Excitation at the maximum absorbance of the ligand (269 nm for NB and 320 nm for NI) for steady-state emission spectroscopy showed that the ligands are poor sensitizers of both europium (Figure S4) and terbium (Figure S5), giving emission profiles and intensities similar to those of the DOTA analogues. Emission from the ligand was observed for EuNI (**2a**) and TbNI (**2b**, Figure S6), distorting emission spectra between 450 and 600 nm but not for the corresponding NB complexes. The excitation spectra of the nitrobenzyl and nitroimidazole complexes (Figures S7–S8) reveal limited contributions from the ligand chromophore to the observed emission, suggesting inefficient transfer between the chromophore and the lanthanide metal, arising due to competing nonradiative deactivation pathways such as back-energy transfer and quenching by the surrounding solvent.

Direct excitation of the europium metal (397 nm, ^5^L_6_ ← ^7^F_0_, Figure 3a) of the NI (**2a**) and NB (**1a**) complexes produced steady-state emission spectra with similar intensities to those of the parent DOTA complexes; however, a difference in the shape of the *J* = 1 band (^5^D_0_ → ^7^F_1_ transition, 585–600 nm) was observed (Figure 3a, inset), alongside a small change in the shape of the *J* = 4 band (Figures 3a, S10, and S12), consistent with a change in symmetry. The *J* = 1 band in EuNB and EuNI was observed as three peaks, consistent with a C_1_ symmetric species having three nondegenerate crystal field levels of the ^7^F_1_ state, whereas EuDOTA gives two peaks, consistent with a C_4_ symmetric species with a nondegenerate and twofold degenerate crystal field level of the ^7^F_1_ state.^45^ In contrast to the similar steady-state emission observed for the europium complexes upon direct metal excitation, the emission of the terbium complexes, upon excitation at 366 nm (Tb ^5^L_10_ ← ^7^F_6_, Figure S9), varied dependent on the ligand, with an increase in intensity observed in the order TbNI < TbNB ∼ TbDOTA, similar to that observed under ligand excitation.

The time-gated emission spectra mirror the trends seen in the steady-state spectra, with little change observed between spectra of the europium complexes irrespective of ligand or direct metal excitation (Figures S10 and S12) and decreased emission intensity observed for TbNB and TbNI in comparison to TbDOTA (Figures S11, S13, and 3b). The largest modulation in emission intensity is observed for the terbium complexes upon time-gated direct metal excitation (Figure 3b). This photophysical data suggest that a change in emission would occur upon conversion to the parent DOTA complex and that the conversion of TbNB and TbNI may be monitored by time-gated luminescence measurements. While the europium complexes exhibit similar quantum yields relative to EuDOTA, there are dramatic differences in the relative quantum yields of the terbium complexes, particularly TbNI (for which conversion to TbDOTA results in a fivefold enhancement of luminescence).

The luminescence lifetimes of the europium and terbium complexes were measured through direct excitation (397 nm for europium and 366 nm for terbium) and ligand excitation in both water and deuterium oxide. These lifetimes were analyzed using the modified Horrocks equation (eq S3, Tables S3–S6, and Figures S14–S17) to determine the number of bound water molecules in the inner coordination sphere, *q*, as this parameter may have a significant influence on relaxivity (eq S5). For EuNB, EuNI, and TbNB in air, a monoexponential decay was observed, and *q* was determined to be approximately 1, within the experimental error, which, in combination with the ^1^H NMR spectra of the europium/terbium complexes (Figures S18–S19), suggests that the ester carbonyl is coordinating to the lanthanide metal, giving an eight-coordinate lanthanide complex. The lifetimes of TbNI measured in air, upon either ligand or metal excitation, were found to be biexponential, containing one short component (∼0.2 ms) and one long component similar to those of the DOTA and NB complexes, with the long component giving a *q* value of approximately 1. Degassing of TbNI samples with argon caused an increase in the time-gated emission intensity (Figure S20) and lifetime measurements to be monoexponential (Figure S21), with lifetimes of ∼2.0 ms, suggesting that oxygen quenching occurs in the air.

### Changes in Relaxivity of Lanthanide Complexes

**Figure 4:**
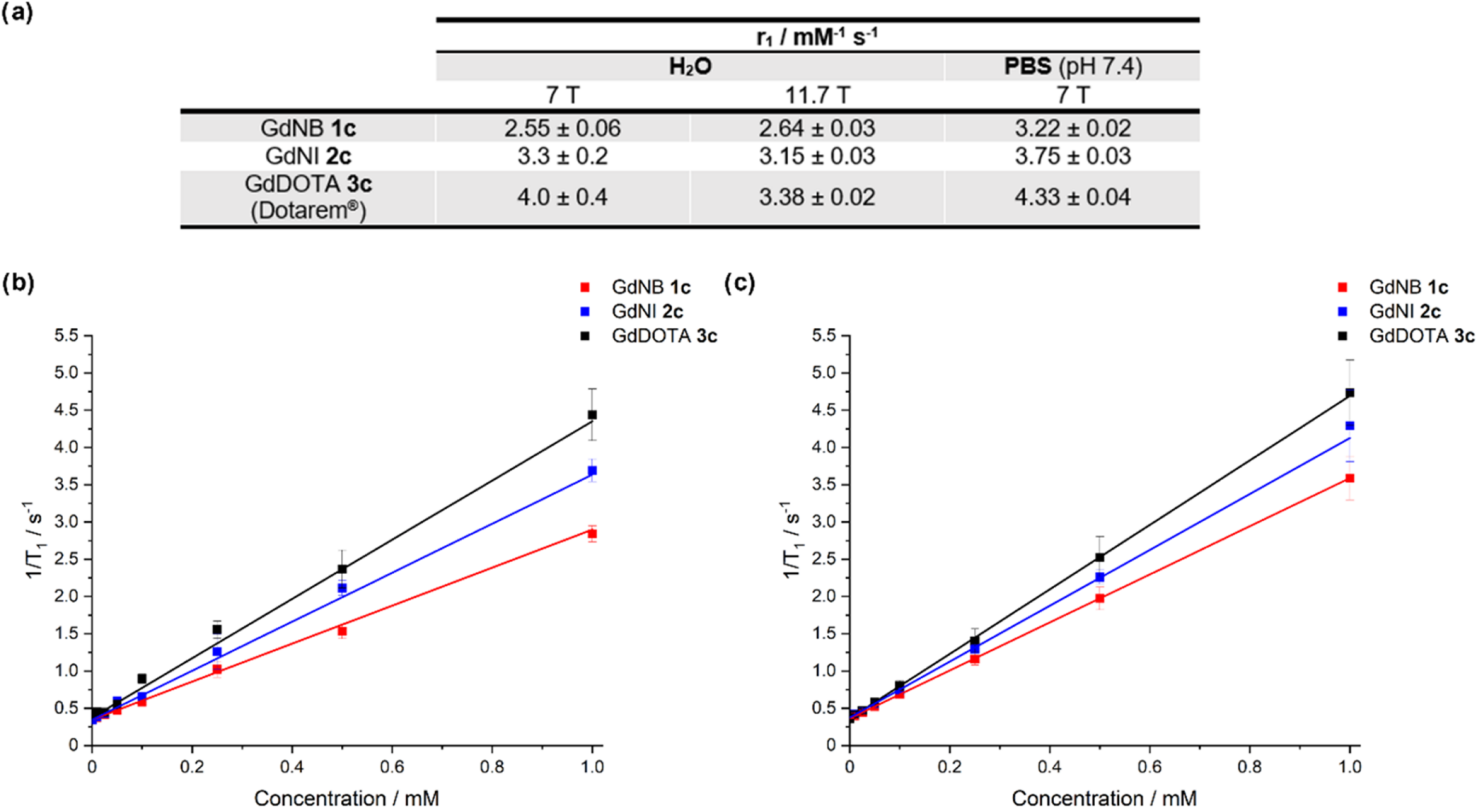
(a) Table of the relaxivity data obtained in water and PBS (pH 7.4) at 11.7 and 7 T, where the error in r_1_ is the error in the slope from line fitting. (b) Graphical data for the T_1_ measurements of GdNB (**1c**, red), GdNI (**2c**, blue), and GdDOTA (Dotarem, black) in water at 7 T. (c) Graphical data for the T_1_ measurements of GdNB, GdNI, and GdDOTA in PBS (pH 7.4) at 7 T. Data shown as mean ± standard deviation.

We hypothesized that a change in relaxivity should be observed between the complexes, despite no change in the *q* value obtained by luminescence. This is due to the difference in symmetry, evidenced by the change in the *J* = 1 band of the europium complexes and the ^1^H NMR spectra, which impacts the rate of molecular tumbling (τ_r_, eq S3). Initial relaxivity measurements for the gadolinium complexes were carried out on an 11.7 T instrument in H_2_O (Figure S22) and showed that, under these conditions, both the GdNB (**1c**) and GdNI (**2c**) exhibit slightly lower relaxivities than GdDOTA (Figure 4a, 2.64 and 3.15 mM^–1^ s^–1^, respectively, compared to 3.38 mM^–1^ s^–1^ for GdDOTA, (**3c**), consistent with the trend previously reported by Nazaré and co-workers;^7^ therefore, an increase in MRI signal would be observed upon activation and conversion. These measurements were repeated on a more clinically relevant 7 T instrument and showed the same trend in water (Figure 4b). Further measurements were carried out in PBS (pH 7.4, Figure 4c) and showed that a smaller difference in relaxivity between the complexes is observed; this may be due to the impact of phosphate binding. A small change in relaxivity is observed between the complexes due to their structural similarity and coordination of the monoester carbonyl group to gadolinium.

### Chemical Activation of Lanthanide Complexes

To determine whether the NB and NI complexes may be activated to the DOTA complexes as proposed, the complexes were subjected to a zinc chemical activation assay using an adapted method previously used to study the activation of bioreductive prodrugs and profluorophores.^5^ The first linear sweep, scanning cathodically, of the cyclic voltammogram (Figure S23 and Table S7) was used to determine the reduction onset potentials of nitro reduction prior to carrying out activation assays to support the proposed trend in activation and confirmed, in agreement with the previous literature, that it is more thermodynamically favorable to reduce the NI complex than the NB complex (−0.236 V, vs SHE, for TbNI at pH 7.4, compared to −0.356 V for TbNB) and that this is more favorable at a lower pH (pH 6.0 vs pH 7.4). No electrochemical activity was observed for the TbBn control; therefore, the onset potential can be attributed to the reduction of the nitro moiety.

**Figure 5:**
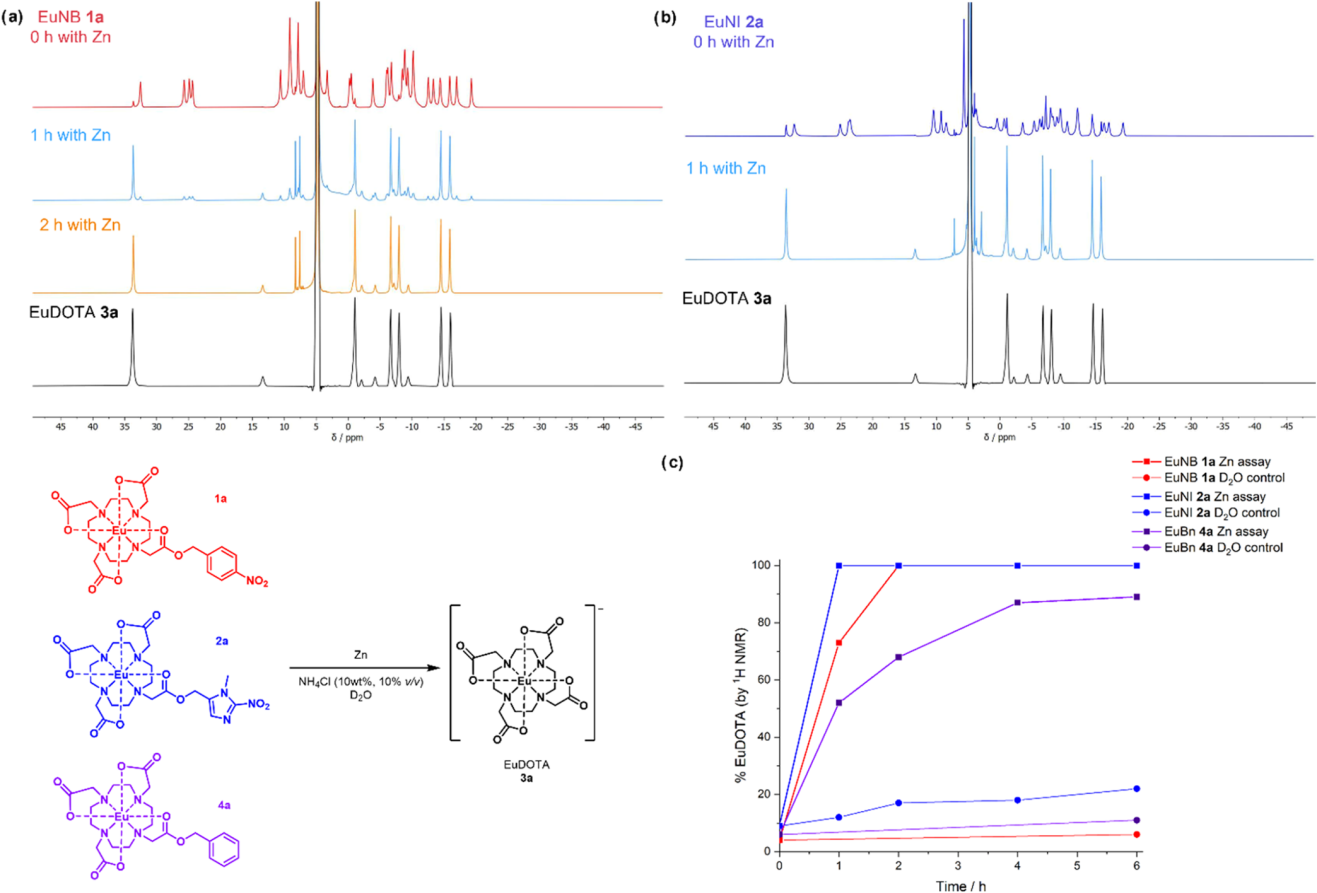
(a) ^1^H NMR spectra of the time points (1 h light blue, 2 h orange) from the zinc assay of EuNB (**1a**, red, 0 h 4% EuDOTA) in D_2_O in comparison to the positive control EuDOTA (**3a**, black). (b) ^1^H NMR spectra of the time points from the zinc assay of EuNI (**2a**, dark blue, 0 h 9% EuDOTA) in D_2_O in comparison to the positive control EuDOTA. The EuDOTA control ^1^H NMR was in D_2_O only. (c) Graph comparing the release of EuDOTA under the zinc reduction conditions for EuNB (**1a**), EuNI (**2a**), and EuBn (**4a**, purple) and the release in D_2_O only. The stability of EuNI in D_2_O only and with NH_4_Cl was monitored by ^1^H NMR in addition to the zinc reduction assay (Figure S33).

The chemical activation assay was monitored by ^1^H NMR spectroscopy of the europium analogues, as the difference in spectra between the NB/NI complexes and the DOTA complex was readily observed (Figures S18–S19) due to the change in symmetry from C_1_ to C_4_. Figure 5a shows that EuNB may be fully reduced to EuDOTA in the presence of zinc under 2 h (with 70% conversion observed after 1 h) and that EuNI (Figure 5b) may be analogously reduced under 1 h. The reduction was confirmed by ESI mass spectrometry (Figures S24–S25), and it was confirmed that significant degradation/hydrolysis to EuDOTA (in clinically relevant times) is not observed in the absence of zinc and ammonium chloride under the assay conditions (Figures S26–S27: 6% EuDOTA was observed in the ammonium chloride control for EuNB after 6 h and 20% EuDOTA for EuNI). The ^1^H NMR spectra (Figure S28a) of the EuNB assay suggest that the formation of EuDOTA in the presence of zinc may occur by ester cleavage rather than the mechanism of nitro reduction and cleavage due to the presence of nitrobenzyl alcohol and absence of aminobenzyl alcohol after 2 h under the zinc reduction conditions (where full conversion to EuDOTA is observed). Evaluation of the control EuBn (**4a**) under zinc reduction conditions (Figures S29–S32) showed that EuDOTA is produced by ester cleavage, with a slower release of EuDOTA compared to EuNB and EuNI (Figure 4c). These results suggest that, under the chemical reduction conditions, the europium ester complexes are activated to form EuDOTA, predominantly by a Lewis acid-mediated ester cleavage mechanism (due to the absence of cleaved amino aryl alcohols by ^1^H NMR) rather than the nitro reduction and cleavage mechanism, with the rate of activation dependent on the ester arm (NI > NB > Bn).

### Enzymatic Activation of Lanthanide Complexes

Prior to conducting enzymatic assays, stability tests for the complexes in water (Figure S34) and PBS buffer at pH 7.4 (Figures S35–S36) were carried out to demonstrate that the complexes are suitably stable over biologically relevant time periods (up to 6 h), with evidence of partial conversion to DOTA over time in solution occurring more readily in PBS buffer than in water. Consistent with our findings during the chemical reduction assay, the nitrobenzyl complexes are more stable in aqueous solutions than the nitroimidazole complexes.

Nitroreductase from *Escherichia coli* was used to evaluate the activation of the NB complexes to determine whether activation can be observed under biologically relevant conditions. The activation of GdNB (**1c**, at 200 μM) was monitored in the presence of nitroreductase enzyme (>100 units/mg, ∼32 μg/mL) and NADH (500 μM) in NaCl solution and water under aerobic conditions. The results were analyzed by analytical HPLC (Figures S37–S41) and suggest that in the presence of nitroreductase and NADH, GdNB (retention time 8.0 min) is converted to the DOTA complex (not observed by HPLC or LRMS) and nitrobenzyl alcohol (retention time 9.7 min) under 2 h (Figures S37–S38); therefore, ester cleavage occurs at a similar rate, as observed in the chemical reduction assay. A small amount of nitrobenzyl alcohol release is observed in the nitroreductase-only control (Figure S39) and in the presence of NADH only (30% after 6 h, Figure S40). These results suggest that NADH alone may accelerate the rate of activation of the NB complexes, albeit more slowly than the nitroreductase enzyme. Activation by NADH alone was further confirmed by a ^1^H NMR study after 6–24 h incubation of EuNB (10 mM) and NADH (25 mM) at 37 °C (Figures S42–S43), showing the formation of EuDOTA and nitrobenzyl alcohol.

Activation of the europium complexes was further investigated using a nickel–iron hydrogenase enzyme Hyd-1 (from *E. coli*) adsorbed onto a carbon black support (Figure S44, Hyd-1/C), which is known to reduce nitroaromatics to the corresponding aniline, following an adapted version of the procedure reported by Sokolova and co-workers.^46^ This enzyme was utilized to further validate the mechanism of activation, as we hypothesized that ester cleavage may occur due to the similarity to reaction conditions commonly used to deprotect benzyl groups (Pd/C with hydrogen). Total conversion to EuDOTA (**3a**) from EuNB (**1a**) was observed after 2 h in the presence of the enzyme (by ^1^H NMR spectroscopy, Figure S45), in comparison to 50% conversion to EuDOTA observed in the negative control (without the hydrogenase enzyme on carbon present). As observed during the chemical reduction assay, ^1^H NMR spectroscopy (Figure S46) shows dominantly the presence of nitrobenzyl alcohol after 2 h and the absence of aminobenzyl alcohol—production of aminobenzyl alcohol was only observed by ^1^H NMR spectroscopy after 4 h, likely due to the enzymatic reduction of the cleaved compound. EuNI (**2a**) was evaluated under the same conditions; however, full conversion/degradation to EuDOTA was observed after 1 h in the absence of the enzyme (Figure S47). Despite the elevated level of activation observed in the controls, the level of activation of EuNI or EuNB observed in the presence of the hydrogenase enzyme matches the rate of conversion to DOTA observed in the nitroreductase enzyme assay or under chemical reduction conditions. In contrast to the chemical reduction conditions, little conversion of EuBn to EuDOTA (6% after 4 h) was observed under the assay conditions (Figure S49), similar to that observed in the control (in the absence of Hyd-1/C), suggesting that the hydrogenase enzyme selectively activates the nitro-aromatic esters.

Despite the desirable change in spectroscopic and relaxometric properties and activation observed for these complexes, no clear evidence of cell permeability was observed for the terbium complexes; work is currently underway to improve both the stability and the cell permeability of activatable lanthanide complexes to achieve practical application *in vivo*.

## Conclusions

The europium, terbium, and gadolinium complexes of the mononitrobenzyl (NB) and nitroimidazole (NI) esters of DOTA have been synthesized and characterized. The terbium complexes exhibit an increase in luminescence (upon direct metal excitation) from the NB/NI complexes upon reductive conversion to the parent DOTA complex for both steady-state and time-gated emission (albeit to the weakly emitting DOTA complexes), while the gadolinium analogues may be activated to cause a change in MRI contrast. Chemical activation of the europium complexes to the desired DOTA complexes was confirmed by ^1^H NMR spectroscopy and MS. Under these conditions, the activation is proposed to occur dominantly by Lewis acid-mediated ester hydrolysis rather than the nitro reduction and cleavage pathway, with differing rates of activation observed for the NB, NI, and Bn complexes. These findings were corroborated by the enzymatic studies, showing that in the presence of nitroreductase and hydrogenase enzymes, the nitrobenzyl lanthanide complexes may be activated to give the corresponding DOTA complexes and nitrobenzyl alcohol. This shows that while the complexes may be activated to give DOTA and cause a change in their spectroscopic and relaxometric properties, further optimization is required to increase their suitability for probing reductive environments due to the possibility of activation via mechanisms other than selective nitro reduction and cleavage. The cleavage of ester groups *in vivo* occurs slowly, and future work could focus on the use of more readily cleaved esters (to enhance the change in signal vs background activation) or the use of reductively activated groups attached by using more hydrolytically stable ether or amide moieties. In a clinical context, these considerations would have to be balanced with the need to retain sufficient stability to allow for localization in appropriate tissue. Nevertheless, this work highlights the versatility and potential applications of these complexes in both optical and MRI imaging, paving the way for further development and refinement of such systems.

## Experimental Section

The details of the experimental section, including synthesis and characterization of all compounds, relevant ^1^H NMR spectra and HPLC traces, and UV–visible and luminescence spectra, are given in the Supporting Information.

## Supporting Information Available

The following files are available free of charge: Synthesis and characterization of compounds; UV–vis absorption and luminescence spectra; cyclic voltammograms; supplementary assay data; NMR spectra; and HPLC traces (PDF)
