# Supplementary material for: LnDOTA Releasing Probes for Luminescence and Magnetic Resonance Imaging
Source: Inorg Chem. 2025 Mar 24;64(13):6640–7. doi: 10.1021/acs.inorgchem.5c00199 (PMC11979886; doi:10.1021/acs.inorgchem.5c00199)
Supplement: Supplementary file 1 — ic5c00199_si_001.pdf [file ic5c00199_si_001.pdf]

Supporting Information for

LnDOTA releasing probes for luminescence and magnetic resonance imaging

Ceri A. Foster,[1] Deborah Sneddon,[1,2] Lina Hacker,[3] Euan T. Sarson,[1] Max Robertson,[1] Daria Sokolova,[1] Louise A. W. Martin,[3] Matthew F. Allen,[1] Alexandr Khrapichev,[3] Kylie A. Vincent,[1] Ester M. Hammond,[3] Stuart J. Conway,[1,4*] Stephen Faulkner[1*]

1) Department of Chemistry, Chemistry Research Laboratory, University of Oxford, Mansfield Road, Oxford, OX1 3TA

2) Department of Chemistry, School of Life Sciences, University of Sussex, Falmer, Brighton, BN1 9QJ

3) Department of Oncology, University of Oxford, Old Road Campus Research Building, Roosevelt Drive, Oxford, OX3 7DA

4) Department of Chemistry and Biochemistry, University of California Los Angeles, 607 Charles E. Young Drive East, Los Angeles, 90095-1569

* stuart.conway@chem.ucla.edu; stephen.faulkner@keble.ox.ac.uk

Supplementary Figures 3
UV-Visible absorption spectra 4
Steady-state emission spectra (ligand excitation) 5
Excitation spectra 7
Metal-centred luminescence 8
Time-gated emission spectra 8
Quantum yield evaluation 10
Luminescence lifetime measurements 10
^{1}H NMR spectra of lanthanide complexes 15
TbNI degassing luminescence experiments 17
Relaxivity measurements 18
Electrochemical measurements 19
Chemical reduction assay 21
Stability data 30
Nitroreductase enzyme assays 33
NADH NMR study 37
Hydrogenase enzyme assays 39
General Experimental 45

Chemistry Experimental 45
Relaxivity measurements.................... 54
Cyclic voltammetry (CV) 55
Chemical activation assays 56
Stability measurements 58
Nitroreductase enzymatic assay.................... 59
NADH NMR study.................... 61
Hyd-1/C enzymatic assay 62
Biological Experimental 63
Synthesis of Compounds 64
Tri-*tert*-butyl 2,2',2''-(1,4,7,10-tetraazacyclododecane-1,4,7-triyl)triacetate hydrobromide, 5........ 64
4’-Nitrobenzyl 2-bromoacetate, 6.................... 64
Tri-*tert*-butyl 2,2',2''-(10-(2-((4-nitrobenzyl)oxy)-2-oxoethyl)-1,4,7,10-tetraazacyclododecane-1,4,7-triyl)triacetate, 8 65
2,2',2''-(10-(2-((4-Nitrobenzyl)oxy)-2-oxoethyl)-1,4,7,10-tetraazacyclododecane-1,4,7-triyl)triacetic acid, 10 66
General lanthanide complexation procedure 68
Europium nitrobenzyl complex (EuNB), 1a.................... 68
Terbium nitrobenzyl complex (TbNB), 1b 68
Gadolinium nitrobenzyl complex (GdNB), 1c.................... 69
Ethyl 2-amino-1-methyl-1*H*-imidazole-5-carboxylate, S1 70
Ethyl 1-methyl-2-nitro-1*H*-imidazole-5-carboxylate, S2 70
(1-Methyl-2-nitro-1*H*-imidazol-5-yl)methanol, S3.................... 71
(1-Methyl-2-nitro-1*H*-imidazol-5-yl)methyl 2-bromoacetate, 7 72
Tri-*tert*-butyl 2,2',2''-(10-(2-((1-methyl-2-nitro-1H-imidazol-5-yl)methoxy)-2-oxoethyl)-1,4,7,10-tetraazacyclododecane-1,4,7-triyl)triacetate, 9.................... 72
2,2',2''-(10-(2-((1-Methyl-2-nitro-1*H*-imidazol-5-yl)methoxy)-2-oxoethyl)-1,4,7,10-tetraazacyclododecane-1,4,7-triyl)triacetic acid, 11.................... 73
Europium nitroimidazole complex (EuNI), 2*a* 74
Terbium nitroimidazole complex (TbNI), 2b.................... 75
Gadolinium nitroimidazole complex (GdNI), 2c 75
EuDOTA, 3a 76
TbDOTA, 3b.................... 76
Tri-*tert*-butyl 2,2',2''-(10-(2-(benzyloxy)-2-oxoethyl)-1,4,7,10-tetraazacyclododecane-1,4,7-triyl)triacetate, S4.................... 77
2,2',2''-(10-(2-(benzyloxy)-2-oxoethyl)-1,4,7,10-tetraazacyclododecane-1,4,7-triyl)triacetic acid, S5 78
Europium benzyl complex (EuBn), 4a 79
Terbium benzyl complex (TbBn), 4b.................... 79
^{1}H and ^{13}C NMR Spectra of Novel and Final Compounds 81

LCMS/HPLC Traces of Compounds 100
Intermediates 100
Final compounds 106
References..120

Supplementary Figures

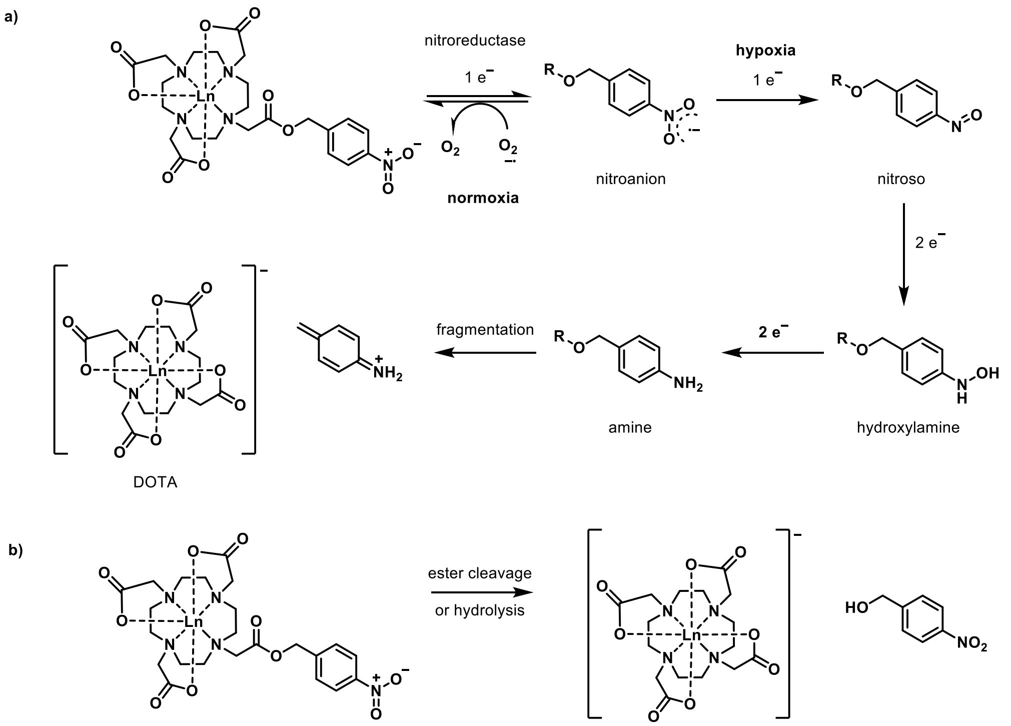

Figure S1: The proposed mechanisms of activation of the nitrobenzyl (NB) complexes to DOTA: a) by 6 one electron reductions of the nitro group followed by fragmentation, adapted from Calder *et al.*[1] and b) by ester cleavage.

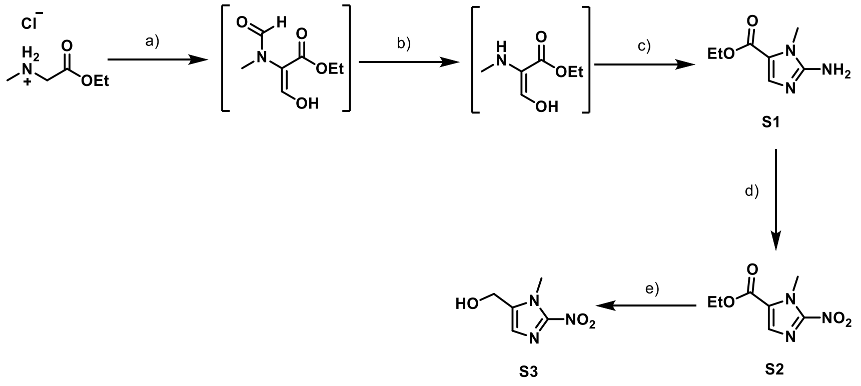

Scheme S1: The synthetic scheme for the formation of the nitroimidazole alcohol **S3**, which was carried out using optimised literature procedures.[2] *Reagents and conditions*: a) NaH, ethyl formate, dry THF, EtOH, 0 °C to rt, 3 h; b) EtOH, conc. HCl, reflux, 2 h; c) Cyanamide, EtOH, water, reflux, 1.5 h, 19-64% over 3 steps; d) $NaNO_2$, water, acetic acid, 0 °C to rt, 1.5 h, 43-61%; (v) $NaBH_4$, dry THF, MeOH, EtOH, 0 °C, 6 h, 37-53%.

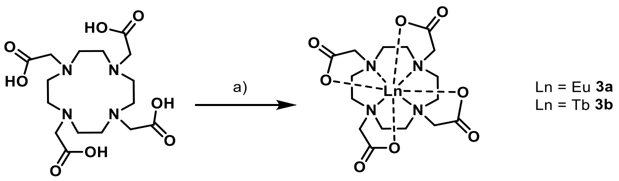

Scheme S2: The synthetic scheme for the formation of the positive control europium DOTA and terbium DOTA complexes, **3**, which was carried out using adapted literature procedures.[3, 4] *Reagents and conditions:* a) $Ln(OTf)_3$, EtOH: H_2O (1:1) or H_2O, 40 °C, 17 h, 25-46%.

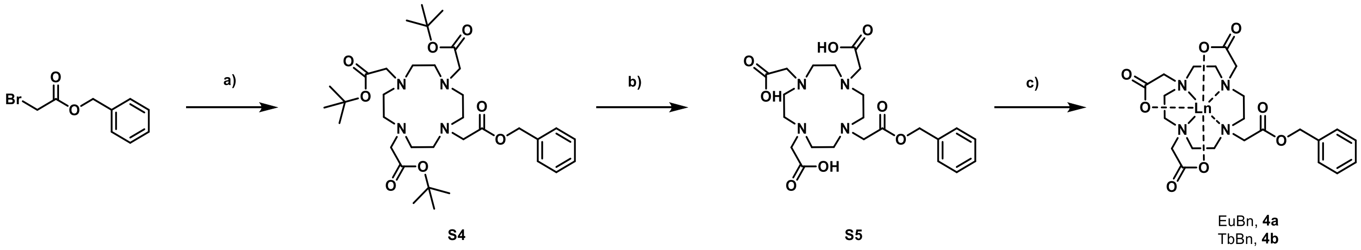

Scheme S3: The synthetic scheme for the synthesis of EuBn and TbBn, **4a-b**, to be used as a control for the reduction assays. *Reagents and conditions:* a) **5**, K_2CO_3, MeCN, rt, 3 h, 91-93%; b) TFA, CH_2Cl_2, rt, 18 h, 40-99%; c) $Ln(OTf)_3$, MES buffer (1 M, pH 6), rt, 1 h, 28-41%.

UV-Visible absorption spectra

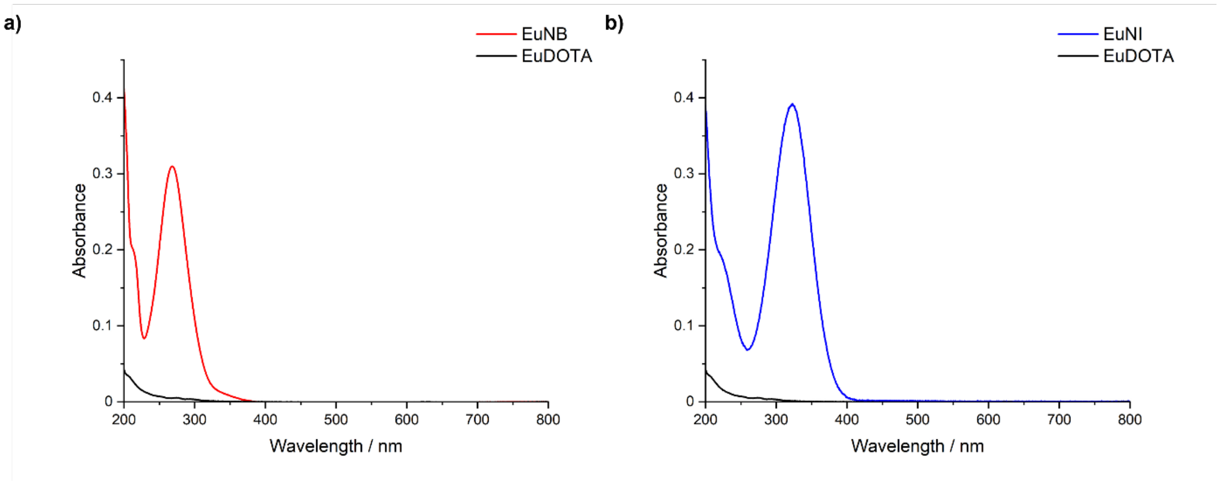

Figure S2: The UV-Vis spectra of a) EuNB (**1a**, red) in comparison to EuDOTA (**3a**, black) and b) EuNI (**2a**, blue) in comparison to EuDOTA, at 50 μM in H_2O.

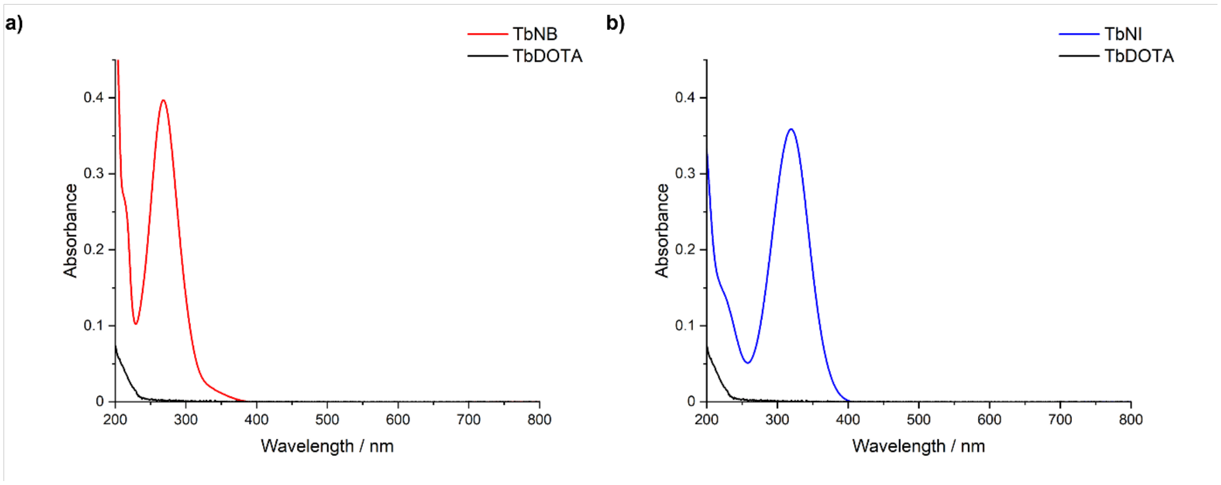

Figure S3: The UV-Vis spectra of a) TbNB (**1b**, red) in comparison to TbDOTA (**3b**, black) and b) TbNI (**2b**, blue) in comparison to TbDOTA, at 50 μM in H_2O.

Steady-state emission spectra (ligand excitation)

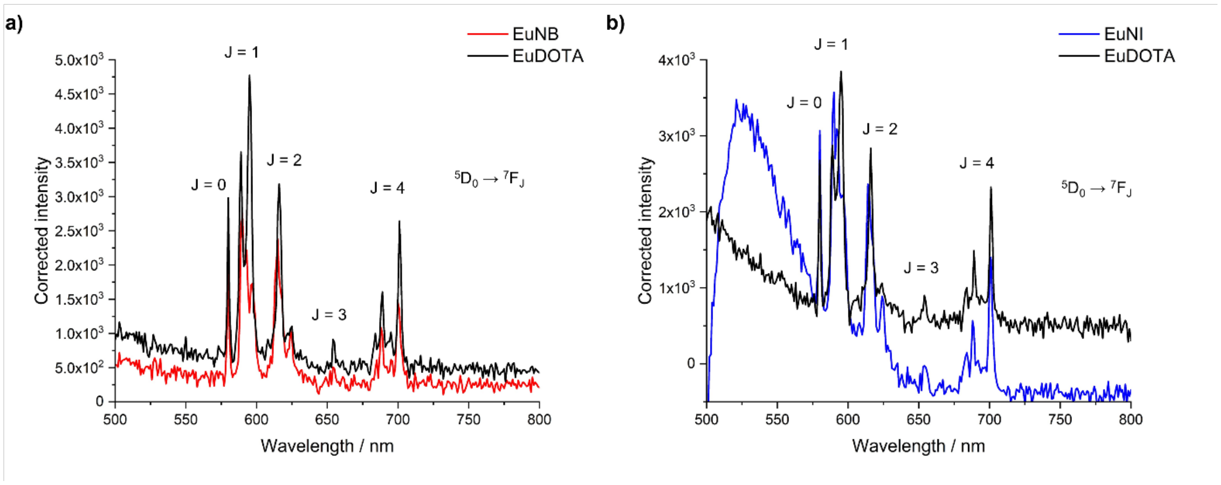

Figure S4: The steady-state emission spectra (ligand excitation, metal region) of a) EuNB (**1a**, red) in comparison to EuDOTA (**3a**, black), λ_{ex} 269 nm and b) EuNI (**2a**, blue) in comparison to EuDOTA, λ_{ex} 320 nm.

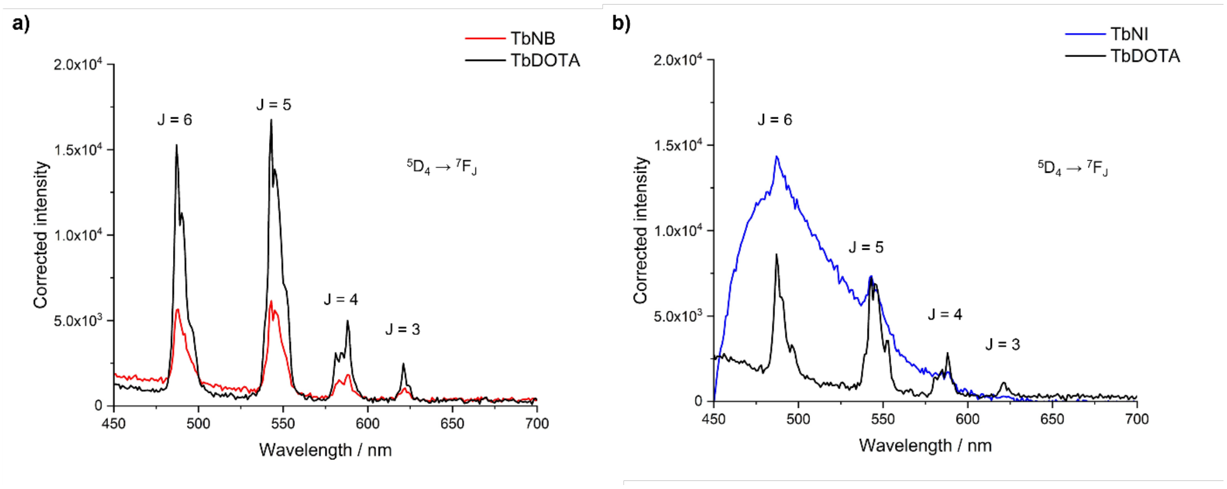

Figure S5: The steady-state emission spectra (ligand excitation, metal region) of a) TbNB (**1b**, red) in comparison to TbDOTA (**3b**, black), λ_{ex} 269 nm and b) TbNI (**2b**, blue) in comparison to TbDOTA, λ_{ex} 320 nm. The relevant transitions are labelled.

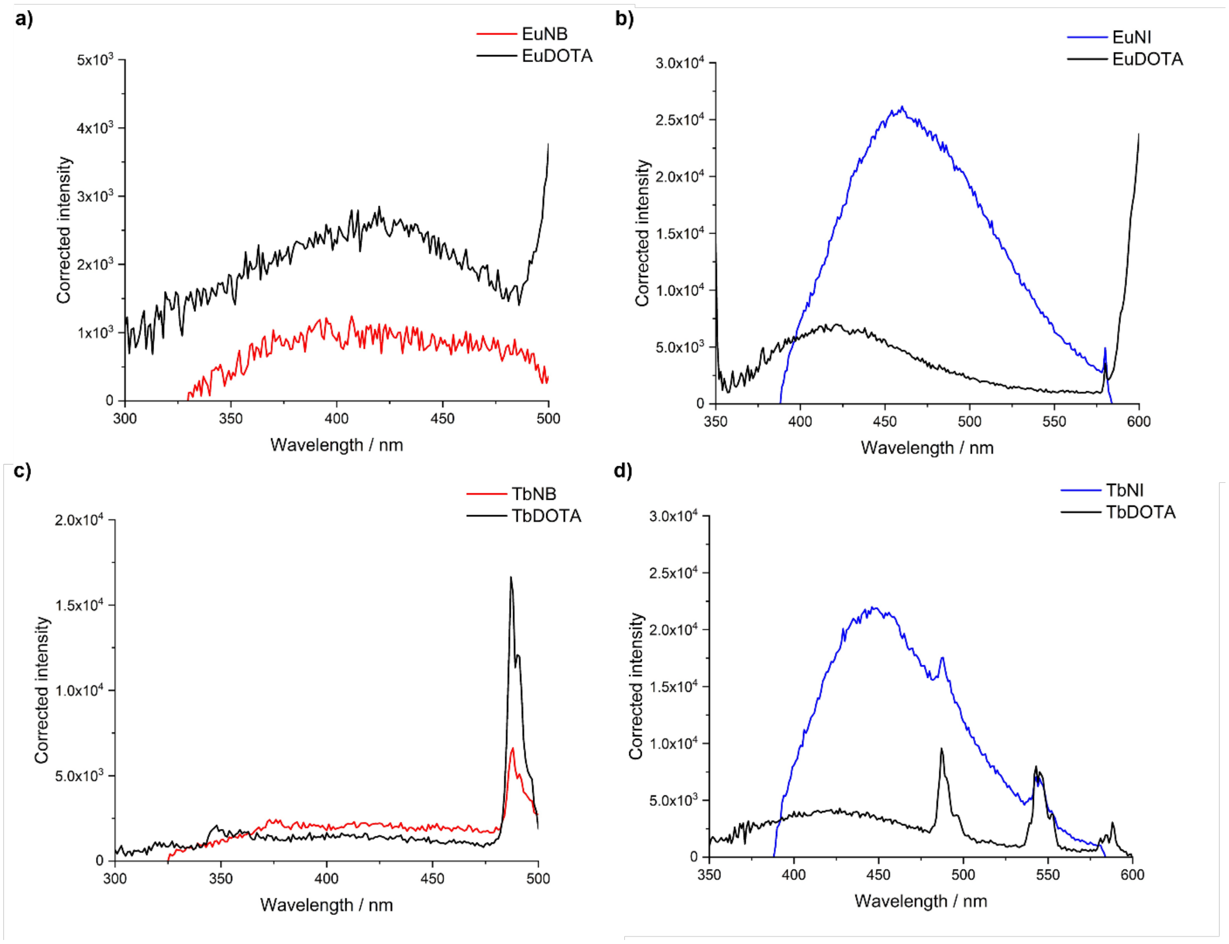

Figure S6: The steady-state emission spectra (ligand excitation, ligand region) of a) EuNB (**1a**, red) in comparison to EuDOTA (**3a**, black), λ_{ex} 269 nm, b) EuNI (**2a**, blue) in comparison to EuDOTA, λ_{ex} 320 nm, c) TbNB (**1b**, red) in comparison to TbDOTA, λ_{ex} 269 nm and d) TbNI (**2b**, blue) in compared to TbDOTA (**3b**, black), λ_{ex} 320 nm. No filters used.

Excitation spectra

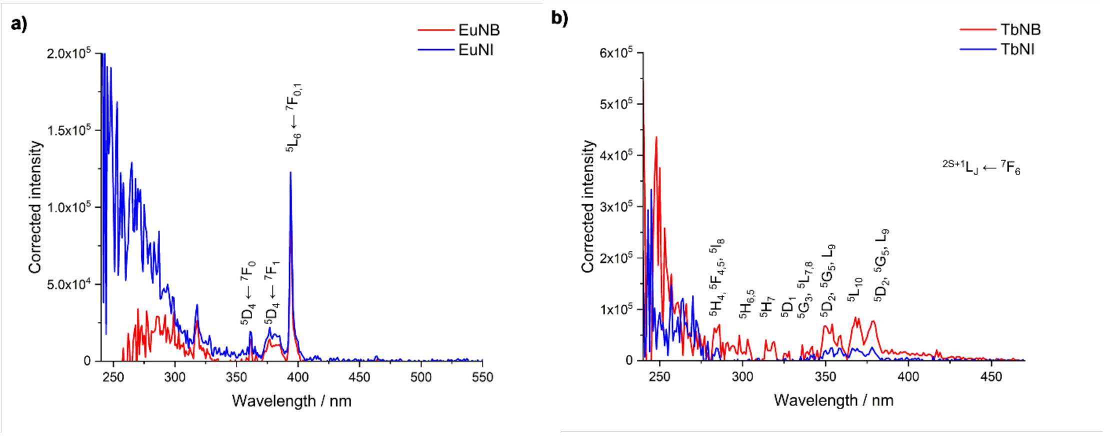

Figure S7: Excitation spectra of compounds measured at 50 μM in H_2O. a) EuNB (**1a**, red) and EuNI (**2a**, blue), λ_{em} 616 nm, λ_{ex} 240-550 nm, slits 1 / 10 nm, 495 nm long pass filter, integration time 1.0 s. The relevant transitions are labelled.[5] b) TbNB (**1b**, red) and TbNI (**2b**, blue), λ_{em} 487 nm, λ_{ex} 240-470 nm, slits 1 / 5 nm, 400 nm long pass filter, integration time 0.2 s. The relevant transitions are labelled.[6]

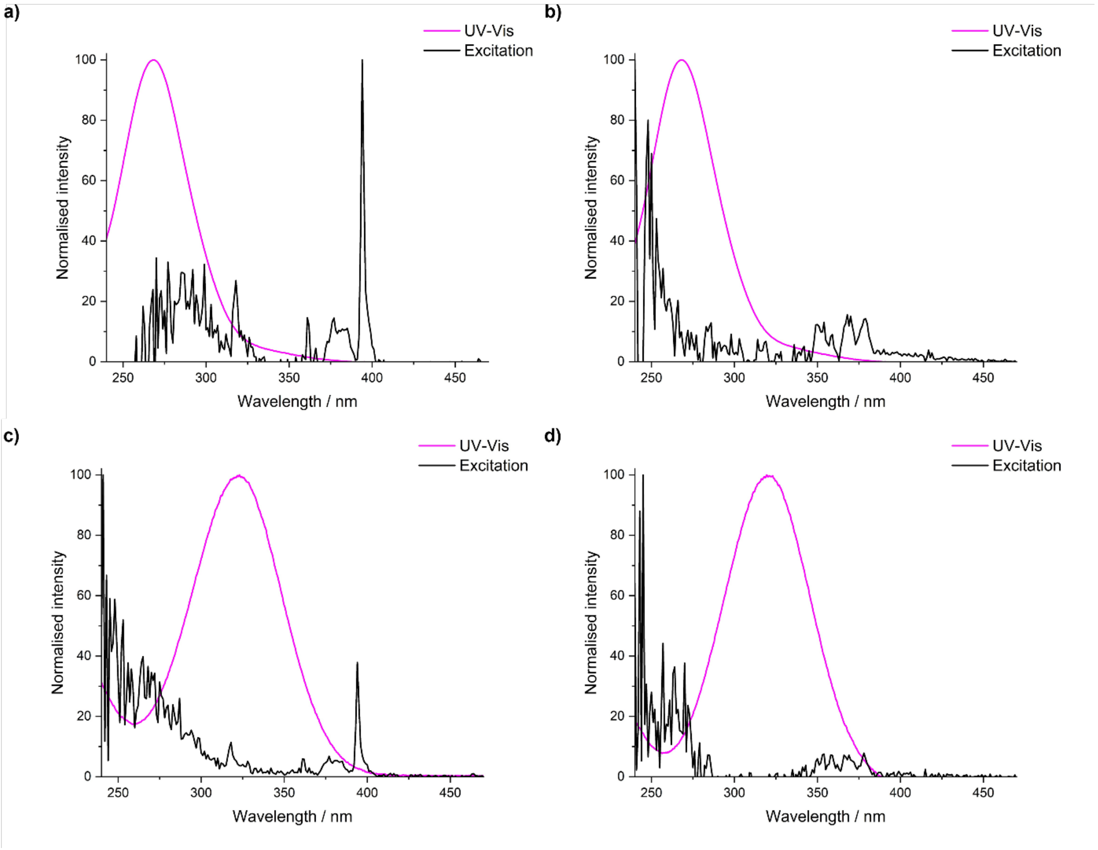

Figure S8: Normalised excitation spectra (black) compared to the normalised UV-Vis spectra (pink) of compounds measured at 50 μM in H_2O. a) EuNB (**1a**), b) TbNB (**1b**), c) EuNI (**2a**), d) TbNI (**2b**). Eu excitation spectra: λ_{em} 616 nm, slits 1 / 10 nm, integration time 0.2 s. Tb excitation spectra: λ_{em} 487 nm, slits 1 / 5 nm, integration time 0.2 s.

Metal-centred luminescence

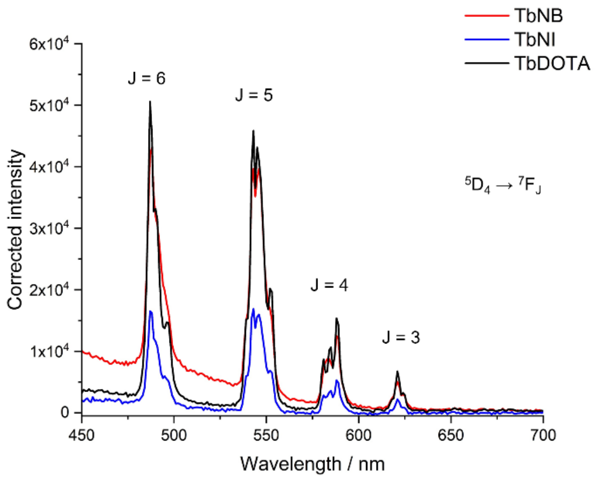

Figure S9: The steady-state emission spectra (terbium excitation) of TbNB (**1b**, red) and TbNI (**2b**, blue) in comparison to TbDOTA (**3b**, black), with λ_{ex} 366 nm. The relevant transitions are labelled.

Time-gated emission spectra

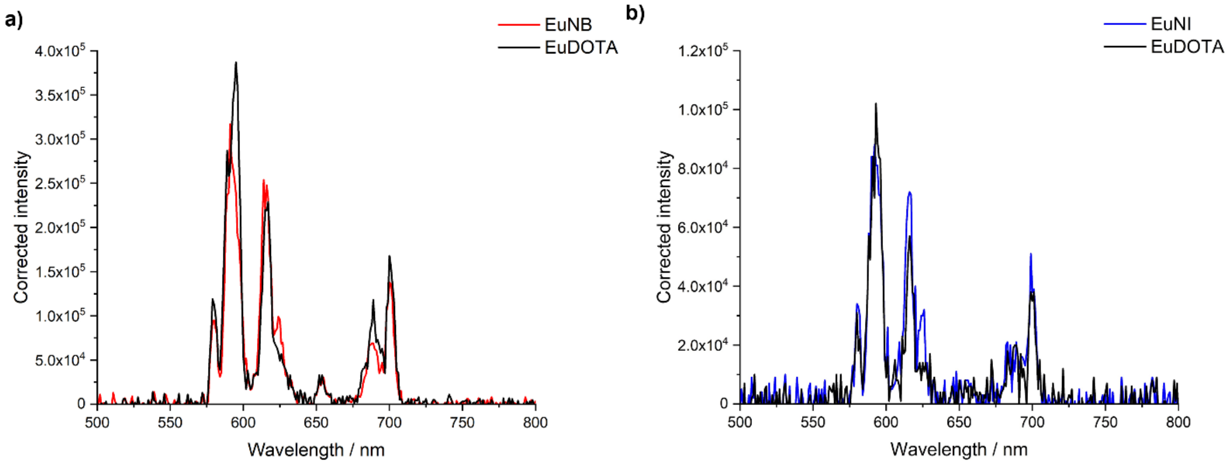

Figure S10: The time-gated emission spectra (ligand excitation) of a) EuNB (**1a**) and b) EuNI (**2a**), excited at the maximum ligand absorbance wavelength (λ_{ex} 269 nm for EuNB, red, or λ_{ex} 320 nm for EuNI, blue) in comparison to excitation of EuDOTA (**3a**, black) under the same conditions.

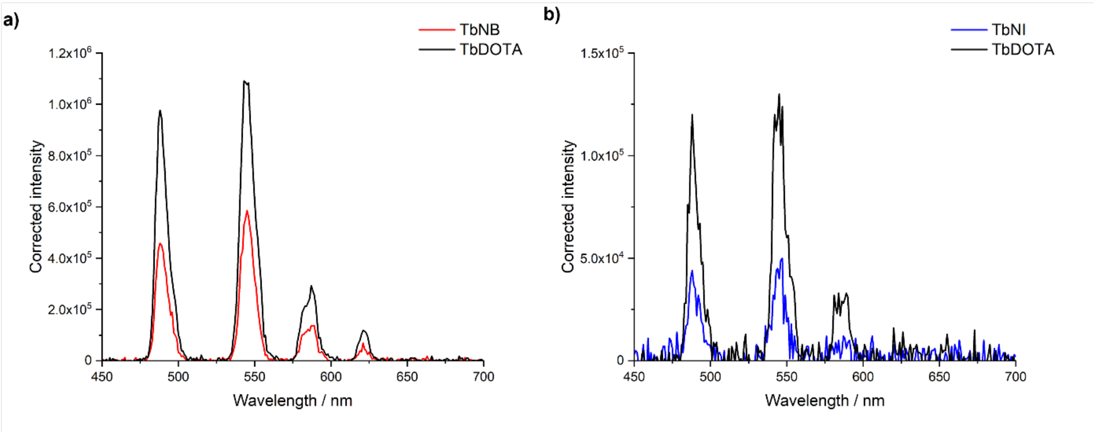

Figure S11: The time-gated emission spectra (ligand excitation) of a) TbNB (**1b**) and b) TbNI (**2b**), excited at the maximum ligand absorbance wavelength (λ_{ex} 269 nm for TbNB, red, or λ_{ex} 320 nm for TbNI, blue) in comparison to excitation of TbDOTA (**3b**, black) under the same conditions.

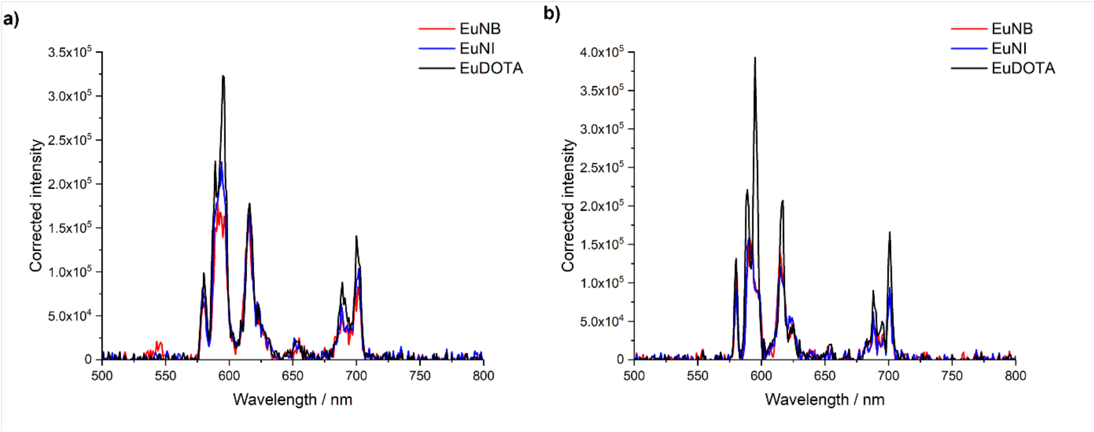

Figure S12: The time-gated emission spectra (direct metal excitation) of EuNB (**1a**, red) and EuNI (**2a**, blue) against EuDOTA (**3a**, black), with λ_{ex} 397 nm and λ_{em} 500-800 nm. a) Complexes were measured at 50 μM in H_2O. b) Complexes were measured at 200 μM in H_2O.

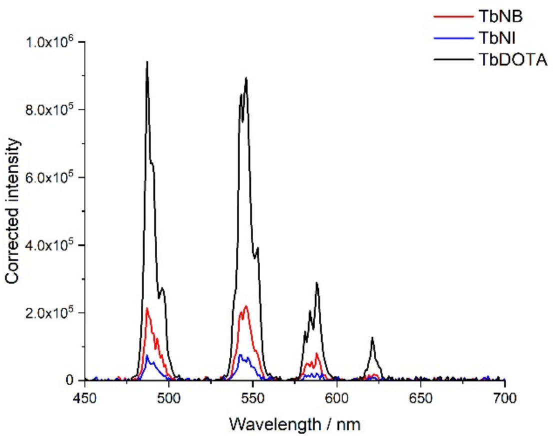

Figure S13*:* The time-gated emission spectra (direct metal excitation) of TbNB (**1b**, red) and TbNI (**2b**, blue) against TbDOTA (**3b**, black), with λ_{ex} 366 nm and λ_{em} 450-700 nm. Complexes were measured at 200 μM in H_2O.

Quantum yield evaluation

The intrinsic quantum yields (QY) of the europium complexes were calculated using their steady state emission spectra upon excitation at 397 nm (using the corrected, S1c, and baseline subtracted files) and their luminescence lifetimes (τ_{obs}), measured in water, using equations S1 and S2. n is the refractive index of the medium (1.33 for water), I_{MD} is the area of the magnetic dipole transition ($^5D_0 \rightarrow {}^7F_1$, defined here as between 583 and 604 nm) and I_{total} is the total area under the emission spectra (500 to 800 nm).[5, 7] A range of intrinsic quantum yields is reported (Table S1) for each complex, due to varying reported values of $A_{MD,0}$ (14.65, 18.45 and 20.46).[8]

$$\Phi_{Ln}^{Ln} = \frac{\tau_{obs}}{\tau_{rad}} \quad \text{(S1)}$$

$$\frac{1}{\tau_{rad}} = A_{MD,0} n^3 \left(\frac{I_{total}}{I_{MD}}\right) \quad \text{(S2)}$$

Table S1: Calculations of the intrinsic quantum yields of the europium complexes, using equations S1 and S2, with n = 1.33. * Data taken from table S3. ** Range reported due to varying reported values for $A_{MD,0}$.

Complex	I_{total}	I_{MD}	$1/\tau_{rad}$ / s^{-1}	τ_{rad} / ms	τ_{obs}* / ms	QY** / %
EuNB, **1a**	932209	214193	209	4.77	0.63	9-13
EuNI, **2a**	1461723	264666	266	3.76	0.61	11-16
EuDOTA, **3a**	845799	234773	173	5.77	0.65	8-11

Relative phosphorescence quantum yields were evaluated for the terbium complexes, using the time-gated emission spectra upon excitation at 366 nm (using the corrected, S1c, and baseline subtracted files), evaluating the area under the graph (from 450 to 700 nm) of the complexes measured under the same conditions, relative to TbDOTA (Table S2). TbDOTA is known to be a poor fluorophore upon direct excitation, by analogy to EuDOTA.

Table S2: Relative quantum yield calculations for the terbium complexes, based on the area under the graph (450 to 700 nm) of the time-gated emission spectra upon excitation at 366 nm, measured under the same conditions for all complexes.

Complex	Area / 10^7	Relative QY / %
TbNB, **1b**	7.80	43
TbNI, **2b**	3.37	21
TbDOTA, **3b**	1.60	100

Luminescence lifetime measurements

Luminescence lifetimes for both the europium and terbium complexes were recorded in H_2O and D_2O in order to calculate the number of bound water molecules, q, according to the modified Horrocks' equation (equation S3), where A is the inner-sphere contribution, B is the outer-sphere contribution and τ is the rate of decay in the specified solvent.[9, 10] The luminescence lifetimes of Eu and Tb complexes have an experimental error of ± 10%.[10] For europium complexes, A = 1.2 ms and B = 0.25 ms. For terbium complexes, A = 5.0 ms and B = 0.06 ms.

$$q = A\left(\frac{1}{\tau_{H_2O}} - \frac{1}{\tau_{D_2O}} - B\right) \quad \text{(S3)}$$

Table S3: Analysis of the luminescence lifetimes (by direct excitation, λ_{ex} 397 nm, λ_{em} 700 nm) of EuDOTA (**3a**), EuNB (**1a**) and EuNI (**2a**), to calculate their q values using the modified Horrocks' equation (equation S1), measured at 200 μM.

	**EuDOTA, 3a**		**EuNB, 1a**		**EuNI, 2a**	
	τ_{H2O} / ms	τ_{D2O} / ms	τ_{H2O} / ms	τ_{D2O} / ms	τ_{H2O} / ms	τ_{D2O} / ms
**n=1**	0.62	2.37	0.63	2.20	0.61	2.46
**n=2**	0.66	2.32	0.62	2.16	0.61	2.43
**n=3**	0.66	2.30	0.63	2.20	0.61	2.45
**Average**	0.65	2.33	0.63	2.19	0.61	2.45
**q value**	**1.04**		**1.07**		**1.18**	

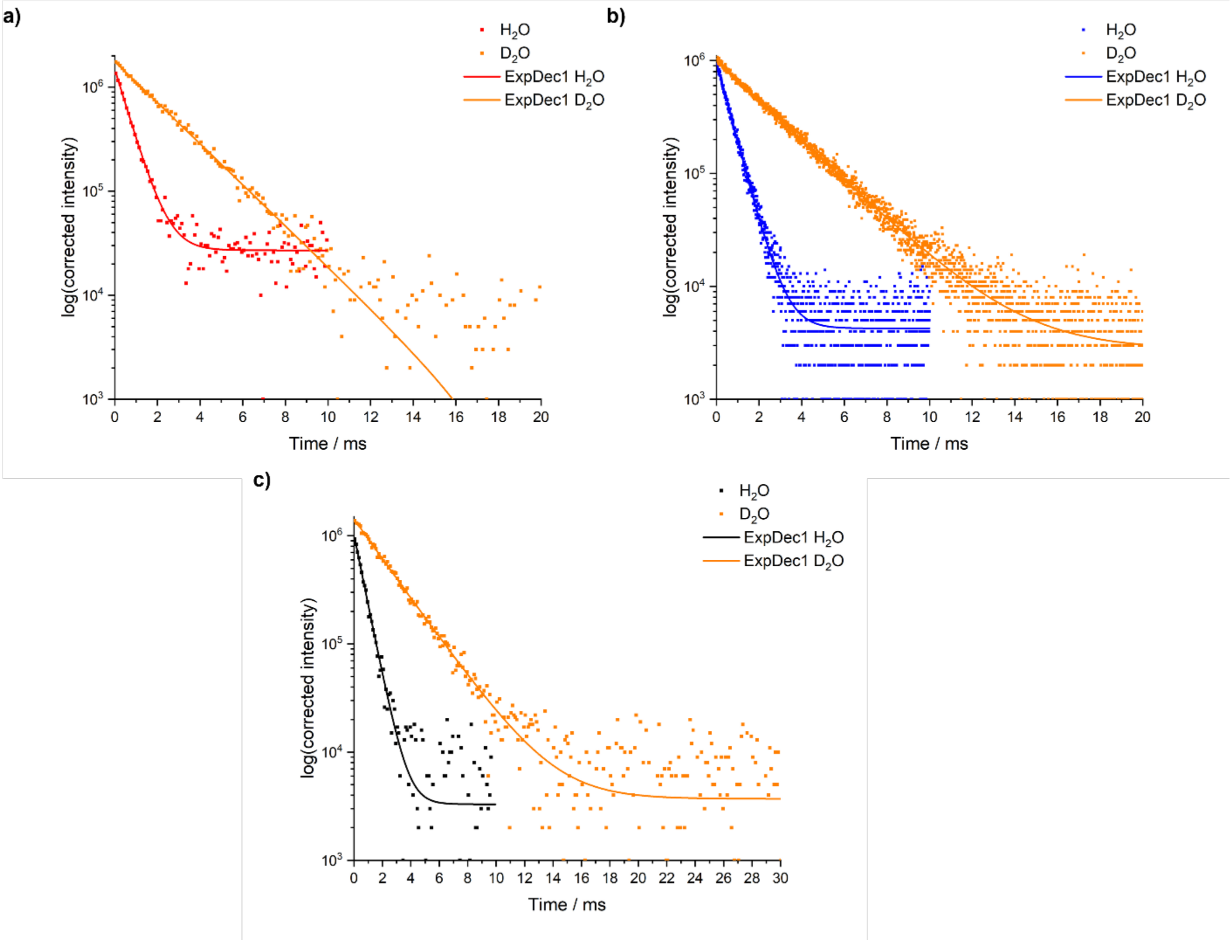

Figure S14: Representative graphs for the luminescence lifetime measurements (by direct metal excitation) of a) EuNB (**1a**), b) EuNI (**2a**) and c) EuDOTA (**3a**) at 200 μM in H_2O and D_2O, λ_{ex} 397 nm, λ_{em} 700 nm, with the corresponding exponential decay curves. Representative of n=3, sample window 1 ms, initial delay 0.05 ms, time per flash 61 ms, 495 nm long pass filter. EuNB H_2O slits 27-29 nm / 14 nm, flash count 100-102, maximum time delay 10 ms, delay increment 0.1 ms; EuNB D_2O slits 25-29 nm / 14 nm, flash count 50-52, maximum time delay 20 ms, delay increment 0.1 ms; EuNI H_2O slits 29 nm / 7-12 nm, flash count 8-10, maximum time delay 10 ms, delay increment 0.01 ms; EuNI D_2O slits 29 nm / 8-10 nm, flash count 8-10, maximum time delay 20 ms, delay increment 0.01 ms; EuDOTA H_2O slits 27-29 nm / 14 nm, flash count 50-54, maximum time delay 10 ms, delay increment 0.1 ms; EuDOTA D_2O slits 27-29 nm / 14 nm, flash count 40-44, maximum time delay 30 ms, delay increment 0.1 ms.

Table S4: Analysis of the luminescence lifetimes (by ligand excitation, λ_{ex} 269 nm for EuNB, λ_{ex} 320 nm for EuNI, λ_{em} 700 nm) of EuNB (**1a**) and EuNI (**2a**), to calculate their q values using the modified Horrocks' equation (equation S1), measured at 50 μM.

	**EuNB, 1a**		**EuNI, 2a**	
	τ_{H2O} / ms	τ_{D2O} / ms	τ_{H2O} / ms	τ_{D2O} / ms
**n=1**	0.61	2.35	0.59	2.38
**n=2**	0.61	2.42	0.59	2.40
**n=3**	0.62	2.43	0.62	2.42
**Average**	0.612	2.40	0.60	2.40
**q value**	**1.16**		**1.20**	

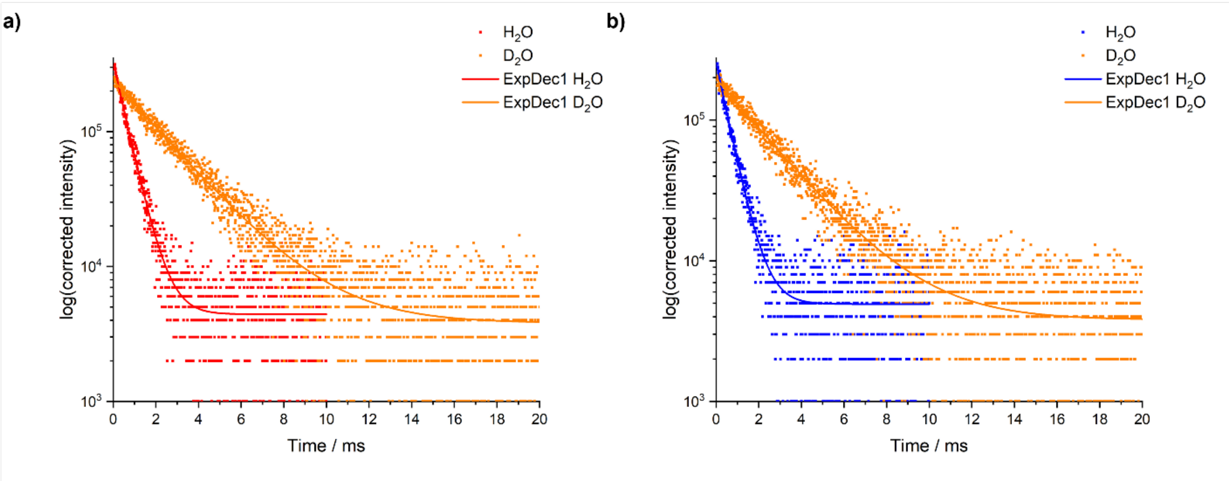

Figure S15: Representative graphs for the luminescence lifetime measurements (by ligand excitation) of a) EuNB (**1a**) and b) EuNI (**2a**) at 50 μM in H_2O and D_2O, λ_{ex} 269 nm for EuNB, λ_{ex} 320 nm for EuNI, λ_{em} 700 nm, with the corresponding exponential decay curves. Representative of n=3, sample window 1 ms, initial delay 0.05 ms, time per flash 61 ms, delay increment 0.01 ms, 495 nm long pass filter. EuNB H_2O slits 27-29 nm / 7 nm, flash count 10-12, maximum time delay 10 ms; EuNB D_2O slits 27-29 nm / 4 nm, flash count 10-12, maximum time delay 20 ms; EuNI H_2O slits 27-29 nm / 14 nm, flash count 10-12, maximum time delay 10 ms; EuNI D_2O slits 27-29 nm / 12 nm, flash count 10-12, maximum time delay 20 ms.

Table S5: Analysis of the luminescence lifetimes (by direct excitation, λ_{ex} 366 nm, λ_{em} 545 nm) of TbDOTA (**3b**), TbNB (**1b**) and TbNI (**2b**), to calculate their q values using the modified Horrocks' equation (equation S1), measured at 200 μM. TbNI appeared biexponential, therefore the two components are reported.

	**TbDOTA, 3b**		**TbNB, 1b**		**TbNI, 2b**			
					Component 1		Component 2	
	τ_{H2O} / ms	τ_{D2O} / ms	τ_{H2O} / ms	τ_{D2O} / ms	τ_{H2O} / ms	τ_{D2O} / ms	τ_{H2O} / ms	τ_{D2O} / ms
**n=1**	2.09	3.68	1.98	3.35	0.20	0.24	2.07	3.69
**n=2**	2.07	3.55	1.94	3.37	0.20	0.23	2.04	3.64
**n=3**	2.03	3.56	1.93	3.31	0.21	0.23	2.07	3.61
**n=4**	2.06	3.57	1.90	3.39	-	-	-	-
**n=5**	2.04	3.51	1.96	3.36	-	-	-	-
**Average**	2.06	3.58	1.94	3.36	0.21	0.23	2.06	3.65
**q value**	**0.73**		**0.78**		**-**		**0.76**	

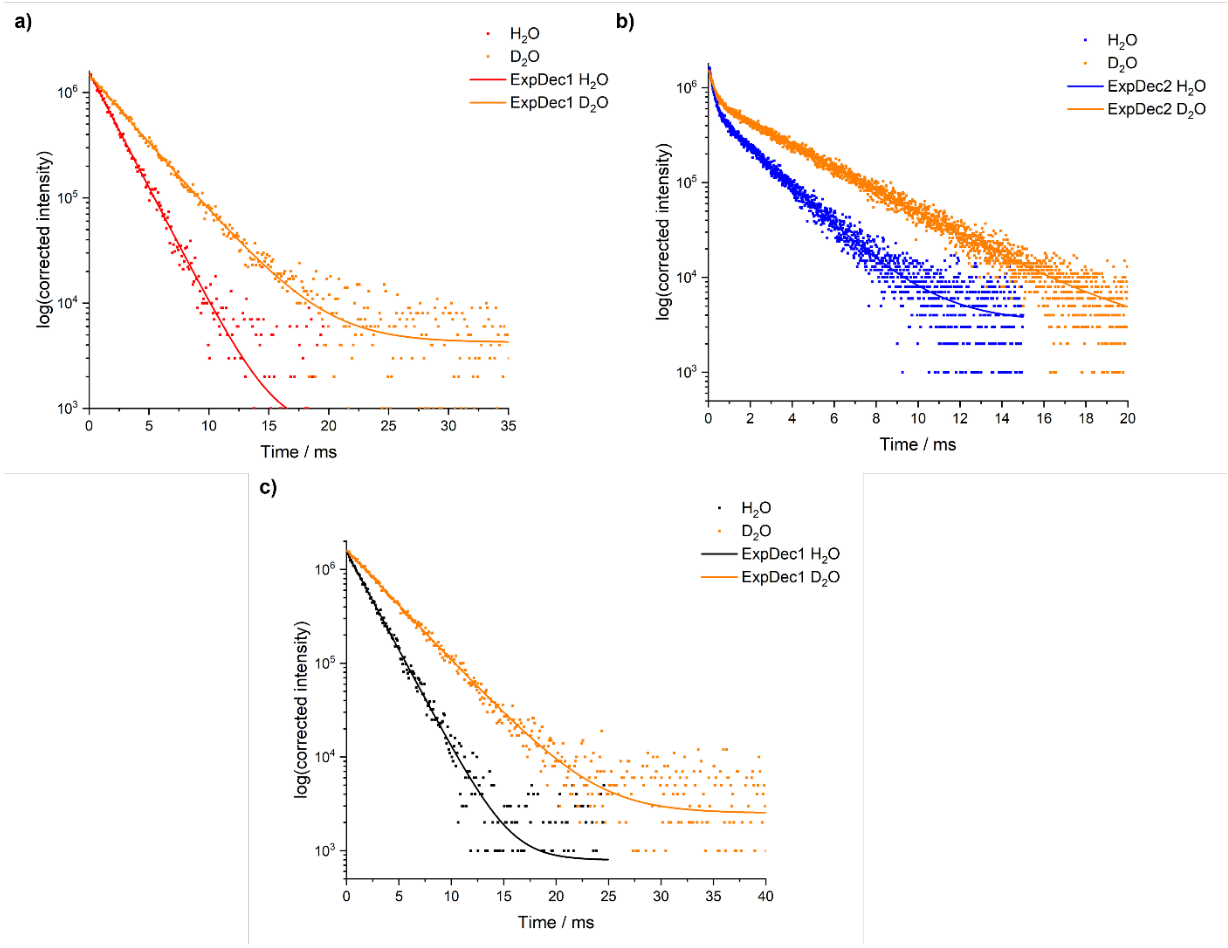

Figure S16: Representative graphs for the luminescence lifetime measurements (by direct metal excitation) of a) TbNB (**1b**), b) TbNI (**2b**) and c) TbDOTA (**3b**) at 200 µM in H_2O and D_2O, λ_{ex} 366 nm, λ_{em} 545 nm, with the corresponding exponential decay curves. Representative of n=3, sample window 1 ms, initial delay 0.05 ms, time per flash 61 ms, 400 nm long pass filter. TbNB: H_2O slits 26-29.4 nm / 14 nm, flash count 12-16, maximum time delay 20 ms, delay increments 0.1 ms; TbNB D_2O slits 25-29 nm / 14 nm, flash count 10-14, maximum time delay 35 ms, delay increments 0.1 ms; TbNI H_2O slits 29 nm / 8-10 nm, flash count 7-10, maximum time delay 15 ms, delay increments 0.01 ms; TbNI D_2O slits 29 nm / 7-9 nm, flash count 5-10, maximum time delay 20 ms, delay increments 0.01 ms; TbDOTA H_2O slits 23-28 nm / 9 nm, flash count 4-8, maximum time delay 25 ms, delay increments 0.1 ms; TbDOTA D_2O slits 23-26 nm / 9 nm, flash count 4-9, maximum time delay 40 ms, delay increments 0.1 ms.

Table S6: Analysis of the luminescence lifetimes (by ligand excitation, λ_{ex} 269 nm for TbNB, λ_{ex} 320 nm for TbNI, λ_{em} 545 nm) of TbDOTA (**3b**), TbNB (**1b**) and TbNI (**2b**), to calculate their q values using the modified Horrocks' equation (equation S1), measured at 50 µM. TbNI appeared biexponential, therefore the two components are reported.

	**TbNB, 1b**		**TbNI, 2b**			
			Component 1		Component 2	
	τ_{H2O} / ms	τ_{D2O} / ms	τ_{H2O} / ms	τ_{D2O} / ms	τ_{H2O} / ms	τ_{D2O} / ms
**n=1**	1.93	3.75	0.25	-	2.05	3.79
**n=2**	1.94	3.74	0.23	-	2.06	3.51
**n=3**	1.92	3.75	0.21	-	2.06	3.54
**Average**	1.93	3.75	0.23	-	2.06	3.61
**q value**	**0.96**		-		0.74	

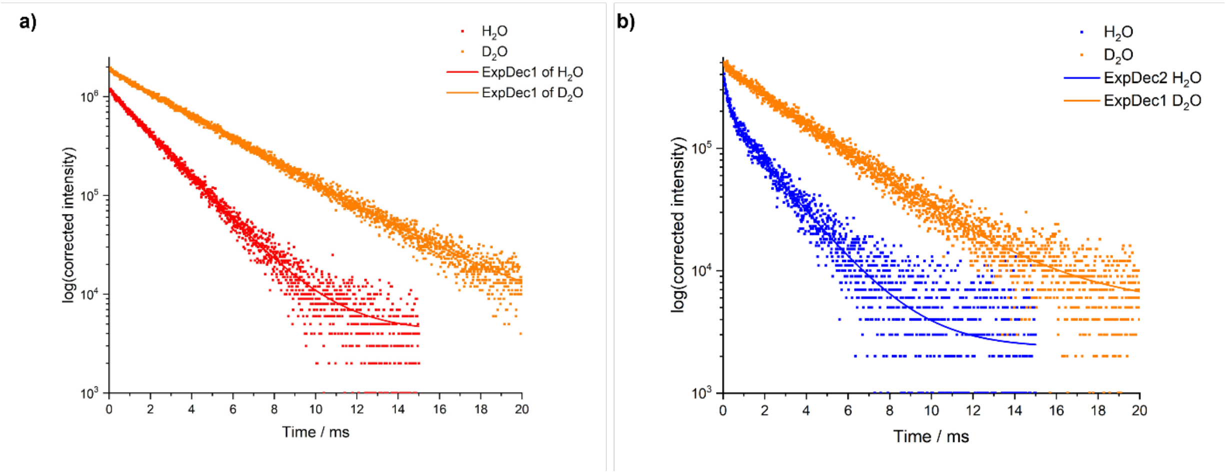

Figure S17: Representative graphs for the luminescence lifetime measurements (by ligand excitation) of a) TbNB (**1b**) and b) TbNI (**2b**) at 50 μM in H_2O and D_2O, λ_{ex} 269 nm for TbNB, λ_{ex} 320 nm for TbNI, λ_{em} 700 nm, with the corresponding exponential decay curves. Representative of n=3, sample window 1 ms, initial delay 0.05 ms, time per flash 61 ms, delay increment 0.01 ms, 400 nm long pass filter. TbNB H_2O slits 27-29 nm / 5 nm, flash count 10-12, maximum time delay 15 ms; TbNB D_2O slits 26-29 nm / 5 nm, flash count 10-12, maximum time delay 20 ms; TbNI H_2O slits 27-29 nm / 12 nm, flash count 10-12, maximum time delay 15 ms; TbNI D_2O slits 27-29 nm / 7 nm, flash count 10-12, maximum time delay 20 ms.

^{1}H NMR spectra of lanthanide complexes

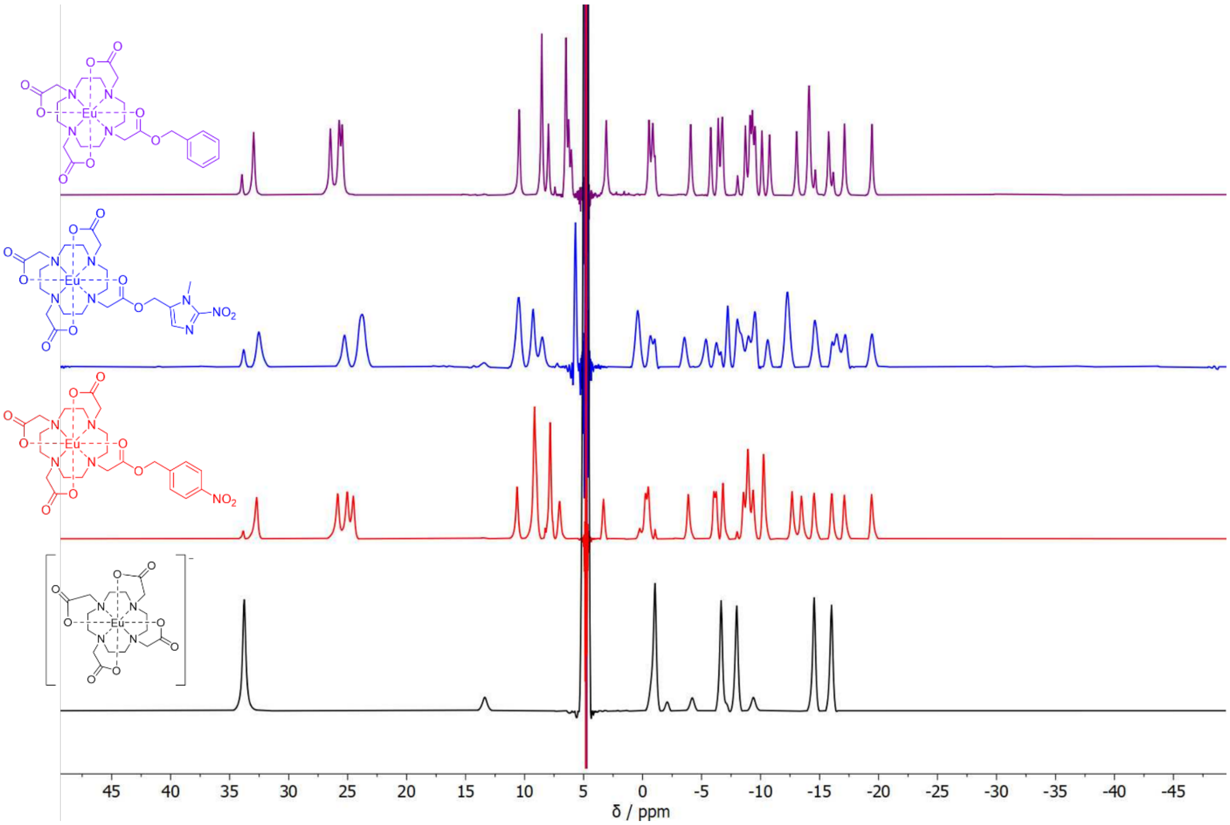

Figure S18: Stacked ^{1}H NMR spectra complexes of compounds EuBn (**4a**, purple), EuNI (**2a**, blue), EuNB (**1a**, red) and EuDOTA (**3a**, black) to highlight the difference in symmetry between the DOTA complex (C_4 symmetric, 6 peaks, TSAP and SAP isomers clearly visible) vs the nitrobenzyl/nitroimidazole/benzyl complexes (C_1 symmetric).

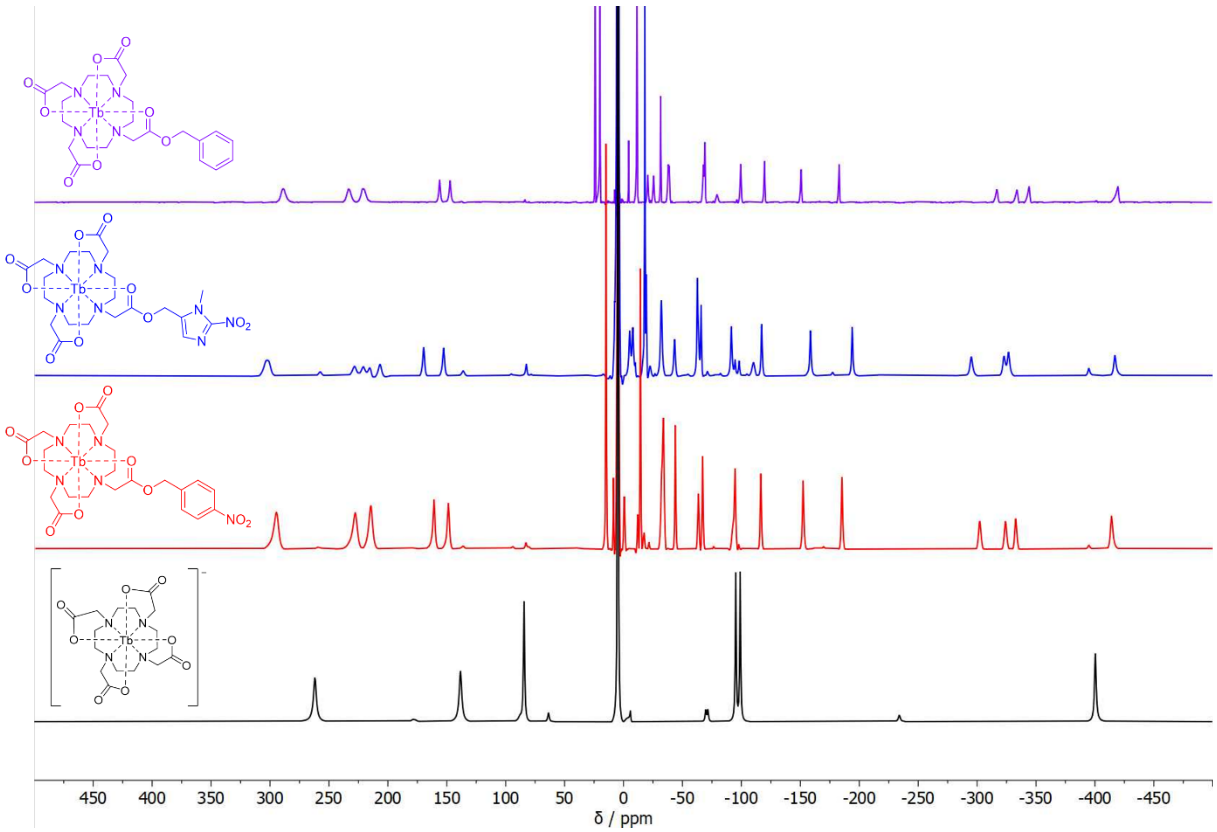

Figure S19: Stacked ^{1}H NMR spectra of compounds TbBn (**4b**, purple), TbNI (**2b**, blue), TbNB (**1b**, red) and TbDOTA (**3b**, black) to highlight the difference in symmetry between the DOTA complex (C_4 symmetric, 6 peaks, TSAP and SAP isomers clearly visible) vs the nitrobenzyl/nitroimidazole complexes (C_1 symmetric).

TbNI degassing luminescence experiments

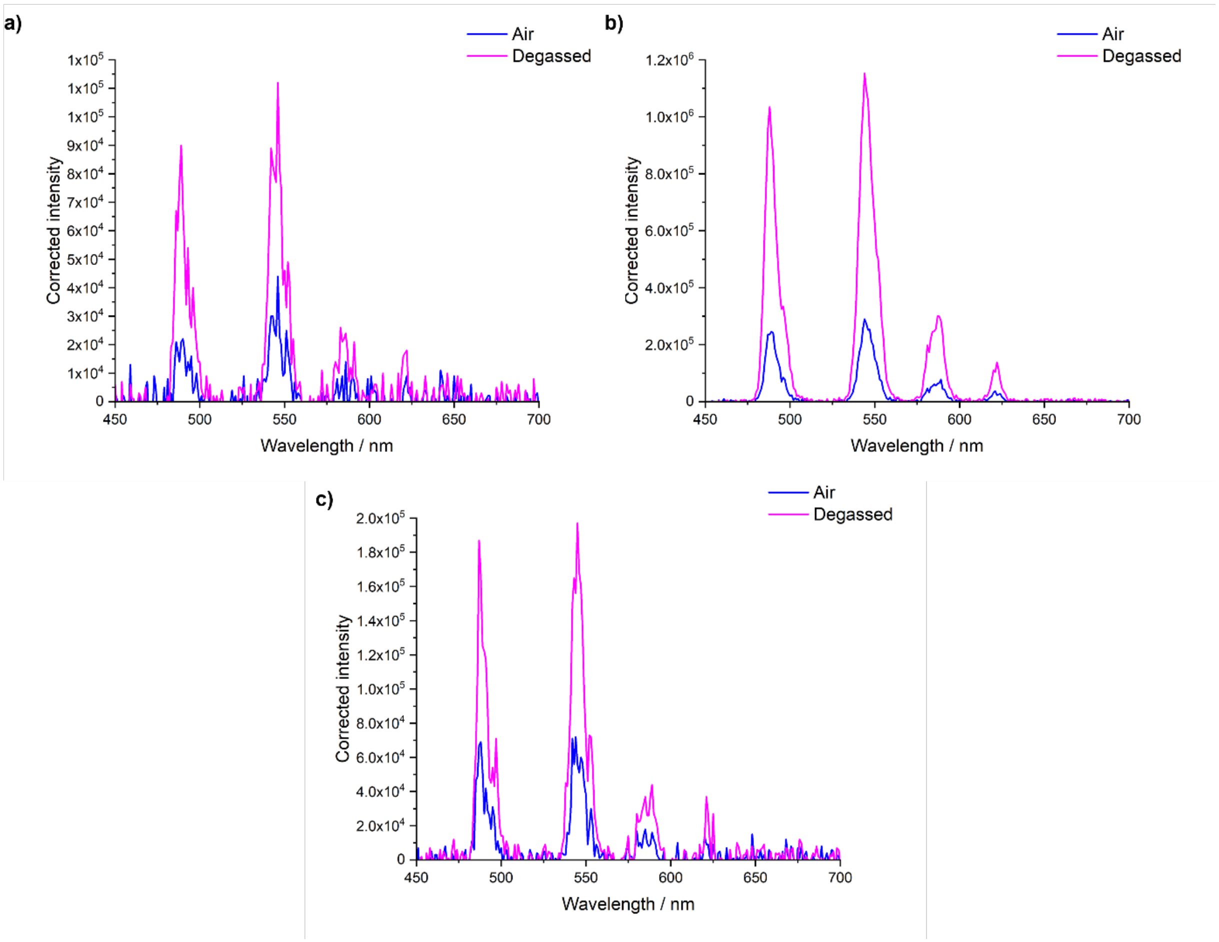

Figure S20: The time-gated emission spectra comparing TbNI (**2b**) measured in air (blue) and degassed (pink, purged under argon), measured at 50 μM (a and b) or 200 μM (c). Panel a: λ_{ex} 320 nm. Panels b and c: λ_{ex} 366 nm. 50 μM slits 29 nm / 3.5 nm; 200 μM slits 29 nm / 1.5 nm.

a)

	50 µM, λ_{ex} 320 nm		200 µM, λ_{ex} 366 nm	
	τ_{H2O} / ms		τ_{H2O} / ms	
**Air**	1.82	0.27	2.08	0.20
**Degassed**	1.95		1.96	

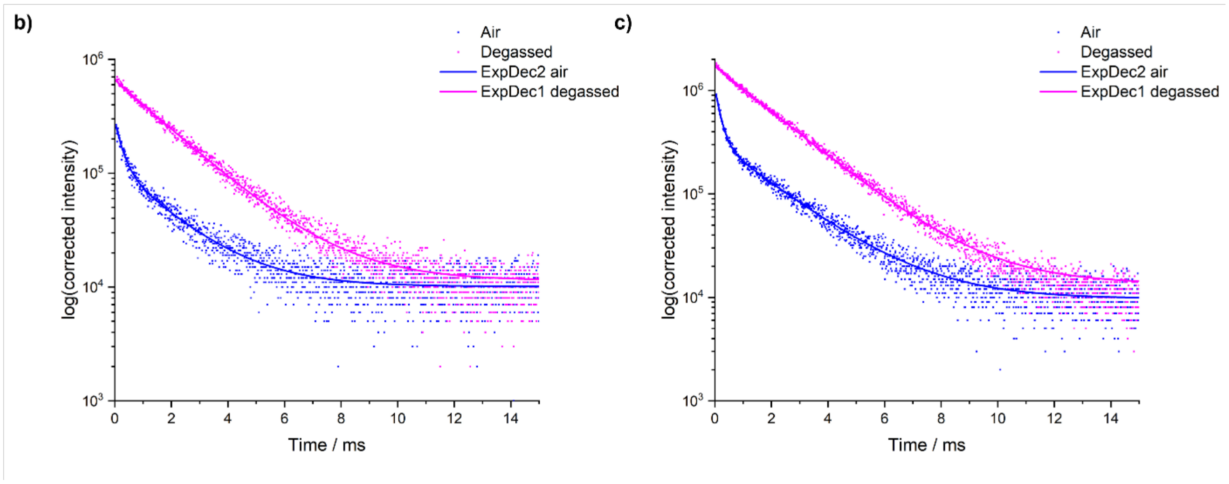

Figure S21: The lifetime measurements (panel a) and corresponding graphs (panel b, 50 µM, λ_{ex} 320 nm; panel c, 200 µM, λ_{ex} 366 nm) for the comparison of air (blue) and degassed (pink, purged under argon) measurements of TbNI (**2b**). For all measurements, λ_{em} 545 nm, sample window 1 ms, initial delay 0.05 ms, time per flash 61 ms, delay increment 0.01 ms, flash count 10, 400 nm long pass filter. Panel b: TbNI 50 µM in H_2O, λ_{ex} 320 nm, slits 29 nm / 12 nm. Panel c: TbNI 200 µM in H_2O, λ_{ex} 366 nm, slits 29 nm / 6 nm for air, 29 nm / 5 nm for degassed. ExpDec1 refers to the monoexponential decay fitting and ExpDec2 refers to the biexponential decay fitting.

Relaxivity measurements

Inner-sphere water relaxivity (r^{IS}) depends on magnetic field strength and temperature and can be described by the Bloch and Solomon-Bloembergen-Morgen equations (see equation S4 for a Bloch equation), assuming that there is fast exchange and bulk water is present at a higher concentration than metal bound water. These equations may be simplified to equation S5 for simple metal complexes, where C is a constant. *q* is the number of inner sphere water molecules, τ_r is the rate of molecular tumbling, τ_m is the rate of water exchange and T_{1m} (and T_{2m}) corresponds to a coordinated water molecule and is described by the Solomon-Bloembergen-Morgen equations.[11-13]

$$r^{IS} = \frac{q/[H_2O]}{T_{1m} + \tau_m} \qquad (S4)$$

$$r^{IS} = Cq\tau_r \qquad (S5)$$

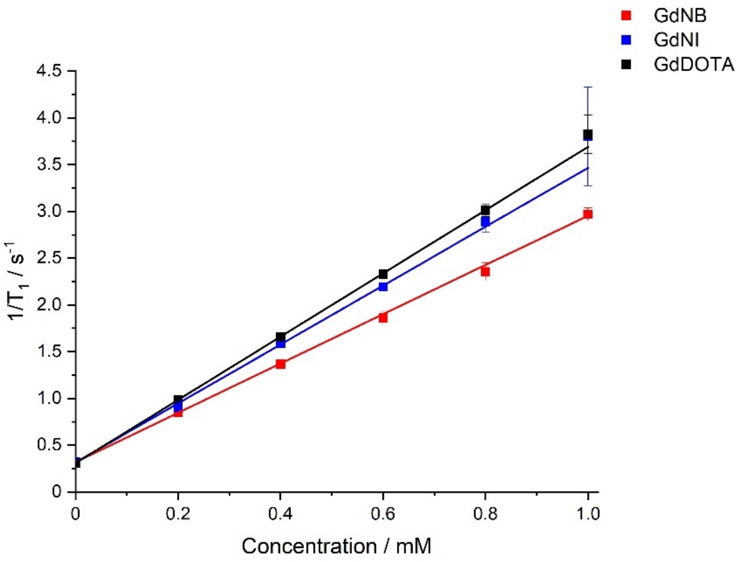

Figure S22: T_1 measurements of GdNB (**1c**, red) and GdNI (**2c**, blue) measured in comparison to GdDOTA (Dotarem®, black). Samples were measured from 0-1 mM in water in a sealed capillary inside an NMR tube on a 500 MHz NMR instrument (11.7 T) at 298 K. No lock solvent was required. Values are shown as the mean of repeats ± the standard deviation and error bars represent the standard deviation, n = 3.

Electrochemical measurements

Table S7: Cathodic onset potentials of 1 mM TbNB (**1b**) and TbNI (**2b**) dissolved in buffer, all measured at ambient temperature.

	**Cathodic onset potential vs SHE / V**	
	pH 7.4 (PBS)	pH 6.0 (MES)
TbNB **1b**	-0.356	-0.217
TbNI **2b**	-0.236	-0.119

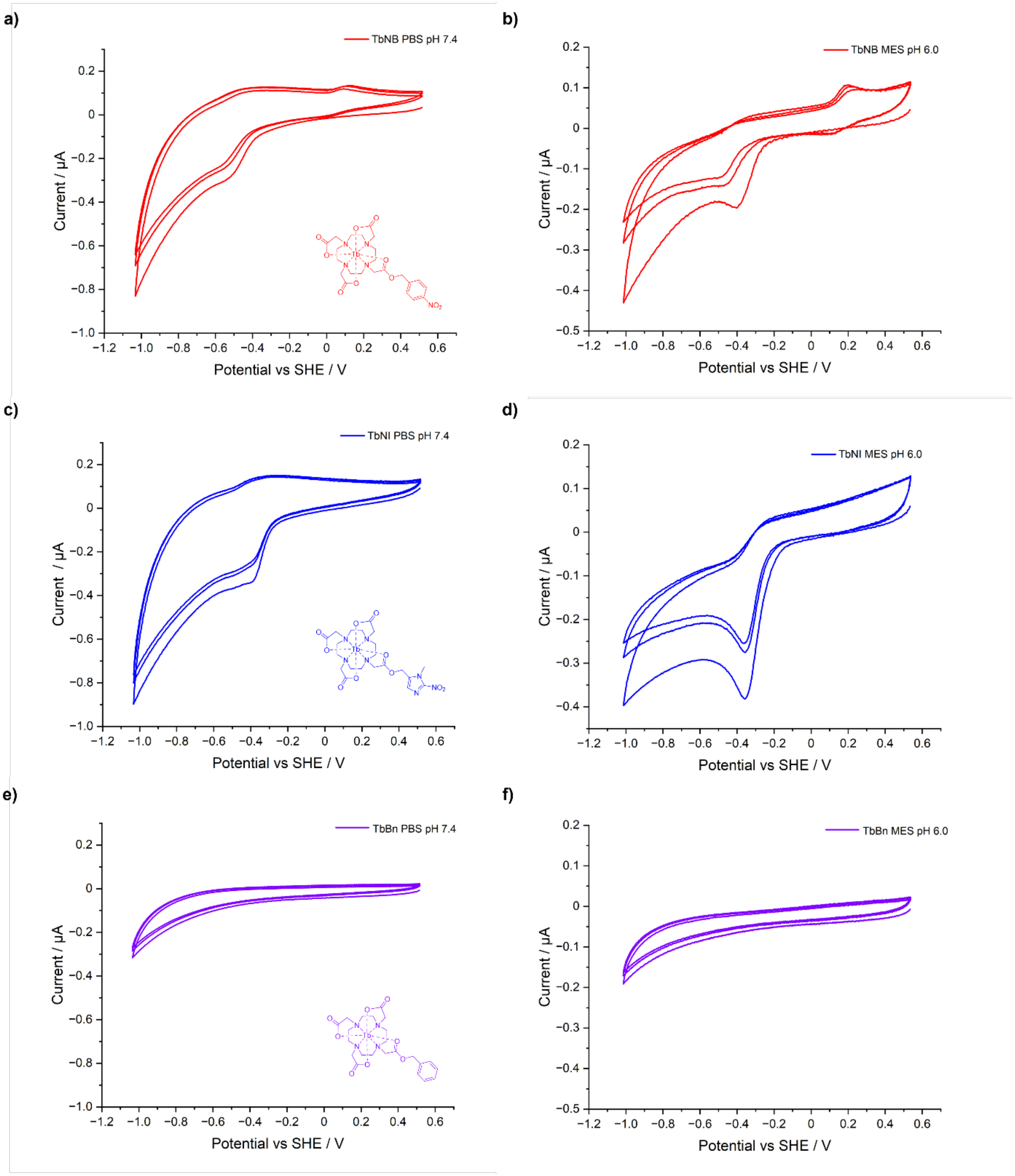

Figure S23: Cyclic voltammograms of a) TbNB (**1b**, red), c) TbNI (**2b**, blue) and e) TbBn (**4b**, purple) in PBS at pH 7.4, b) TbNB, d) TbNI and f) TbBn in MES at pH 6, showing 3 cycles.

Chemical reduction assay

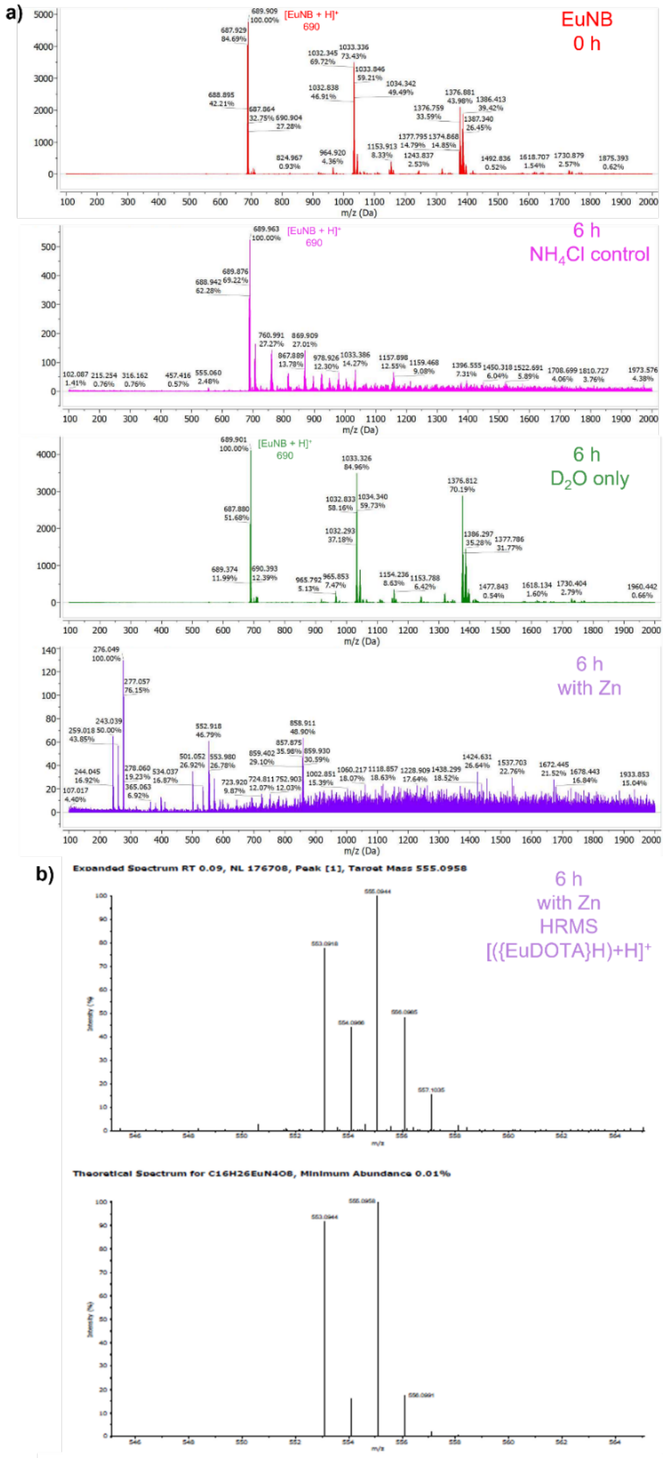

Measured Mass	Calculated Mass	Error (mDa)	Error (ppm)	Formula [M+H]$^+$	Response
555.0944	555.0958	-1.36	-2.46	$C_{16}H_{26}EuN_4O_8$	461298

Figure S24: a) The stacked LRMS spectra (ESI$^+$ ionisation) from the zinc assay of EuNB (**1a**), showing the controls (D_2O only, green, and NH_4Cl, pink, after 6 hours) compared to the initial t=0 measurement (red) and the end timepoint of the assay (t=6, light purple). b) The results of HRMS analysis (ESI$^+$ ionisation) of the t=6 timepoint, showing the presence of the [(EuDOTA+H)+H]$^+$ fragment. The chemical reduction assay protocol was adapted from O'Connor *et al.*[14]

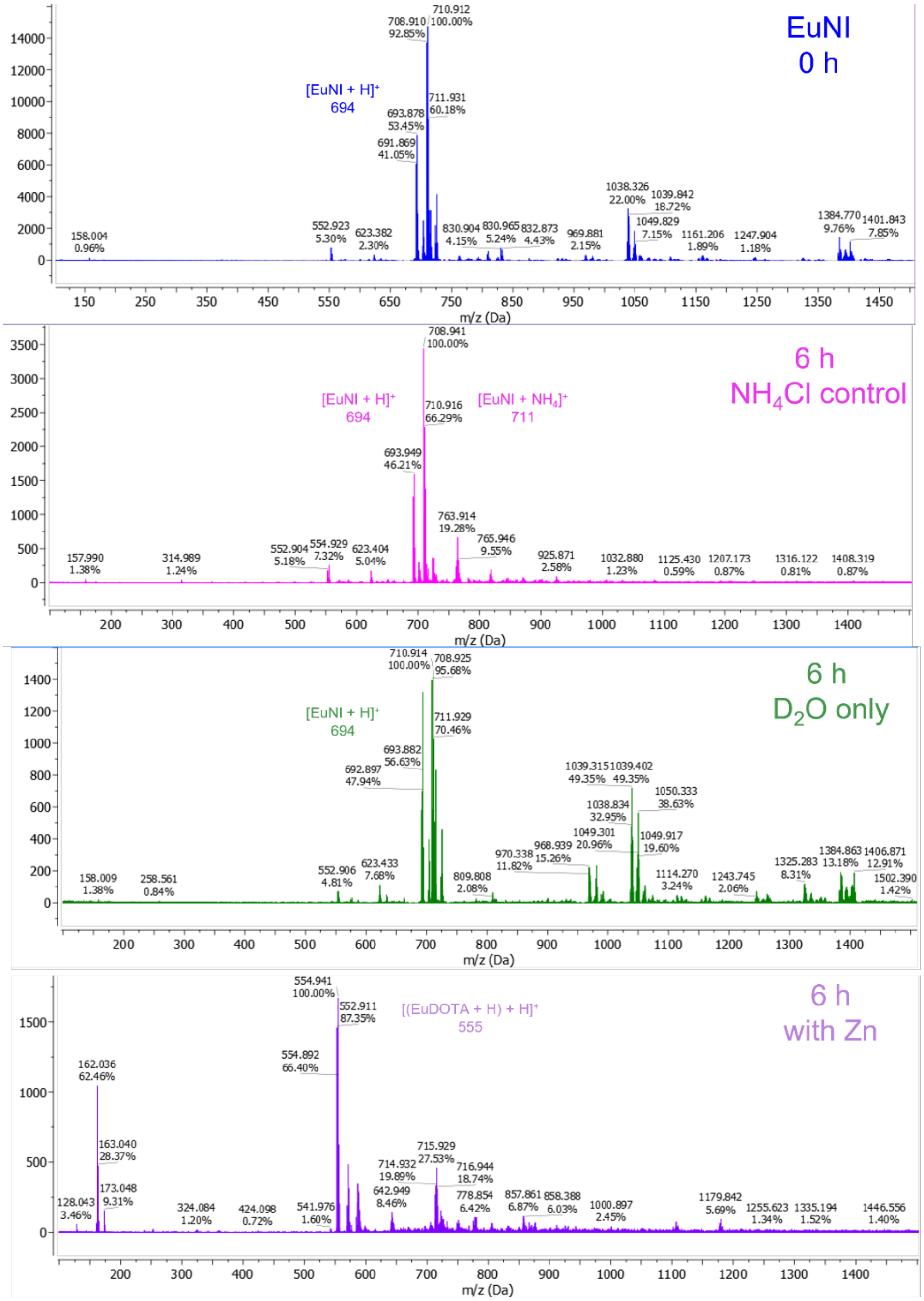

Figure S25: The stacked LRMS spectra (ESI$^+$ ionisation) from the zinc assay of EuNI (**2a**), showing the controls (D_2O only, green, and NH_4Cl, pink, after 6 hours) compared to the initial t=0 measurement (dark blue) and the end timepoint of the assay (t=6, light purple). The chemical reduction assay protocol was adapted from O'Connor *et al.*[14]

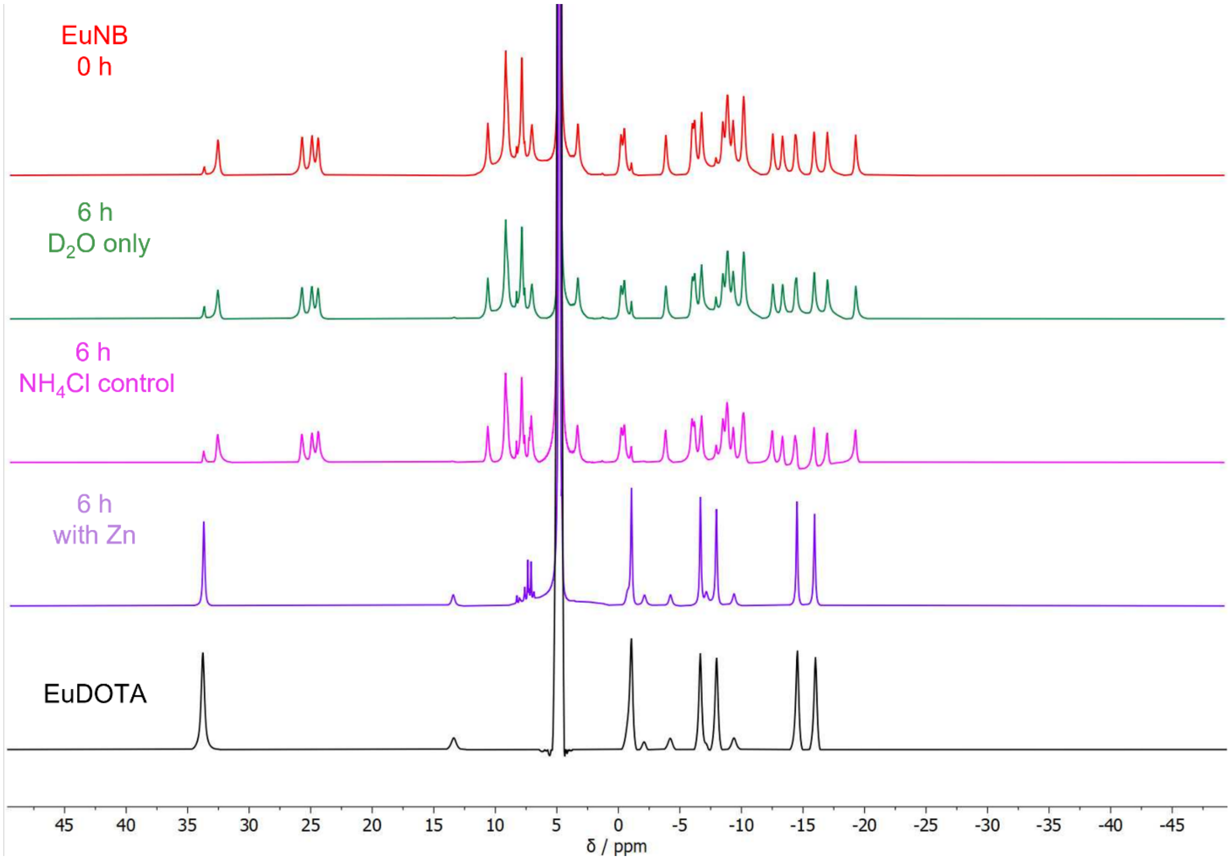

Figure S26: The stacked ^{1}H NMR spectra from the zinc assay of EuNB (**1a**), showing the controls (D_2O only, green, and NH_4Cl, pink, after 6 hours) compared to the initial t=0 measurement (red), EuDOTA (**3a**, the positive control, black) and the end timepoint of the assay (t=6, light purple). The chemical reduction assay protocol was adapted from O'Connor *et al.*[14] The EuDOTA control ^{1}H NMR was in D_2O only.

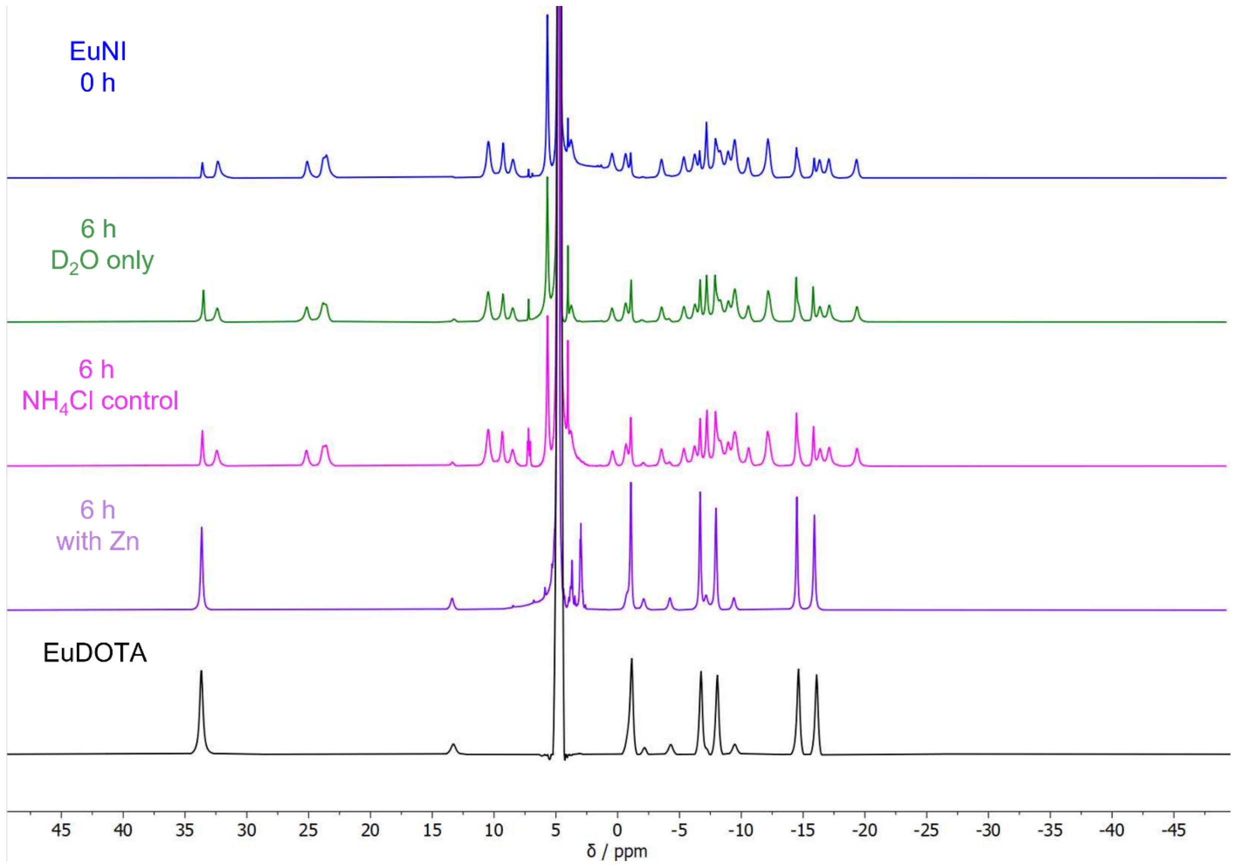

Figure S27: The stacked ^1H NMR spectra from the zinc assay of EuNI (**2a**), showing the controls (D_2O only, green, and NH_4Cl, pink, after 6 hours) compared to the initial t=0 measurement (dark blue), EuDOTA (**3a**, the positive control, black) and the end timepoint of the assay (t=6, light purple). The chemical reduction assay protocol was adapted from O'Connor *et al.*[14] The EuDOTA control ^1H NMR was in D_2O only.

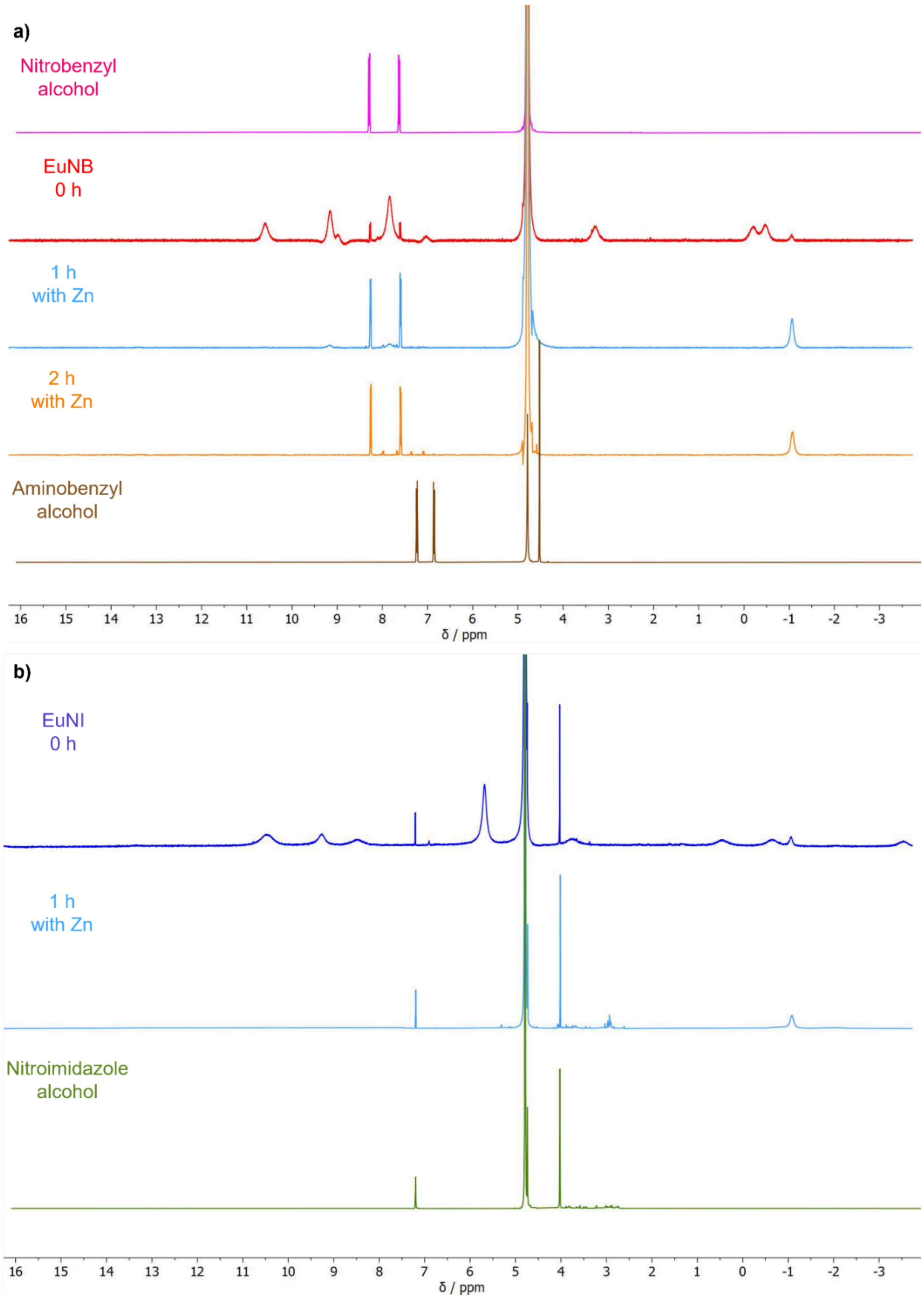

Figure S28: The stacked ^1H NMR spectra (-4 to 16 ppm range) from the zinc assays of a) EuNB (**1a**, red) and b) EuNI (**2a**, dark blue), showing the timepoints (1 h light blue, 2 h orange) up to full EuDOTA conversion (as determined by the ^1H NMR spectra for europium complexes, Figure 4), in comparison to the initial t=0 measurement. For comparison, the ^1H NMR spectra of nitrobenzyl alcohol (hot pink) and aminobenzyl alcohol (brown) were analysed by diluting solid in 540 µL D_2O and 60 µL NH_4Cl solution (10wt% in D_2O) and filtering (Nylon syringe filter) in an NMR tube. The ^1H NMR spectra of nitroimidazole alcohol was measured in D_2O only.

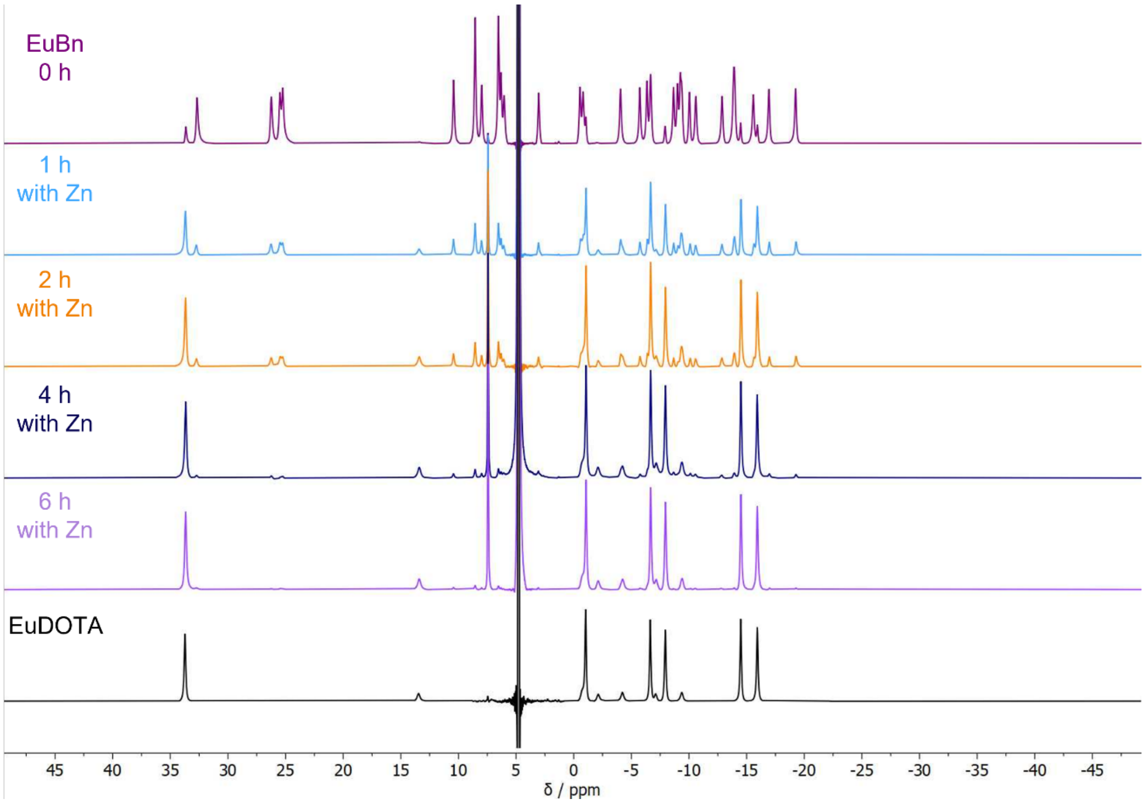

Figure S29: The stacked ^{1}H NMR spectra of the timepoints (1 h light blue, 2 h orange, 4 h dark blue, 6 h light purple) from the zinc assay of EuBn (**4a**, dark purple, 0 h 6% EuDOTA) in D_2O, in comparison to the positive control EuDOTA (**3a**, black).

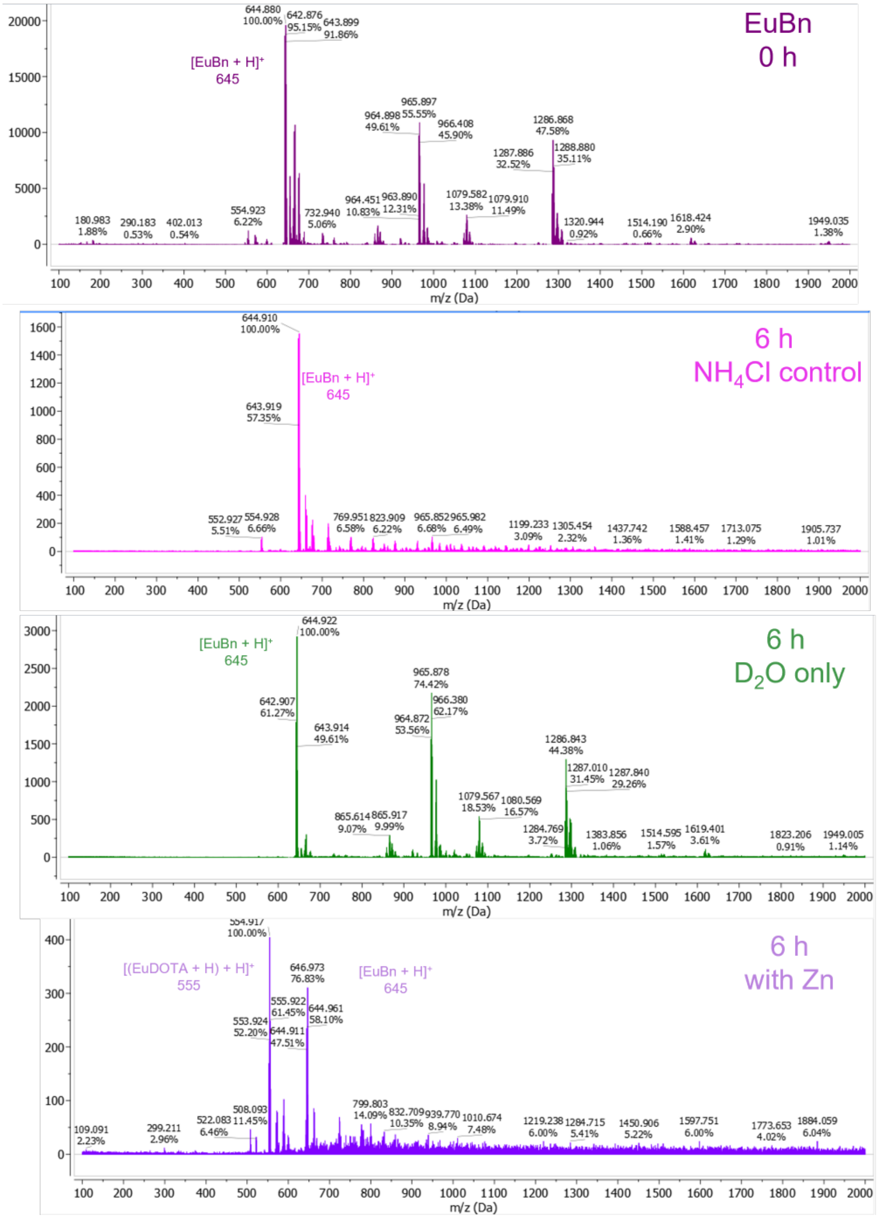

Figure S30: The stacked LRMS spectra (ESI$^+$ ionisation) from the zinc assay of EuBn (**4a**), showing the controls (D_2O only, green, and NH_4Cl, pink, after 6 hours) compared to the initial t=0 measurement (dark purple) and the end timepoint of the assay (t=6, light purple).

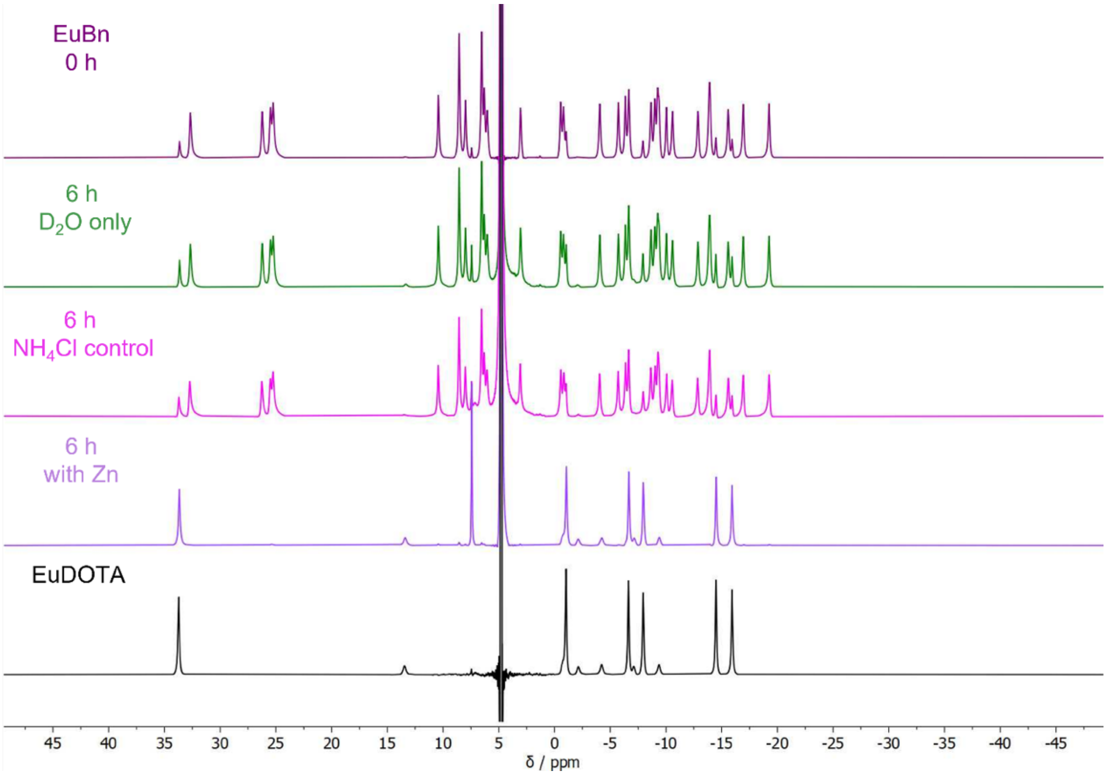

Figure S31: The stacked ^{1}H NMR spectra from the zinc assay of EuBn (**4a**), showing the controls (D_2O only, green, and NH_4Cl, pink, after 6 hours) compared to the initial t=0 measurement (dark purple), EuDOTA (**3a**, the positive control, black) and the end timepoint of the assay (t=6, light purple).

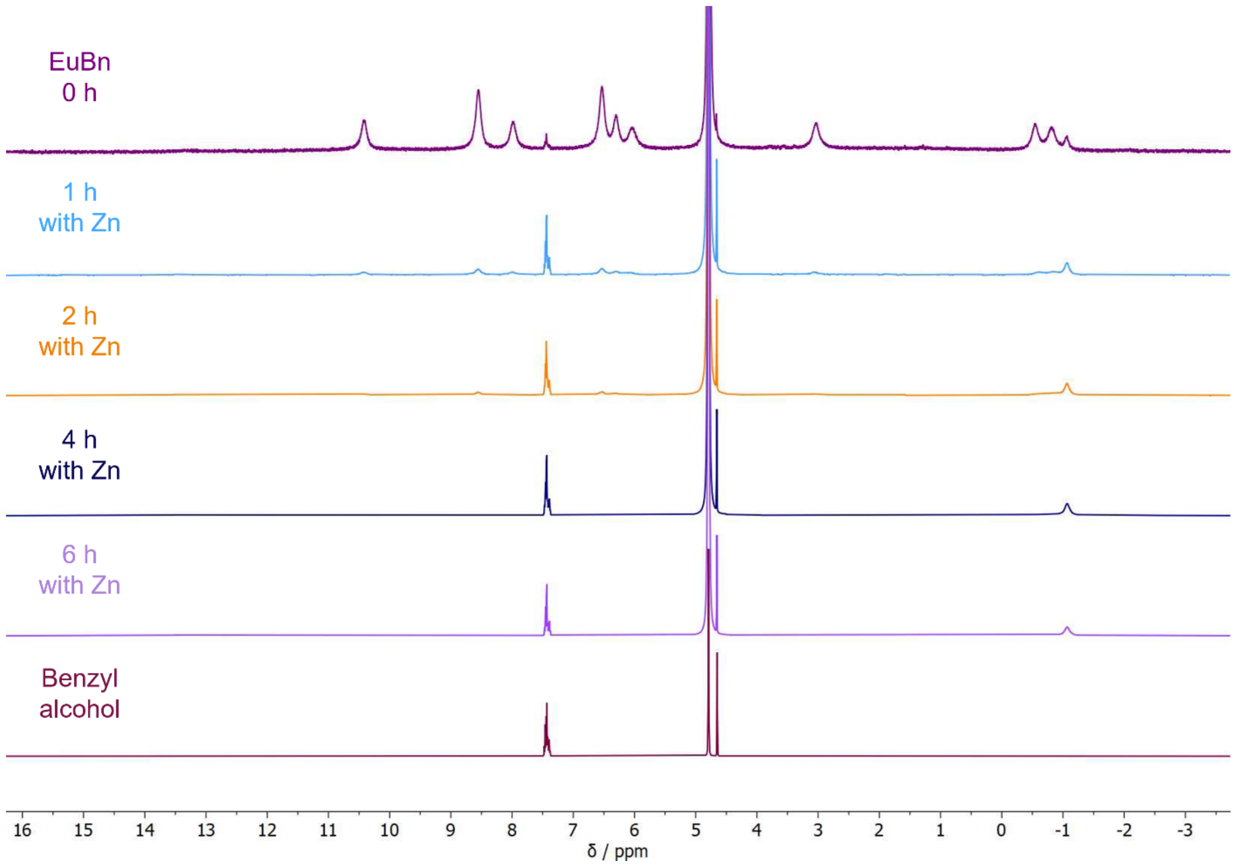

Figure S32: The stacked ^{1}H NMR spectra (-4 to 16 ppm range) from the zinc assay of EuBn (**4a**, dark purple), showing the timepoints (1 h light blue, 2 h orange, 4 h dark blue, 6 h light purple), in comparison to the initial t=0 measurement. For comparison, ^{1}H NMR spectra of benzyl alcohol (dark red) was analysed by diluting solid in 540 µL D_2O and 60 µL NH_4Cl solution (10wt% in D_2O) and filtering (Nylon syringe filter) in an NMR tube.

Stability data

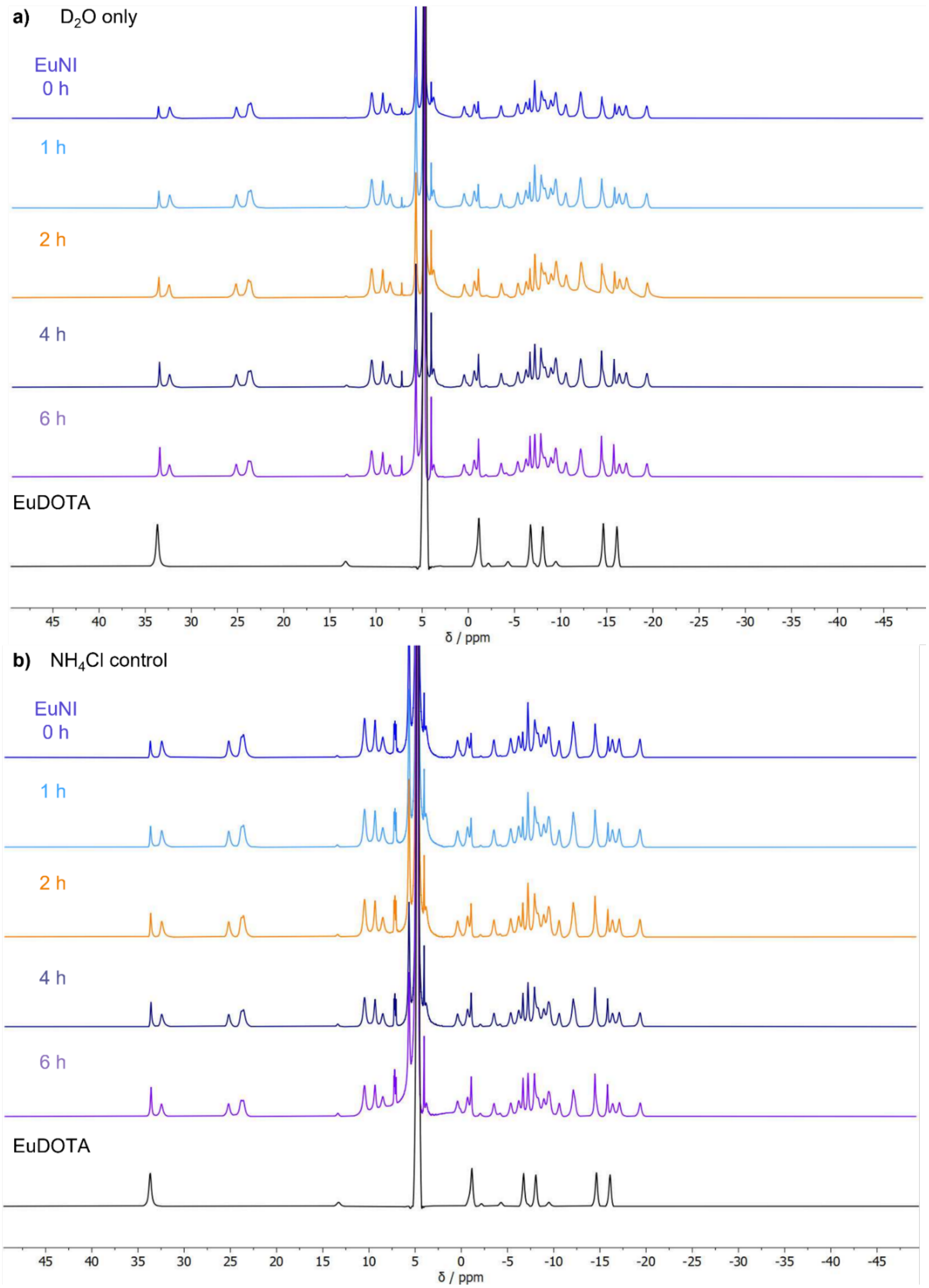

Figure S33: The stacked ^1H NMR spectra from the zinc assay control experiment, showing the timepoints compared to EuNI (**2a**, dark blue, t=0 10% EuDOTA) and EuDOTA (**3a**, black). a) D_2O only, b) NH_4Cl and D_2O. In both assays, 22% EuDOTA was observed after 6 h (by integration of the peak at 34 ppm, 4H, belonging to EuDOTA, compared to the peak at 33 ppm, 1H, belonging to EuNI). The EuDOTA control ^1H NMR was in D_2O only.

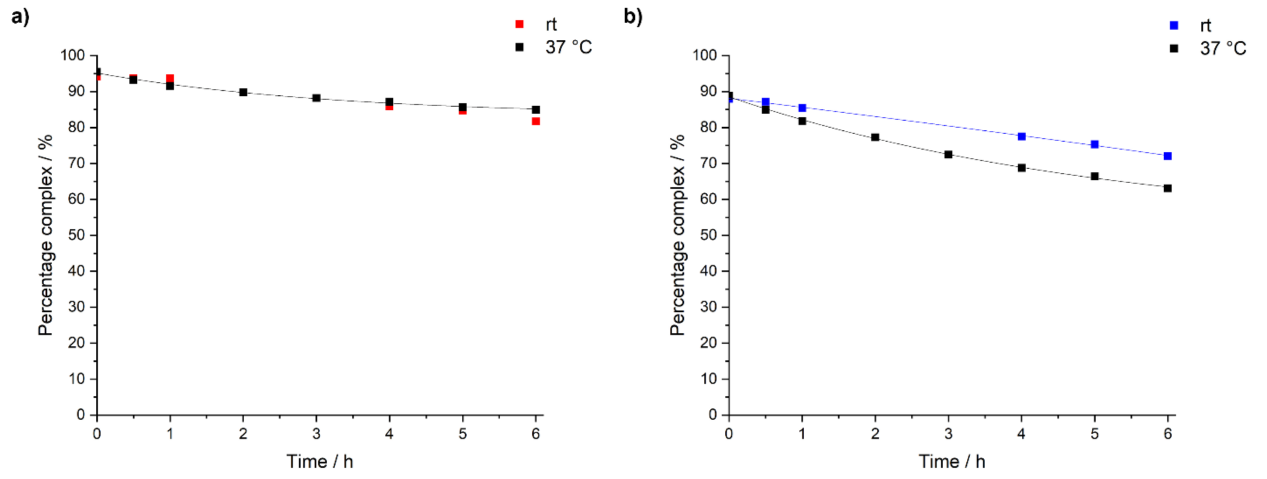

Figure S34: Stability data for a) TbNB (**1b**) and b) TbNI (**2b**) in water over time, analysed by percentage complex by LCMS (method 3) total UV absorbance, see the general experimental for more information. The samples were analysed by percentage purity of the complex at the indicated timepoints (area of the peak corresponding to the complex compared to total area of UV active components, as a percentage). TbDOTA was not observed by LCMS.

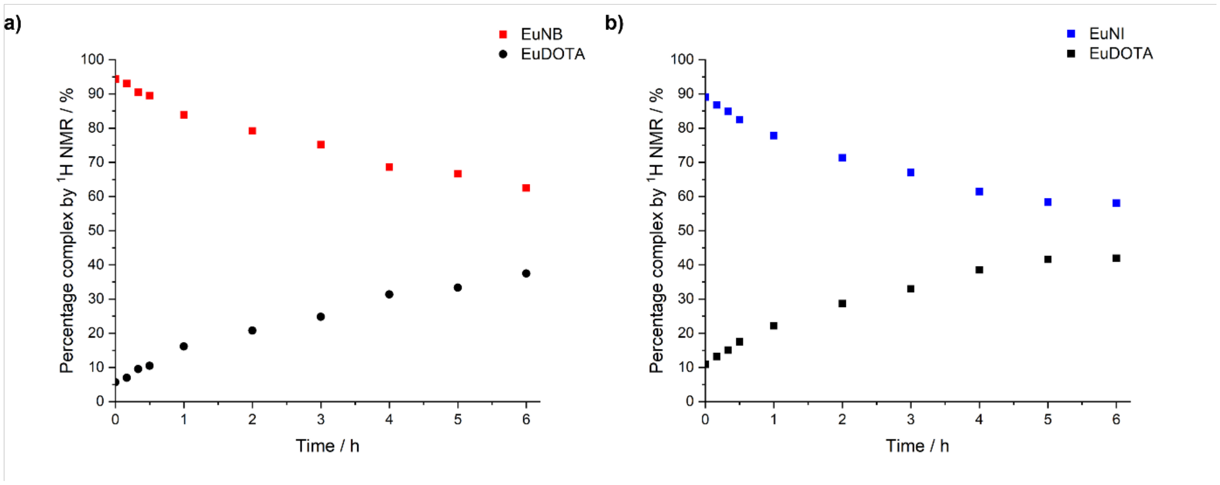

Figure S35: Quantitative analysis of the stability data for a) EuNB (**1a**) and b) EuNI (**2a**) in PBS over time, by ^{1}H NMR spectroscopy. The percentage of complex was determined by comparing the integral of the peak at 34 ppm (4H) belonging to EuDOTA (**3a**) to the integral of the peak at 33 ppm (1H) belonging to EuNB/EuNI. 5-7 mg of EuNB/EuNI was dissolved in 700 µL PBS (pH 7.4) and ^{1}H NMR spectra for europium complexes were recorded at the indicated timepoints. For comparison, 6.3 mg EuDOTA was dissolved in 700 µL PBS buffer and the ^{1}H NMR spectrum was recorded (black).

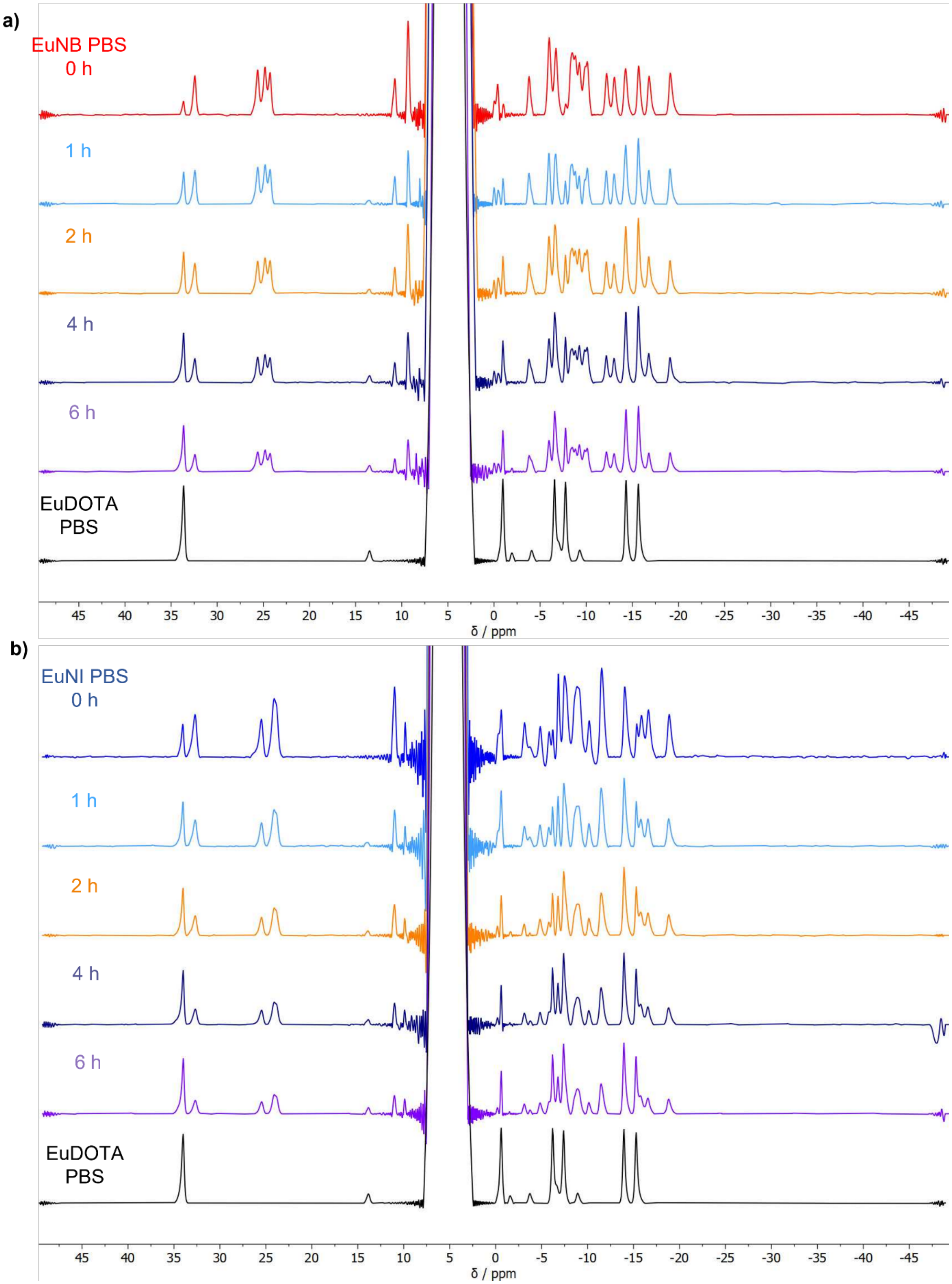

Figure S36: Stacked ^{1}H NMR spectra from the stability analysis of a) EuNB (**1a**) and b) EuNI (**2a**) in PBS over time. 5-7 mg of EuNB/EuNI was dissolved in 700 µL PBS (pH 7.4) and ^{1}H NMR spectra for europium complexes were recorded at the indicated timepoints. For comparison, 6.3 mg EuDOTA (**3a**) was dissolved in 700 µL PBS buffer and the ^{1}H NMR spectrum was recorded (black).

Nitroreductase enzyme assays

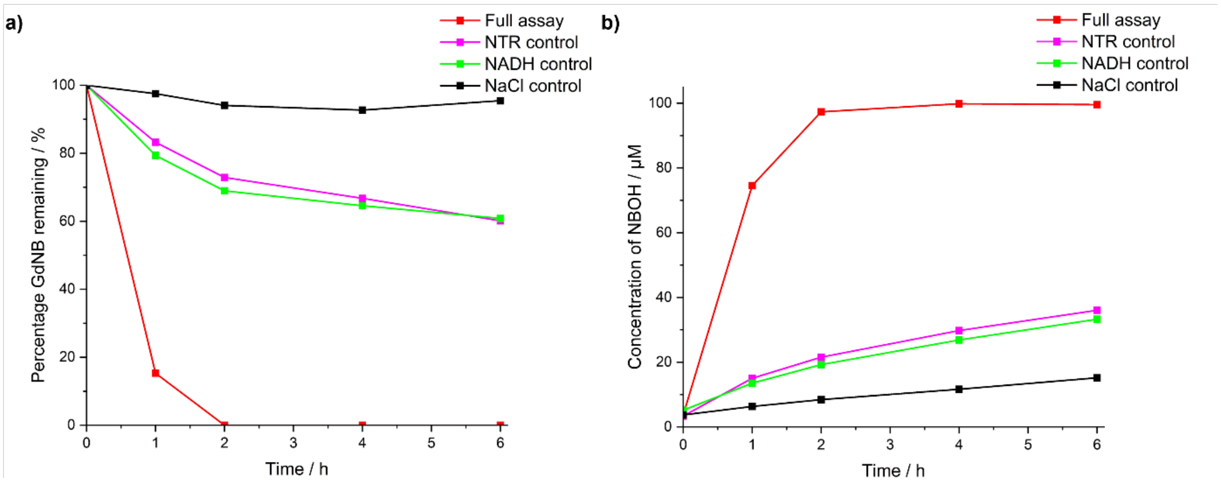

Figure S37: Quantitative analysis of the analytical HPLC data (method C, analysed at 269 nm, 50 µL injections) from the nitroreductase assay of GdNB (**1c**). a) The percentage of GdNB remaining, by analysis of the area of the peak at 8.0 min compared to the corresponding area at t=0. b) The concentration of nitrobenzyl alcohol (NBOH) released, by analysis of the area of the peak at 9.7 min compared to the corresponding area in the nitrobenzyl alcohol control.

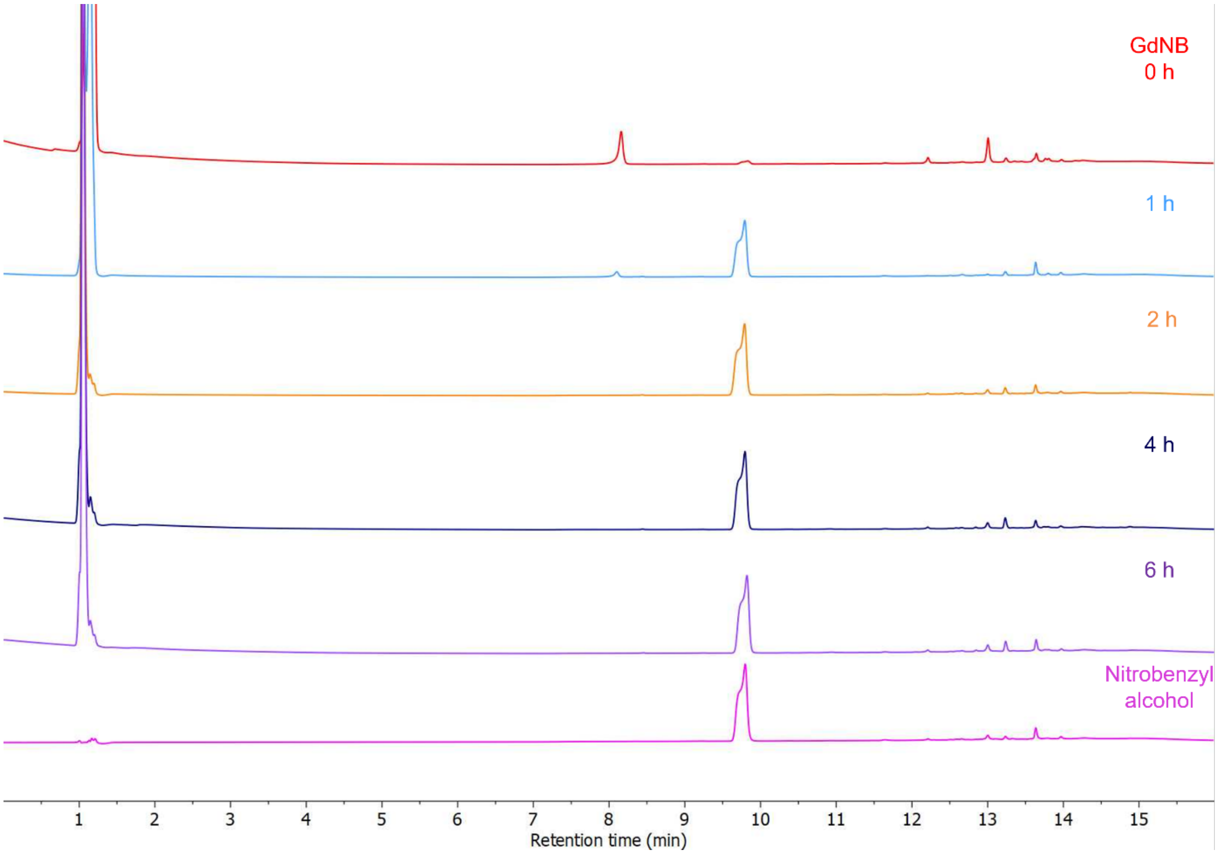

Figure S38: Stacks of the timepoints from the full nitroreductase assay of GdNB, analysed at 269 nm by analytical HPLC (method C, 50 µL injection), in comparison to the nitrobenzyl alcohol control.

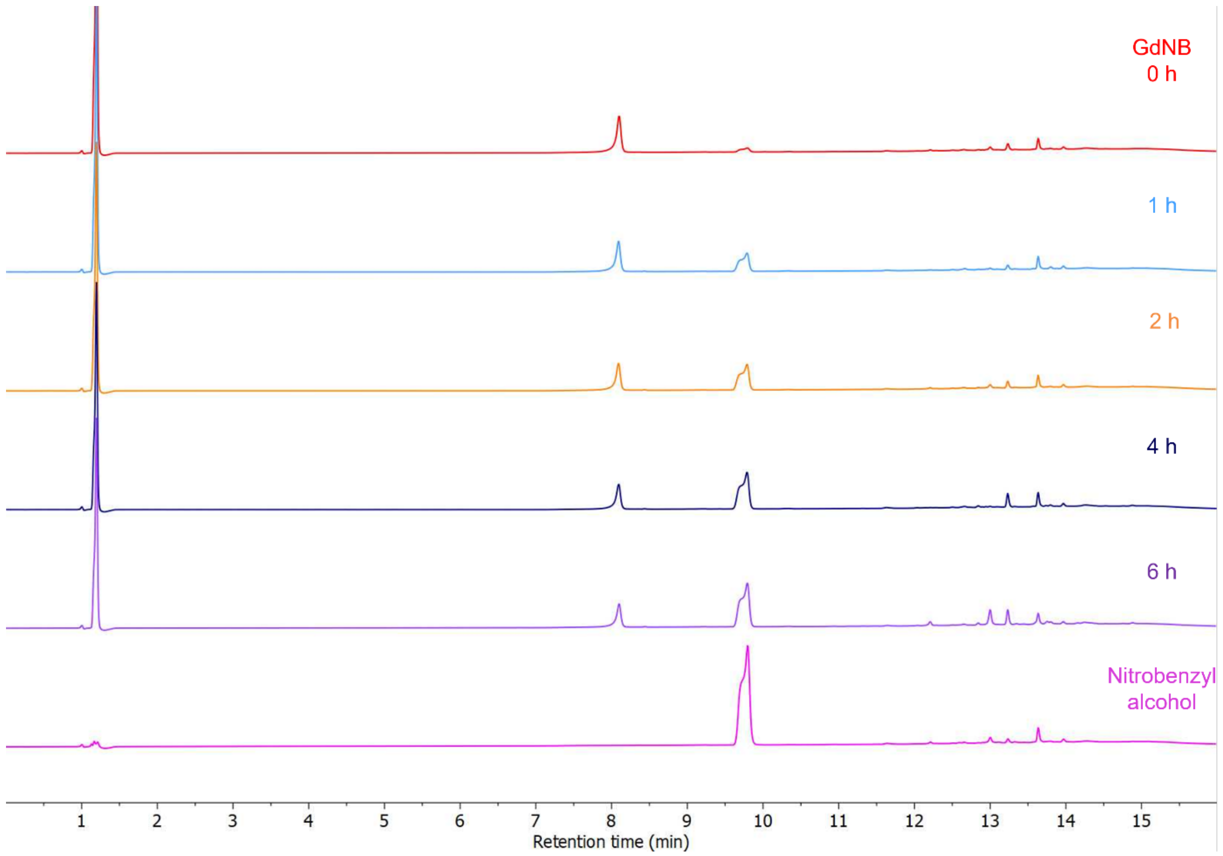

Figure S39: Stacks of the timepoints from the nitroreductase only control from the nitroreductase assay of GdNB, analysed at 269 nm by analytical HPLC (method C, 50 µL injection), in comparison to the nitrobenzyl alcohol control.

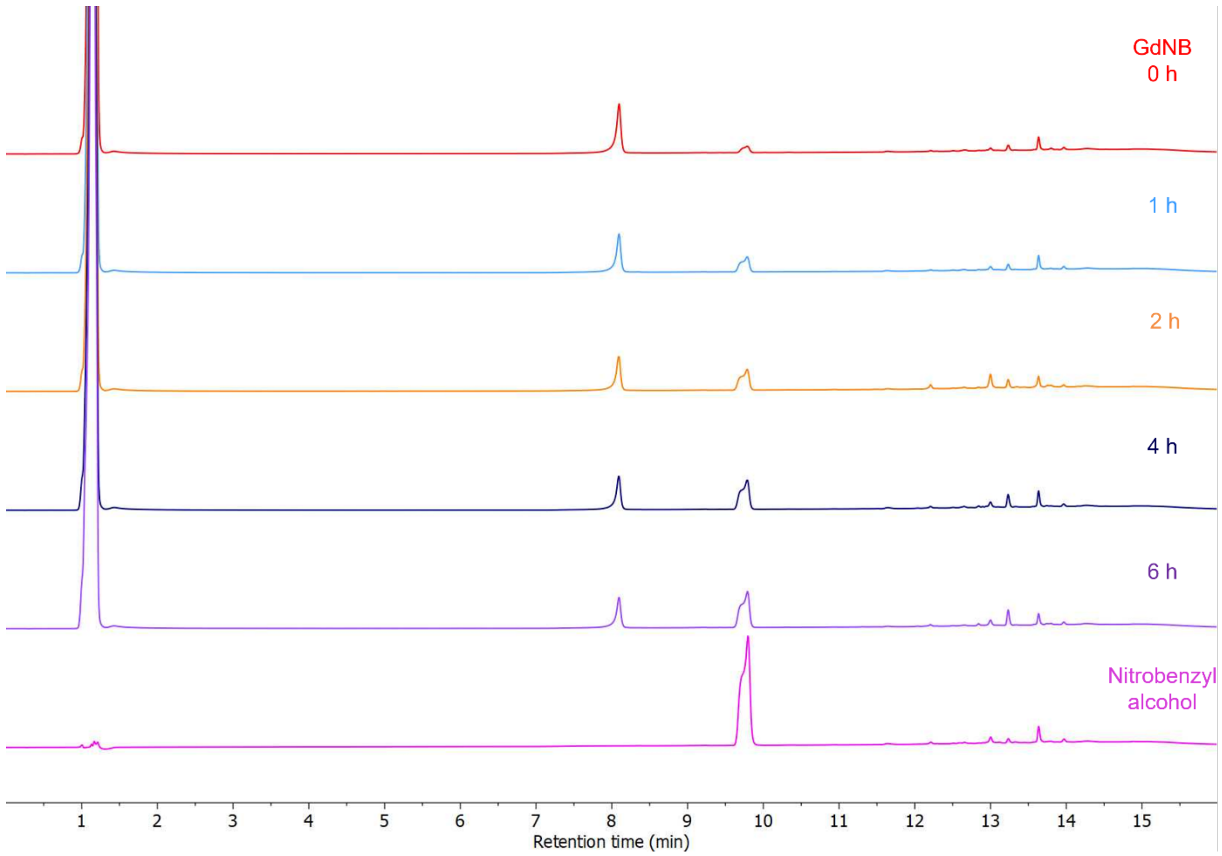

Figure S40: Stacks of the timepoints from the NADH only control from the nitroreductase assay of GdNB, analysed at 269 nm by analytical HPLC (method C, 50 µL injection), in comparison to the nitrobenzyl alcohol control.

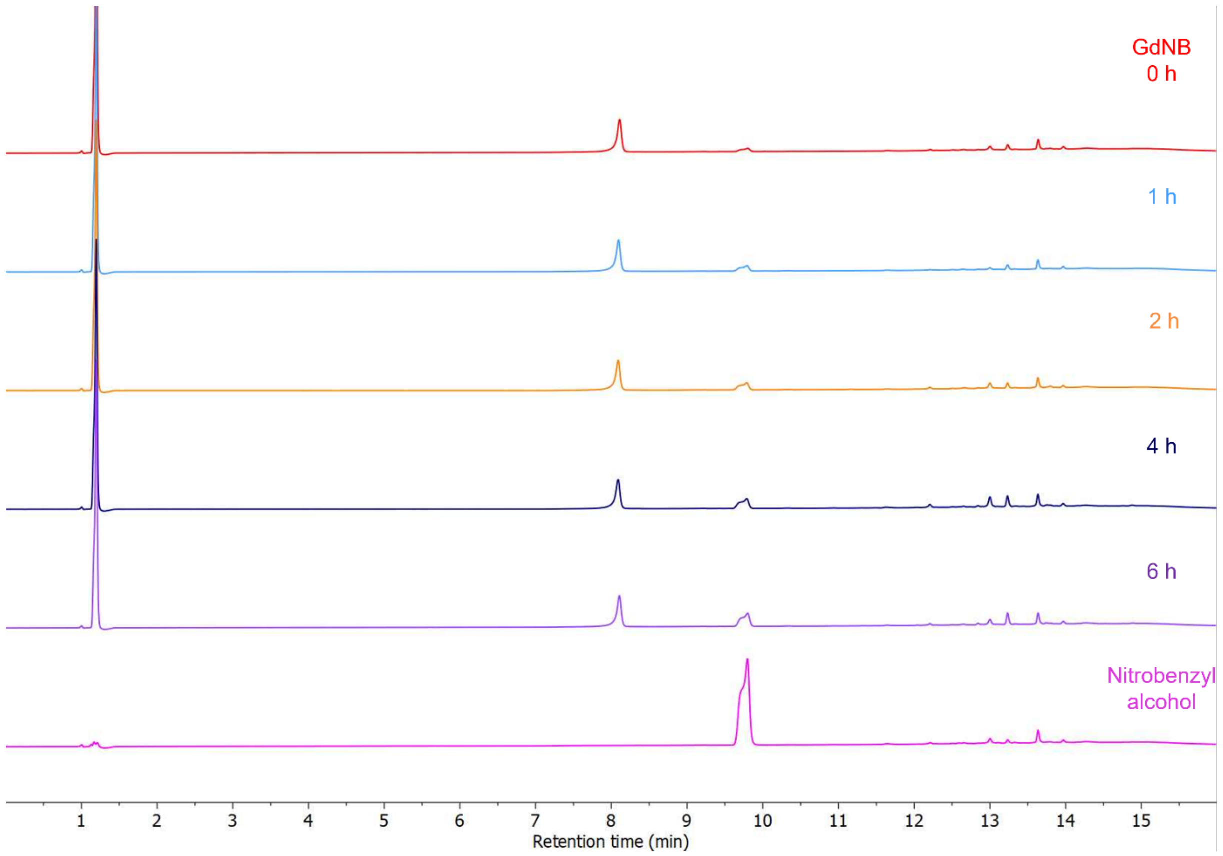

Figure S41: Stacks of the timepoints from the NaCl only control from the nitroreductase assay of GdNB, analysed at 269 nm by analytical HPLC (method C, 50 µL injection), in comparison to the nitrobenzyl alcohol control.

NADH NMR study

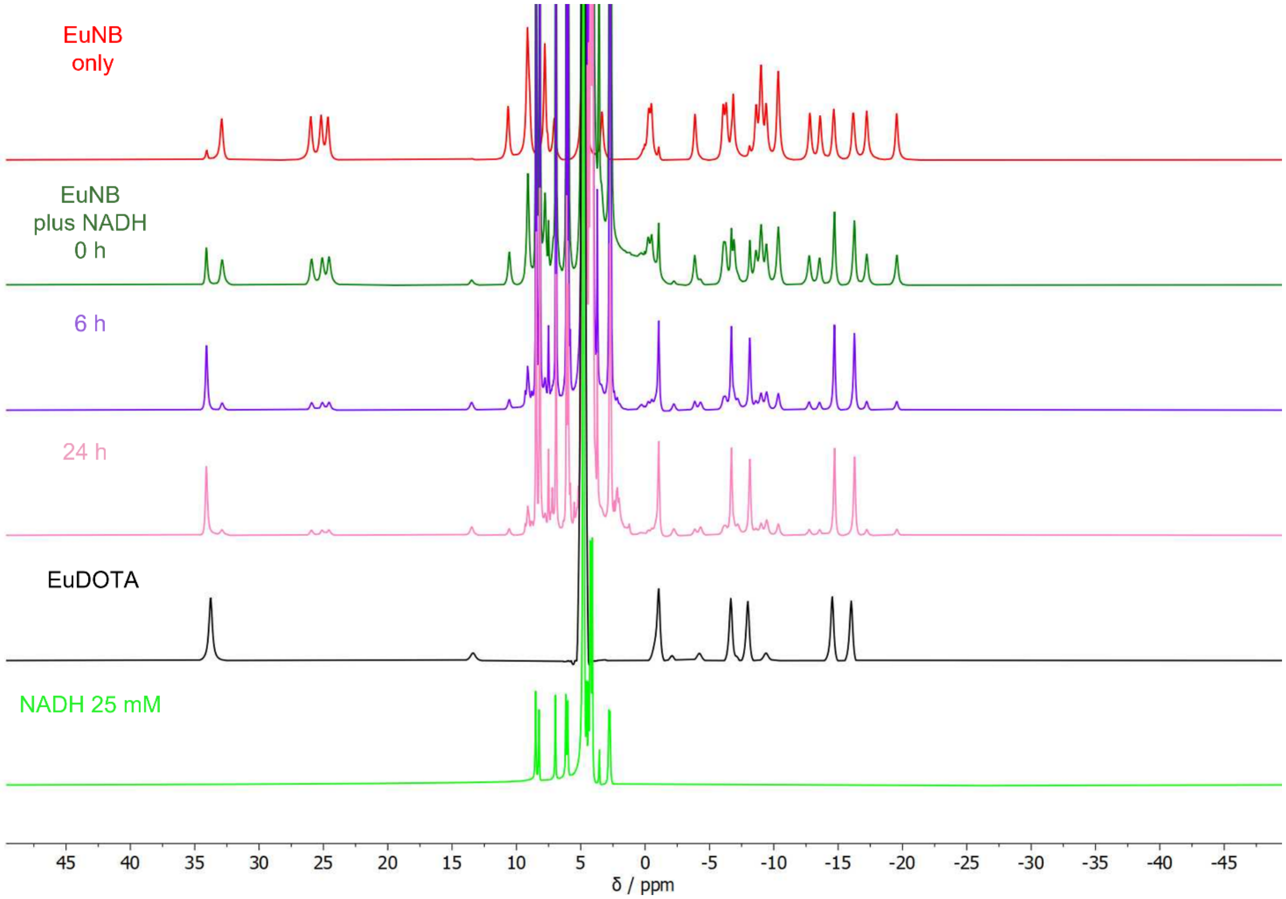

Figure S42: The stacked ^{1}H NMR spectra from the NADH assay of EuNB (**1a**, 10 mM), showing the timepoints (6 h, purple, and 24 h, pink) compared to the initial EuNB measurement (red), t=0 measurement (immediately after addition of NADH, dark green), EuDOTA (**3a**, the positive control, black) and NADH (25 mM, light green).

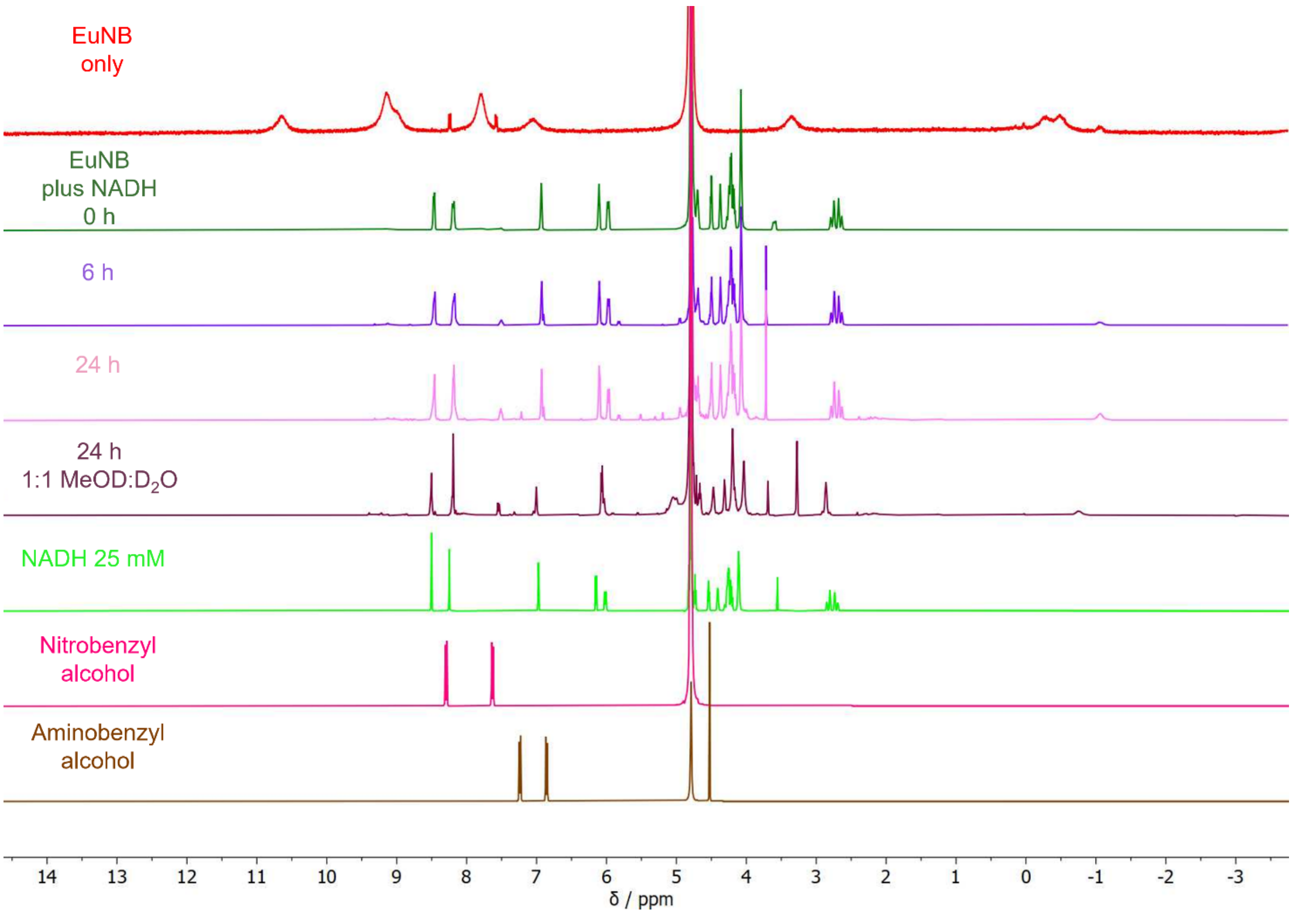

Figure S43: The stacked ^1H NMR spectra (-4 to 16 ppm range) from the NADH assay of EuNB (**1a**, 10 mM), showing the timepoints (6 h, purple, 24 h D_2O only, pink, and 24 h MeOD:D_2O 1:1, dark purple) compared to the initial EuNB measurement (red), t=0 measurement (immediately after addition of NADH, dark green), NADH (25 mM, light green), nitrobenzyl alcohol (hot pink) and aminobenzyl alcohol (brown). The nitrobenzyl alcohol and aminobenzyl alcohol control ^1H NMR spectra were measured in D_2O with 10% NH_4Cl (as in the zinc reduction assay).

Hydrogenase enzyme assays

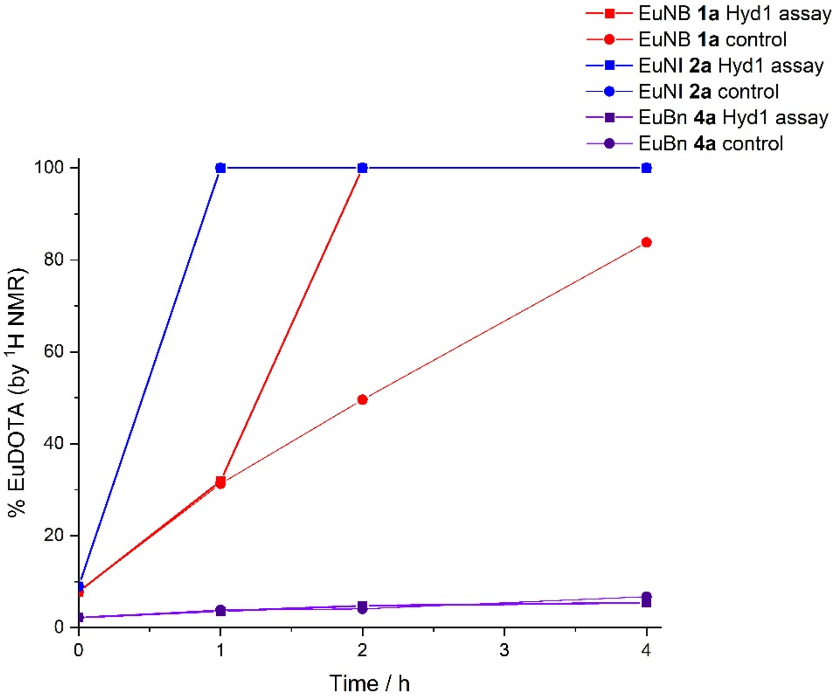

Figure S44: Quantitative analysis of the percentage EuDOTA released, compared to the starting europium complex, in the presence of the Hyd1 enzyme (squares) and in the control (without Hyd1 enzyme, circles), for EuNB (1a, red), EuNI (**2a**, blue) and EuBn (**3a**, purple).

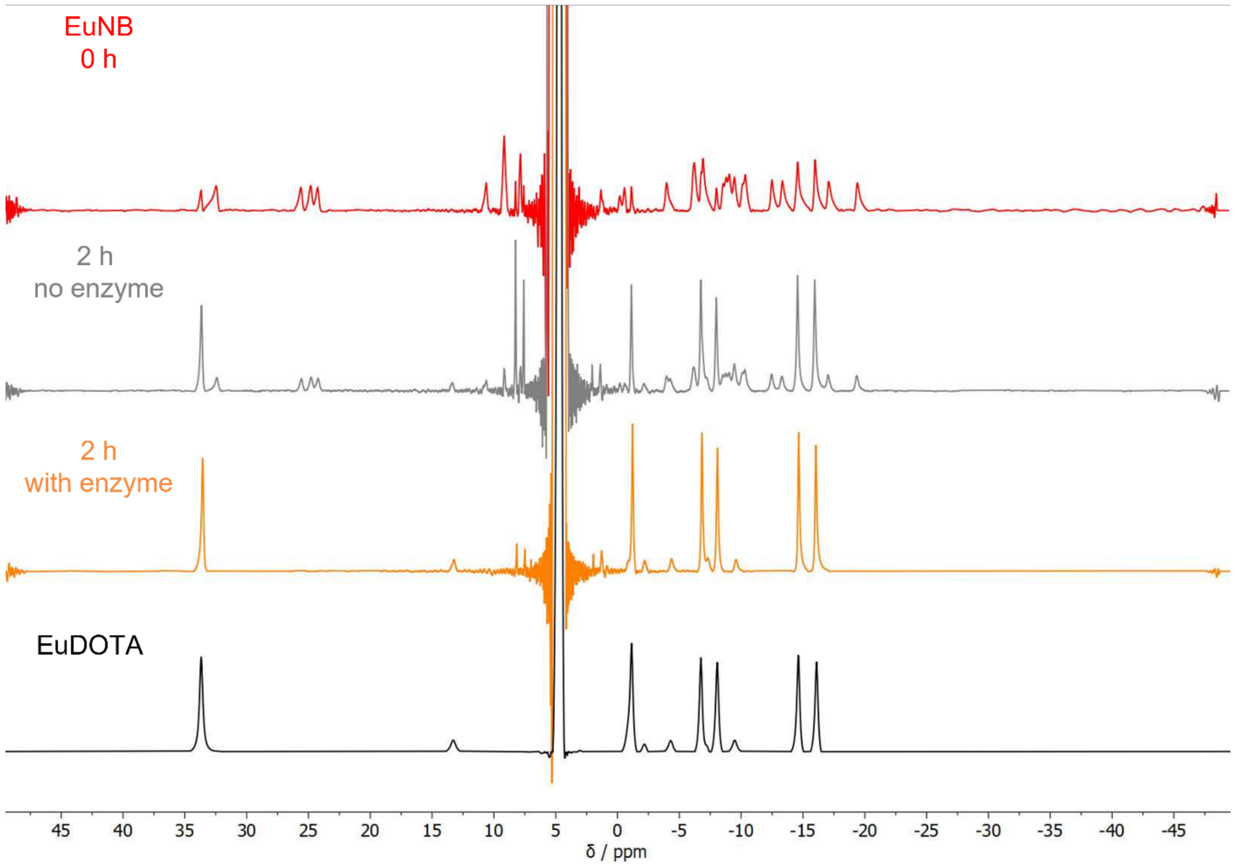

Figure S45: The stacked ^1H NMR spectra from the Hyd1 enzyme assay of EuNB (**1a**), showing the 2-hour timepoints (with enzyme, orange, and the control, grey) compared to the initial t=0 measurement (red) and EuDOTA (**3a**, the positive control, black). The enzymatic assay protocol was adapted from Sokolova *et al.*[15] The EuDOTA control ^1H NMR was in D_2O only.

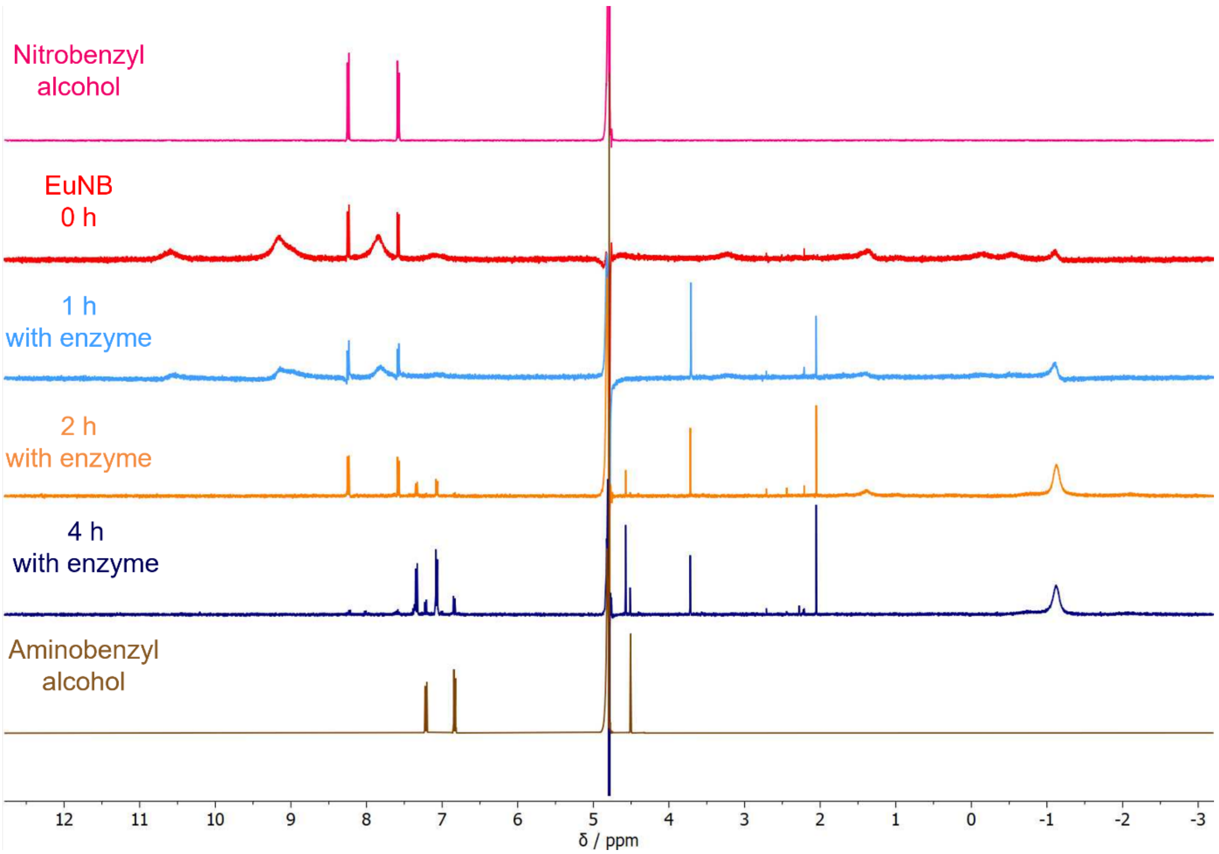

Figure S46: The stacked ^{1}H NMR spectra (with water suppression, -4 to 16 ppm range) from the Hyd1 enzyme assay of EuNB (**1a**), showing the timepoints compared to the initial t=0 measurement (red) and nitrobenzyl alcohol (hot pink) and aminobenzyl alcohol (brown) as controls. The enzymatic assay protocol was adapted from Sokolova *et al.*[15]

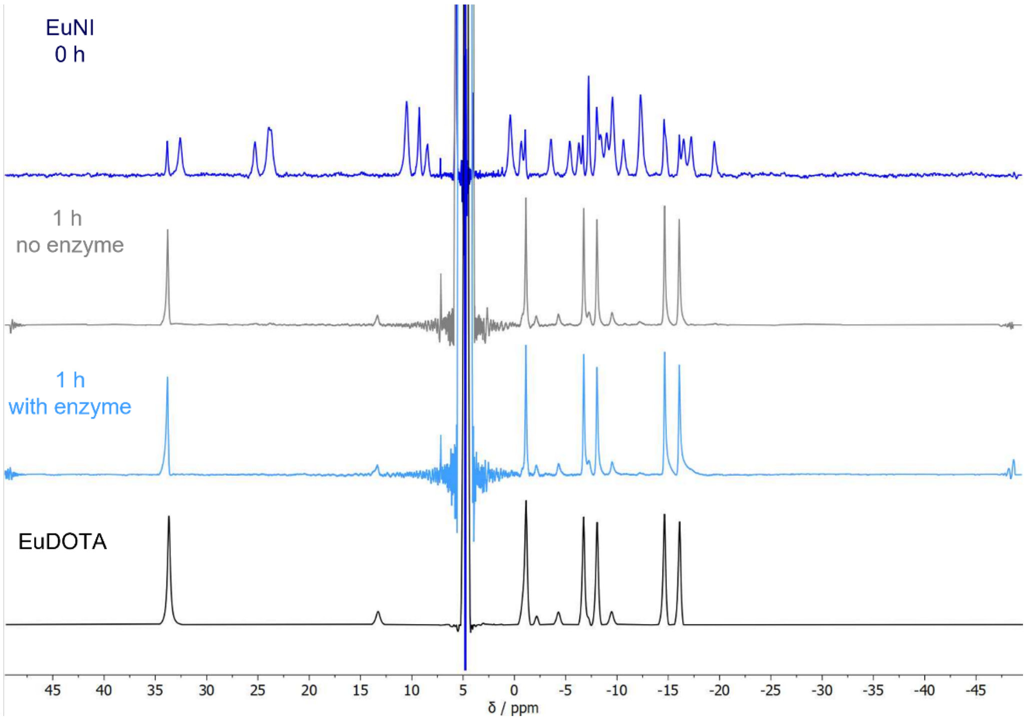

Figure S47: The stacked ^1H NMR spectra from the Hyd1 enzyme assay of EuNI (**2a**), showing the 1-hour timepoints (with enzyme, light blue, and the control, grey) compared to the initial t=0 measurement (dark blue) and EuDOTA (**3a**, the positive control, black). The enzymatic assay protocol was adapted from Sokolova *et al.*[15] The EuDOTA control ^1H NMR was in D_2O only.

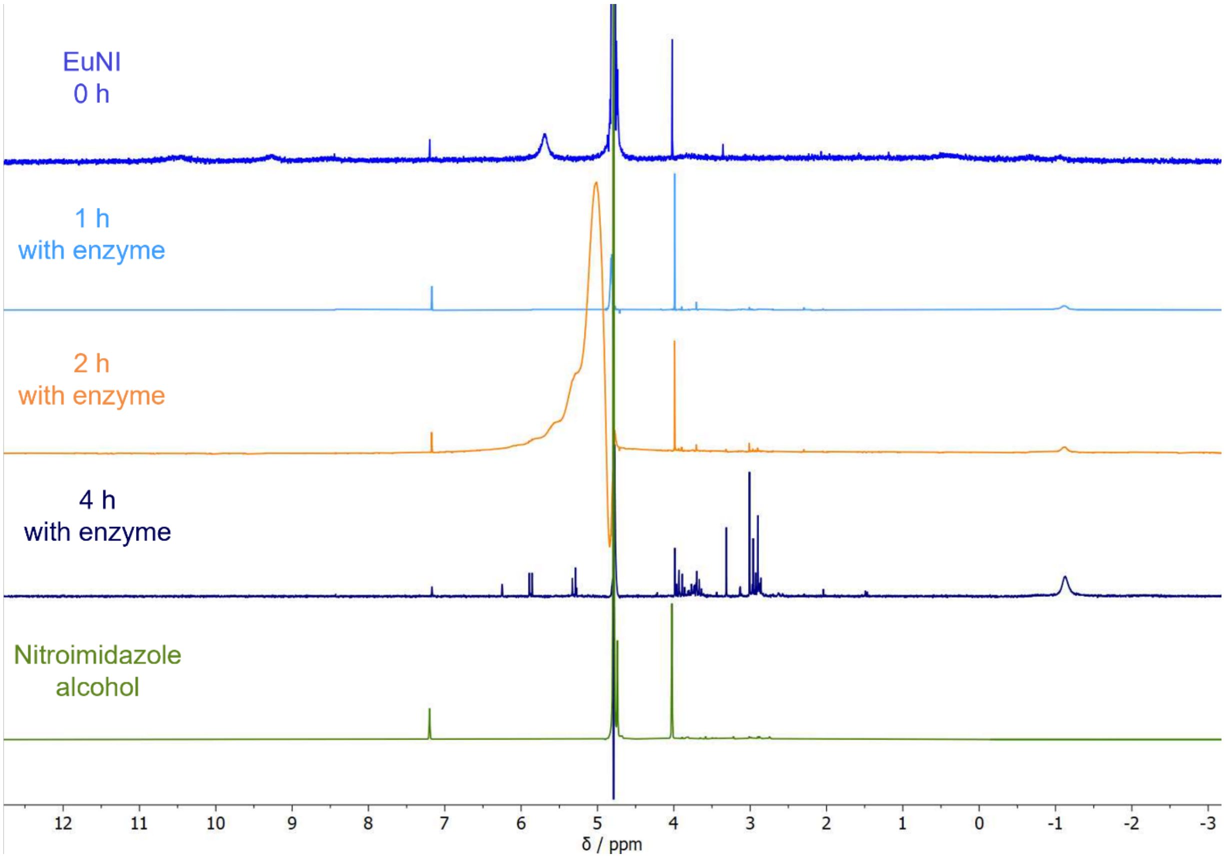

Figure S48: The stacked ^{1}H NMR spectra (with water suppression, -4 to 16 ppm range) from the Hyd1 enzyme assay of EuNI (**2a**), showing the timepoints compared to the initial t=0 measurement (bright blue) and nitroimidazole alcohol (green) as a control. The enzymatic assay protocol was adapted from Sokolova *et al.*[15]

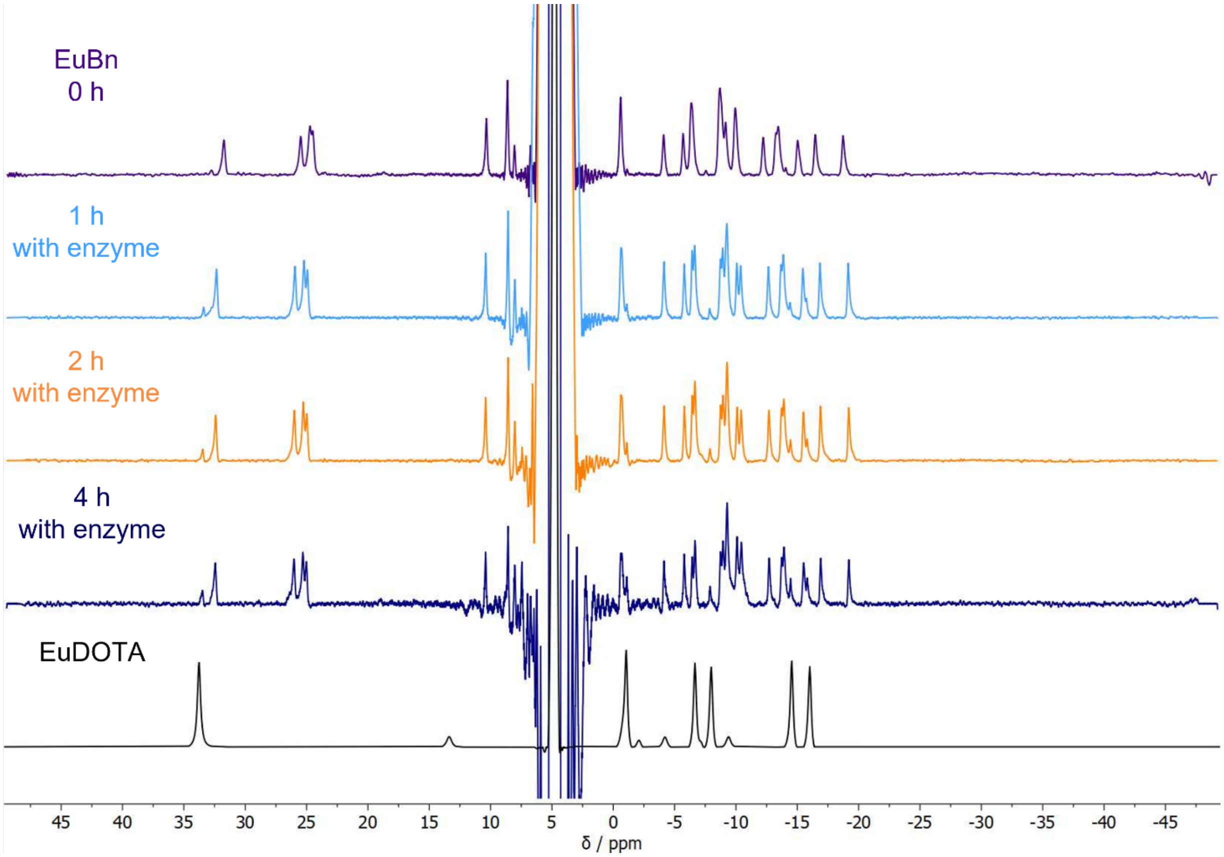

Figure S49: The stacked ^{1}H NMR spectra from the Hyd1 enzyme assay of EuBn (**4a**), showing the timepoints (with enzyme) compared to the initial t=0 measurement (dark purple) and EuDOTA (**3a**, the positive control, black). The enzymatic assay protocol was adapted from Sokolova *et al.*[15] The EuDOTA control ^{1}H NMR was in D_2O only.

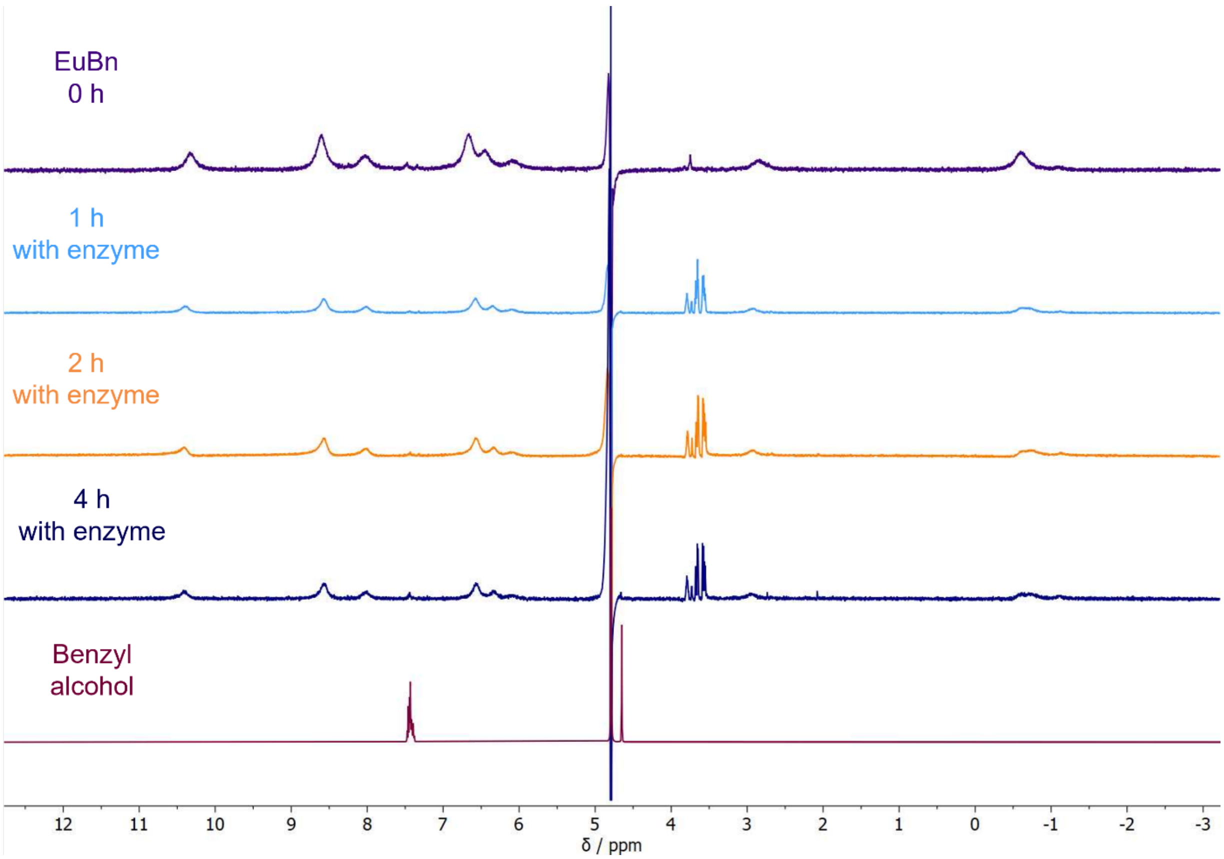

Figure S50: The stacked ^{1}H NMR spectra (with water suppression, -4 to 16 ppm range) from the Hyd1 enzyme assay of EuBn (**4a**), showing the timepoints compared to the initial t=0 measurement (dark purple) and benzyl alcohol (purple) as a control. The enzymatic assay protocol was adapted from Sokolova *et al.*[15]

General Experimental

Chemistry Experimental

Reagents and solvents were obtained from Sigma Aldrich U.K., ChemCruz, Alfa Aesar and BDH Chemicals and were used without further purification, unless otherwise specified. Dry toluene, dry THF and dry DMF was taken from MBRAUN MB5 Solvent Purification System, and stored over 3 Å molecular sieves, under an inert atmosphere of argon, unless otherwise stated. Dry EtOH and dry MeOH were purchased from Sigma Aldrich U.K. in SureSeal™ bottles and used without further purification. Gadolinium DOTA (Dotarem®) was obtained from Guerbet.

Concentration *in vacuo* refers to solvent removal under reduced pressure at 40 °C using a Buchi™ rotary evaporator, unless otherwise stated. Brine is a saturated aqueous solution of NaCl. Hexane includes a mixture of hexane isomers. Petroleum ether refers to the fractions collected between 40–

60 °C when distilled. Celite® refers to Celite®545 filter aid, treated with sodium carbonate, flux-calcined, which was purchased from Sigma Aldrich. Deionised water (18.2 MΩ) was obtained using an Elix® Essential water purification system. Unless otherwise stated, all reactions were carried out open to air at atmospheric pressure.

All tested compounds (for fluorescence, chemical or enzymatic assays) were determined to be over 90% pure by ^{1}H NMR for europium/terbium complexes and/or analytical HPLC prior to testing.

Silica gel column chromatography was carried out using Merck silica gel (40–63 μm), eluting with solvents as supplied under a positive pressure of N_2 gas.

Melting points were obtained using a Griffin capillary tube melting apparatus and are uncorrected. The crystallisation solvent is given in parentheses.

Infrared (IR) spectra were obtained from neat samples or thin liquid films. The spectra were recorded using a Bruker Tensor 27 spectrometer with a diamond ATR module. Absorption maxima are given in wavenumbers (cm^{-1}) and reported as s (strong), m (medium), or w (weak).

Analytical thin layer chromatography (TLC) was carried out on Merck silica gel 60 F_{254} aluminium-supported thin layer chromatography sheets. Spots were visualised by absorption of UV light (λ_{max} 254 nm and 365 nm), or thermal development after staining with an aqueous solution of potassium permanganate.

^{1}H NMR spectra were recorded using a Bruker AVIII HD 400 nanobay (400 MHz), AVII 500 (500 MHz), AVIII HD 600 (600 MHz) or Bruker NEO 600 (600 MHz) spectrometer with broadband helium cryoprobe, using the stated solvent as a reference for internal deuterium lock. The chemical shift data for each signal are given as δ_H in units of parts per million (ppm). The spectra are calibrated using the solvent peak with the data provided by Fulmer *et al.*[16] The multiplicity of each signal is indicated by: s (singlet); br s (broad singlet); d (doublet); t (triplet); q (quartet); m (multiplet) or combinations thereof. The number of protons, n, for a given resonance signal is indicated by nH. Where appropriate, coupling constants (J) are expressed in Hz and are recorded to the nearest 0.1 Hz. Identical proton coupling constants (J)

are averaged in each spectrum and reported to the nearest 0.1 Hz. MestReNova and Topspin software were used for NMR analysis. ^{1}H NMR spectra were assigned using 2D NMR such as COSY, HSQC and HMBC, as necessary. ^{1}H NMR spectra for europium complexes and ^{1}H NMR spectra for terbium complexes used wide ranges (-50 to +50 ppm for europium, -500 to +500 ppm for terbium). The ^{1}H NMR with water suppression method used NOESY presaturation.

^{13}C NMR spectra were recorded using a Bruker AVIII HD 600 (151 MHz) or Bruker NEO 600 spectrometer (151 MHz) with broadband proton decoupling, using the stated solvent as a reference for internal deuterium lock. The chemical shift data for each signal are quoted as δ_C in parts per million (ppm). The spectra are calibrated using the solvent peak with the data provided by Fulmer *et al.*[16] The chemical shift is quoted to 1 decimal place, unless two different shifts are indistinguishable, where the shifts are then quoted to 2 decimal places. MestReNova and Topspin software were used for NMR analysis. ^{13}C NMR spectra were assigned using 2D NMR such as HSQC and HMBC.

Mass spectra were acquired on either an Agilent 6120 (low resolution), Waters LCT Premier XE bench-top orthogonal acceleration time-of-flight LC-MS system (low resolution), Thermo Exactive High-Resolution Orbitrap FTMS (high resolution) or Waters RDa bench-top TOF used with an Acquity LC system (high resolution) using electrospray ionisation (ESI) from solutions of methanol, water or acetonitrile. m/z values are reported in Daltons and followed by their percentage abundance in parentheses. Data were processed using MestReNova software.

Analytical high-performance liquid chromatography (HPLC) was carried out to determine compound purity. Samples (in MeCN or H_2O) were all filtered through a PTFE syringe filter (0.2 μM) or nylon syringe filter (0.45 μM, 13 mm) before injection. HPLC was carried out on an Agilent 1260 Infinity II system with the column oven at 40 °C and UV/Vis detection at 220, 240, 254, 280, 360 and 480 nm, with percentage purity averaged over a minimum of three wavelengths. For methods A and B, a Poroshell 120 EC-C18 reverse phase column (4 μM, 4.6 × 150 mm) was used, with a constant flow rate of 1 ml min^{-1} and a gradient method as shown in Table S6 or Table S9. For method C (used for the nitroreductase assay), a Poroshell 120 EC-C18 reverse phase column (4 μM, 4.6 × 150 mm) was used,

with a constant flow rate of 1 ml min^{-1} and a gradient method as shown in Table S10, with UV/Vis detection at 220, 240, 254, 269, 280, 320, 360 and 480 nm. For all methods, solvent A is H_2O with 0.1% formic acid and B is MeCN with 0.1% formic acid.

Table S8: The solvent gradient used for analytical HPLC **method A.**

Time / min	%A	%B
0.0	95	5
5.0	95	5
10.0	50	50
10.5	5	95
11.0	5	95
11.5	95	5
14.0	95	5

Table S9: The solvent gradient used for analytical HPLC **method B**.

Time / min	%A	%B
0.0	95	5
1.0	95	5
11.0	5	95
16.0	5	95

Table S10: The solvent gradient used for analytical HPLC **method C**.

Time / min	%A	%B
0.0	95	5
5.0	95	5
10.0	50	50
12.0	5	95
13.0	5	95
15.0	95	5
16.0	95	5

Liquid chromatography mass spectrometry (LCMS) was carried out to determine compound purity of more polar compounds. Samples (in MeCN or H_2O) were all filtered through a PTFE syringe filter (0.2 μM) or nylon syringe filter (0.45 μM, 13 mm) before injection. Electrospray ionisation (ESI) liquid chromatography mass spectrometry (LCMS) were acquired using Agilent 1260 Infinity II® fitted with a Quat. Pump (G7111A), autosampler (G7129A), Column oven (G7130A), DAD (G7115A) and mass spectrometer (G6125B). The signals acquired were at 254 nm, 365 nm, 220 nm, 450 nm and 280 nm (with bandwidths of 4 nm and reference wavelength of 800 nm, bandwidth 100 nm). Solvent A is H_2O

with 0.1% formic acid and B is MeCN with 0.1% formic acid. LCMS data were processed using the Agilent LCMS software and MestReNova software.

Method 1: See Table S11 for the solvent gradient. A Poroshell 120 SB-C18 (50 × 2.1 mm, 2.7 μM) column was used, with the column oven at 40 °C, a flow rate of 0.4 mL min^{-1} and injection volumes between 2 μL and 20 μL.

Table S11: The solvent gradient used for LCMS **method 1**.

Time / min	%A	%B
0.0	95	5
0.5	95	5
3.5	5	95
6.5	5	95
7.0	95	5
10.0	95	5

Method 2: See Table S12 for the solvent gradient. A Poroshell 120 SB-C18 (50 × 2.1 mm, 2.7 μM) column was used, with the column oven at 40 °C, a flow rate of 0.4 mL min^{-1} and injection volumes between 2 μL and 20 μL.

Table S12: The solvent gradient used for LCMS **method 2**.

Time / min	%A	%B
0.0	95	5
1.0	95	5
6.0	50	50
6.5	0	100
7.0	0	100
7.5	95	5
10.0	95	5

Methods 3 and 4: See Table S13 for the solvent gradient. Method 3: A Poroshell 120 SB-C18 (50 × 2.1 mm, 2.7 μM) column was used, with the column oven at 40 °C, a flow rate of 0.4 mL min^{-1} and injection volumes between 2 μL and 20 μL. Method 4: An InfinityLab Poroshell 120 EC-C18 (4.6 × 100 mm, 4.0 μM) column was used, with the column oven at 40 °C, a flow rate of 1.0 mL min^{-1} and injection volumes between 10 μL and 50 μL.

Table S13: The solvent gradient used for LCMS **methods 3 and 4**.

Time / min	%A	%B
0.0	95	5
5.0	95	5
10.0	50	50
10.5	5	95
11.0	5	95
11.50	95	5
14.0	95	5

Semi-preparative high performance liquid chromatography (SP-HPLC) was carried out on an Agilent Infinity II 1260 fitted with a preparatory pump (G7161A), prep sampler (G7157A), VWD (G7114A), fraction collector (G1364E) and a 1 mL sample loop. Complex samples (in MES buffer 0.5 M, filtered through a 0.45 µm nylon syringe filters) were purified with sample detection at 254 nm. An Agilent 5 Prep C18 (p/N 446905-702, 50 × 21.2 mm, 5 µL) column was used, with injection volumes between 100 µL and 750 µL.

Method 1: used for nitrobenzyl and benzyl complexes, Table S14, where solvent A is H_2O (no additive) and solvent B is MeCN (no additive), flow rate 25 mL min^{-1}.

Table S14: The solvent gradient used for SP-HPLC **method 1**.

Time / min	%A	%B
0	95	5
4	95	5
11	50	50
12	5	95
14	95	5
15	95	5

Method 2: used for nitroimidazole complexes,

Table S15, where solvent A is H_2O (no additive) and solvent B is MeCN (no additive), flow rate 15 mL min^{-1}.

Table S15: The solvent gradient used for SP-HPLC **method 2**.

Time / min	%A	%B
0	95	5
5	95	5
7	5	95
8	5	95
8.5	95	5
9	95	5

Method 3: use for benzyl complexes, Table S16, where solvent A is H_2O (no additive) and solvent B is MeCN (no additive), flow rate 15 mL min^{-1}.

Table S16: The solvent gradient used for SP-HPLC **method 3**.

Time / min	%A	%B
0	95	5
2	95	5
9	50	50
11	50	50
13	5	95
15	5	95
16	95	5

UV-Vis spectroscopy was carried out on a Jasco V-770 UV-Visible spectrophotometer running Spectra Manager™ software, with the temperature maintained at 25 °C. Samples were measured in high precision cuvettes from Hellma Analytics (path length 10 mm), using technical grade solvents, and the cuvette was washed with water and EtOH between measurements and with 25% nitric acid solution between different lanthanide metals. Measurements were obtained using Spectra Measurement, with the following parameters: UV bandwidth 1 nm, NIR bandwidth 20 nm, data interval 0.2 nm, scan rate 200 nm/min, source change at 340 nm. Spectra were analysed using Origin™ software. All UV-Vis data was measured on samples determined to be over 90% pure by ^{1}H NMR, LCMS or analytical HPLC.

Fluorescence and **phosphorescence spectroscopy** and **lifetime measurements** were carried out on a Horiba Jobin Yvon FluoroLog® 3-12 equipped with a Hamamatsu R928 detector and a double-grating emission monochromator. A 2" square unmounted longpass 400 nm filter (FGL400S) from Thor labs was used for all steady state and time gated emission spectra of terbium complexes measured

between 450 nm and 700 nm. A 2” square unmounted longpass 495 nm filter (FGL495) from Thor labs was used for all steady state and time gated emission spectra of europium complexes measured between 500 and 800 nm. Complexes were measured at 50 μM in water, unless otherwise stated. All measurements were measured with dark offset enabled. Corrected intensity refers to the intensity following blank subtraction. Steady-state emission spectra were measured with the following parameters: slits 29 nm / 1 nm, integration time 0.5 s. Time-gated emission spectra were measured with the following parameters, unless otherwise specified: integration time 100 ms, flash delay 0.05 ms, sample window 1 ms, time per flash 61 ms, flash count 10. Time-gated emission spectra were measured with the following slit parameters, unless otherwise stated: Eu 50 μM 29 nm / 4 nm; Eu 200 μM 29 nm / 2 nm; Tb 50 μM 29 nm / 3.5 nm; Tb 200 μM 29 nm / 1.5 nm. For all emission spectra the uncorrected S1 channel intensity is plotted, following blank subtraction of the solvent measured under the same conditions. For excitation spectra the corrected S1/R1 channel intensity is plotted, following blank subtraction of the solvent measured under the same conditions. For lifetime measurements the S1c channel is plotted. **Degassing measurements** were carried out by purging the sample under a flow of argon for minimum 30 minutes and transferring the solution inertly to a degassing cuvette, which had been purged by alternating between vacuum and argon three times prior to sample addition. The data was obtained using FluorEssence™ software and processed using Origin™ software.

Centrifugation for purification of triacid compounds was carried out on a Beckman Coulter® Allegra® X-12R Benchtop Centrifuge and centrifugations were performed at 3750 rpm for 5 min at 4 °C. For centrifugation during nitroreductase enzymatic assays, a Labnet Prism™ Mini Centrifuge was used and centrifugations were performed at 6000 rpm for 4 min at room temperature.

Buffer preparation

- **Phosphate buffer saline (PBS)** (pH 7.4): One phosphate buffered saline tablet (Thermo Scientific™, Oxoid™, BR0014G) was dissolved in deionised water (100 mL) and the pH adjusted to pH 7.4 using NaOH (2M aqueous solution) and HCl (1M aqueous solution).

- **2-(N-Morpholino)ethanesulfonic acid (MES)** (1M, pH 6.0): 2-(N-Morpholino)ethanesulfonic acid (MES) monohydrate (5.43 g, 25.0 mmol) was dissolved in deionised water (20 mL) and the pH adjusted to pH 6.0 (tolerance 6.00-6.04) using NaOH (6M aqueous solution). Deionised water was added to give a total volume of 25 mL.
- **Sodium phosphate buffer** (100 mM, pH 7.4): Na_2HPO_4 (70 mg) and NaH_2PO_4 (484 mg) were dissolved in deionised water (40 mL) and the pH adjusted to pH 7.4 using NaOH.

pH measurements were determined using a Jenway pH Meter 3305, with an Aldrich glass/calomel combination electrode, or a HI 2210 pH Meter. Calibration for the Jenway pH Meter 3305 was carried out between pH 4.0–7.0 or 7.0–10.0 immediately before use, with buffer solutions of phthalate (pH 4.0), phosphate (pH 7.0) and borate (pH 10.0) from Fisher Scientific. Electrodes were stored in a 4 M aqueous potassium chloride solution. Calibration for the HI 2210 pH Meter was carried out between pH 4.0-7.0 or 7.0-10.0 immediately before use, using the calibration solutions (pH 4.01, 7.01 and 10.01) from Hanna Instruments. Following use, the electrodes were submerged in the cleaning solution (HI 7061) for at least 20 minutes then stored in the storage solution (HI 70300), from Hanna Instruments.

Dialysis was carried out, for the DOTA positive control compounds only, using Spectra-Por® Float-A-Lyzer® G2 dialysis membrane tubing made from regenerated cellulose ester (Spectrum Labs®) with a molecular weight cut-off of 500 Da. The dialysis tube was activated by 15% EtOH solution in deionised water (20 min, 2 L), and rinsed with deionised water before soaking with deionised water (15-20 min, 2 L) before use. The deionised water was removed and the complexes (dissolved in deionised water) transferred into the dialysis tube. The dialysis tube was stirred gently for a minimum of 2 weeks in deionised water (2.5 L) and the water replaced a minimum of 5 times.

Relaxivity measurements

<u>11 T measurements:</u> T_1 measurements were carried out using a Bruker AVIII HD 500 NMR instrument equipped with 5 mm z-gradient broadband X-19F/1H BBFO SMART probe. Samples were prepared between 0 and 1 mM (0.2 mM increments, 200 µL, H_2O), transferred to a capillary tube (~70 µL), flame sealed, transferred to a 5 mm NMR tube and analysed at 298 K. No lock solvent was required. For each

concentration, an initial T_1 measurement was carried out using time delays of 0.01, 0.1, 0.5, 1, 5 and 20 s and a more accurate measurement was carried out by measuring at 20-25 time delays (from 0 to 10 s), based on the initial rough T_1 measurement. T_1 data was analysed using TopSpin software and plotted using Origin software.

7 T measurements: Relaxivity determination by MRI was conducted using an Agilent 7 T scanner equipped with a DirectDrive console and 400 mT/m imaging gradients (Varian, UK). The RF coil employed was a 72 mm i.d. birdcage resonator (Rapid Biomedical, Germany). T1 measurements were carried out using an inversion recovery spin-echo sequence with the following parameters: slice thickness of 1 mm, field of view (FoV) of 72 x 72 mm, matrix size of 128 x 128, four averages, TR/TE of 10 s/8 ms, and 12 inversion times (Ti) ranging from 0.01 to 6.0 seconds (exponentially spaced). T2 measurements were performed using a spin-echo sequence with the same single-slice sequence as the T1 measurements: slice thickness of 1 mm, FoV of 72 x 72 mm, matrix size of 128 x 128, four averages, TR of 10 s, and 12 echo times (TE) ranging from 8 to 300 ms (exponentially spaced). All samples were placed in 1 ml syringes (Terumo) in a custom-made 3D-printed phantom holder enabling reproducible measurements. Acquired data was analysed using Matlab (version R2022a).

Cyclic voltammetry (CV)

The electrochemical cell consisted of a polyether ether ketone (PEEK) cylinder containing a working electrode of glassy carbon (1 mm diameter), a counter electrode of graphite, and a leak-free Ag/AgCl reference electrode (LF-2-45 model from Alvatek Ltd). The cell was constructed in a N_2 atmosphere in a glove box (O_2 < 2 ppm), using a 1 mM solution of complex dissolved in degassed H_2O containing PBS at pH 7.4 or 100 mM MES and 50 mM NaCl at pH 6.0. After construction, the air-tight electrochemical cell was removed from the glove box and connected to an Autolab potentiostat. Nova 2.1.7 software was used to run the cyclic voltammograms with step potential = -0.00244 V and scan rate = 0.02 V/s, cycling thrice. The Ag/AgCl reference electrode was calibrated to vs SHE by measuring the CV of FcMeOH (0.1 mM in 4:1 buffer:EtOH) and comparing to a literature midpoint potential ($E_{1/2}$) value of +420 mV vs SHE for FcMeOH.[17] Cathodic onset potentials were calculated as follows: the first linear

sweep of the voltammogram of the blank (buffer only) was subtracted from the first linear sweep of the voltammogram of the substrate; a linear baseline in a region of no electrochemical activity was applied; the potential at which the difference between the substrate voltammogram minus blank voltammogram current and the baseline current began to increase exponentially, exceeding a threshold value of 10 nA (instrument current resolution 0.0003% of current range), was taken to be the cathodic onset potential.

Chemical activation assays

The chemical activation (zinc) assay was carried out by dissolving compound in D_2O and adding 10% *v/v* NH_4Cl solution (10% *w/v* in D_2O) and zinc (38 eq.). The chemical reduction assay protocol was adapted from O'Connor et al.[14]

A stock solution of the europium complex was prepared by dissolving the solid in 3.78 mL D_2O (540 µL x 7) to give final concentrations of 11-14 mM (see Table S18), suitable for good intensity ^1H NMR spectra for europium complexes. A 10 wt% solution of NH_4Cl was prepared in D_2O and 60 µL (10% of the total volume) was added to the relevant aliquot of europium complex. Following the addition of zinc (38 eq.) to the required solutions, the resulting solutions (prepared as described in

Table S17) were heated to 37 °C (using a heat block) for the indicated time and stirred, cooled to room temperature, filtered (0.45 µm nylon syringe filter) and diluted with D_2O (100 µL) to give a final concentration of complex around 9-12 mM. ^{1}H NMR spectra (for europium complexes and a standard ^{1}H experiment) were ran at 500 MHz. The NMR samples were diluted with H_2O for LRMS analysis. For EuNB only, the 6-hour timepoint was further analysed by HRMS. The masses used for each europium complex are described in Table S18.

Table S17: The composition of the solutions prepared and analysed in the chemical reduction assays of the europium complexes.

Description	Volume of compound stock / µL	Volume of NH_4Cl solution / µL	Volume of D_2O / µL	Zinc added?
t = 0 h	540	-	60	No
Timepoints (t = 1, 2, 4, 6 h)	540	60	-	Yes
D_2O control (t = 6 h)	540	-	60	No
NH_4Cl control (t = 6 h)	540	60	-	No

Table S18: The masses of complex and zinc used for each chemical reduction assay.

**Europium complex**	**Mass of complex in stock solution / mg**	**Mass of complex per vial / mg**	**Mass of zinc for t=1 timepoint / mg**	**Mass of zinc for t=2 timepoint / mg**	**Mass of zinc for t=4 timepoint / mg**	**Mass of zinc for t=6 timepoint / mg**
EuNB (**1a**)	35.6	5.09	18.2	18.4	18.6	18.6
EuNI (**2a**)	39.7	5.67	20.2	20.2	20.4	20.5
EuBn (**4a**)	25.8	4.07	15.6	15.7	15.7	15.9

As controls, ^{1}H NMR spectra of nitrobenzyl alcohol and aminobenzyl alcohol were analysed by diluting solid in 540 µL D_2O and 60 µL NH_4Cl solution (10wt% in D_2O) and filtering (Nylon syringe filter) in an NMR tube. The ^{1}H NMR spectra of nitroimidazole alcohol was recorded in D_2O only.

The percentage of EuDOTA released was calculated by dividing the area of the EuDOTA peak at 33.8 ppm (divided by 4 for 1H) by the sum of the area of the EuDOTA peak (divided by 4 for 1H) and the corresponding peak of the starting Eu complex (1H – 32.5 ppm for EuNB, 32.3 ppm for EuNI, 32.7 ppm for EuBn).

Stability measurements

Zinc reduction assay EuNI extra controls: A stock solution of EuNI was prepared by dissolving 11.3 mg of solid in 1.08 mL D_2O (~14 mM). A 10 wt% solution of NH_4Cl was made by dissolving 61.0 mg NH_4Cl in 610 µL D_2O. 540 µL of compound stock solution was diluted with either 60 µL D_2O (D_2O only control) or 60 µL NH_4Cl solution (NH_4Cl control). The resulting solutions were heated to 37 °C in an oil bath with

no stirring and analysed by ^{1}H NMR spectroscopy (^{1}H for europium complexes and standard ^{1}H, 500 MHz) at the indicated timepoints (with the time of analysis not included in the assay time).

Water room temperature measurements: 250 µM solutions of TbNI and TbNB were prepared by dilution from a 10 mM stock solution in water, by adding 20 µL of stock solution to 780 µL water. The stability in water was analysed by 10 µL injections onto the LCMS (method 3) at given timepoints. The samples were left at room temperature between injections, with no stirring between timepoints. The stability was analysed by percentage purity of the UV peak corresponding to the desired compound (area of desired peak compared to total area of all peaks), at 254 nm. Data were analysed using MestReNova and processed using Origin™ software.

Water 37 °C measurements: 500 µM solutions of TbNI and TbNB were prepared and heated to 37 °C using a water bath with no stirring. At given timepoints, 50 µL aliquots were taken and diluted with 50 µL water to give a final concentration of 250 µM. The samples were analysed by LCMS (method 3). The stability data were analysed and processed as above for the water measurements.

PBS measurements: EuNB (6.8 mg) was dissolved in 700 µL PBS buffer (pH 7.4) and analysed by ^{1}H NMR spectroscopy for europium complexes (at 500 MHz) at the indicated time points, with no stirring between timepoints. EuNI (5.2 mg) was dissolved in 700 µL PBS buffer (pH 7.4) and analysed by ^{1}H NMR spectroscopy for europium complexes at the indicated time points, with no stirring between timepoints. For analysis and comparison, EuDOTA (6.3 mg) was dissolved in 700 µL PBS buffer and one ^{1}H NMR spectrum for europium was obtained immediately. The stability/percentage purity was determined by comparing the integral of the peak at 34 ppm (4H) belonging to EuDOTA to the integral of the peak at 33 ppm (1H) belonging to EuNB/EuNI.

Nitroreductase enzymatic assay

The nitroreductase enzymatic assay procedure was based on that reported by Nazaré and co-workers.[18] The assay was carried out using nitroreductase (NTR) enzyme from *E. coli* (expressed in *E. coli*, >100 units/mg), purchased from Sigma Aldrich, and NADH, purchased from Prozomix (as the disodium salt). The nitroreductase enzyme was prepared by dissolving 25.3 mg of the lyophilised powder (out of 26.6

mg total powder, total containing 1 mg enzyme) in 951 µL water (to give stock solutions of 1 mg/mL), aliquoted (in 100 µL portions), stored at -20 °C and thawed immediately prior to use. NADH was stored at -20 °C and weighed and diluted in water to 5 mM immediately prior to addition to the assay. The lanthanide complexes were stored at -80 °C and weighed and diluted in water to 1 mM immediately prior to addition to the assay.

The assay samples were prepared as described in Table S19, with samples prepared by first adding the 0.9% NaCl solution and water, then NADH solution (assay concentration 500 µM) followed by addition of the lanthanide complex stock solution (assay concentration 200 µM). The t=0 timepoints were taken prior to addition of the NTR enzyme and were taken for all assays. The enzyme solution was added (assay concentration ~32 µg/mL) and the Eppendorfs containing the assays were heated to 37 °C using a water bath. At the indicated timepoints, 100 µL aliquots were taken from each assay, diluted with 100 µL MeCN and centrifuged at 6000 rpm for 4 minutes. The supernatant was filtered (Nylon syringe filter, 0.45 µM, 13 mm) into HPLC vials (final concentration ~100 µM complex). The assay was monitored by analytical HPLC (**method C**, 50 µL injection), with samples injected in the order 1, 4, 2, 3 (see Table S19), and analysed using the Agilent HPLC software (with NaCl/water blank subtraction, for numerical analysis) and MestReNova software (for stacking of the raw spectra, without blank subtraction).

Table S19: The volumes of the reagents used for the nitroreductase assays of EuNB and GdNB.

	**1**<br>**Full assay**	**2**<br>**NTR control**	**3**<br>**NADH control**	**4**<br>**NaCl control**
Lanthanide complex (1 mM) / µL	200	200	200	200
NADH (5 mM) / µL	100	-	100	-
NTR (1 mg/mL) / µL	30	30	-	-
0.9% NaCl solution / µL	670	670	670	670
Water / µL	-	100	30	130
Total volume / µL	1000	1000	1000	1000

For numerical analysis, the HPLC spectra following blank subtraction (NaCl solution/H_2O/MeCN blank) were used. The concentration of nitrobenzyl alcohol released was calculated by comparing the area of the peak (measured at 269 nm) at 9.6-9.8 min with the control injected at 100 µM. The percentage of GdNB remaining was calculated by comparing the area of the peak (measured at 269 nm) at 8.0-8.1

min to the area of the same peak in the t=0 sample. The stacked spectra (in Figures S38-S41) show the uncorrected spectra at 269 nm (no blank subtraction) and any peaks appearing after 12 minutes are artefacts on the column.

The following controls (see **Error! Reference source not found.**) were also analysed by analytical HPLC (**method C,** 50 μL injection): GdNB, at an unspecified concentration, in water, for purity testing, filtered; a HPLC blank where 670 μL 0.9% NaCl solution was added to 330 μL water and 100 μL of this solution was added to 100 μL MeCN and filtered; a NADH blank, with 100 μL of the 5 mM stock solution (in water) diluted with 670 μL 0.9% NaCl solution and 230 μL water, and 100 μL of this solution was added to 100 μL MeCN and filtered; nitrobenzyl alcohol (final concentration 100 μM) was prepared from a 10 mM stock solution in DMSO, diluted to 200 μM by adding 20 μL of 10 mM stock solution to 670 μL 0.9% NaCl solution and 310 μL water, and 100 μL of this solution added to 100 μL MeCN and filtered; aminobenzyl alcohol, at an unspecified concentration, in MeOH, filtered.

NADH NMR study

EuNB (6.3 mg) was dissolved in D_2O (868 μL) to give a 13.3 mM stock solution and an initial ^{1}H NMR spectra and ^{1}H NMR for europium spectra was ran. NADH (37.1 mg) was dissolved in D_2O (523 μL) to give a 100 mM stock solution. 200 μL of the NADH stock solution was added to 600 μL of the EuNB solution to give final concentrations of 25 mM NADH and 10 mM EuNB and t=0 NMR spectra were acquired (15 minutes following the previous NMR spectra). The NMR tube was heated to 37 °C in a water bath for 6 h and NMR spectra (^{1}H and ^{1}H for europium) were acquired before heating to 37 °C for a further 18 h (24 h timepoint). Following final NMR analysis in D_2O only, 250 μL MeOD and then a further 650 μL MeOD was added to the NMR tube (to give final 1:1 MeOD:D_2O) to aid solubility of the organic components (likely due to the higher solubility of the high concentration NADH). The controls were as follows: EuDOTA in D_2O, nitrobenzyl alcohol in D_2O and 10% NH_4Cl and aminobenzyl alcohol in D_2O and 10% NH_4Cl (as used for the chemical reduction assay controls). The percentage of EuDOTA released was calculated by dividing the area of the EuDOTA peak at 33.8 ppm (4H, therefore divided

by 4) by the sum of the area of the EuDOTA peak (divided by 4 for 1H) and the corresponding peak of the starting EuNB (1H, 32.5 ppm).

Hyd-1/C enzymatic assay

This procedure is adapted from the procedure previously reported.[15] An initial study at 24 h in buffer at pH 6 saw complete conversion to EuDOTA for both EuNB and EuNI in the presence and absence of the enzyme, therefore the procedure was optimised to be carried out up to 4 hours in buffer at pH 7.4 (based on the PBS stability studies).

Catalyst preparation and reaction set-up was carried out in a glove box (Glove Box Technology Ltd.) under a protective N*2* atmosphere (O_2 < 3 ppm). Carbon black (BP2000) was brought into a glovebox, suspended in buffer (sodium phosphate buffer, 100 mM, pH 7.4, 20 mg/mL suspension) and was sonicated in the glovebox for 1 h. Overexpressed Hyd-1 (23.1 µL, expressed according to the protocol reported by Sokolova *et al.*)[15] was brought into the glovebox and combined with the sonicated carbon suspension (79.2 µL), removed from the glovebox and immobilised in the fridge (4 °C) for 1 h. The desired complex was dissolved in buffer in the glovebox to make a 10 mM stock solution, which was added to two DrySyn® OCTO tubes A and B. The immobilised Hyd-1/C was centrifuged in the glovebox, supernatant removed, resuspended in buffer (300 µL) and added to tube A. Tube A contained 1200 µL stock solution of europium complex and 300 µL of Hyd-1/C solution. No catalyst (Hyd-1/C) was added to the control tube B. The DrySyn® OCTO was sealed, removed from the glovebox and connected to H_2 and the samples were stirred at room temperature for 4 h. Aliquots were taken at the given timepoints (1 h, 2 h and 4 h) and the time when DrySyn® OCTO was not connected to H_2 was not included in the time count. The aliquots were centrifuged (3 min, 14500 rpm), 450 µL supernatant removed, diluted with 50 µL D_2O and measured by ^1H NMR spectroscopy (^1H for europium complexes and ^1H with water suppression).

Controls: The ^1H NMR (for europium complexes) spectrum of EuDOTA was measured in D_2O only. The ^1H NMR spectra of nitrobenzyl alcohol and aminobenzyl alcohol were measured following preparation of ~10 mM stock solutions of the compounds in sodium phosphate buffer (pH 7.4) and

dilution 10% *v/v* with D_2O. The ^1H NMR spectra of nitroimidazole alcohol and benzyl alcohol were measured in D_2O.

EuNB, 1a: t=0 is the solid dissolved in buffer (to 10 mM) and diluted with 10% *v/v* D_2O.

EuNI, 2a: t=0 is the solid dissolved in D_2O.

EuBn, 3a: t=0 is the solid dissolved in buffer (to 10 mM) and diluted with 10% *v/v* D_2O. Peaks observed in the ^1H spectra in the presence of the Hyd1 enzyme between 3 and 4 ppm (in the assay timepoints) are postulated to arise from residual glycerol from the protein.

Biological Experimental

Cell culture was carried out using HCT116 cells were cultured in DMEM medium supplemented with 10% FBS at 37°C, 5% CO_2 in a humidified incubator. All cell lines were routinely mycoplasma tested using a HEK-Blue™ detection kit (Invivogen) and found to be negative. Hypoxia treatments were performed at <0.1% O_2 in a Bactron II Chamber (Shel Laboratory). Oxygen concentrations were periodically validated using anaerobic oxygen indicator strips (ThermoFisher).

For preparation of samples for **LCMS analysis**, 4.5 Mio. cells were seeded in 10 cm^2 glass dishes and allowed to adhere for 7 hours. Cells were treated with the indicated probe concentrations and exposed to hypoxia (<0.1% O_2) for 16 hours. Afterwards, cells were washed three times in HEPES inside the hypoxia chamber, scraped into 1 mL of HEPES and spun down. The remaining cell pellet was lysed and evenly resuspended in 200 µL of acetonitrile/distilled water (MeCN: deionised H_2O) (1:1) and sonicated for 10 minutes. Samples were spun down at 4°C at 13,000 rpm for 15 minutes. The cleared supernatant was carefully removed. The pellet was lysed in 100 µL deionised H_2O, spun down again at 4°C at 13,000 rpm for 15 minutes, and the remaining supernatant was added to the prior supernatant. The combined supernatant was centrifuged in an Amicon Ultra Centrifugal Filter (Merck) for 10 minutes at 4000 g. The resulting filtered supernatant was collected and stored at -80 °C (or on dry ice during transport) prior to analysis by LCMS (method 4).

Synthesis of Compounds

Tri-*tert*-butyl 2,2',2''-(1,4,7,10-tetraazacyclododecane-1,4,7-triyl)triacetate hydrobromide, 5

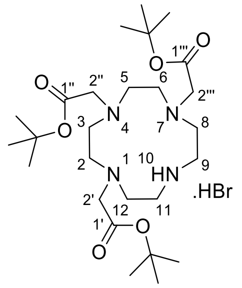

1,4,7,10-tetraazacyclododecane (cyclen, 5.01 g, 29.1 mmol, 1.0 eq.) was dissolved in MeCN (150 mL) and cooled to 0 °C. $NaHCO_3$ (8.09 g, 96.3 mmol, 3.3 eq.) was added and the mixture was stirred for 15 mins, before *tert*-butylbromoacetate (14.7 mL, 99.6 mmol, 3.4 eq.) in MeCN (50 mL) was added dropwise over 1 h. The reaction mixture was stirred at 0 °C for 2 h, allowed to warm to room temperature and stirred and room temperature for 44 h. The inorganic solids were removed by filtration and the filtrate was concentrated *in vacuo*. The resulting off-white solid was triturated with cold toluene (42 mL), filtered and washed with toluene (2 × 4 mL) to give **5** as a colourless solid (6.87 g, 40%): R_f 0.37 (MeOH : CH_2Cl_2 5:95); mp 170–172 °C (toluene) [lit.[19] 176–178 °C, lit.[20] 187–188 °C, lit.[21] 190–191 °C, lit.[22] 178-180 °C]; ^1H NMR (400 MHz, DMSO-d$_6$): 1.38–1.46 (27H, m, 3 × (C*H$_3$*)$_3$), 2.62–2.75 (8H, m, 4 × ring C*H$_2$*), 2.80–2.88 (4H, m, 2 × ring C*H$_2$*), 2.94–3.03 (4H, m, 2 × ring C*H$_2$*), 3.35 (2H, s, arm C*H$_2$*), 3.41 (4H, s, 2 × arm C*H$_2$*), 8.80 (2H, br s, NH_2^+); LRMS *m/z* (ESI$^+$) 495 ([M-tBu-Br]$^+$, 61%), 515 ([M-Br]$^+$, 100%), 1111 ([2M-Br]$^+$, 94%). These data are in good agreement with the reported values.[19-23]

4'-Nitrobenzyl 2-bromoacetate, 6

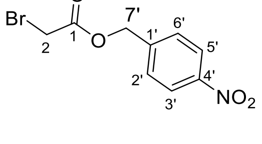

To a suspension of 4-nitrobenzyl alcohol (2.00 g, 13.1 mmol, 1.0 eq.) and $NaHCO_3$ (3.34 g, 39.8 mmol, 3.0 eq.) in MeCN (20 mL), bromoacetyl bromide (1.50 mL, 17.2 mmol, 1.3 eq.) dissolved in MeCN (6.0 mL) was added dropwise at 0 °C. The resulting suspension was warmed to 45 °C and stirred for a

further 18 h, cooled to room temperature, filtered through Celite®, washed with CH_2Cl_2 and the filtrate was concentrated *in vacuo*. The resulting material was adsorbed onto Celite® and purified by silica column chromatography, eluting with EtOAc and petroleum ether (gradient, 0–15% EtOAc), and then was crystallised from Et_2O/hexane to yield compound **6** as a colourless solid (1.99 g, 55%): R_f 0.29 (petroleum ether : EtOAc 4:1); mp 43–45 °C (hexane); ν_{max} (solid) cm^{-1}: 1753 (C=O, s), 1517 (N-O, s), 1151 (C-O, s); ^{1}H NMR (400 MHz, $CDCl_3$): 3.91 (2H, s, C^2H_2), 5.30 (2H, s, $C^{7'}H_2$), 7.51–7.57 (2H, m, $C^{2'}H/C^{6'}H$), 8.20–8.28 (2H, m, $C^{3'}H/C^{5'}H$); ^{13}C NMR (151 MHz, $CDCl_3$): 25.4 (C^2), 66.4 ($C^{7'}$), 124.1 ($C^{3'}/C^{5'}$), 128.7 ($C^{2'}/C^{6'}$), 142.2 ($C^{1'}$), 148.1 ($C^{4'}$), 167.0 (C^1); LRMS not detected by ESI/EI/APCI. These data are in good agreement with the reported values.[18]

Tri-*tert*-butyl 2,2',2''-(10-(2-((4-nitrobenzyl)oxy)-2-oxoethyl)-1,4,7,10-tetraazacyclododecane-1,4,7-triyl)triacetate, 8

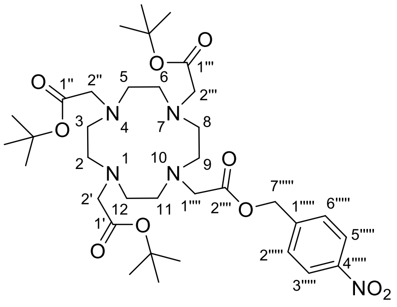

5 (1.35 g, 2.27 mmol, 1.0 eq.) and $NaHCO_3$ (584 mg, 6.95 mmol, 3.0 eq.) was added to a solution of **6** (812 mg, 2.96 mmol, 1.3 eq.) in dry MeCN (20.0 mL) under an argon atmosphere. The resulting suspension was stirred at 80 °C for 39 h, cooled to room temperature, filtered through Celite®, washed with CH_2Cl_2 and the filtrate was concentrated *in vacuo*. The resulting material was adsorbed onto Celite® and purified by silica column chromatography, eluting with EtOH and $CHCl_3$ (gradient, 0–15% EtOH), to yield **8** as an off-white foam (1.48 mg, 92%): R_f 0.36 ($CHCl_3$: EtOH 8:2); ν_{max} (solid) cm^{-1}: 1726 (C=O, s), 1627 (C=C, m), 1522 (N-O, s), 1162 (C-O, s); ^{1}H NMR (400 MHz, $CDCl_3$): 1.34 (27H, s, 3 × $C(CH_3)_3$), 1.79–3.52 (24H, m, 4 × $COCH_2$, 8 × ring CH_2), 5.14 (2H, s, $C^{7'''''}H_2$), 7.39–7.47 (2H, m, $C^{2'''''}H/C^{6'''''}H$), 8.02–8.15 (2H, m, $C^{3'''''}H/C^{5'''''}H$); ^{13}C NMR (151 MHz, $CDCl_3$): 27.7 (3 × $(CH_3)_3$), 46.3-53.5 (m, 8 × ring CH_2), 54.8 (arm CH_2), 55.4 (arm CH_2), 55.6 (2 × arm CH_2), 65.2 ($C^{7'''''}$), 81.9 (3 × $C(CH_3)_3$), 123.6 ($C^{3'''''}/C^{5'''''}$), 128.7 ($C^{2'''''}/C^{6'''''}$), 142.3 ($C^{1'''''}$), 147.6 ($C^{4'''''}$), 172.7 ($C^{1'}/C^{1''}/C^{1'''}$), 172.9 (2 × $C^{1'}/C^{1''}/C^{1'''}$), 173.4

($C^{2''''}$); HRMS *m/z* (ESI^+) [Found: 708.4188, $C_{35}H_{57}N_5O_{10}$ requires $[M+H]^+$ 708.4178]; LRMS *m/z* (ESI^+) 708 ($[M+H]^+$, 90%), 730 ($[M+Na]^+$, 94%); LCMS (method 1) R_t 3.9 min, 89.4%. These data are generally in good agreement with the reported values.[18]

2,2',2"-(10-(2-((4-Nitrobenzyl)oxy)-2-oxoethyl)-1,4,7,10-tetraazacyclododecane-1,4,7-triyl)triacetic acid, 10

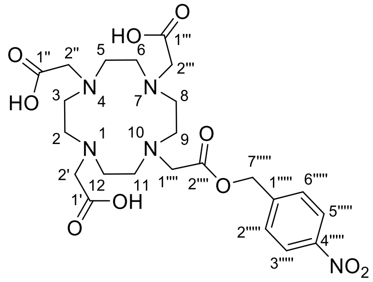

8 (1.71 g, 2.41 mmol, 1 eq.) was dissolved in CH_2Cl_2 (70.0 mL) and TFA (70.0 mL, 915 mmol, 380 eq.) was added and the resulting solution was stirred at rt for 43 h. The resulting solution was concentrated *in vacuo* and washed with CH_2Cl_2 (70 mL) and concentrated *in vacuo* and this procedure was repeated two times. The resulting residue was dissolved in MeOH (70 mL) and concentrated *in vacuo* and this procedure was repeated three times. The resulting residue was dissolved in a minimum amount of MeOH, diethyl ether was added until a white precipitate formed. The precipitate was isolated by centrifugation (3750 rpm, 5 minutes, 4 C). The precipitation procedure was repeated twice and the resulting solid was collected in MeOH then concentrated *in vacuo* to give **10** as an off-white solid (1.01 g, 78%): mp 205 °C (decomposition, MeOH); ν_{max} (solid) cm^{-1}: 3448 (O-H, br, w), 1727 (C=O, m), 1676 (C=O, m), 1636 (C=C, m), 1522 (N-O, m), 1349 (O-H, m) 1189 (C-O, s); ^1H NMR (400 MHz, D_2O): 2.92–3.27 (8H, m, 4 × ring CH_2), 3.39–3.60 (8H, m, 4 × ring CH_2), 3.62–4.00 (8H, m, 4 × $COCH_2$), 5.35 (2H, s, $C^{7'''''}H_2$), 7.62–7.68 (2H, m, $C^{2'''''}H/C^{6'''''}H$), 8.24–8.30 (2H, m, $C^{3'''''}H/C^{5'''''}H$); ^{13}C NMR (151 MHz, D_2O): 48.7 (ring CH_2), 51.9 (ring CH_2, arm CH_2), 53.7 (arm CH_2), 56.6 (arm CH_2), 66.6 ($C^{7'''''}$), 124.4 ($C^{3'''''}/C^{5'''''}$), 129.5 ($C^{2'''''}/C^{6'''''}$), 143.8 ($C^{1'''''}$), 148.0 ($C^{4'''''}$), 170.5 ($C^{1'}/C^{1''}/C^{1'''}$), 172.5 ($C^{2''''}$), 174.5 ($C^{1'}/C^{1''}/C^{1'''}$); HRMS *m/z* (ESI^+) [Found: 540.2314, $C_{23}H_{33}N_5O_{10}$ requires $[M+H]^+$ 540.2300]; LRMS *m/z* (ESI^+) 540 ($[M+H]^+$, 97%), 562 ($[M+Na]^+$, 44%); LCMS (method 2) R_t 2.4 min, 98.6%. These data are generally in good agreement with the reported values.[18]

General lanthanide complexation procedure

The corresponding lanthanide triflate (1.00-1.12 eq.) was added to a solution of the triacid (1.0 eq.) in MES buffer (1M, pH 6.0, compound at ~65 mM) and the resulting solution was stirred at room temperature for 1-1.5 h, until the reaction was deemed complete by LCMS analysis (method 2). The reaction mixture was diluted by water (same as or larger volume than MES buffer), filtered (nylon syringe filter, 0.45 μM, 13 mm) and purified by semi-preparative HPLC (method 1 for nitrobenzyl complexes, method 2 for nitroimidazole complexes and method 3 for benzyl complexes). The complexes were recovered as colourless solids from water/acetonitrile mixtures by lyophilisation.

Europium nitrobenzyl complex (EuNB), 1a

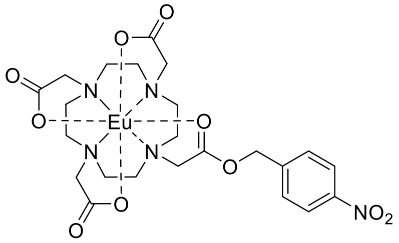

The reaction was carried out following the general lanthanide complexation procedure, using **10** (76.6 mg, 142 μmol, 1.0 eq.) and europium triflate (90.4 mg, 151 μmol, 1.06 eq.) in MES buffer (2.70 mL). The reaction was left at room temperature for 1 hour, purified by semi-preparative HPLC method 1 and lyophilised for 2 days to yield compound **1a** as a colourless solid (65.4 mg, 66%): ^{1}H NMR (400 MHz, D_2O):* −19.4, −17.1, −16.0, −14.5, −13.5, −12.7, −10.3, −9.4, −8.9, −8.6, −8.0, −6.8, −6.2, −6.1, −3.9, −1.1, −0.5, −0.3, 0.2, 3.3, 7.0, 7.8, 8.3, 9.2, 10.6, 24.5, 25.0, 25.8, 32.7, 33.8; HRMS *m/z* (ESI^+) [Found: 690.1275, $C_{23}H_{30}N_5O_{10}Eu$ requires $[M+H]^+$ 690.1278]; LRMS *m/z* (ESI^+) 712 ($[M+Na]^+$, 10%); LCMS (method 2) R_t 2.5 min, 95.4%; Analytical HPLC (method A) R_t 9.1 min, 96.3%. *^{1}H NMR contains ~2% EuDOTA so total purity ~93%, all peaks reported including EuDOTA.

Terbium nitrobenzyl complex (TbNB), 1b

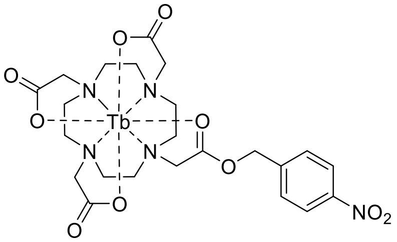

The reaction was carried out following the general lanthanide complexation procedure, using **10** (76.0 mg, 141 μmol, 1.0 eq.) and terbium triflate (90.0 mg, 149 μmol, 1.06 eq.) in MES buffer (2.15 mL). The reaction was left at room temperature for 1 hour, purified by semi-preparative HPLC method 1 and lyophilised for 2 days to give compound **1b** as a colourless solid (42.6 mg, 47%): ^{1}H NMR (400 MHz, D_2O):* −416.7, −398.2, −336.0, −326.9, −305.1, −186.6, −153.7, −118.6, −99.3, −96.8, −95.7, −92.3, −69.5, −65.1, −43.6, −35.7, −32.4, −18.3, −15.8, −14.0, −2.0, −0.45, 1.0, 1.4, 14.5, 82.8, 136.1, 148.0, 159.6, 214.1, 228.4, 293.7; HRMS *m/z* (ESI$^+$) [Found: 696.1319, $C_{23}H_{30}N_5O_{10}Tb$ requires $[M+H]^+$ 696.1319]; LRMS *m/z* (ESI$^+$) 696 ($[M+H]^+$, 37%), 718 ($[M+Na]^+$, 100%), 1413 ($[2M+Na]^+$, 59%); LCMS (method 2) R_t 2.8 min, 95.1%; Analytical HPLC (method A) R_t 9.0 min, 94.5%.

*^{1}H NMR contains 5% TbDOTA, therefore total purity ~90%, all peaks reported including TbDOTA.

Gadolinium nitrobenzyl complex (GdNB), 1c

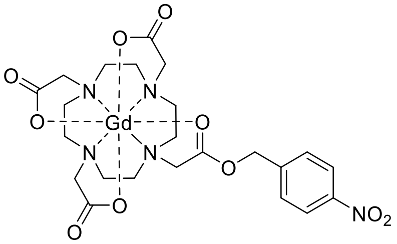

The reaction was carried out following the general lanthanide complexation procedure, using **10** (97.9 mg, 181 μmol, 1.0 eq.) and gadolinium triflate (116 mg, 192 μmol, 1.06 eq.) in MES buffer (2.80 mL). The reaction was left at room temperature for 1 hour, purified by semi-preparative HPLC method 1 and lyophilised for 2 days to give compound **1c** as a colourless solid (54.1 mg, 43%): HRMS *m/z* (ESI$^+$) [Found: 695.1310, $C_{23}H_{30}N_5O_{10}Gd$ requires $[M+H]^+$ 695.1307]; LRMS *m/z* (ESI$^+$) 608 (100%),

717 ([M+Na]$^+$, 4%); LCMS (method 2) R_t 2.15 min, 97.2%; Analytical HPLC (method A) R_t 9.1 min, 96.1%.

Ethyl 2-amino-1-methyl-1*H*-imidazole-5-carboxylate, S1

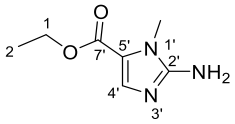

To a suspension of sarcosine ethyl ester hydrochloride (4.00 g, 26.0 mmol, 1.0 eq.) and ethyl formate (14.6 mL, 182 mmol, 7.0 eq.) in dry THF (45 mL) and EtOH (6.5 mL), NaH (60% dispersion in mineral oil, 4.19 g, 105 mmol, 4.0 eq.) was added portion-wise at 0 °C. The reaction mixture was warmed to room temperature and stirred for 3 h. The resulting yellow suspension was concentrated *in vacuo* and the resulting solid was triturated with hexane (2 × 75 mL), the hexane decanted and the resulting solid concentrated *in vacuo*. The light-yellow solid was suspended in EtOH (80 mL) and concentrated aqueous HCl (37%, 16 mL) was added dropwise, then the suspension was heated under reflux for 2 h. The reaction mixture was filtered whilst hot, washed with hot EtOH (2 × 30 mL) and the filtrate was concentrated *in vacuo* to leave an aqueous solution, which was diluted with EtOH (70 mL) and water (30 mL). The pH of the resulting green solution was adjusted to 3-4 using aqueous NaOH (2 M). Cyanamide (2.20 g, 52.2 mmol, 2.0 eq.) was added and the resulting solution was heated under reflux for 1.5 h, then cooled to room temperature and concentrated *in vacuo* until approximately 1/8th of the original volume. The pH was adjusted to 8-9 using solid K_2CO_3, resulting in the formation of a precipitate. The solid was removed by filtration, washed with saturated aqueous $NaHCO_3$ (2 × 20 mL) and water (20 mL) and dried *in vacuo* to yield compound **S1** as a yellow solid (2.18 g, 48%): R_f 0.34 (MeOH : CH_2Cl_2 1:9); mp 150–152 °C (H_2O) [lit.[2] 130–133 °C (H_2O)]; ^{1}H NMR (400 MHz, $CDCl_3$): 1.33 (3H, t, *J* = 7.1, C^2H_3), 3.68 (3H, s, $N^{1'}CH_3$), 4.27 (2H, q, *J* = 7.1, C^1H_2), 7.43 (1H, s, $C^{4'}H$); LRMS *m/z* (ESI$^+$) 170 ([M+H]$^+$, 100%). These data are generally in good agreement with the reported values.[2, 24]

Ethyl 1-methyl-2-nitro-1*H*-imidazole-5-carboxylate, S2

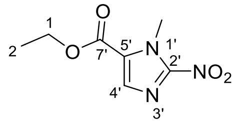

A solution of **S1** (2.89 g, 17.1 mmol, 1.0 eq.) in acetic acid (58.0 mL) was added dropwise at 0 °C to a solution of $NaNO_2$ (11.8 g, 171 mmol, 10 eq.) in water (27.0 mL) and the resulting solution was stirred at 0 °C for 1 h, then warmed to room temperature and stirred for 3 h. The resulting mixture was extracted with CH_2Cl_2 (3 × 100 mL) and the organic components were washed with saturated aqueous sodium sulfite (200 mL) and brine (200 mL), dried over $MgSO_4$, filtered and concentrated *in vacuo*. The resulting residue was dissolved in CH_2Cl_2 and purified by silica column chromatography, eluting with CH_2Cl_2, to yield compound **S2** as a yellow solid (1.96 g, 58%): R_f 0.86 (MeOH : CH_2Cl_2 2:98); mp 50–52 °C (CH_2Cl_2) [lit.[2] 56–58 °C (CH_2Cl_2), lit.[25] 65–66 °C]; ^{1}H NMR (400 MHz, $CDCl_3$): 1.40 (3H, t, J = 7.1, C^2H_3), 4.34 (3H, s, $N^{1'}CH_3$), 4.40 (2H, q, J = 7.1, C^1H_2), 7.74 (1H, s, C^4H); LRMS *m/z* (ESI$^+$) 200 ($[M+H]^+$, 100%), 222 ($[M+Na]^+$, 89%). These data are generally in good agreement with the reported values.[2, 25]

(1-Methyl-2-nitro-1*H*-imidazol-5-yl)methanol, S3

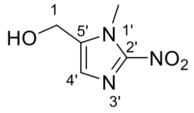

A suspension of $NaBH_4$ (335 mg, 8.50 mmol, 3.0 eq.) in dry EtOH (9.3 mL) was added dropwise at 0 °C to a solution of compound **S2** (570 mg, 2.90 mmol, 1.0 eq.) in dry THF (14.7 mL) and MeOH (0.97 mL). The resulting solution was stirred at 0 °C for 3 h, then further $NaBH_4$ (335 mg, 8.0 mmol, 3.0 eq.) in dry EtOH (10.0 mL) was added dropwise, as the reaction was not complete by TLC analysis. After a further 3 h, MeOH (15.0 mL) was added and the resulting solution was stirred at 0 °C for a further 30 min, then acidified to pH 7 using 1M aqueous HCl and concentrated *in vacuo* to leave an aqueous solution. The aqueous solution was extracted with EtOAc (3 × 40 mL) and the organic components were washed with saturated aqueous $NaHCO_3$ (120 mL), dried over Na_2SO_4, filtered and concentrated *in vacuo*. The resulting residue was adsorbed onto Celite® and purified by silica column chromatography, eluting with MeOH and CH_2Cl_2 (gradient, 2–3% MeOH), to yield compound **S3** as a pale-yellow solid (239 mg, 53%):

R_f 0.32 (MeOH : CH_2Cl_2 2:98); mp 120–122 °C (MeOH) [lit.[2] 141–143 °C (EtOAc), lit.[25] 142–144 °C]; ^{1}H NMR (400 MHz, DMSO-d$_6$): 3.91 (3H, s, $N^{1'}CH_3$), 4.54 (2H, s, C^1H_2), 5.49 (1H, s, O*H*), 7.11 (1H, d, *J* = 0.6, $C^{4'}H$); LRMS *m/z* (ESI$^+$) 158 ([M+H]$^+$, 100%). These data are generally in good agreement with the reported values.[2, 25]

(1-Methyl-2-nitro-1*H*-imidazol-5-yl)methyl 2-bromoacetate, 7

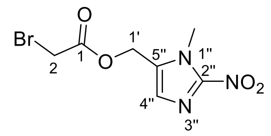

To a solution of compound **S3** (97.3 mg, 0.619 mmol, 1 eq.) in CH_2Cl_2 (4.13 mL) and DMF (4.13 mL) at 0 °C, 2,6-di-tertbutylpyridine (0.41 mL, 1.90 mmol, 3.1 eq.) was added and the resulting solution was stirred at 0 °C for 10 minutes. Bromoacetyl bromide (0.07 mL, 0.804 mmol, 1.3 eq.) was added and the resulting solution was warmed to room temperature and stirred for a further 16 h then the solvent was reduced *in vacuo*. The resulting mixture was diluted with Et_2O (90 mL) and the organic components were washed with 0.5 M LiCl solution (3 × 30 mL), dried over Na_2SO_4, filtered and concentrated *in vacuo*. The resulting residue was adsorbed onto Celite® and purified by silica column chromatography, eluting with EtOAc and petroleum ether (isocratic, 40% EtOAc), to yield compound **7** as a yellow solid (138 mg, 80%): R_f 0.21 (EtOAc : petroleum ether 4:6); mp 63–65 °C (EtOAc); ν_{max} (thin film) cm^{-1}: 1746 (C=O, s), 1541 (N-O, m), 1282 (C-O, s); ^{1}H NMR (600 MHz, CD_3CN): 3.93 (3H, s, $N^{1''}CH_3$), 3.97 (2H, s, C^2H_2), 5.23 (2H, s, $C^{1'}H_2$), 7.19 (1H, s, $C^{4''}H$); ^{13}C NMR (151 MHz, MeCN-d$_3$): 27.1 ($N^{1''}C$), 35.2 (C^2), 57.6 ($C^{1'}$), 130.1 ($C^{4''}$), 133.1 ($C^{5''}$), 147.7 ($C^{2''}$), 167.7 (C^1); HRMS *m/z* (ESI$^+$) [Found: 277.9775, $C_7H_8BrN_3O_4$ requires [M+H]$^+$ 277.9771]; LRMS *m/z* (ESI$^+$) 278 ([M+H]$^+$, ^{79}Br, 14%), 280 ([M+H]$^+$, ^{81}Br, 12%), 577 ([2M+Na]$^+$, ^{79}Br^{79}Br, 38%), 579 ([2M+Na]$^+$, ^{79}Br^{81}Br, 66%), 581 ([2M+Na]$^+$, ^{81}Br^{81}Br, 39%); LCMS (method 1) R_t 3.6 min, 91.5%.

Tri-*tert*-butyl 2,2',2''-(10-(2-((1-methyl-2-nitro-1H-imidazol-5-yl)methoxy)-2-oxoethyl)-1,4,7,10-tetraazacyclododecane-1,4,7-triyl)triacetate, 9

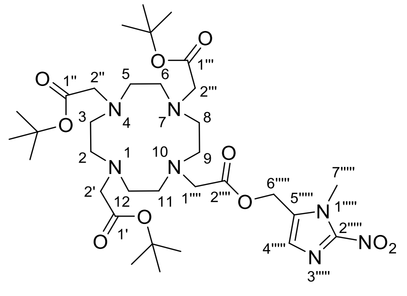

5 (516 mg, 0.866 mmol, 1 eq.) and $NaHCO_3$ (219 mg, 2.61 mmol, 3.0 eq.) was added to a solution of **7** (314 mg, 1.13 mmol, 1.3 eq.) in MeCN (14.0 mL). The resulting suspension was stirred at 80 °C for 42 h, cooled to room temperature, filtered through Celite®, washed with CH_2Cl_2 and the filtrate was concentrated *in vacuo*. The resulting material was adsorbed onto Celite® and purified by silica column chromatography, eluting with EtOH and chloroform (gradient, 0–20% EtOH), resulting in an orange foam **9** (510 mg, 83%) which was stored at -20 °C: R_f 0.33 (EtOH : chloroform 2:8); v_{max} (solid) cm^{-1}: 1725 (C=O, s), 1536 (N-O, w), 1227 (C-O, s); ^{1}H NMR (400 MHz, $CDCl_3$): 1.24–1.46 (27H, m, 3 × C(CH_3)$_3$), 1.54-2.78 (16H, m, 8 × ring CH_2), 2.98-3.51 (8H, m, 4 × COCH_2), 4.07 (3H, s, $C^{7'''''}H_3$), 5.31 (2H, s, $C^{6'''''}H_2$), 7.06 (1H, s, $C^{4'''''}H$); ^{1}H NMR (600 MHz, CD_3CN): 1.29–1.54 (27H, s, 3 × C(CH_3)$_3$), 1.99–3.49 (24H, m, 4 × COCH_2, 8 × ring CH_2), 3.91–3.97 (3H, m, $C^{7'''''}H_3$), 5.15–5.30 (2H, m, $C^{6'''''}H_2$), 7.12–7.19 (1H, s, $C^{4'''''}H$); ^{13}C NMR (151 MHz, CD_3CN): 28.2 (3 × (CH$_3$)$_3$), 35.1 ($C^{7'''''}$), 47.9–50.2 (m, ring CH$_2$), 52.1–54.5 (m, ring CH$_2$), 55.5 (arm CH$_2$), 56.3 (arm CH$_2$), 56.5 (arm CH$_2$), 62.0 (arm CH$_2$), 82.8 (3 × C(CH$_3$)$_3$), 130.3 ($C^{4'''''}$), 133.4 ($C^{5'''''}$), 147.5 ($C^{2'''''}$), 167.9 ($C^{2''''}$), 174.1 ($C^{1'}/C^{1''}/C^{1'''}$), 174.3 ($C^{1'}/C^{1''}/C^{1'''}$), 174.4 ($C^{1'}/C^{1''}/C^{1'''}$); HRMS *m/z* (ESI$^+$) [Found: 712.4230, $C_{33}H_{57}N_7O_{10}$ requires $[M+H]^+$ 712.4240]; LRMS *m/z* (ESI$^+$) 712 ($[M+H]^+$, 27%), 734 ($[M+Na]^+$, 100%); LCMS (method 1) R_t 3.6 min, 98.3%.

2,2',2''-(10-(2-((1-Methyl-2-nitro-1*H*-imidazol-5-yl)methoxy)-2-oxoethyl)-1,4,7,10-tetraazacyclododecane-1,4,7-triyl)triacetic acid, 11

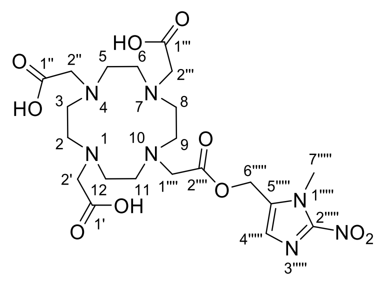

9 (129 mg, 0.181 mmol, 1 eq.) was dissolved in CH_2Cl_2 (5.2 mL) and TFA (5.2 mL, 68.0 mmol, 376 eq.) was added and the resulting solution was stirred at rt for 18 h. The resulting solution was concentrated *in vacuo* and washed with CH_2Cl_2 (10 mL) and concentrated *in vacuo* and this procedure was repeated two times. The resulting residue was dissolved in MeOH (10 mL) and concentrated *in vacuo* and this procedure was repeated three times. The resulting residue was dissolved in a minimum amount of MeOH, diethyl ether was added until a white precipitate formed. The precipitate was isolated by centrifugation (3750 rpm, 5 minutes, 4 °C). The precipitation procedure was repeated twice and the resulting solid was collected in MeOH then concentrated *in vacuo* to give **11** as an off-white solid (79.5 mg, 81%): ν_{max} (solid) cm^{-1}: 3448 (O-H, br, w), 1727 (C=O, m), 1676 (C=O, m), 1636 (C=C, m), 1522 (N-O, m), 1350 (O-H, m), 1189 (C-O, s); ^{1}H NMR (600 MHz, D_2O): 2.96–3.30 (8H, m, 4 × ring CH_2), 3.40–3.64 (8H, m, 4 × ring CH_2), 3.64–3.84 (4H, m, 2 × arm CH_2), 3.94–4.11 (7H, m, 2 × arm CH_2, $C^{7'''''}H_3$), 5.32–5.42 (2H, m, $C^{6'''''}H_2$), 7.25–7.35 (1H, m, $C^{4'''''}H$); ^{13}C NMR (151 MHz, D_2O): 34.9 ($C^{7'''''}$), 48.0–49.2 (m, ring CH_2), 51.2–52.3 (m, ring CH_2), 53.0–54.1 (m, arm CH_2), 55.4–56.2 (m, arm CH_2), 56.3–56.8 ($C^{6'''''}$), 129.8 ($C^{4'''''}$), 133.6 ($C^{5'''''}$), 146.4 ($C^{2'''''}$), 170.0 ($C^{1'}/C^{1''}/C^{1'''}$), 172.2 ($C^{2''''}$), 174.5 ($C^{1'}/C^{1''}/C^{1'''}$); HRMS *m/z* (ESI^+) [Found: 544.2347, $C_{21}H_{34}N_7O_{10}$ requires $[M+H]^+$ 544.2362; LRMS *m/z* (ESI^+) 544 ($[M+H]^+$, 100%), 566 ($[M+Na]^+$, 31%); LCMS (method 2) R_t 0.6 min, >99%.

Europium nitroimidazole complex (EuNI), 2a

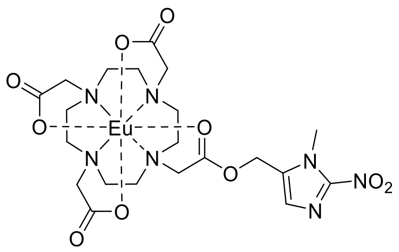

The reaction was carried out following the general lanthanide complexation procedure, using **11** (75.0 mg, 138 µmol, 1.0 eq.) and europium triflate (86.8 mg, 145 µmol, 1.07 eq.) in MES buffer (2.12 mL). The reaction was left at room temperature for 1 hour, purified by semi-preparative HPLC method 2 and lyophilised for 2 days to yield compound **2a** as a colourless solid (36.9 mg, 39%): ^{1}H NMR (400 MHz, D_2O)*: −19.5, −17.2, −16.5, −16.0, −14.6, −12.3, −10.6, −9.5, −9.0, −8.4, −8.0, −7.2, −6.6,

−6.3, −5.4, −3.6, −1.0, −0.7, 0.4, 5.7, 7.2, 8.5, 9.3, 10.5, 13.4, 23.8, 25.3, 32.5, 33.8; LRMS *m/z* (ESI^+) 715 ($[M+Na]^+$, 9%); HRMS *m/z* (ESI^+) [Found: 694.1333, $C_{21}H_{30}N_7O_{10}Eu$ requires $[M+H]^+$ 694.1339]; LCMS (method 2) 1.1 min, 94.5%; Analytical HPLC (method A): R_t 3.0 min, 95.1%.

*^{1}H NMR contains 6% EuDOTA, therefore total purity ~89%, all peaks reported including EuDOTA.

Terbium nitroimidazole complex (TbNI), 2b

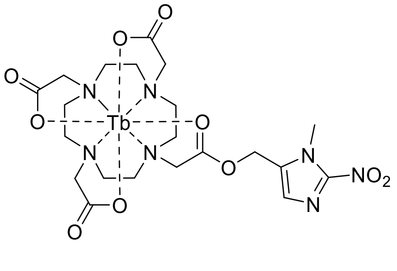

The reaction was carried out following the general lanthanide complexation procedure, using **11** (75.5 mg, 139 µmol, 1.0 eq.) and terbium triflate (88.9 mg, 147 µmol, 1.08 eq.) in MES buffer (2.12 mL). The reaction was left at room temperature for 1 hour, purified by semi-preparative HPLC method 2 and lyophilised for 2 days to yield compound **2b** as a colourless solid (38.6 mg, 40%): ^{1}H NMR (400 MHz, D_2O)*: −417.0, −394.7, −326.7, −322.7, −295.3, −217.9, −194.0, −177.6, −158.6, −117.2, −98.1, −94.7, −91.5, −71.2, −65.8, −62.6, −43.3, −32.2, −22.2, −19.4, −18.1, −7.9, −5.2, 78.3, 82.5, 95.3, 136.0, 152.7, 169.5, 206.4, 215.4, 220.8, 228.3, 257.5, 303.5; HRMS *m/z* (ESI^+) [Found: 700.1357, $C_{21}H_{30}N_7O_{10}Tb$ requires $[M+H]^+$ 700.1380]; LCMS (method 2) R_t 0.7 min, 98.5%; Analytical HPLC (method A) R_t 2.9 min, 93.8%.

*^{1}H NMR contains 7% TbDOTA (total purity ~89%), all peaks reported including TbDOTA.

Gadolinium nitroimidazole complex (GdNI), 2c

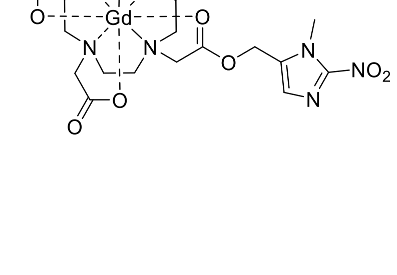

The reaction was carried out following the general lanthanide complexation procedure, using **11** (49.3 mg, 90.7 µmol, 1.0 eq.) and gadolinium triflate (60.0 mg, 99.3 µmol, 1.07 eq.) in MES buffer (1.40 mL). The reaction was left at room temperature for 1 hour, purified by semi-preparative HPLC method 2 and lyophilised to yield compound **2c** as a colourless solid (35.4 mg, 56%): HRMS *m/z* (ESI$^+$) [Found: 721.1167, $C_{21}H_{30}N_7O_{10}Gd$ requires $[M+Na]^+$ 721.1188]; LRMS *m/z* (ESI$^+$) 721 ($[M+Na]^+$, 3%); LCMS (method 2) R_t 1.1 min, 95.3%; Analytical HPLC (method A) R_t 3.0 min, 95.6%.

EuDOTA, 3a

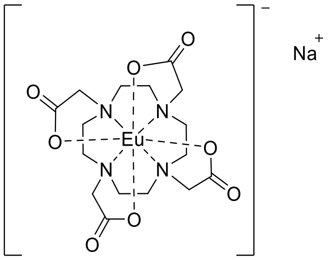

Europium triflate (119 mg, 0.199 mmol, 1.2 eq.) was added to a suspension of DOTA (67.4 mg, 0.167 mmol, 1 eq.) in EtOH (0.95 mL) and water (0.95 mL), and the resulting suspension was heated to 40 °C for 17 h. 1M NaOH solution was added portion-wise (1 eq. every 30 minutes, total 0.58 mL, 3.5 eq.) until pH 7 was reached. The resulting suspension was cooled to room temperature, split into two 1.5 mL Eppendorf tubes, centrifuged for 10 minutes and the supernatants were filtered (through a Nylon syringe filter), combined and concentrated *in vacuo* to give a colourless solid. The crude residue was redissolved in a minimal amount of water and purified by dialysis (2 weeks, procedure as described in the general experimental), then concentrated *in vacuo* to give compound **3a** as a colourless solid (42.2 mg, 46%): ^1H NMR (400 MHz, D_2O): −16.0, −14.6, −8.0, −6.7, −1.1, 33.8 (only SAP isomer reported); HRMS *m/z* (ESI$^+$) [Found: 577.0769, $C_{16}H_{24}EuN_4NaO_8$ requires $[M+H]^+$ 577.0777]; LRMS *m/z* (ESI$^+$) 577 ($[M+H]^+$, 12%), 599 ($[M+Na]^+$, 100%). These data are in good agreement with the previously reported values.[26]

TbDOTA, 3b

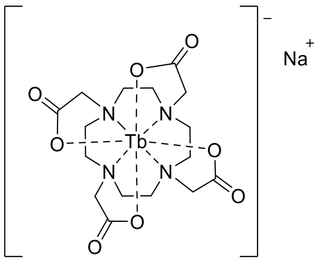

Terbium triflate (133 mg, 0.219 mmol, 1.1 eq.) was added to a solution of DOTA (80.0 mg, 0.198 mmol, 1.0 eq.) in water (4.0 mL), and the resulting solution was heated to 40 °C for 19 h. 1M NaOH solution was added portion-wise (1 eq. every 30 minutes, total 0.50 mL, 2.5 eq.) until pH 7 was reached. The resulting suspension was cooled to room temperature, filtered (through a Nylon syringe filter) and concentrated *in vacuo* to give a colourless solid. The crude residue was redissolved in a minimal amount of water and purified by dialysis (2 weeks, procedure as described in the general experimental), then concentrated *in vacuo* to give compound **3b** as a colourless solid (27.6 mg, 25%): ^{1}H NMR (400 MHz, D_2O): −400.3 (SAP, axial ring CH), −240.0 (TSAP), −99.0 (SAP), −95.2 (SAP), −71.5 (TSAP), −69.8 (TSAP), −5.9 (TSAP), 63.6 (TSAP), 84.4 (SAP), 138.4 (SAP), 177.9 (TSAP), 261.9 (SAP, arm CH); HRMS *m/z* (ESI$^+$) [Found: 583.0821, $C_{16}H_{24}TbN_4NaO_8$ requires [M+H]$^+$ 583.0818]; LRMS *m/z* (ESI$^+$) 583 ([M+H]$^+$, 21%), 605 ([M+Na]$^+$, 100%). These data are in good agreement with the previously reported values.[27]

Tri-*tert*-butyl 2,2',2''-(10-(2-(benzyloxy)-2-oxoethyl)-1,4,7,10-tetraazacyclododecane-1,4,7-triyl)triacetate, S4

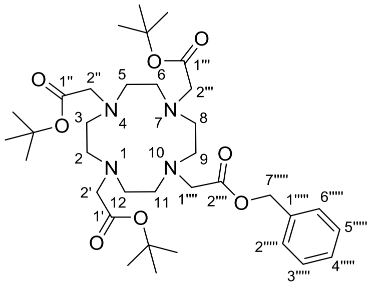

Benzyl 2-bromoacetate (0.40 mL, 2.50 mmol, 1.5 eq.) was added dropwise at 0 °C to a solution of **5** (1.00 g, 1.68 mmol, 1.0 eq.) and K_2CO_3 (1.09 g, 7.89 mmol, 4.7 eq.) in MeCN (20.0 mL). The resulting suspension was stirred at rt for 3 h, filtered, washed with MeCN and the filtrate was concentrated *in*

vacuo. The resulting material was dissolved in CH_2Cl_2 and purified by silica column chromatography, eluting with MeOH and CH_2Cl_2 (gradient, 0–10% MeOH), resulting in a yellow oil **S4** (1.02 g, 91%) which was stored at -20 °C: R_f 0.18 (MeOH : CH_2Cl_2 5:95); ν_{max} (solid) cm^{-1}: 1727 (C=O, s), 1627 (C=C, w), 1229 (C-O, m), 1162 (C-O, m); ^{1}H NMR (400 MHz, $CDCl_3$): 1.45 (27H, s, 3 × $C(CH_3)_3$), 1.65–3.44 (24H, m, 4 × $COCH_2$, 8 × ring CH_2), 5.13 (2H, s, $C^{7'''''}H_2$), 7.28–7.39 (5H, m, $C1'''''H/C^{2'''''}H/C^{3'''''}H/C^{4'''''}H/C^{5'''''}H$); ^{13}C NMR (151 MHz, $CDCl_3$): 27.9–28.5 (m, 3 × $(CH_3)_3$), 50.6–51.5 (ring CH_2), 51.5–53.5 (ring CH_2), 55.2 (arm CH_2), 55.6–56.4 (arm CH_2), 67.0 ($C^{7'''''}$), 81.9–82.0 (m, 3 × $C(CH_3)_3$), 128.3–128.9 ($C^{2''''}/C^{3'''''}/C^{4'''''}/C^{5''''''}/C^{6'''''}$), 135.4 ($C^{1'''''}$), 173.2 ($C^{1'}/C^{1''}/C^{1'''}$), 173.3 ($C^{1'}/C^{1''}/C^{1'''}$), 173.8 ($C^{2''''}$); HRMS *m/z* ($ESI^+$) [Found: 685.4159, $C_{35}H_{58}N_4O_8$ requires $[M+Na]^+$ 685.4147]; LRMS *m/z* (ESI^+) 663 ($[M+H]^+$, 99%), 685 ($[M+Na]^+$, 100%); Analytical HPLC (method B) R_t 7.8 min, 95.1%. These data are in good agreement with the available literature values.[28-31]

2,2',2''-(10-(2-(benzyloxy)-2-oxoethyl)-1,4,7,10-tetraazacyclododecane-1,4,7-triyl)triacetic acid, S5

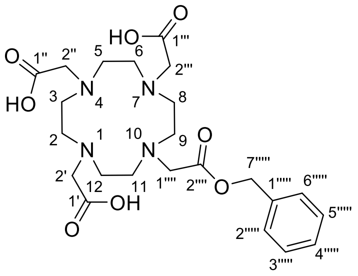

S4 (1.08 g, 1.63 mmol, 1 eq.) was dissolved in CH_2Cl_2 (50.0 mL) and TFA (50.0 mL, 653 mmol, 401 eq.) was added and the resulting solution was stirred at rt for 18 h. The resulting solution was concentrated *in vacuo* and washed with CH_2Cl_2 (50 mL) and concentrated *in vacuo* and this procedure was repeated two times. The resulting residue was dissolved in MeOH (50 mL) and concentrated *in vacuo* and this procedure was repeated three times. The resulting residue was dissolved in a minimum amount of MeOH and added into diethyl ether until a white precipitate formed. The precipitation procedure was repeated twice and the resulting solid was collected in MeOH then concentrated *in vacuo* to give **S5** as an off-white solid (799 mg, 99%): ν_{max} (solid) cm^{-1}: 1779 (C=O, m), 1713 (C=O, m), 1687 (C=C, s), 1386 (O-H, m), 1355 (O-H, m), 1200 (C-O, s); ^{1}H NMR (400 MHz, D_2O): 2.92–3.24 (8H, m, 4 × ring CH_2),

3.41–3.54 (8H, m, 4 × ring CH_2), 3.63–4.02 (8H, m, 4 × COCH_2), 5.25 (2H, s, $C^{7'''''}H_2$), 7.39-7.53 (5H, m, $C^{2'''''}H$/$C^{3'''''}H$/$C^{4'''''}H$/$C^{5'''''}H$/$C^{6'''''}H$); ^{13}C NMR (151 MHz, D_2O): 48.8 (ring CH_2), 51.8 (ring CH_2), 53.8 (arm CH_2), 56.5 (arm CH_2), 68.0 ($C^{7'''''}$), 129.4 ($C^{2'''''}$/$C^{3'''''}$/$C^{4'''''}$/$C^{5'''''}$), 136.1 ($C^{6'''''}$), 170.6 ($C^{1'}$/$C^{1''}$/$C^{1'''}$), 172.4 ($C^{2''''}$), 174.4 ($C^{1'}$/$C^{1''}$/$C^{1'''}$); HRMS *m/z* (ESI$^+$) [Found: 517.2272, $C_{23}H_{34}N_4O_8$ requires $[M+Na]^+$ 517.2269]; LRMS *m/z* (ESI$^+$) 495 ($[M+H]^+$, 100%), 517 ($[M+Na]^+$, 91%); Analytical HPLC (method A) R_t 8.6 min, 92.8%.

Europium benzyl complex (EuBn), 4a

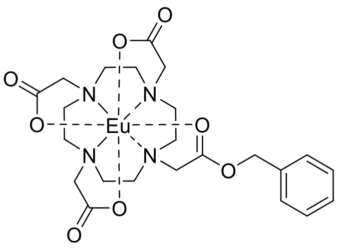

The reaction was carried out following the general lanthanide complexation procedure, using **S5** (81.9 mg, 166 μmol, 1.0 eq.) and europium triflate (111 mg, 185 μmol, 1.12 eq.) in MES buffer (2.54 mL, 1 M, pH 6). The reaction was stirred at room temperature for 1 hour, purified by semi-preparative HPLC method 3 and lyophilised for 2 days to yield compound **4a** as a colourless solid (43.2 mg, 41%): ^{1}H NMR (400 MHz, D_2O)*: −19.5, −17.2, −16.2, −15.8, −14.7, −14.2, −13.1, −10.8, −10.1, −9.6, −9.3, −9.1, −8.7, −8.1, −6.8, −6.4, −5.8, −4.1, −0.9, −0.5, 0.4, 3.1, 6.0, 6.2, 6.5, 7.4, 8.0, 8.5, 10.5, 25.5, 25.8, 26.5, 33.0, 34.0; HRMS *m/z* (ESI$^+$) [Found: 645.1420, $C_{23}H_{31}N_4O_8Eu$ requires $[M+H]^+$ 645.1427]; LRMS *m/z* (ESI$^+$) 645 ($[M+H]^+$, 97%), 667 ($[M+Na]^+$, 30%); Analytical HPLC (method A) R_t 8.9 min, 99.0%.

*^{1}H NMR contains 2% EuDOTA, therefore total purity ~97%, all peaks reported including EuDOTA.

Terbium benzyl complex (TbBn), 4b

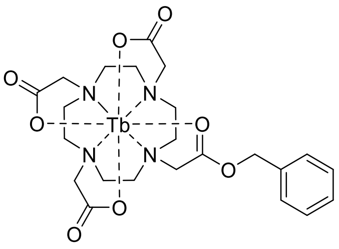

The reaction was carried out following the general lanthanide complexation procedure, using **S5** (53.4 mg, 108 μmol, 1.0 eq.) and terbium triflate (66.0 mg, 109 μmol, 1.01 eq.) in MES buffer (1.65 mL, 1 M, pH 6). The reaction was stirred at room temperature for 1 hour, purified by semi-preparative HPLC method 3 and lyophilised for 2 days to yield compound **4b** as a colourless solid (27.1 mg, 39%): ^{1}H NMR (400 MHz, D_2O)*: −419.3, −401.4, −344.0, −333.7, −316.8, −182.9, −150.7, −119.6, −99.4, −79.4, −69.0, −67.8, −39.0, −38.7, −37.9, −31.5, −25.5, −20.6, −11.4, −4.3, 12.0, 24.1, 83.8, 147.2, 156.1, 220.9, 233.2, 288.7; HRMS *m/z* (ESI^+) [Found: 651.1476, $C_{23}H_{31}N_4O_8Tb$ requires $[M+H]^+$ 651.1468]; LRMS *m/z* (ESI^+) 651 ($[M+H]^+$, 100%), 673 ($[M+Na]^+$, 71%); Analytical HPLC (method A) R_t 8.8 min, >99%.

*^{1}H NMR contains 1% TbDOTA, therefore total purity ~98%, all peaks reported including TbDOTA.

^{1}H and ^{13}C NMR Spectra of Novel and Final Compounds

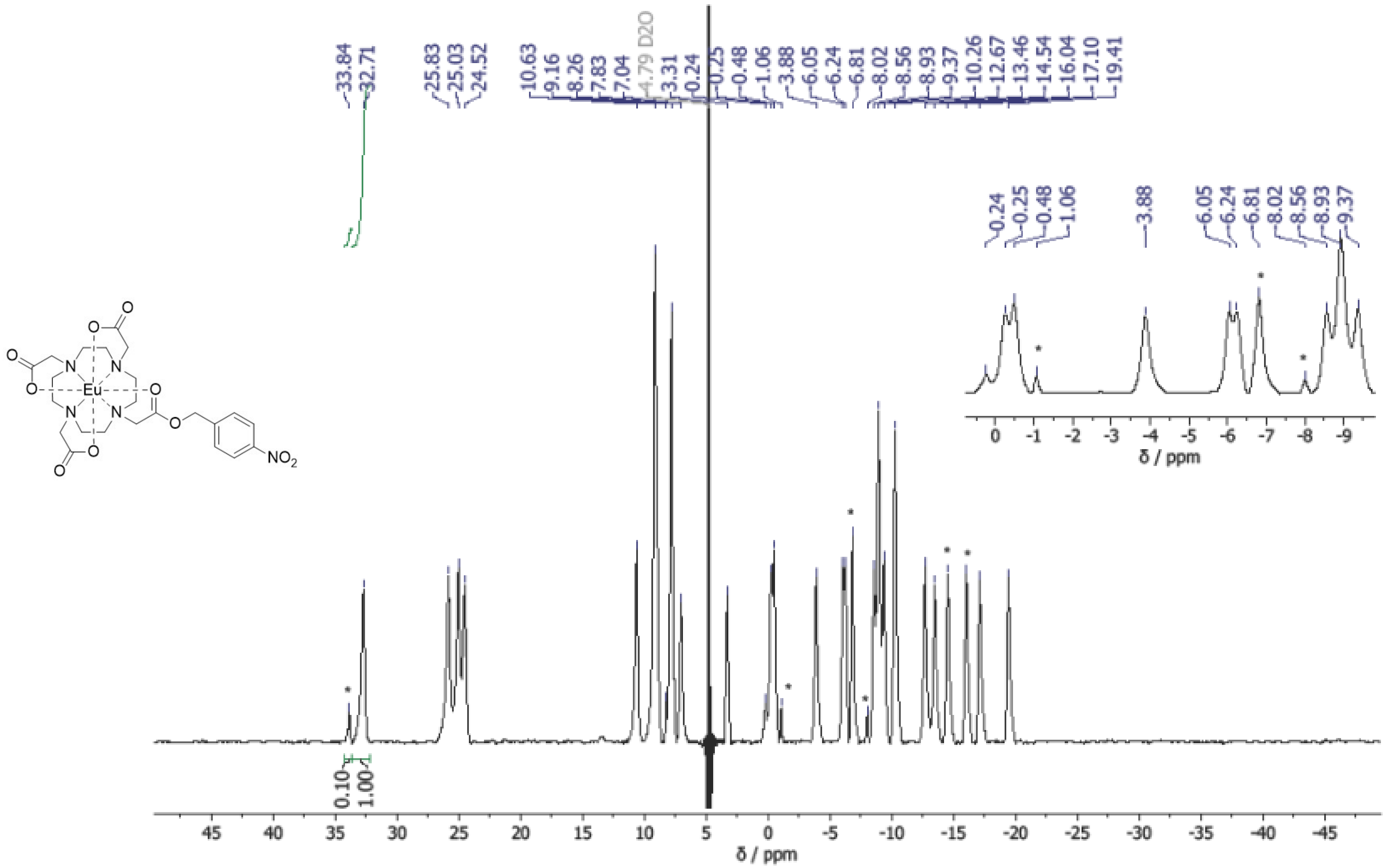

Figure S51: The ^{1}H (for europium complexes) NMR spectrum (400 MHz) of EuNB **1a** in D_2O. The stars represent peaks which may arise due to the presence of EuDOTA as an impurity. The integrals suggest that ~2% EuDOTA is present (assuming the EuDOTA peak contains 4 protons due to symmetry and the EuNB peak contains 1 proton).

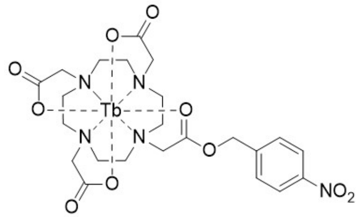

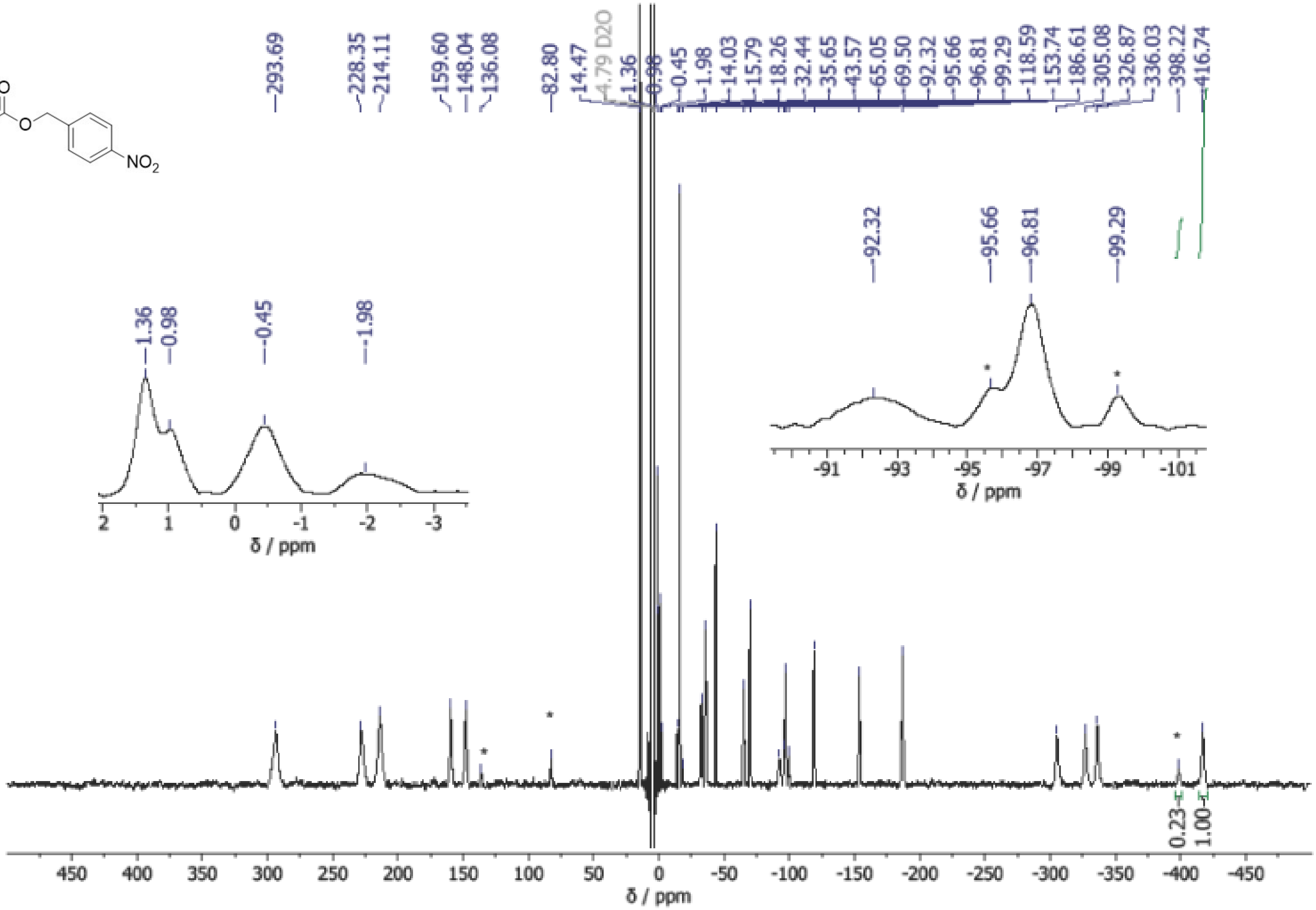

Figure S52: The ^{1}H (for terbium complexes) NMR spectrum (400 MHz) of TbNB **1b** in D_2O. The stars represent peaks which may arise due to the presence of TbDOTA as an impurity. The integrals suggest that ~5% TbDOTA is present (assuming the TbDOTA peak contains 4 protons due to symmetry and the TbNB peak contains 1 proton).

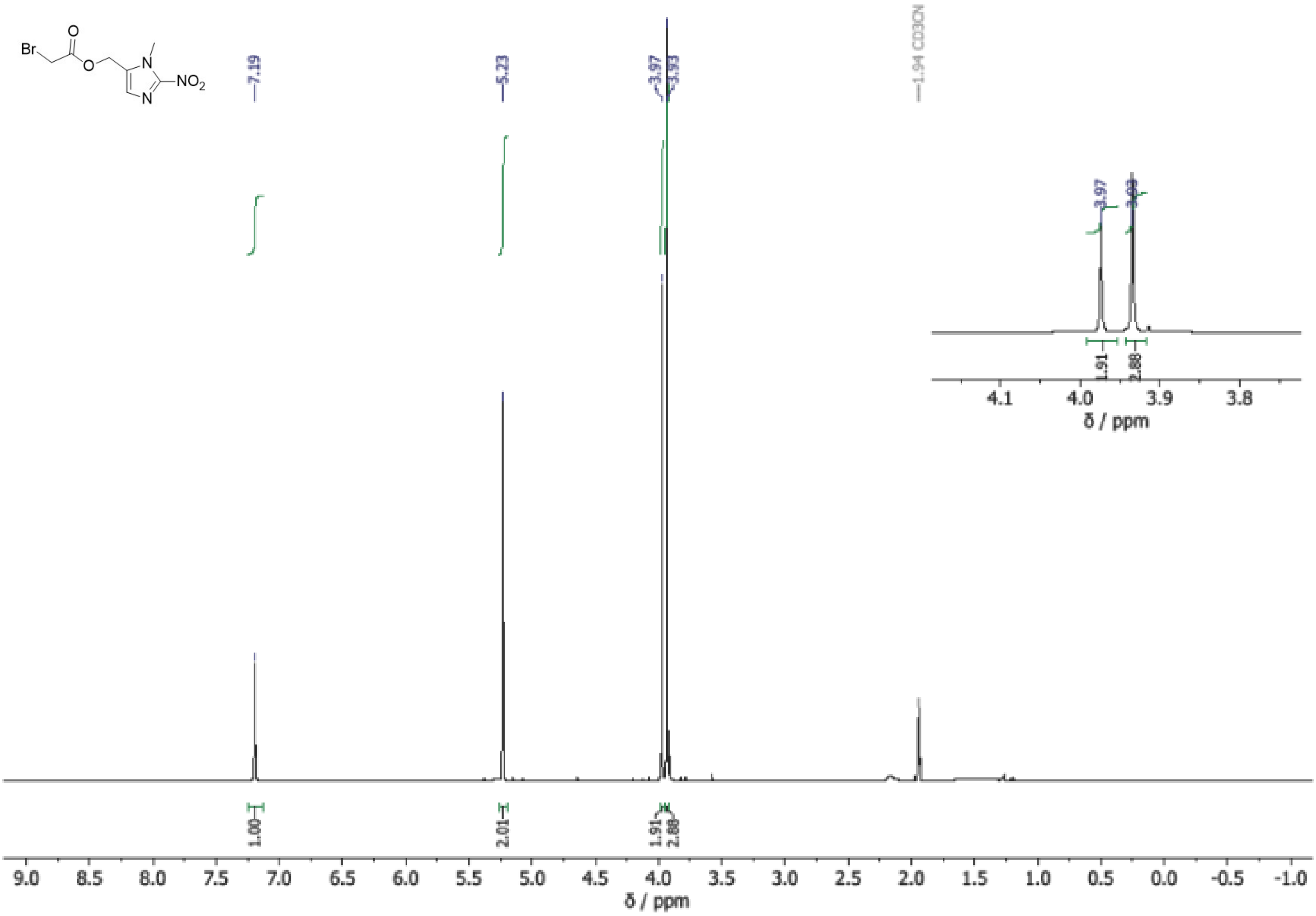

Figure S53: The ^{1}H NMR spectrum (600 MHz) of (1-methyl-2-nitro-1H-imidazol-5-yl)methyl 2-bromoacetate **7** in CD_3CN.

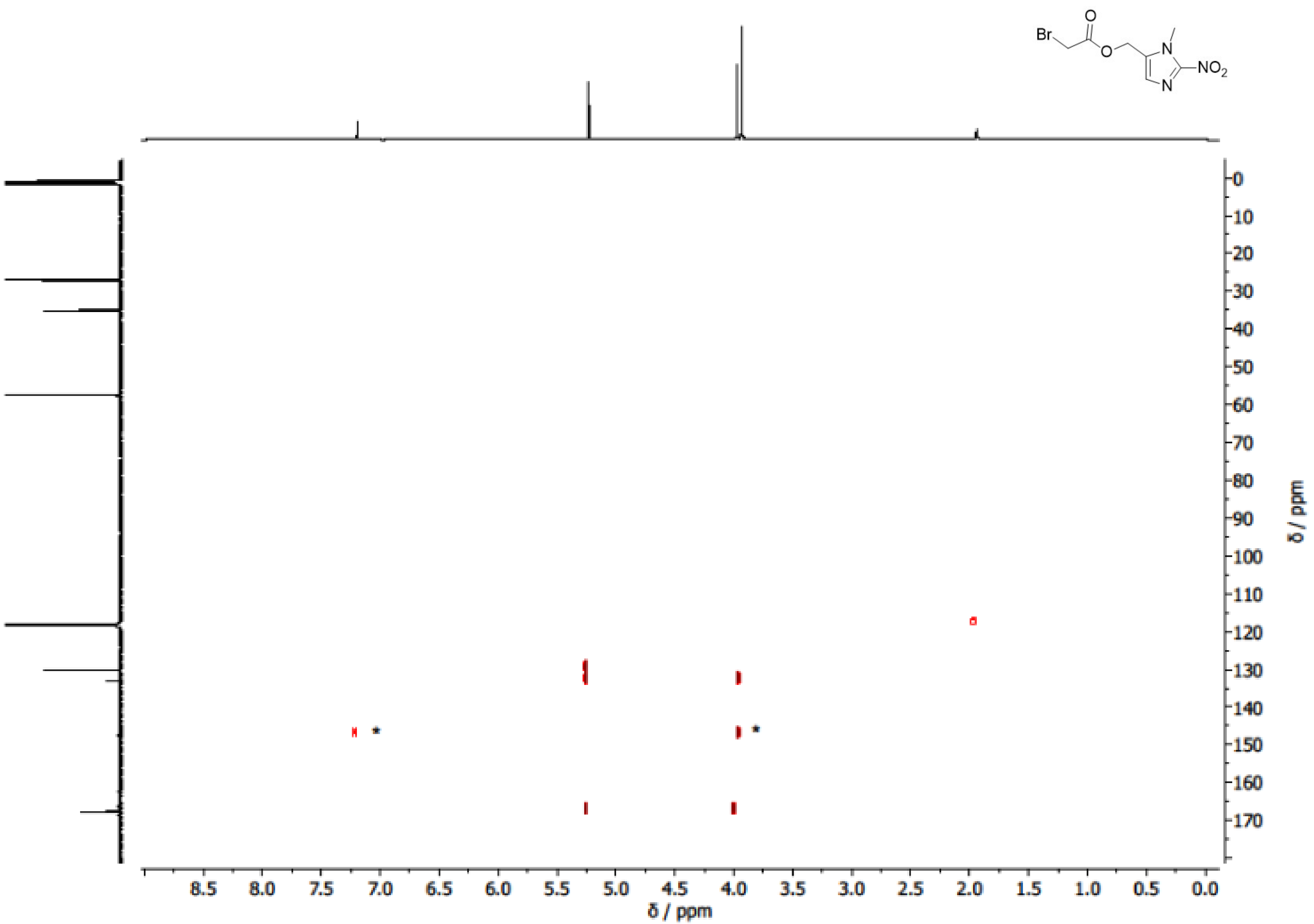

Figure S54: The HMBC NMR spectrum of (1-methyl-2-nitro-1H-imidazol-5-yl)methyl 2-bromoacetate **7** in CD_3CN, to prove the presence of the low intensity peaks in the ^{13}C NMR spectrum (highlighted with an asterisk).

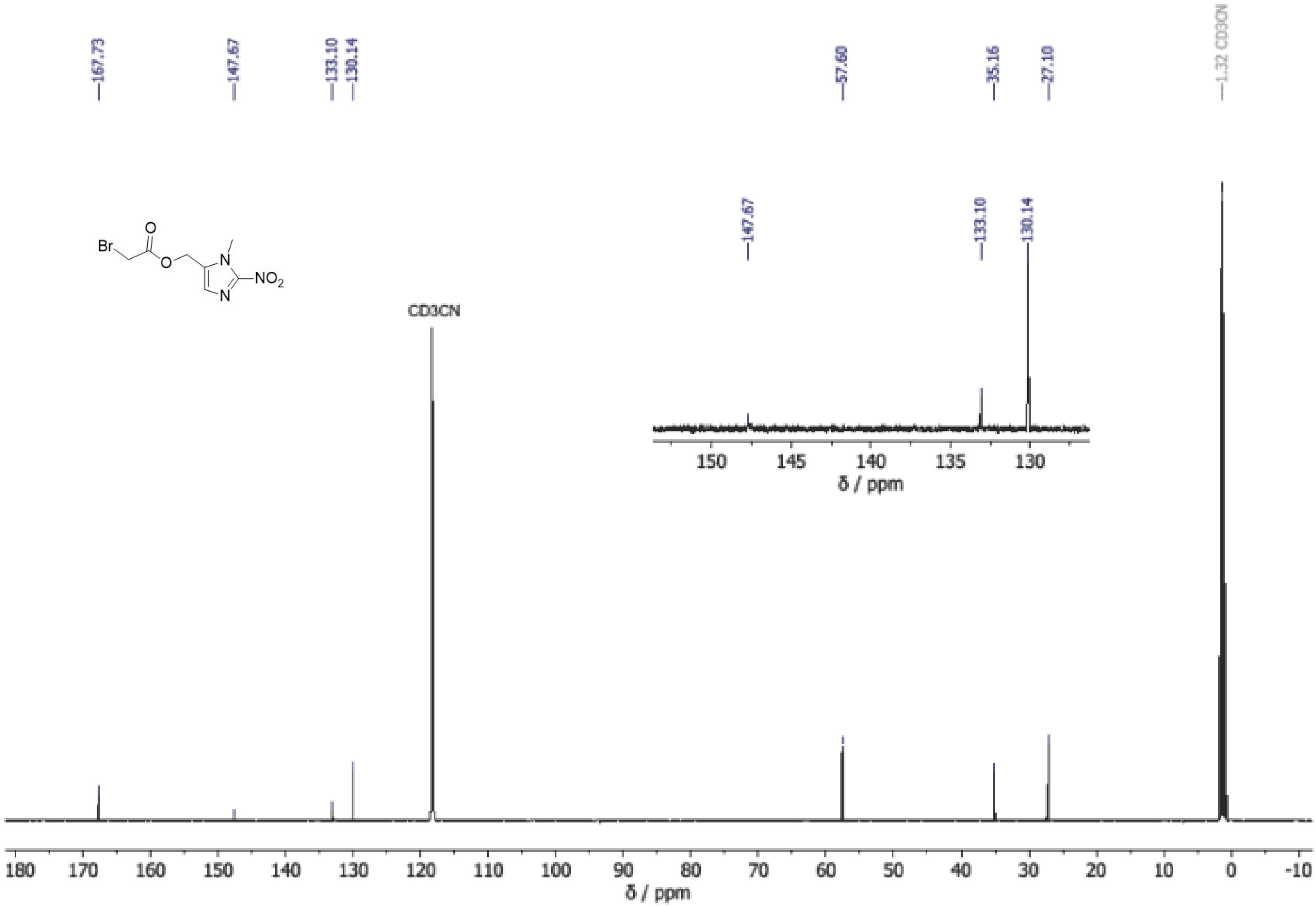

Figure S55: The ^{13}C NMR (151 MHz) spectrum of (1-methyl-2-nitro-1H-imidazol-5-yl)methyl 2-bromoacetate **7** in CD_3CN.

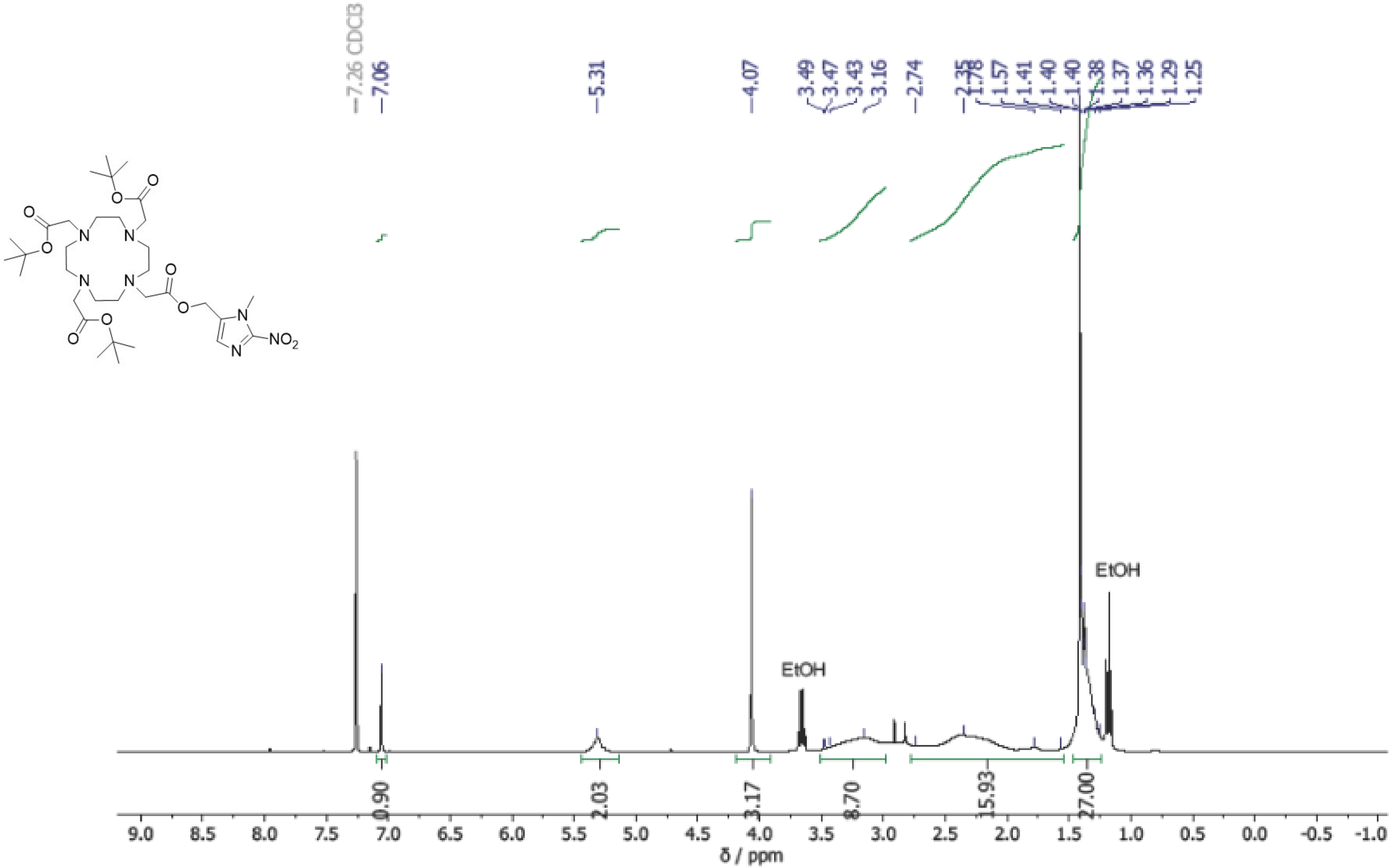

Figure S56: The ^{1}H NMR spectrum (400 MHz) of tri-tert-butyl 2,2',2''-(10-(2-((1-methyl-2-nitro-1H-imidazol-5-yl)methoxy)-2-oxoethyl)-1,4,7,10-tetraazacyclododecane-1,4,7-triyl)triacetate **9** in $CDCl_3$.

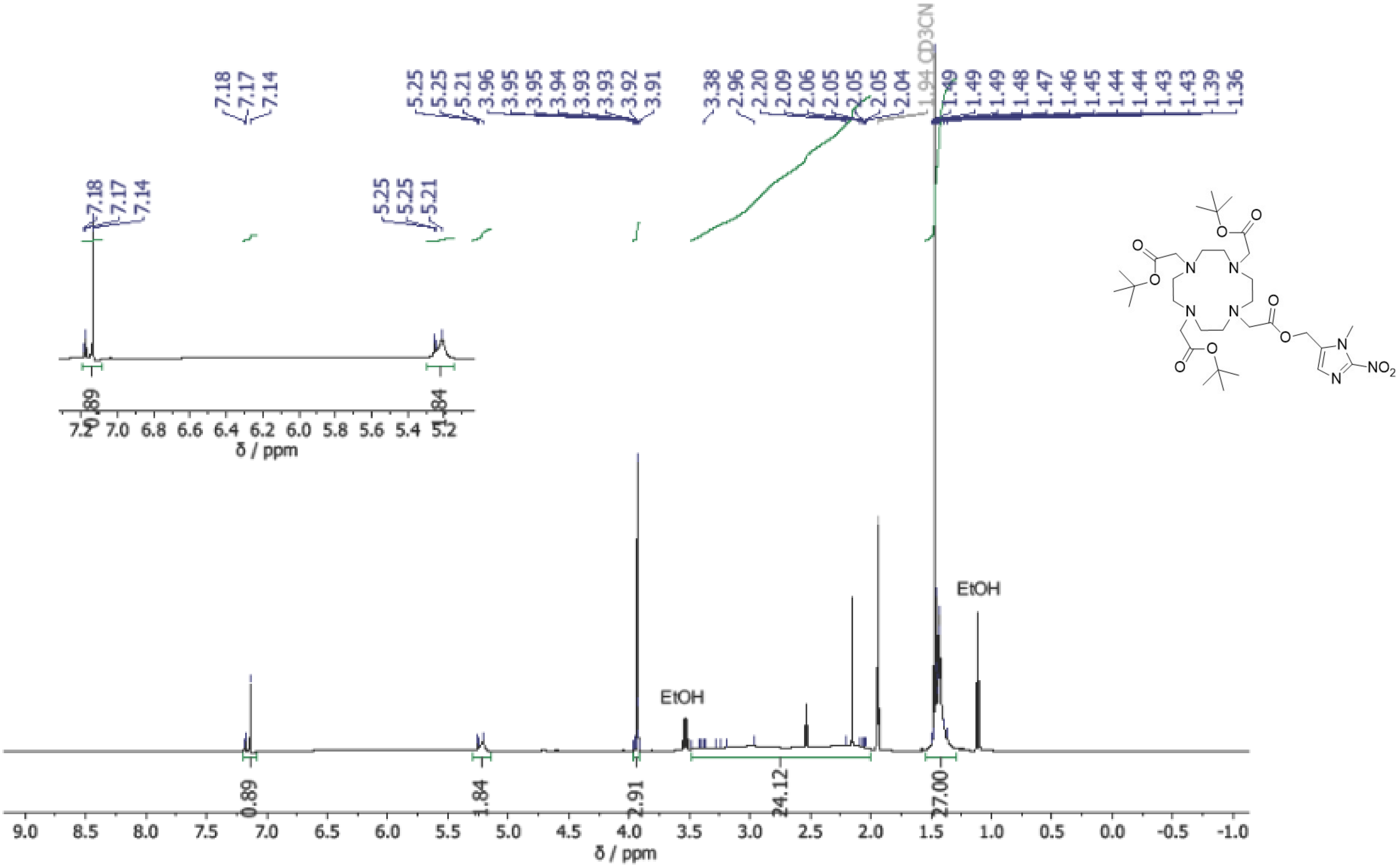

Figure S57: The ^{1}H NMR spectrum (600 MHz) of tri-tert-butyl 2,2',2''-(10-(2-((1-methyl-2-nitro-1H-imidazol-5-yl)methoxy)-2-oxoethyl)-1,4,7,10-tetraazacyclododecane-1,4,7-triyl)triacetate **9** in CD_3CN.

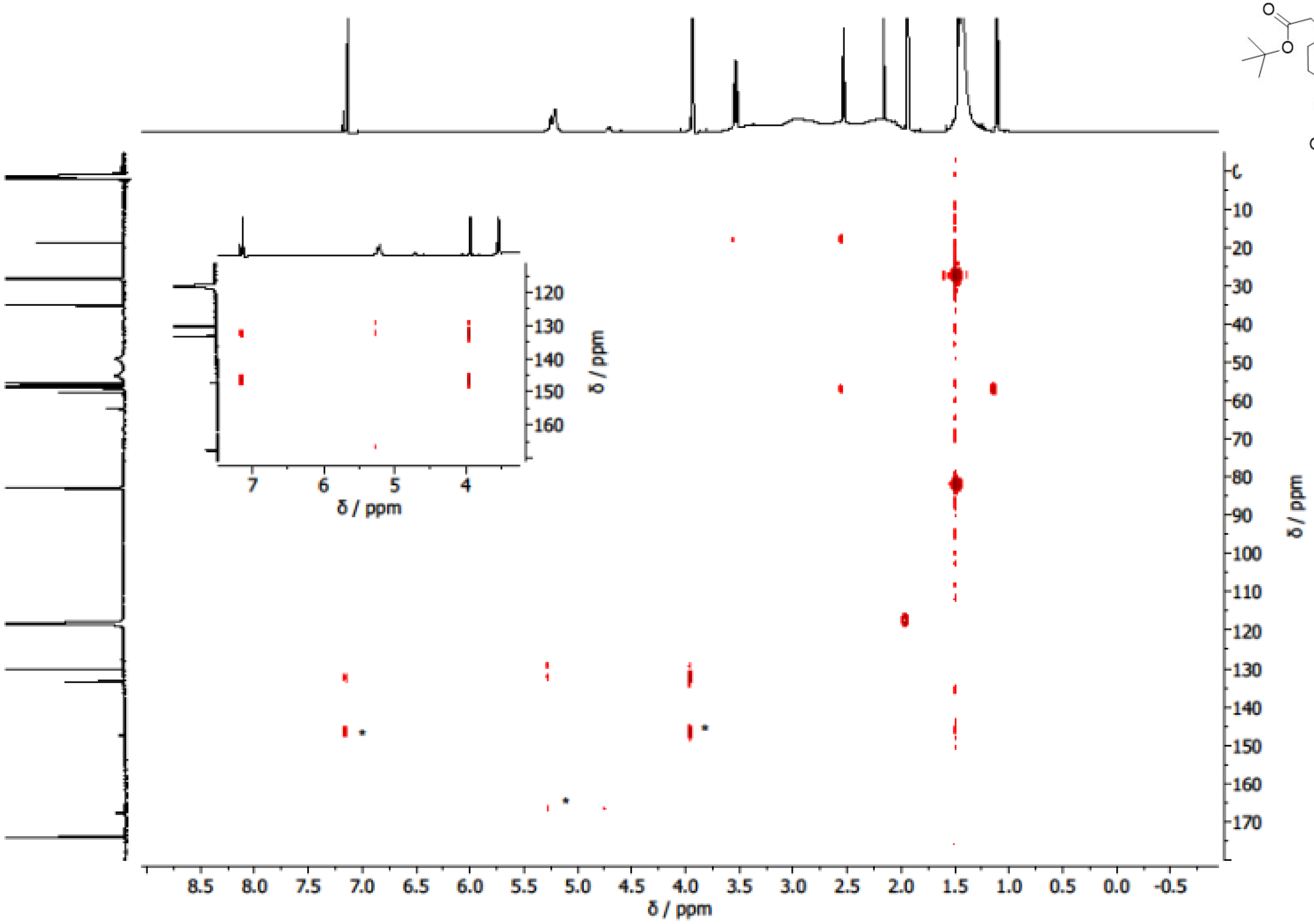

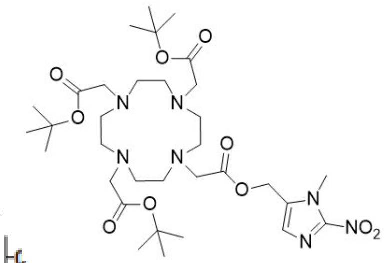

Figure S58: The HMBC NMR spectrum of tri-tert-butyl 2,2',2''-(10-(2-((1-methyl-2-nitro-1H-imidazol-5-yl)methoxy)-2-oxoethyl)-1,4,7,10-tetraazacyclododecane-1,4,7-triyl)triacetate **9** in CD_3CN, to prove the presence of the low intensity peaks in the ^{13}C NMR spectrum (highlighted with an asterisk).

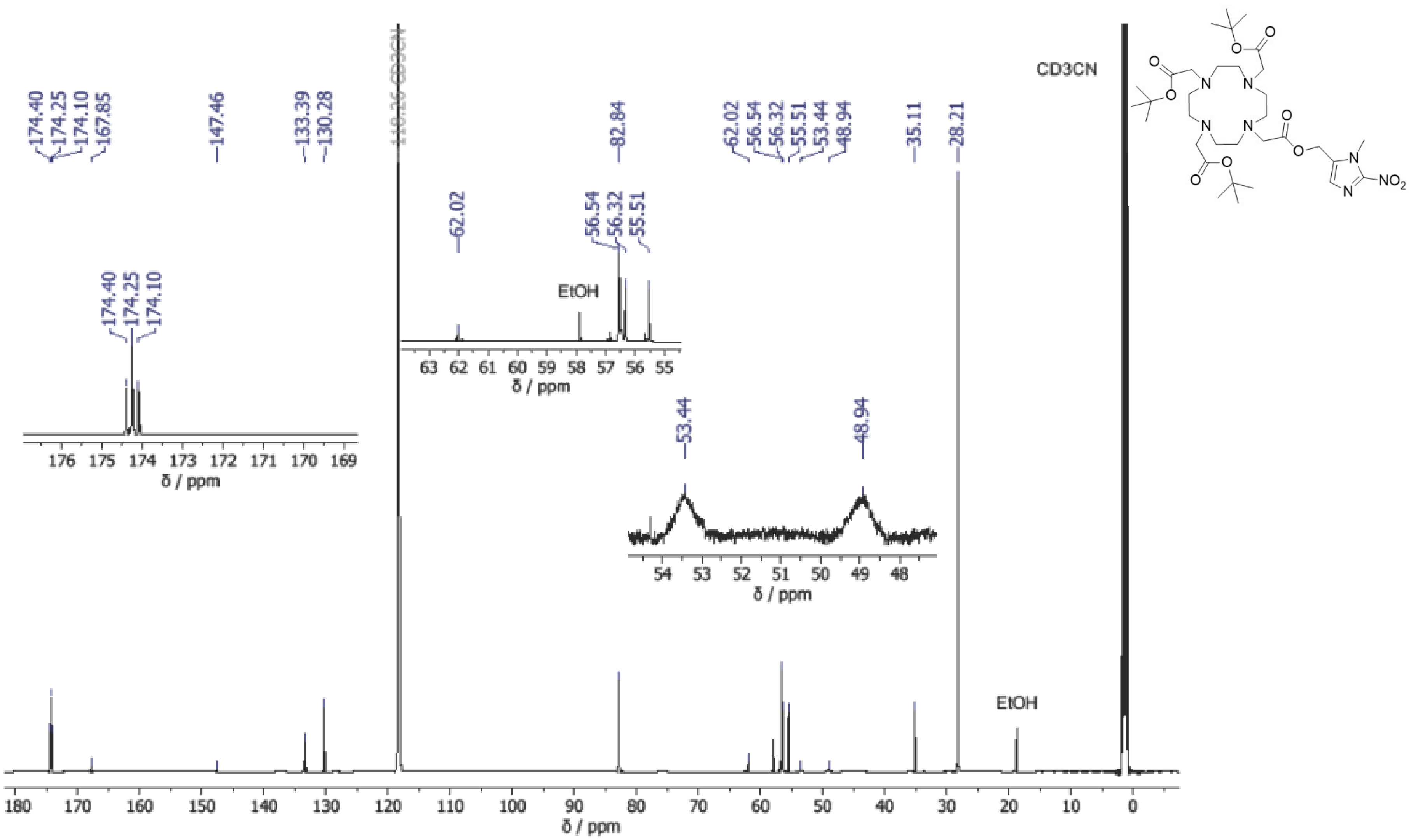

Figure S59: The ^{13}C NMR spectrum (151 MHz) of tri-tert-butyl 2,2',2''-(10-(2-((1-methyl-2-nitro-1H-imidazol-5-yl)methoxy)-2-oxoethyl)-1,4,7,10-tetraazacyclododecane-1,4,7-triyl)triacetate **9** in CD_3CN.

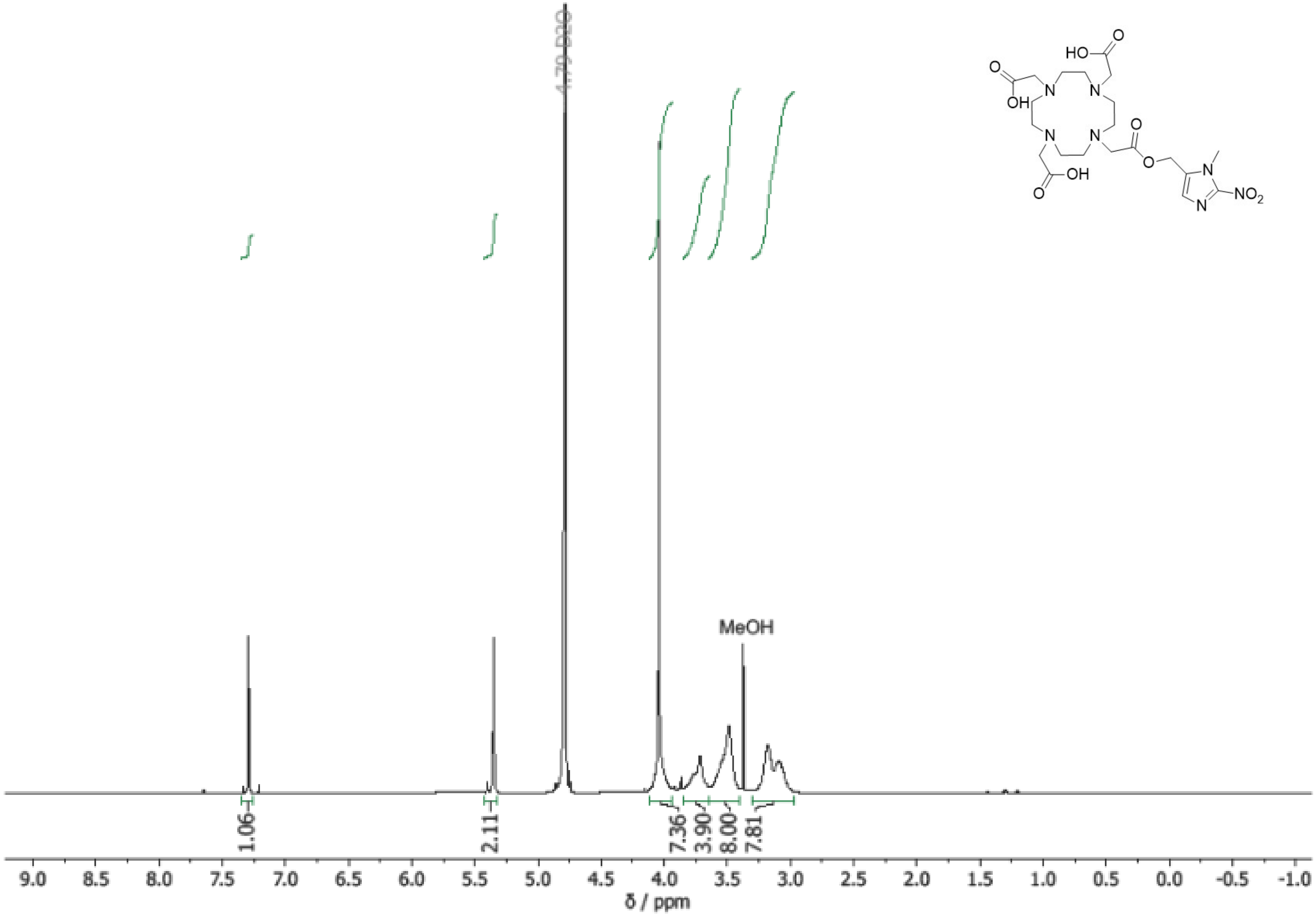

Figure S60: The ^{1}H NMR spectrum (600 MHz) of 2,2',2''-(10-(2-((1-methyl-2-nitro-1H-imidazol-5-yl)methoxy)-2-oxoethyl)-1,4,7,10-tetraazacyclododecane-1,4,7-triyl)triacetic acid **11** in D_2O, with residual MeOH remaining for reference in the ^{13}C NMR spectrum.

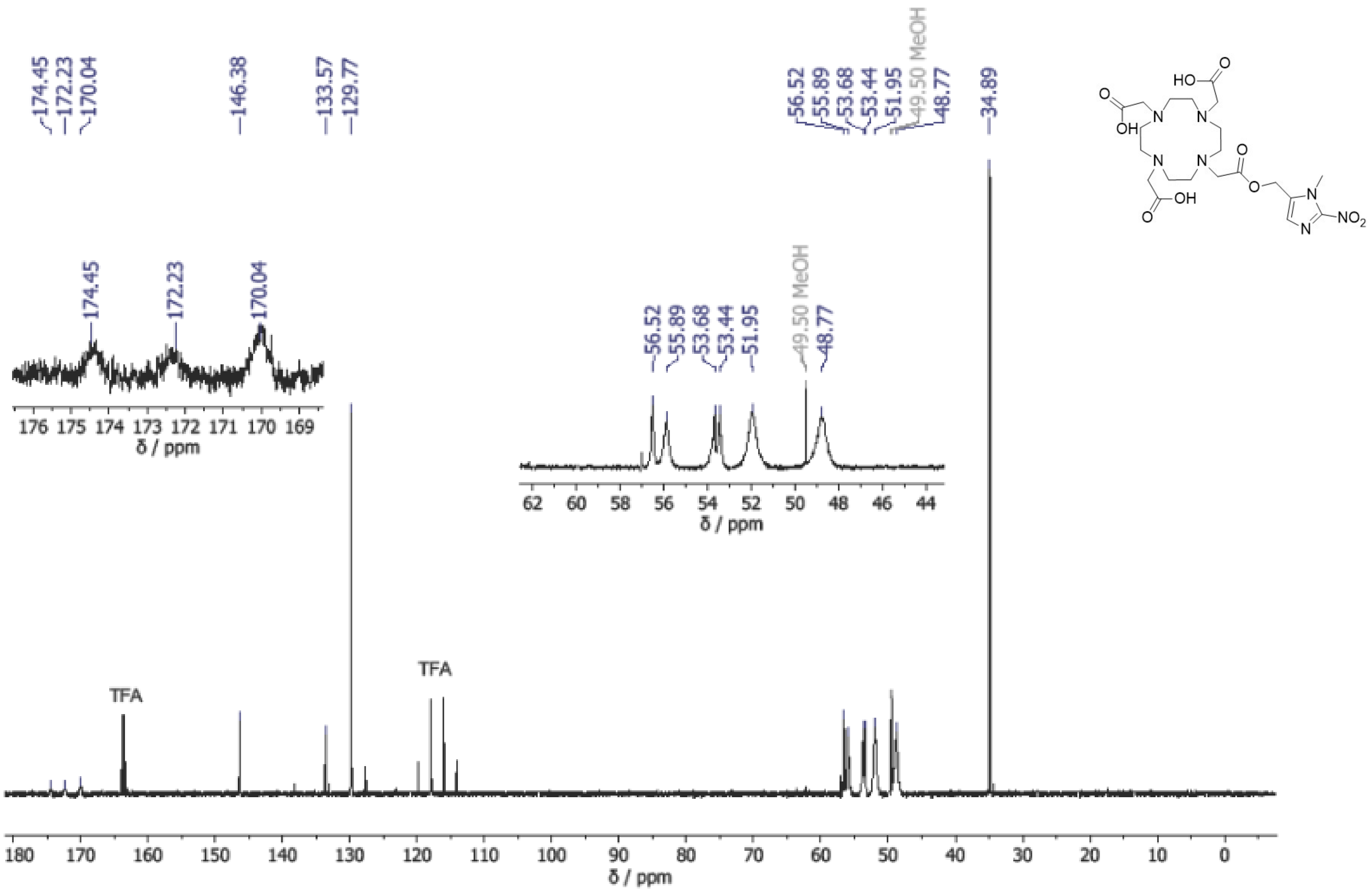

Figure S61: The ^{13}C NMR spectrum (151 MHz) of 2,2',2''-(10-(2-((1-methyl-2-nitro-1H-imidazol-5-yl)methoxy)-2-oxoethyl)-1,4,7,10-tetraazacyclododecane-1,4,7-triyl)triacetic acid **11** in D_2O, with residual MeOH remaining for reference.

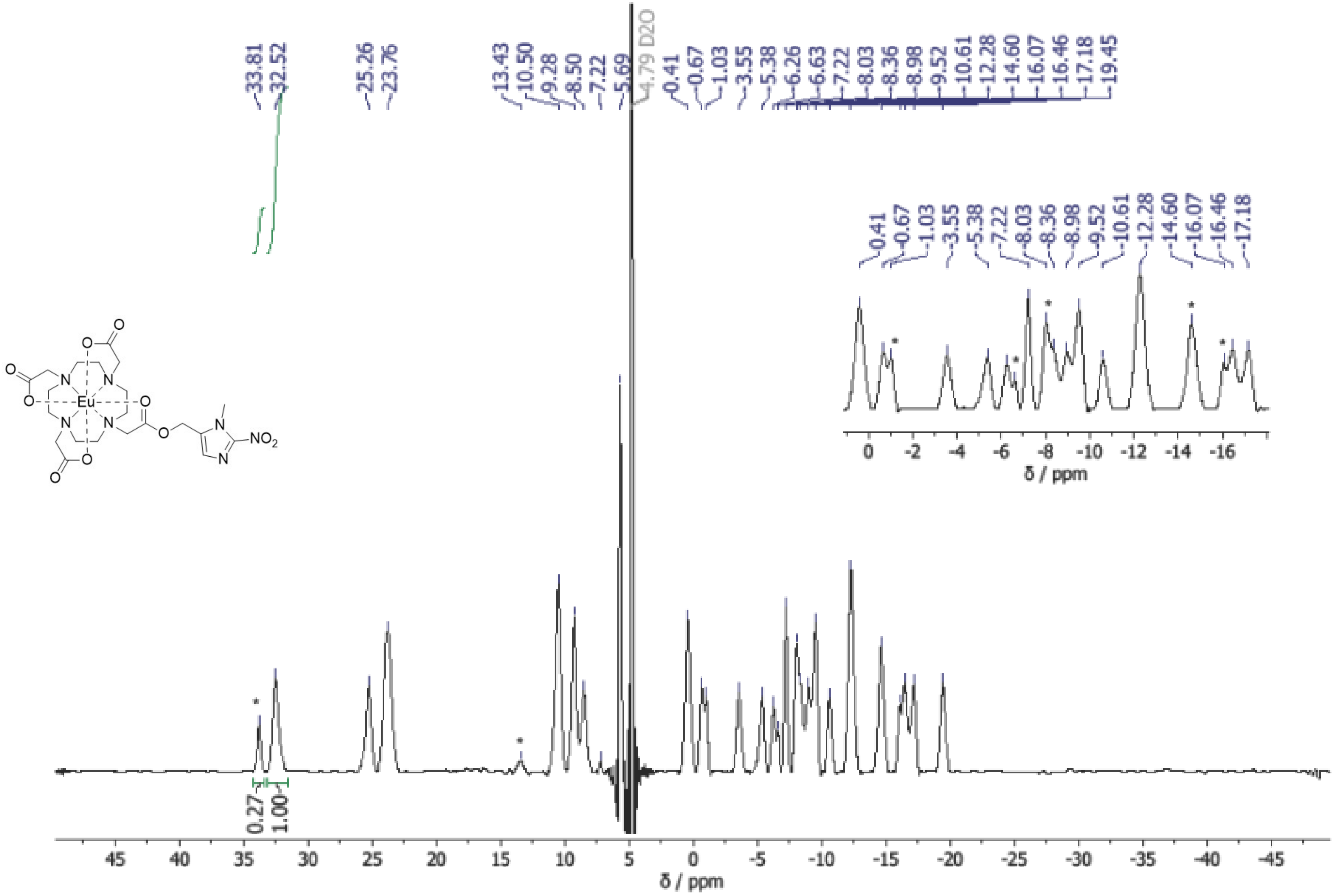

Figure S62: The ^{1}H (for europium complexes) NMR spectrum (400 MHz) of EuNI **2a** in D_2O. The stars represent peaks which may arise due to the presence of EuDOTA as an impurity. The integrals suggest that ~6% EuDOTA is present (assuming the EuDOTA peak contains 4 protons due to symmetry and the EuNI peak contains 1 proton).

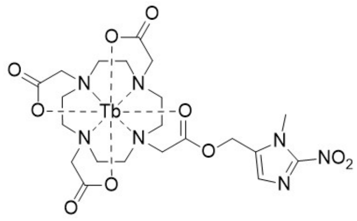

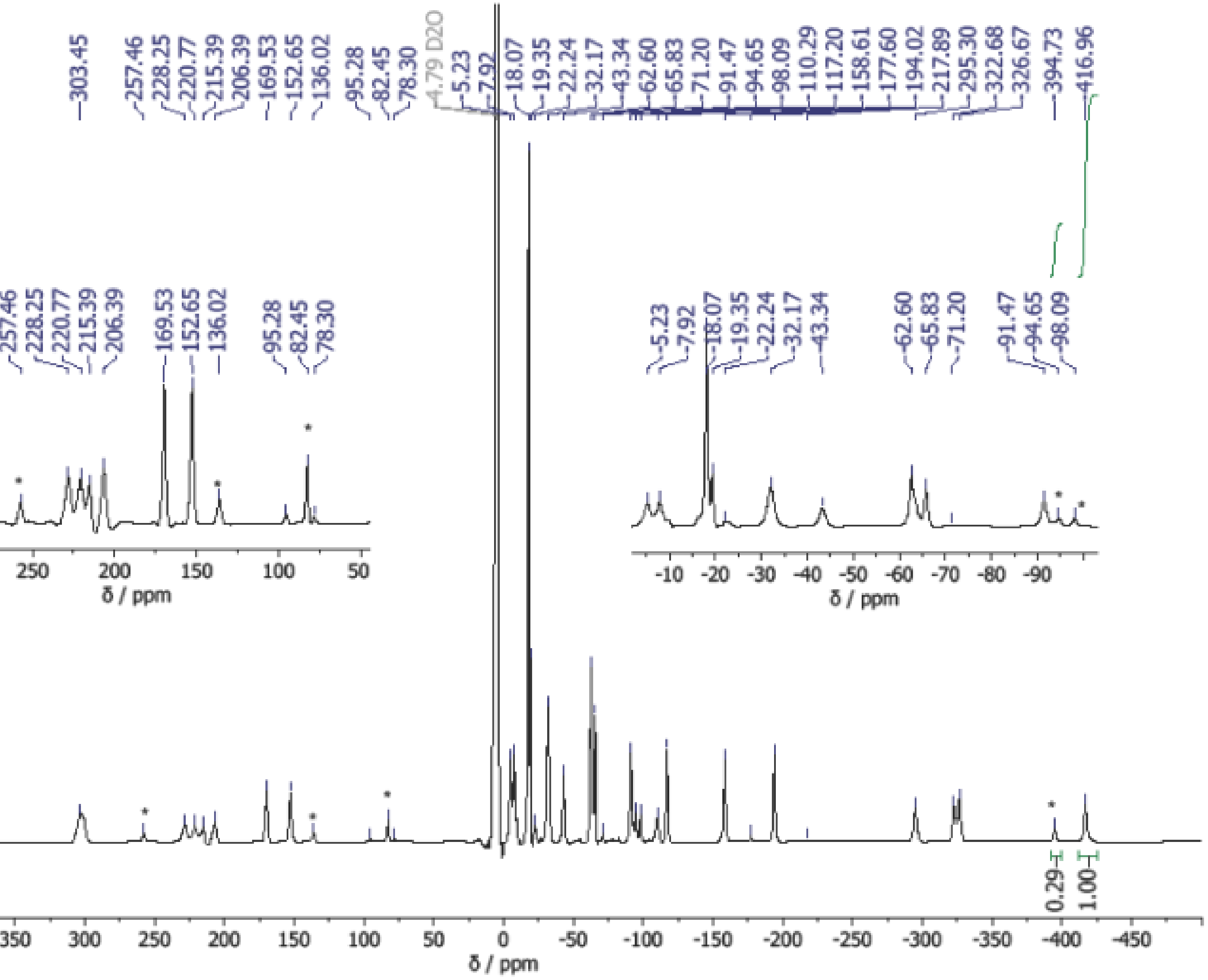

Figure S63: The ^{1}H (for terbium complexes) NMR (400 MHz) spectrum of TbNI **2b** in D_2O. The stars represent peaks which may arise due to the presence of TbDOTA as an impurity. The integrals suggest that ~7% TbDOTA is present (assuming the TbDOTA peak contains 4 protons due to symmetry and the TbNI peak contains 1 proton).

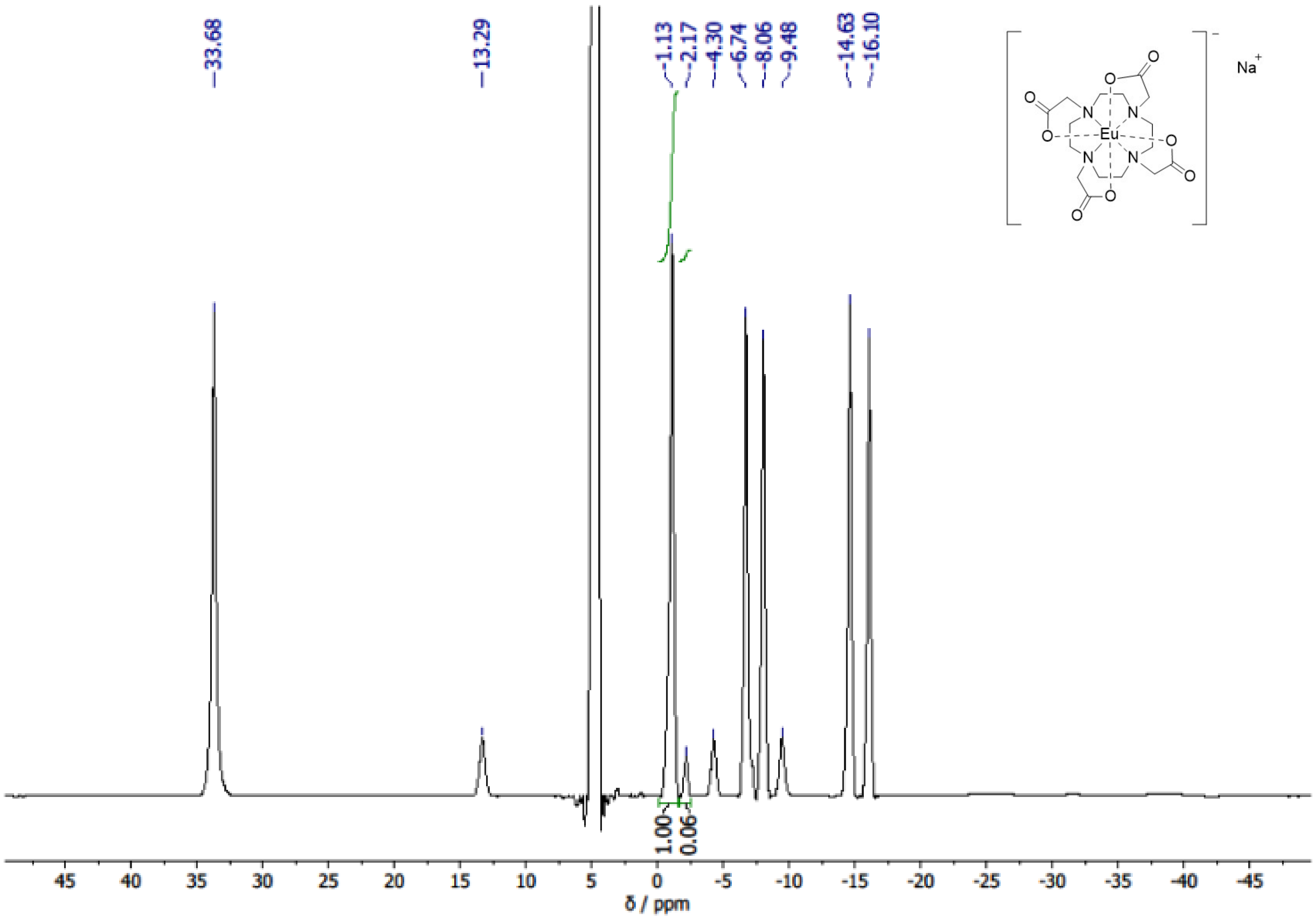

Figure S64: The ^{1}H (for europium complexes) NMR spectrum (400 MHz) of EuDOTA **3a** in D_2O. The integrals show the ratio between TSAP and SAP isomers.

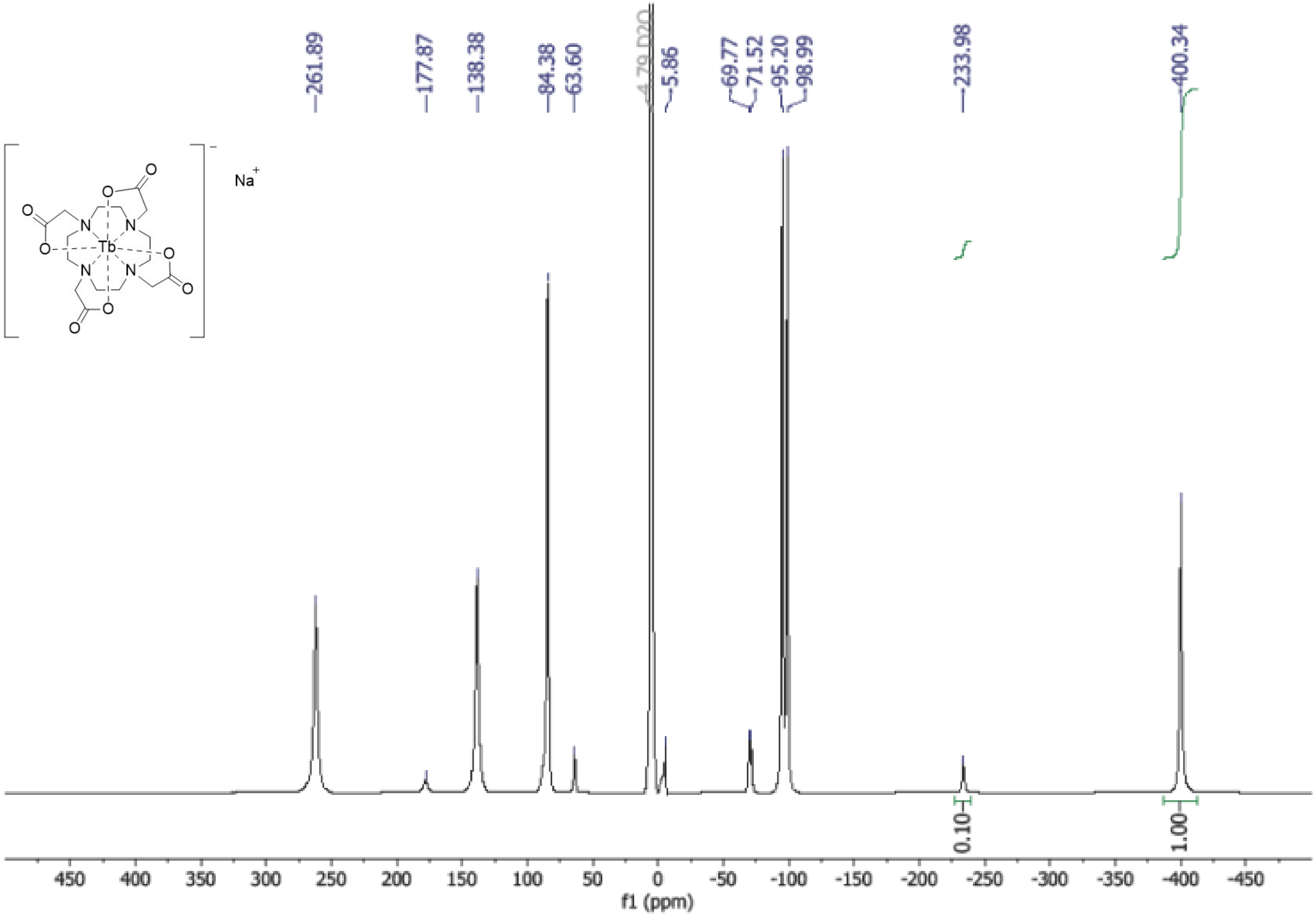

Figure S65: The ^{1}H (for terbium complexes) NMR spectrum (400 MHz) of TbDOTA **3b** in D_2O. The integrals show the ratio between TSAP and SAP isomers.

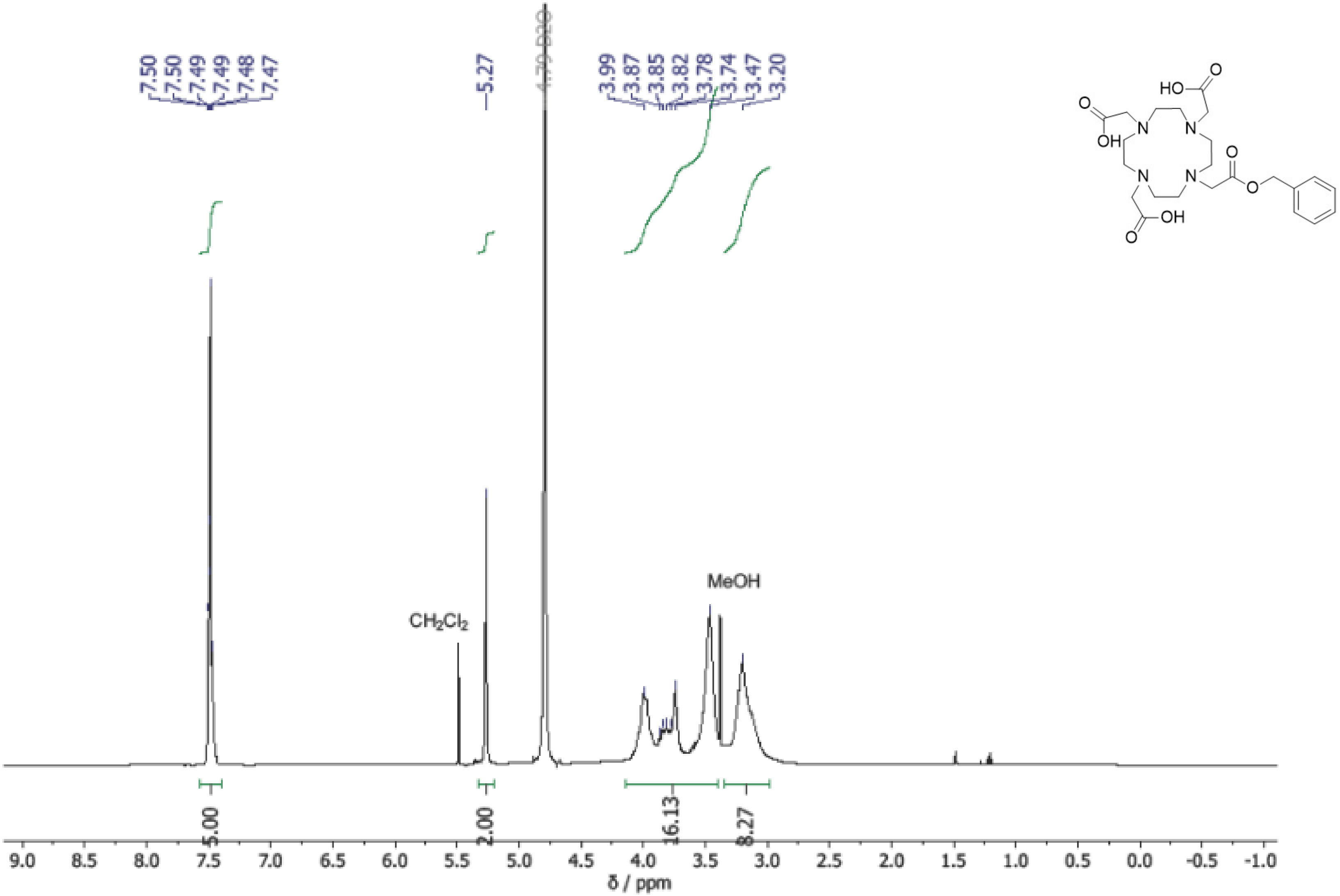

Figure S66: The ^{1}H NMR spectrum (400 MHz) of 2,2',2''-(10-(2-(benzyloxy)-2-oxoethyl)-1,4,7,10-tetraazacyclododecane-1,4,7-triyl)triacetic acid, **S5** in D_2O, with residual MeOH remaining for reference in the ^{13}C NMR spectrum.

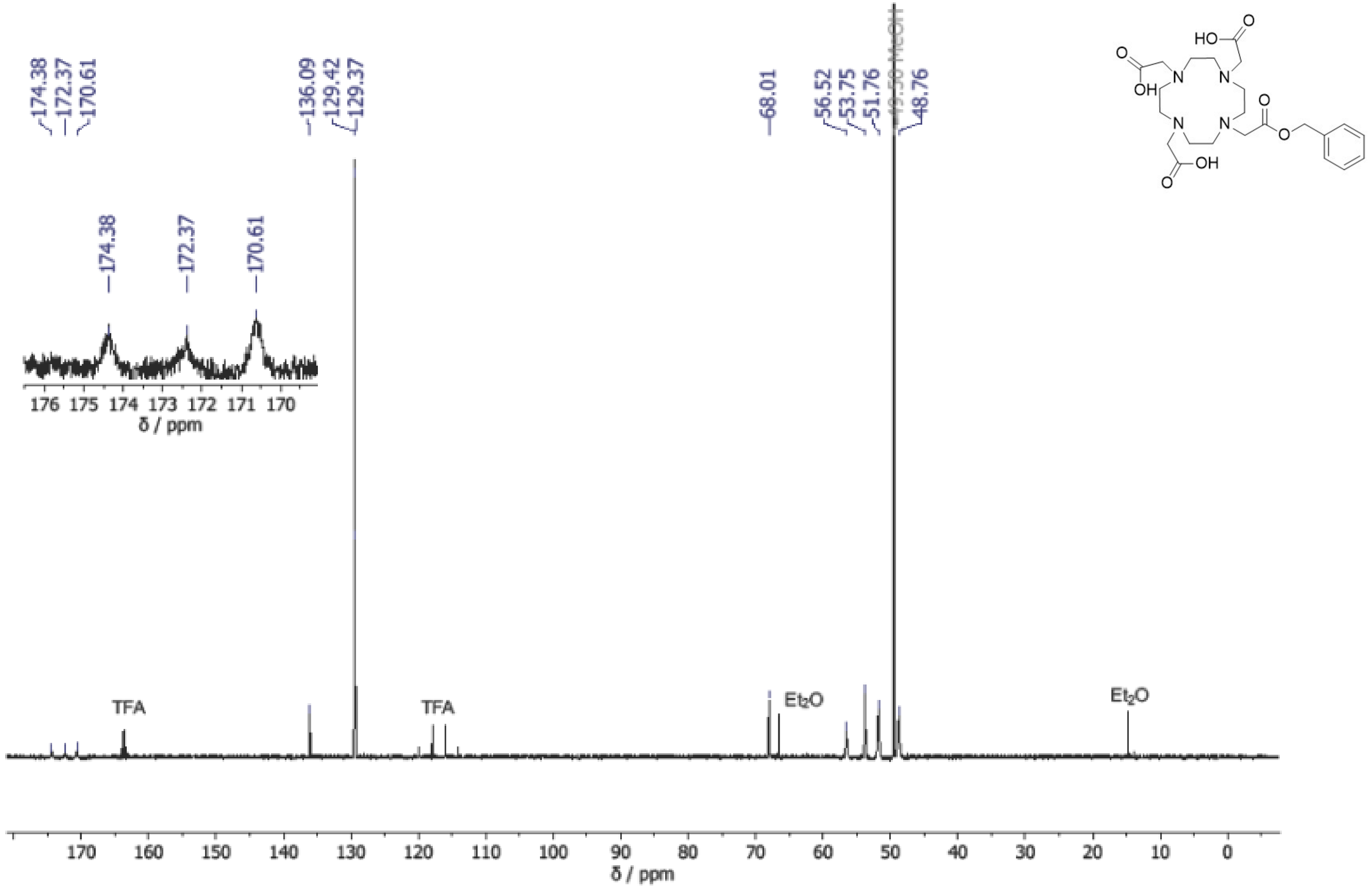

Figure S67: The ^{13}C NMR spectrum (151 MHz) of 2,2',2''-(10-(2-(benzyloxy)-2-oxoethyl)-1,4,7,10-tetraazacyclododecane-1,4,7-triyl)triacetic acid, **S5** in D_2O, with MeOH remaining for reference.

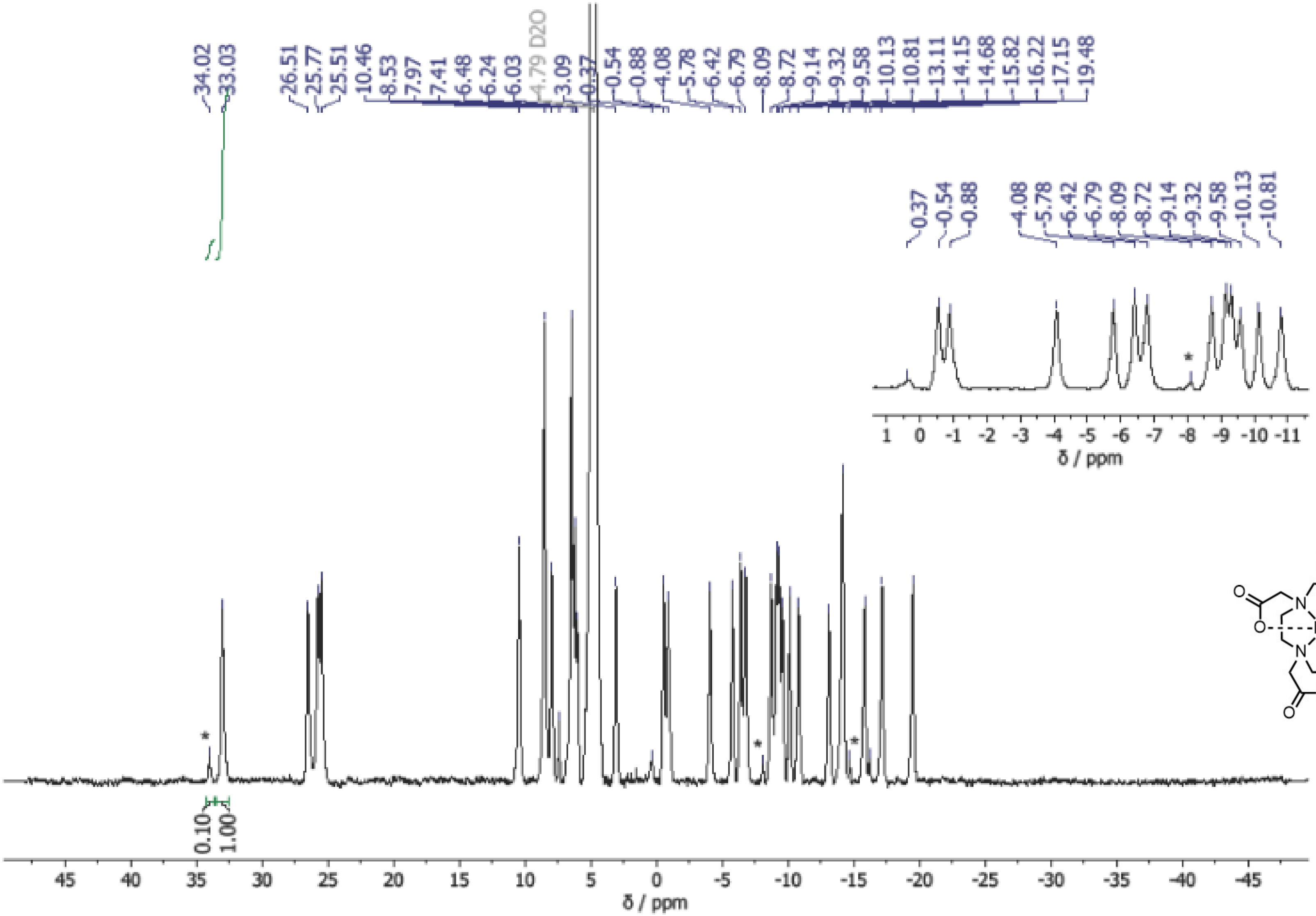

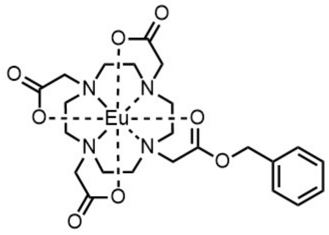

Figure S68: The ¹H (for europium complexes) NMR spectrum (400 MHz) of EuBn **4a** in D_2O. The stars represent peaks which may arise due to the presence of EuDOTA as an impurity. The integrals suggest that ~2% EuDOTA is present (assuming the EuDOTA peak contains 4 protons due to symmetry and the EuBn peak contains 1 proton).

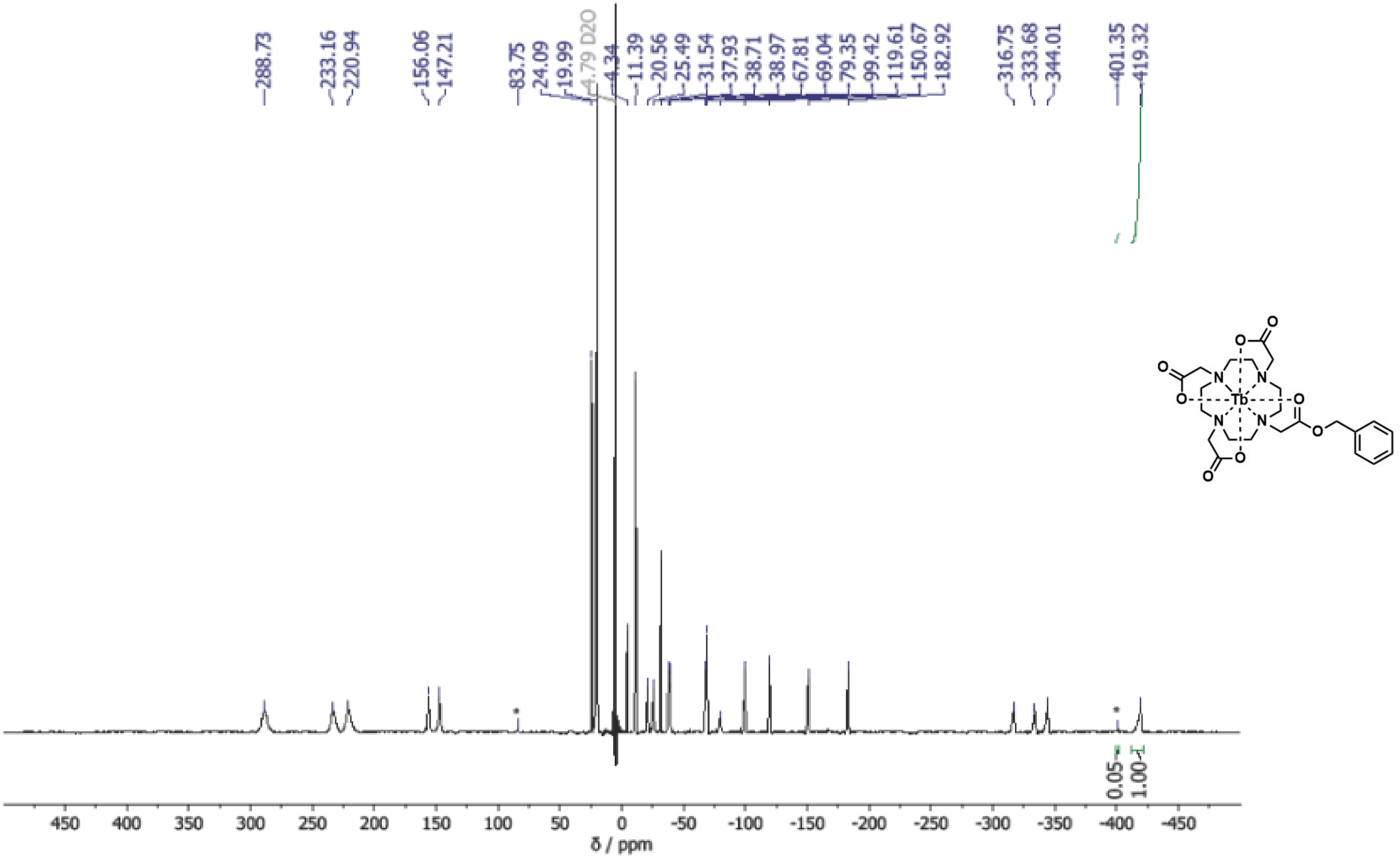

Figure S69: The ¹H (for terbium complexes) NMR spectrum (400 MHz) of TbBn **4b** in D_2O. The stars represent peaks which may arise due to the presence of TbDOTA as an impurity. The integrals suggest that ~1% EuDOTA is present (assuming the TbDOTA peak contains 4 protons due to symmetry and the TbBn peak contains 1 proton).

LCMS/HPLC Traces of Compounds

Intermediates

Tri-*tert*-butyl 2,2',2''-(10-(2-((4-nitrobenzyl)oxy)-2-oxoethyl)-1,4,7,10-tetraazacyclododecane-1,4,7-triyl)triacetate, **8** – LCMS method 1

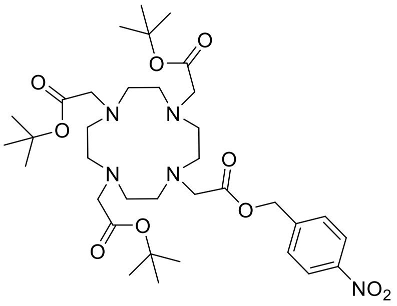

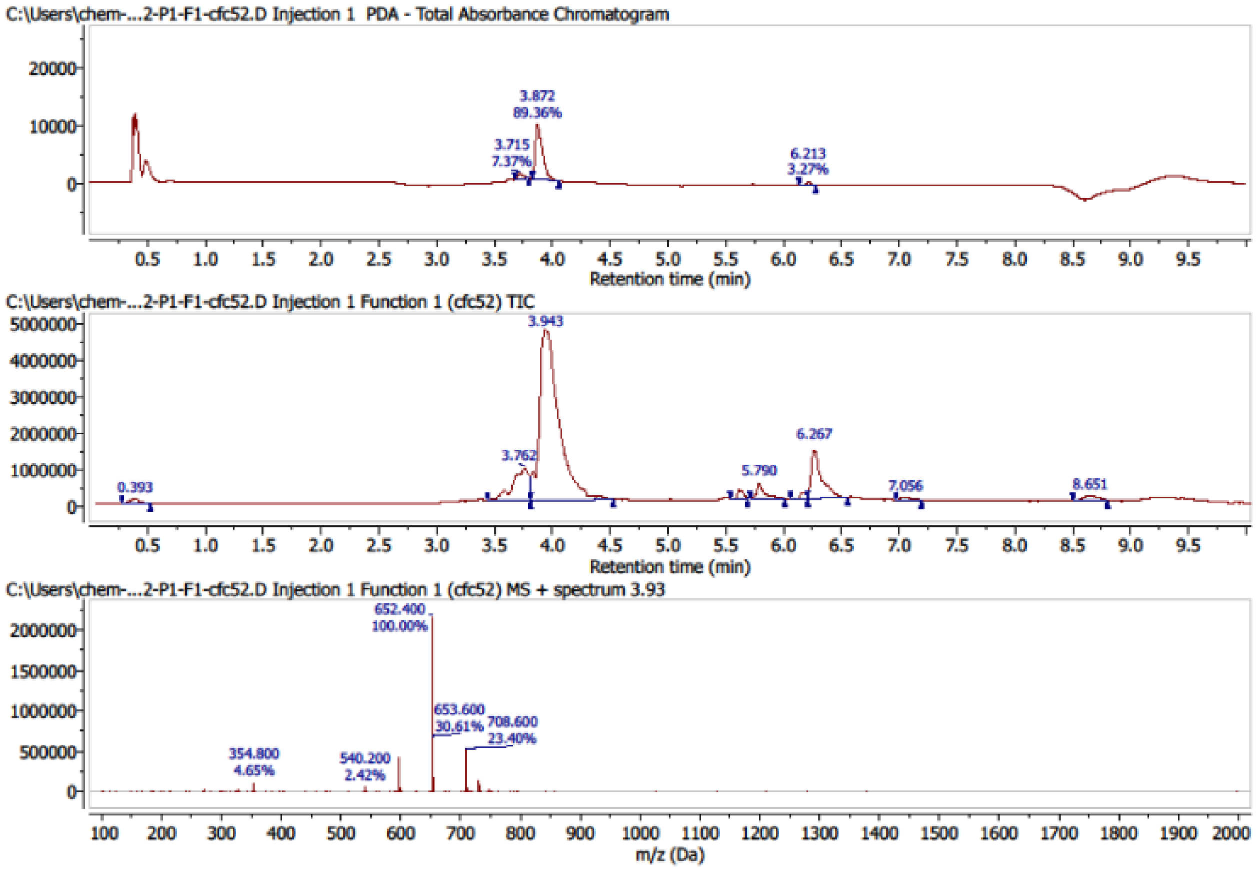

2,2',2''-(10-(2-((4-Nitrobenzyl)oxy)-2-oxoethyl)-1,4,7,10-tetraazacyclododecane-1,4,7-triyl)triacetic acid, **10** - LCMS method 1

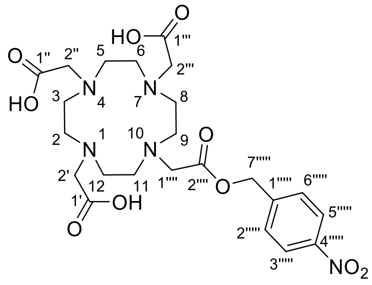

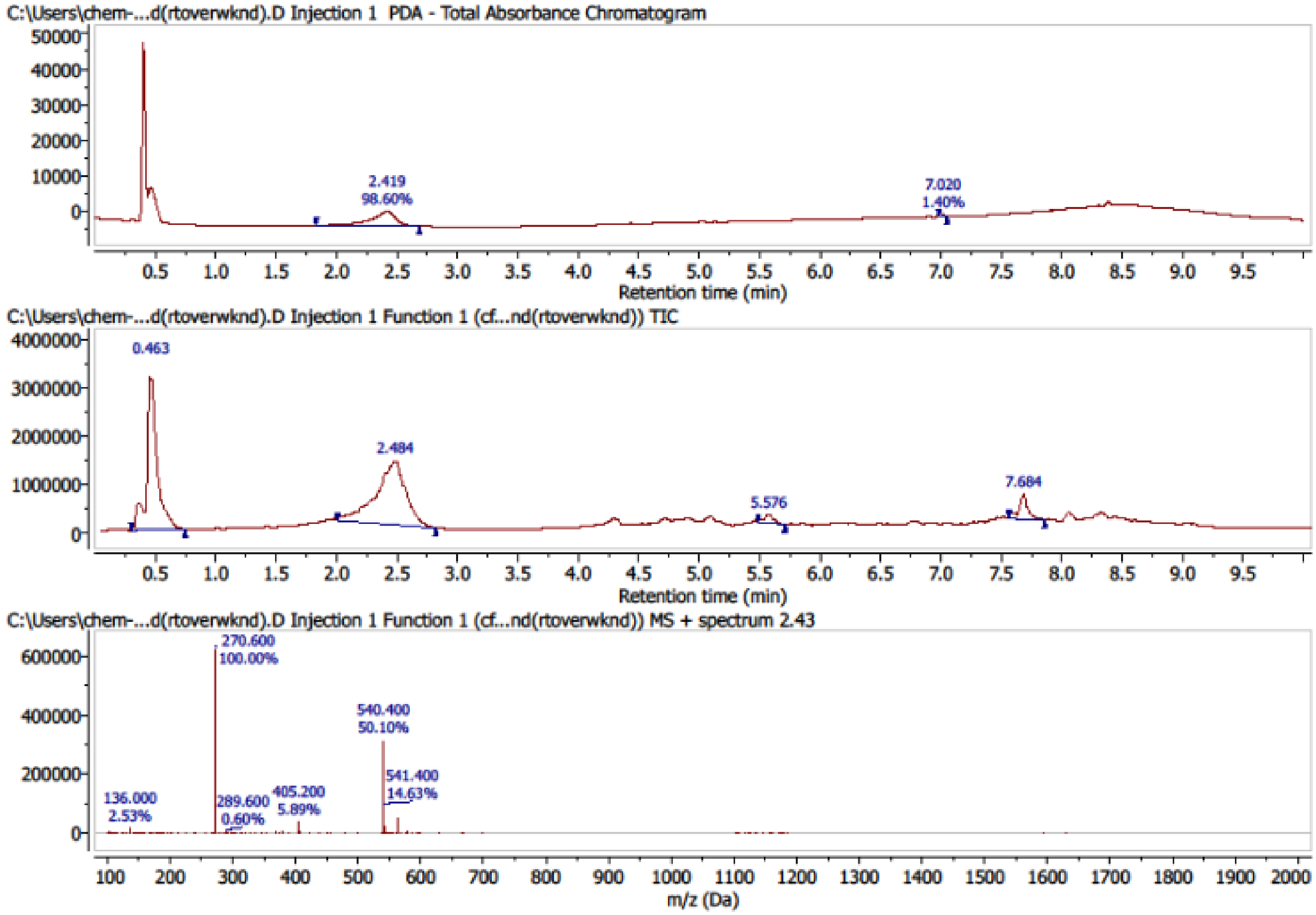

(1-Methyl-2-nitro-1*H*-imidazol-5-yl)methyl 2-bromoacetate, **7** – LCMS method 1

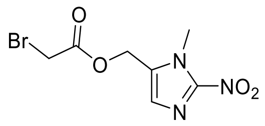

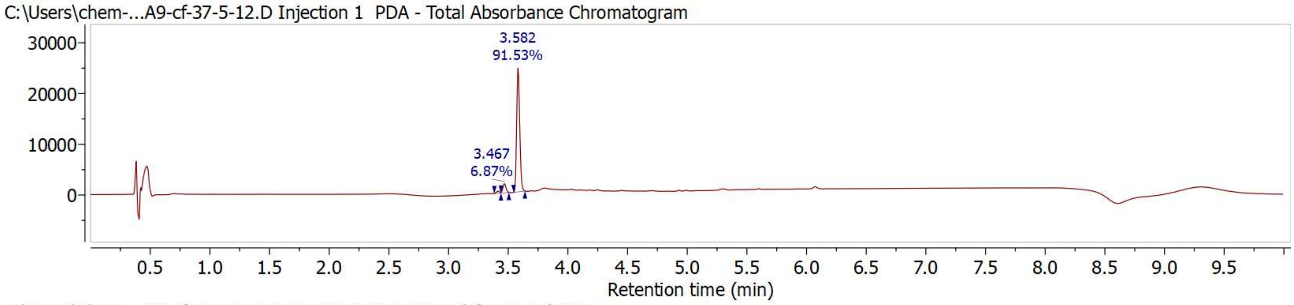

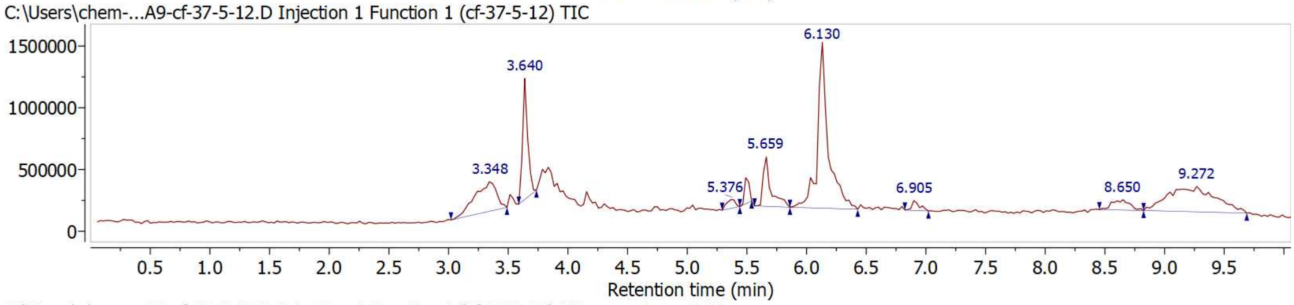

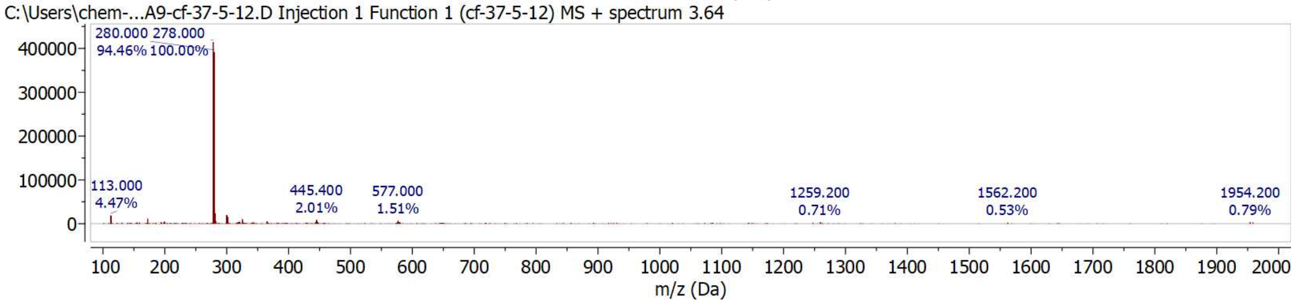

Tri-*tert*-butyl 2,2',2''-(10-(2-((1-methyl-2-nitro-1H-imidazol-5-yl)methoxy)-2-oxoethyl)-1,4,7,10-tetraazacyclododecane-1,4,7-triyl)triacetate, **9** – LCMS method 1

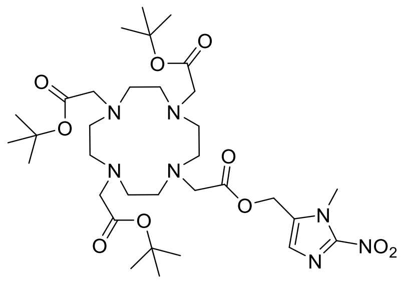

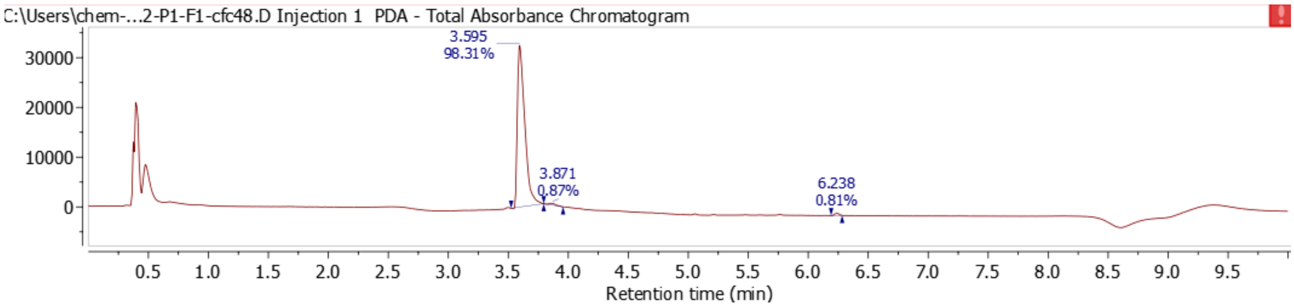

2,2',2''-(10-(2-((1-Methyl-2-nitro-1*H*-imidazol-5-yl)methoxy)-2-oxoethyl)-1,4,7,10-tetraazacyclododecane-1,4,7-triyl)triacetic acid, **11** – LCMS method 2

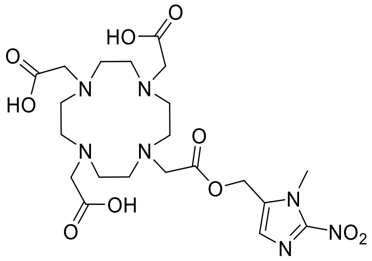

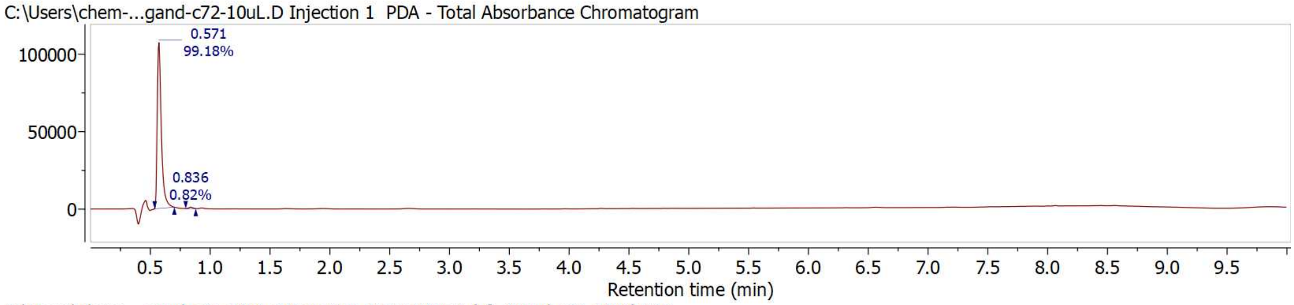

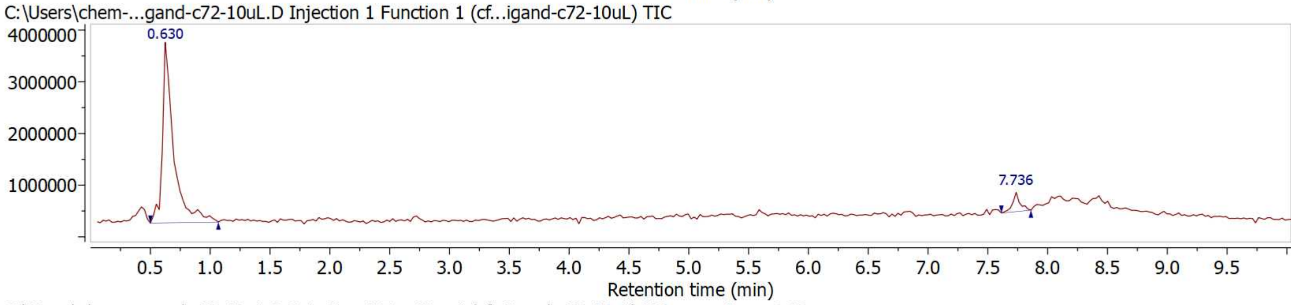

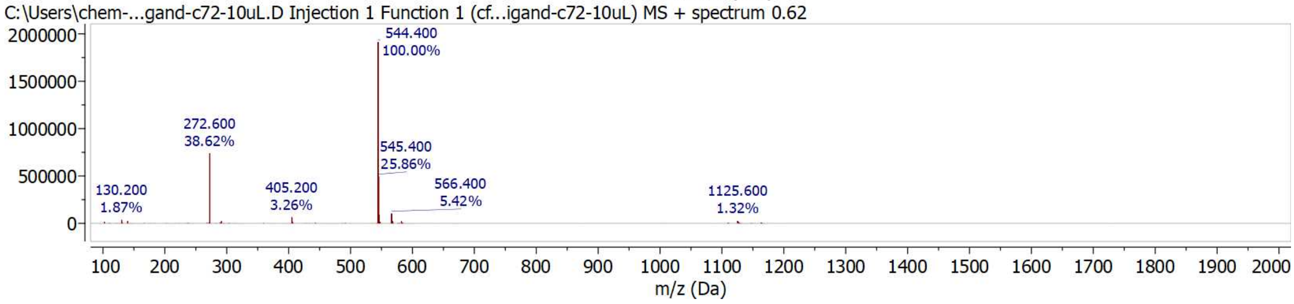

Tri-*tert*-butyl 2,2',2''-(10-(2-(benzyloxy)-2-oxoethyl)-1,4,7,10-tetraazacyclododecane-1,4,7-triyl)triacetate, **S4** – Analytical HPLC method B

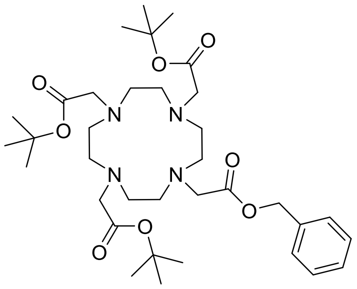

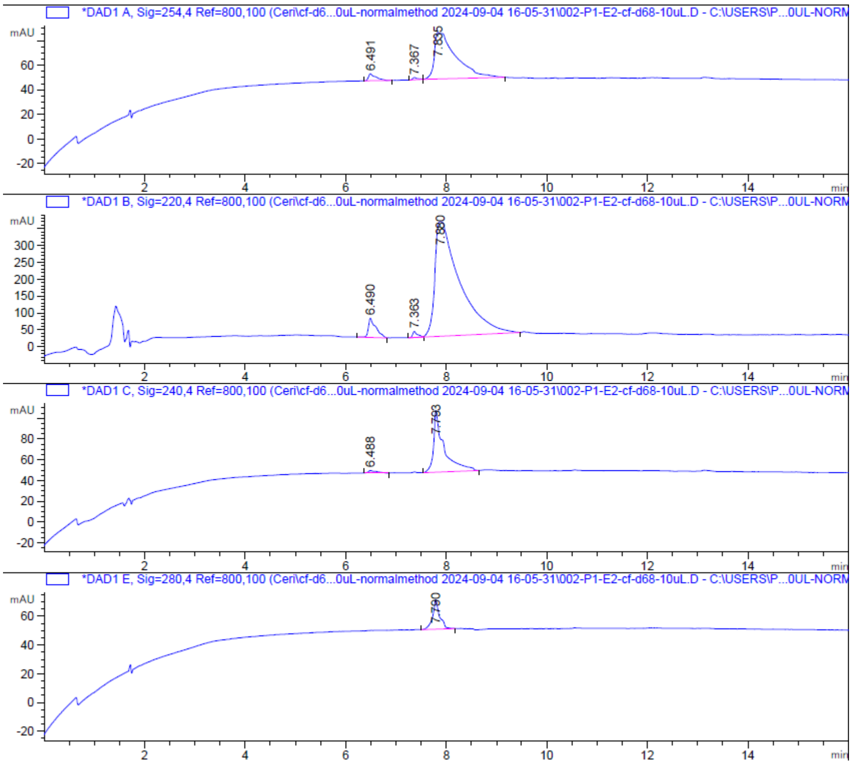

Signal 1: DAD1 A, Sig=254,4 Ref=800,100
Signal has been modified after loading from rawdata file!

Peak #	RetTime [min]	Type	Width [min]	Area [mAU*s]	Height [mAU]	Area %
1	6.491	BV R	0.1346	57.93521	5.64973	4.8358
2	7.367	BB	0.0906	11.61551	1.76897	0.9695
3	7.835	BB	0.3714	1128.50159	37.78876	94.1947
Totals :				1198.05231	45.20746	

Signal 2: DAD1 B, Sig=220,4 Ref=800,100
Signal has been modified after loading from rawdata file!

Peak #	RetTime [min]	Type	Width [min]	Area [mAU*s]	Height [mAU]	Area %
1	6.490	VB R	0.1382	591.11090	56.91418	4.7556
2	7.363	BV E	0.0899	118.21483	17.94540	0.9511
3	7.880	VV R	0.4392	1.17204e4	339.96286	94.2933
Totals :				1.24298e4	414.82244	

Signal 3: DAD1 C, Sig=240,4 Ref=800,100
Signal has been modified after loading from rawdata file!

Peak #	RetTime [min]	Type	Width [min]	Area [mAU*s]	Height [mAU]	Area %
1	6.488	BB	0.1487	28.01552	2.39183	3.1786
2	7.793	BB	0.1895	853.36548	59.42991	96.8214
Totals :				881.38100	61.82174	

Signal 4: DAD1 E, Sig=280,4 Ref=800,100
Signal has been modified after loading from rawdata file!

Peak #	RetTime [min]	Type	Width [min]	Area [mAU*s]	Height [mAU]	Area %
1	7.790	BB	0.1239	197.66843	20.60901	100.0000
Totals :				197.66843	20.60901	

2,2',2''-(10-(2-(benzyloxy)-2-oxoethyl)-1,4,7,10-tetraazacyclododecane-1,4,7-triyl)triacetic acid, **S5** – Analytical HPLC method A

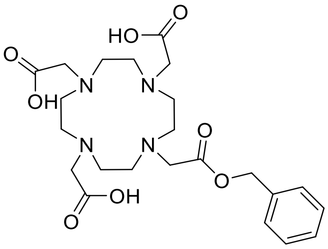

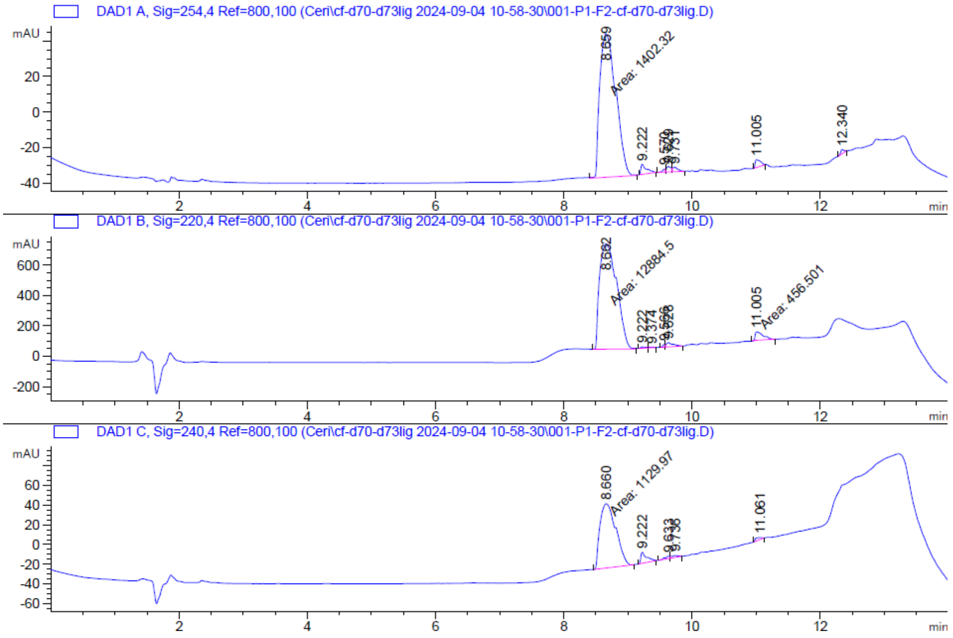

Signal 1: DAD1 A, Sig=254,4 Ref=800,100

Peak #	RetTime [min]	Type	Width [min]	Area [mAU*s]	Height [mAU]	Area %
1	8.659	MM	0.2896	1402.31934	80.69407	93.1688
2	9.222	BB	0.0832	35.23592	5.69580	2.3410
3	9.570	BV	0.0440	5.14081	1.76249	0.3416
4	9.629	VV	0.0689	15.37317	3.09774	1.0214
5	9.731	VB	0.0774	14.80011	2.52623	0.9833
6	11.005	BB	0.0712	24.01400	4.43028	1.5955
7	12.340	BB	0.0503	8.25489	2.33183	0.5484
Totals :				1505.13823	100.53844	

Signal 2: DAD1 B, Sig=220,4 Ref=800,100

Peak #	RetTime [min]	Type	Width [min]	Area [mAU*s]	Height [mAU]	Area %
1	8.662	MM	0.3098	1.28845e4	693.13135	94.3385
2	9.222	BV	0.0651	40.87700	9.15284	0.2993
3	9.374	VB	0.0744	18.32190	3.31853	0.1342
4	9.566	BV	0.0414	46.65302	17.30879	0.3416
5	9.628	VB	0.0980	210.87888	28.27490	1.5440
6	11.005	MM	0.1348	456.50125	56.45050	3.3424
Totals :				1.36577e4	807.63691	

Signal 3: DAD1 C, Sig=240,4 Ref=800,100

Peak #	RetTime [min]	Type	Width [min]	Area [mAU*s]	Height [mAU]	Area %
1	8.660	MM	0.2875	1129.97437	65.51482	90.9467
2	9.222	BB	0.0840	70.90374	11.32937	5.7067
3	9.633	BV	0.0709	12.38240	2.37123	0.9966
4	9.736	VB	0.0908	13.20370	1.90740	1.0627
5	11.061	BB	0.1025	15.99366	1.97435	1.2873
Totals :				1242.45787	83.09717	

Final compounds

Europium nitrobenzyl complex (EuNB) **1a** – LCMS method 2

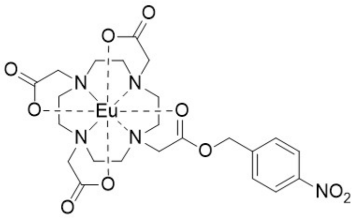

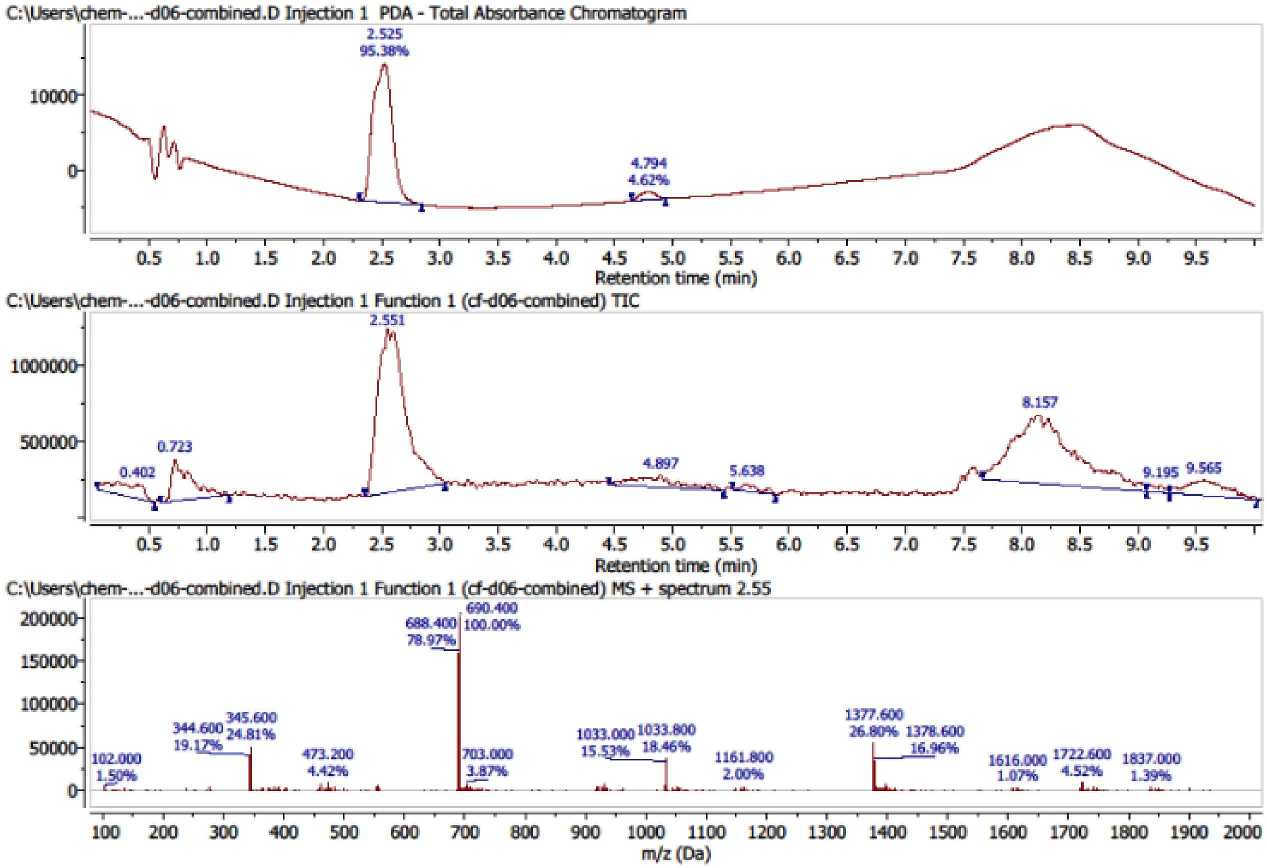

Europium nitrobenzyl complex (EuNB) **1a** – Analytical HPLC method A

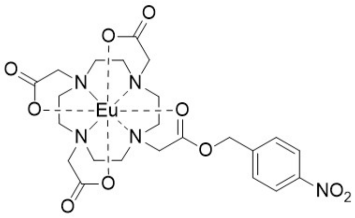

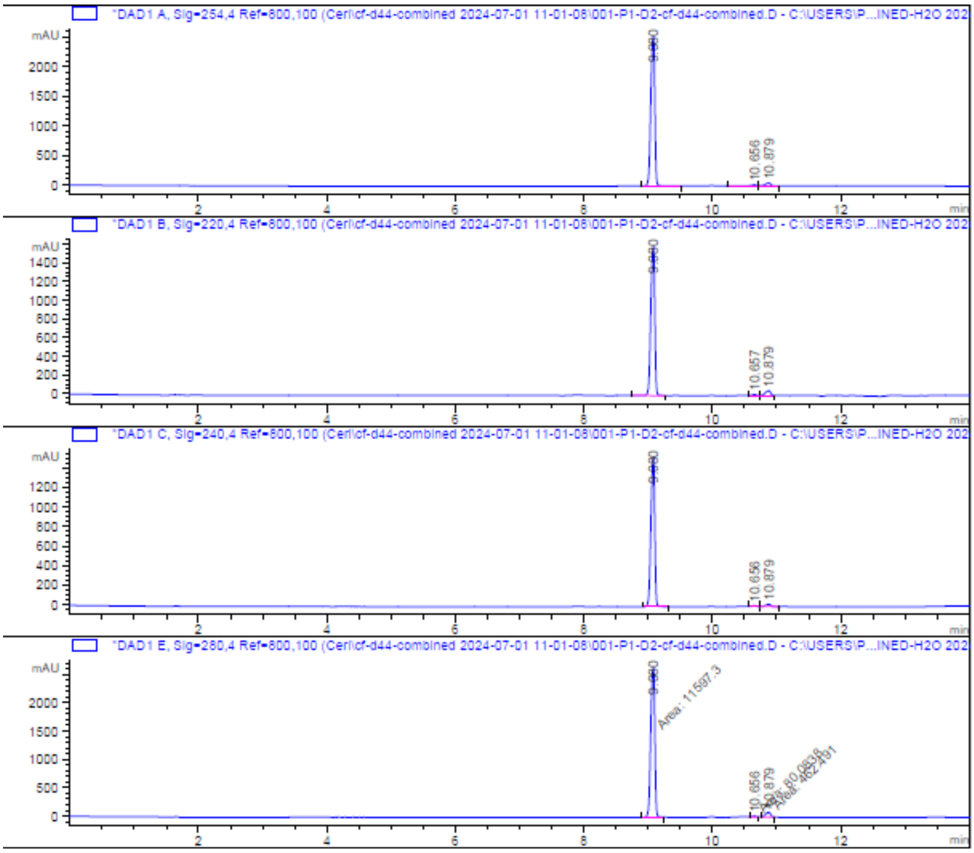

Signal 1: DAD1 A, Sig=254,4 Ref=800,100
Signal has been modified after loading from rawdata file!

Peak #	RetTime [min]	Type	Width [min]	Area [mAU*s]	Height [mAU]	Area %
1	9.080	BV R	0.0710	1.08583e4	2532.66089	96.5157
2	10.656	VV R	0.0834	114.38853	19.79097	1.0168
3	10.879	VV R	0.0953	277.60193	46.71708	2.4675

Totals : 1.12502e4 2599.16894

Signal 2: DAD1 B, Sig=220,4 Ref=800,100
Signal has been modified after loading from rawdata file!

Peak #	RetTime [min]	Type	Width [min]	Area [mAU*s]	Height [mAU]	Area %
1	9.080	VB R	0.0699	6814.35205	1593.28894	95.5534
2	10.657	BV	0.0549	40.13428	10.43367	0.5628
3	10.879	VB	0.0904	276.97504	50.14054	3.8838

Totals : 7131.46136 1653.86315

Signal 3: DAD1 C, Sig=240,4 Ref=800,100
Signal has been modified after loading from rawdata file!

Peak #	RetTime [min]	Type	Width [min]	Area [mAU*s]	Height [mAU]	Area %
1	9.080	BB	0.0678	6264.52979	1530.38879	97.4399
2	10.656	BV	0.0588	35.15849	9.57716	0.5469
3	10.879	VV R	0.0902	129.43283	23.15787	2.0132

Totals : 6429.12110 1563.12382

Signal 4: DAD1 E, Sig=280,4 Ref=800,100
Signal has been modified after loading from rawdata file!

Peak #	RetTime [min]	Type	Width [min]	Area [mAU*s]	Height [mAU]	Area %
1	9.080	MM	0.0732	1.15973e4	2641.97388	95.5306
2	10.656	MM	0.0703	80.08376	18.98098	0.6597
3	10.879	MM	0.0937	462.49091	82.28905	3.8097

Totals : 1.21398e4 2743.24391

Terbium nitrobenzyl complex (TbNB) **1b** – LCMS method 2

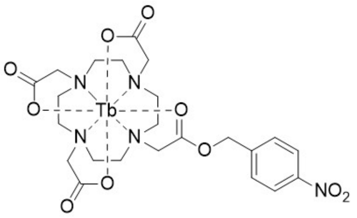

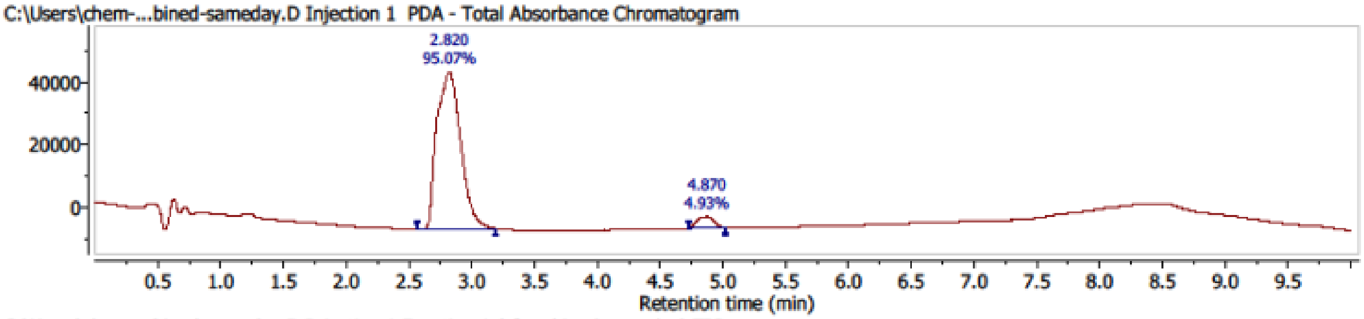

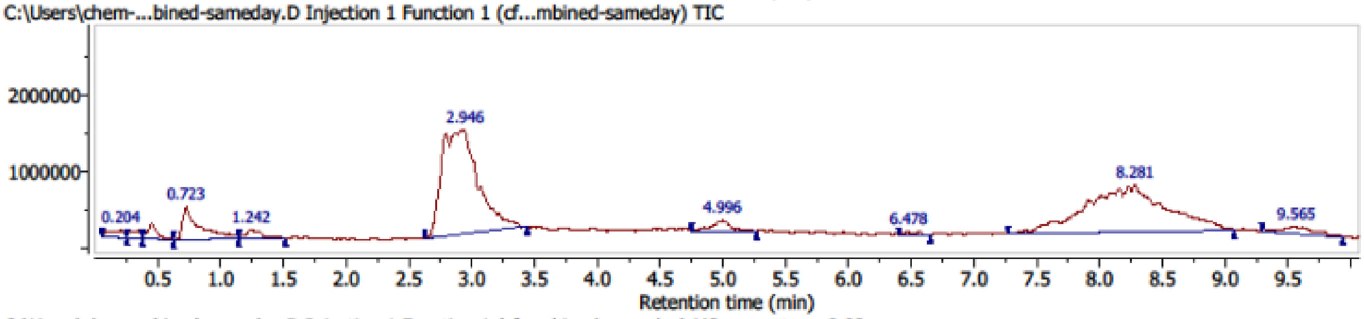

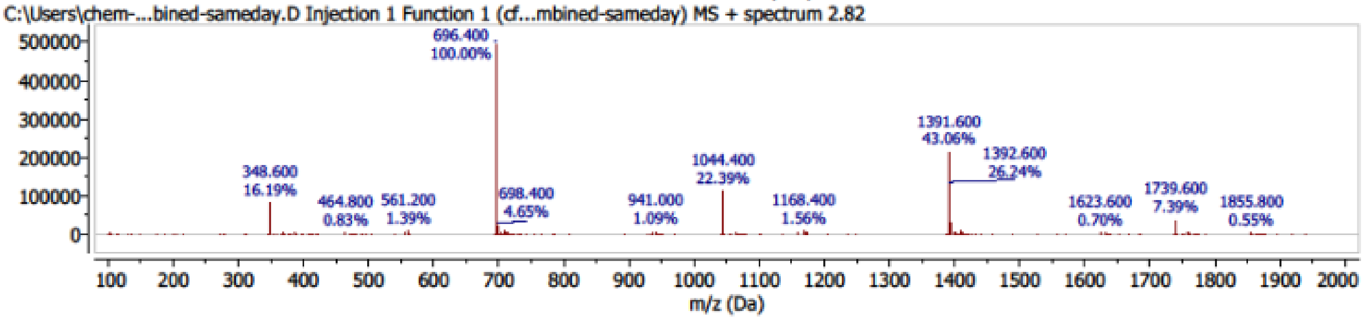

Terbium nitrobenzyl complex (TbNB) **1b** – Analytical HPLC method A

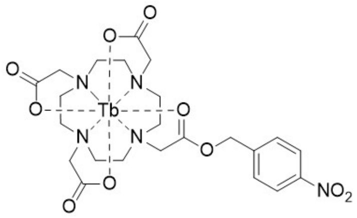

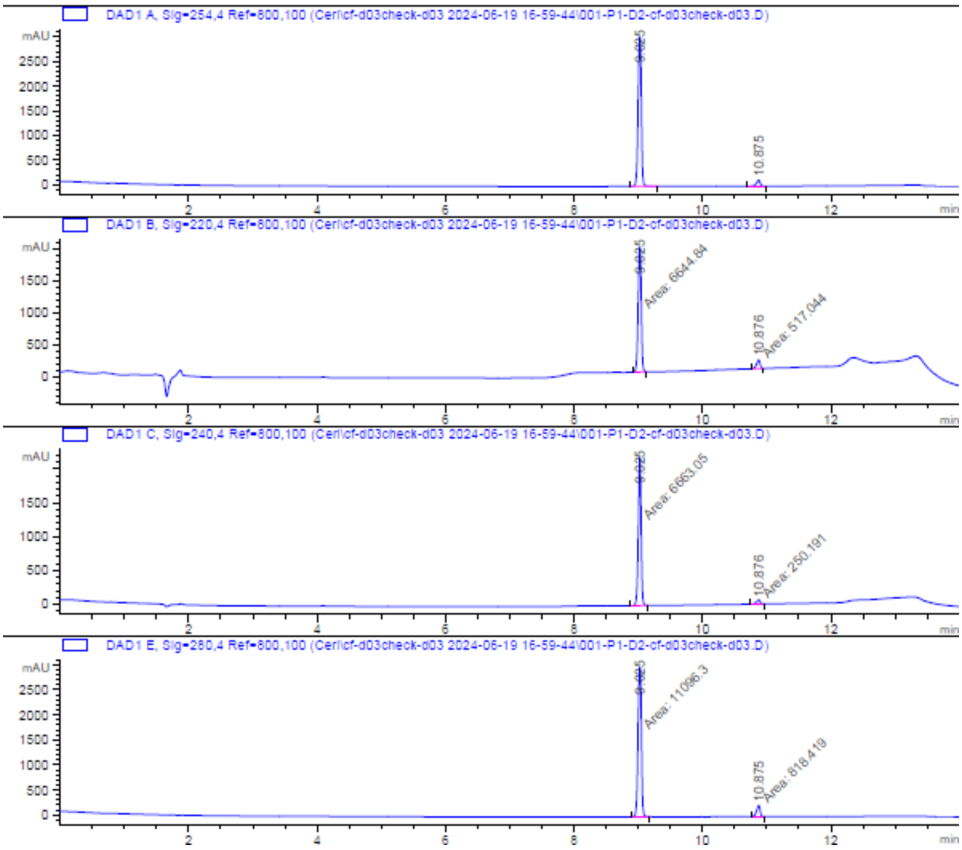

Signal 1: DAD1 A, Sig=254,4 Ref=800,100

Peak #	RetTime [min]	Type	Width [min]	Area [mAU*s]	Height [mAU]	Area %
1	9.025	BB	0.0548	1.06195e4	3035.96606	95.7272
2	10.875	BB	0.0586	474.00876	123.91591	4.2728
Totals :				1.10935e4	3159.88197	

Signal 2: DAD1 B, Sig=220,4 Ref=800,100

Peak #	RetTime [min]	Type	Width [min]	Area [mAU*s]	Height [mAU]	Area %
1	9.025	MM	0.0563	6644.84229	1965.71191	92.7806
2	10.876	MM	0.0635	517.04388	135.71552	7.2194
Totals :				7161.88617	2101.42743	

Signal 3: DAD1 C, Sig=240,4 Ref=800,100

Peak #	RetTime [min]	Type	Width [min]	Area [mAU*s]	Height [mAU]	Area %
1	9.025	MM	0.0504	6663.04590	2203.55713	96.3810
2	10.876	MM	0.0655	250.19104	63.66663	3.6190
Totals :				6913.23694	2267.22376	

Signal 4: DAD1 E, Sig=280,4 Ref=800,100

Peak #	RetTime [min]	Type	Width [min]	Area [mAU*s]	Height [mAU]	Area %
1	9.025	MM	0.0617	1.10963e4	2997.78491	93.1310
2	10.875	MM	0.0618	818.41882	220.74960	6.8690
Totals :				1.19147e4	3218.53452	

Gadolinium nitrobenzyl complex (GdNB) **1c** – LCMS method 2

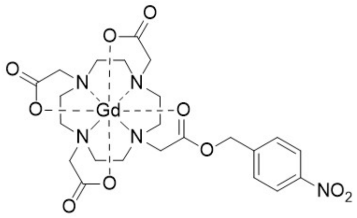

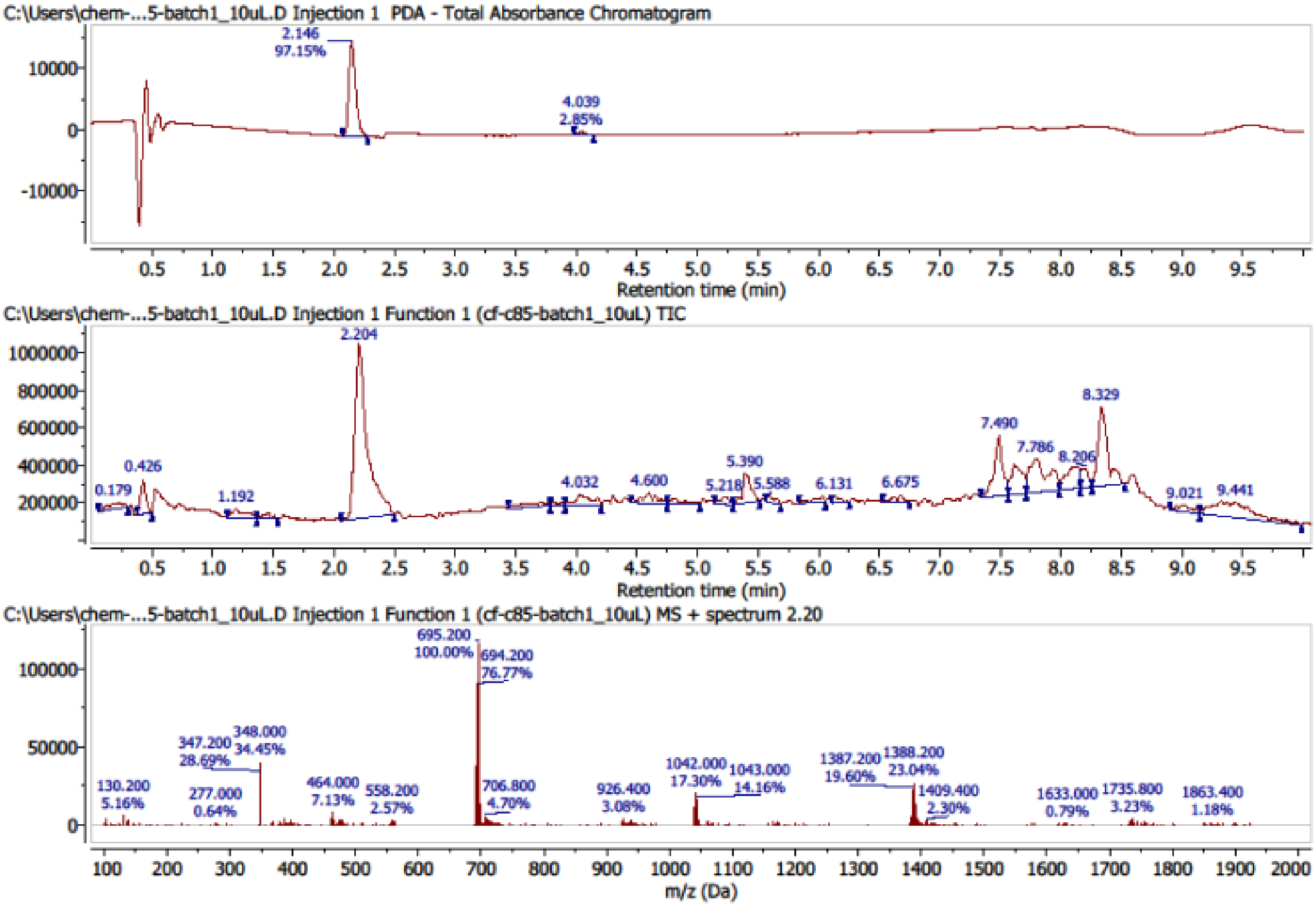

Gadolinium nitrobenzyl complex (GdNB) **1c** – Analytical HPLC method A

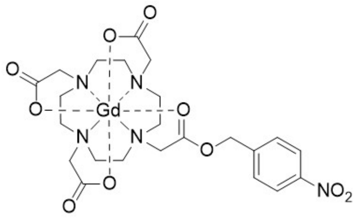

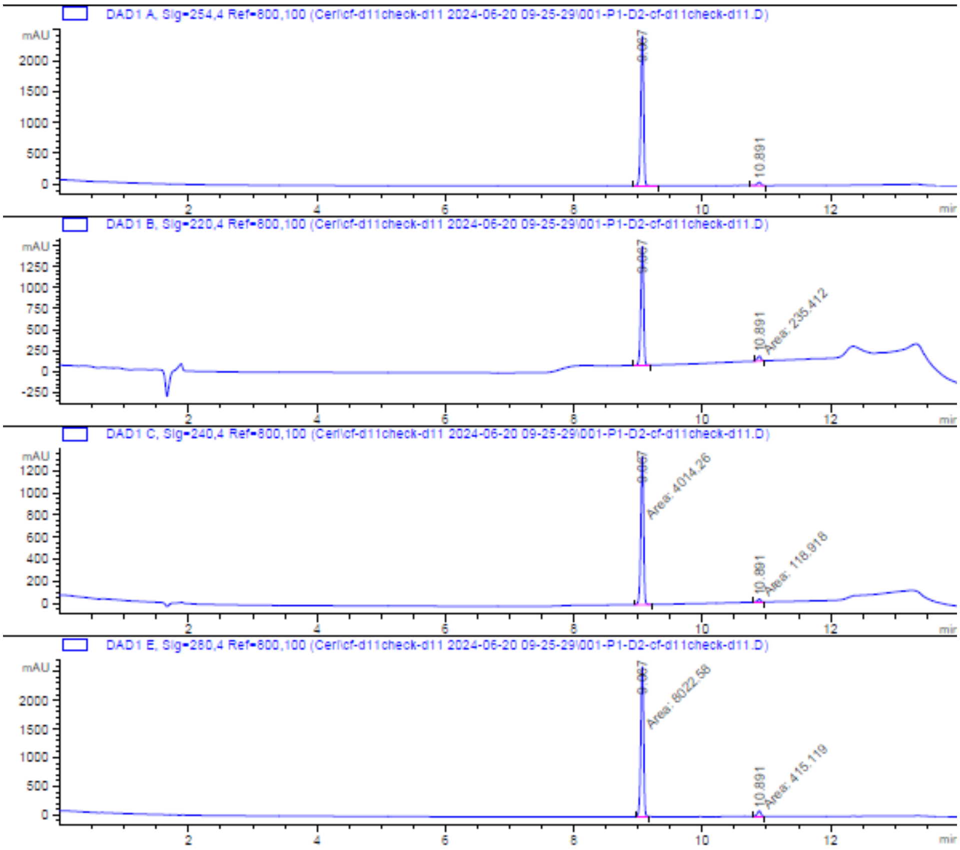

Signal 1: DAD1 A, Sig=254,4 Ref=800,100

Peak #	RetTime [min]	Type	Width [min]	Area [mAU*s]	Height [mAU]	Area %
1	9.067	BB	0.0474	7360.82080	2428.98340	97.1821
2	10.891	BB	0.0618	213.43527	53.21606	2.8179
Totals :				7574.25607	2482.19946	

Signal 2: DAD1 B, Sig=220,4 Ref=800,100

Peak #	RetTime [min]	Type	Width [min]	Area [mAU*s]	Height [mAU]	Area %
1	9.067	BB	0.0475	4317.02295	1419.23792	94.8289
2	10.891	MM	0.0669	235.41168	58.67155	5.1711
Totals :				4552.43463	1477.90946	

Signal 3: DAD1 C, Sig=240,4 Ref=800,100

Peak #	RetTime [min]	Type	Width [min]	Area [mAU*s]	Height [mAU]	Area %
1	9.067	MM	0.0497	4014.26343	1345.05127	97.1228
2	10.891	MM	0.0705	118.91805	28.12196	2.8772
Totals :				4133.18148	1373.17323	

Signal 4: DAD1 E, Sig=280,4 Ref=800,100

Peak #	RetTime [min]	Type	Width [min]	Area [mAU*s]	Height [mAU]	Area %
1	9.067	MM	0.0510	8022.57764	2623.73413	95.0802
2	10.891	MM	0.0697	415.11920	99.21050	4.9198
Totals :				8437.69684	2722.94463	

Europium nitroimidazole complex (EuNI) **2a** – LCMS method 2

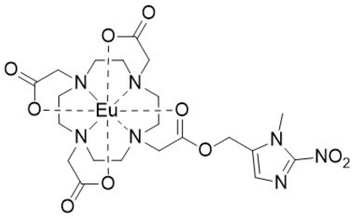

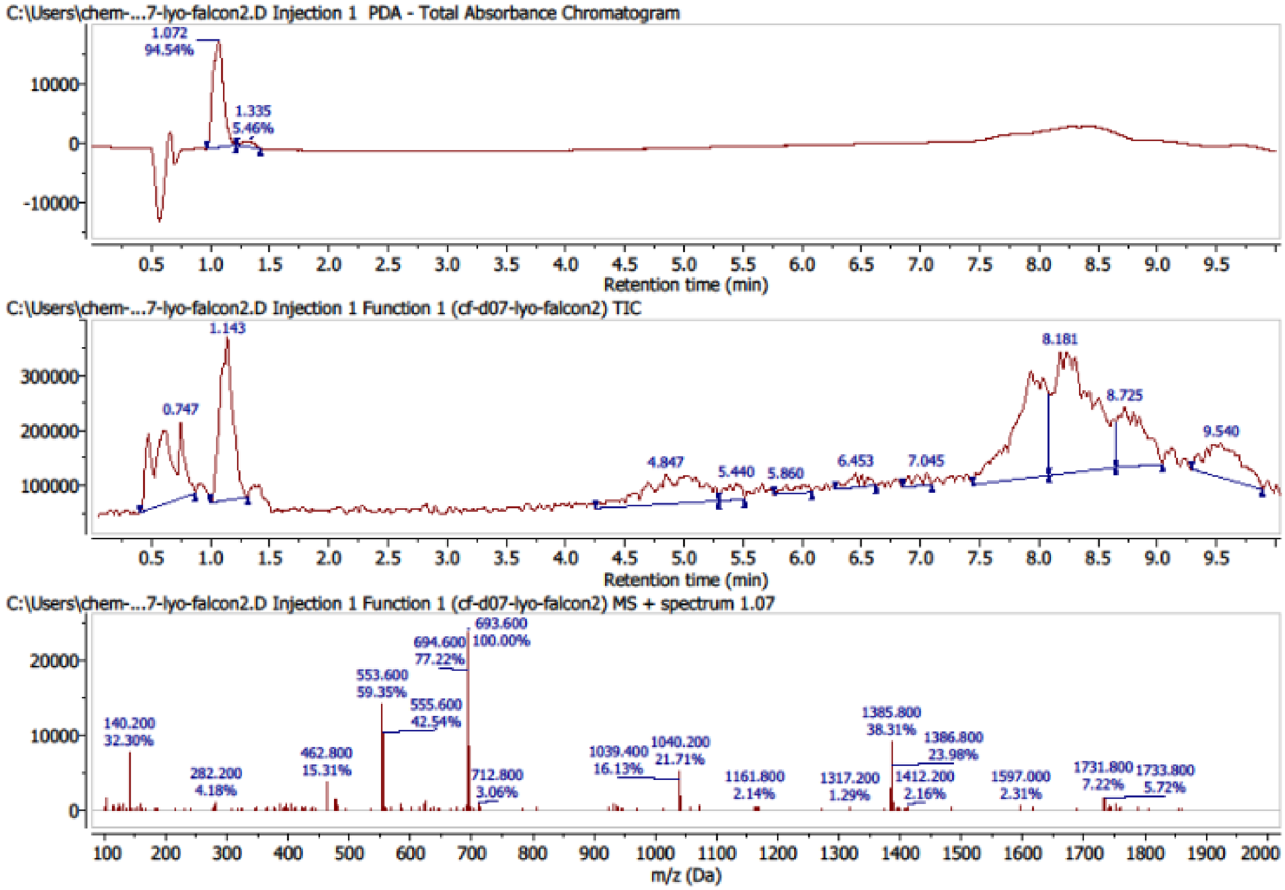

Europium nitroimidazole complex (EuNI) **2a** – Analytical HPLC method A

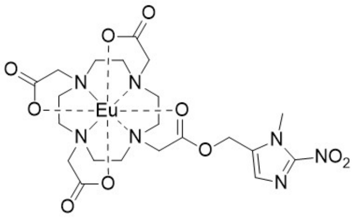

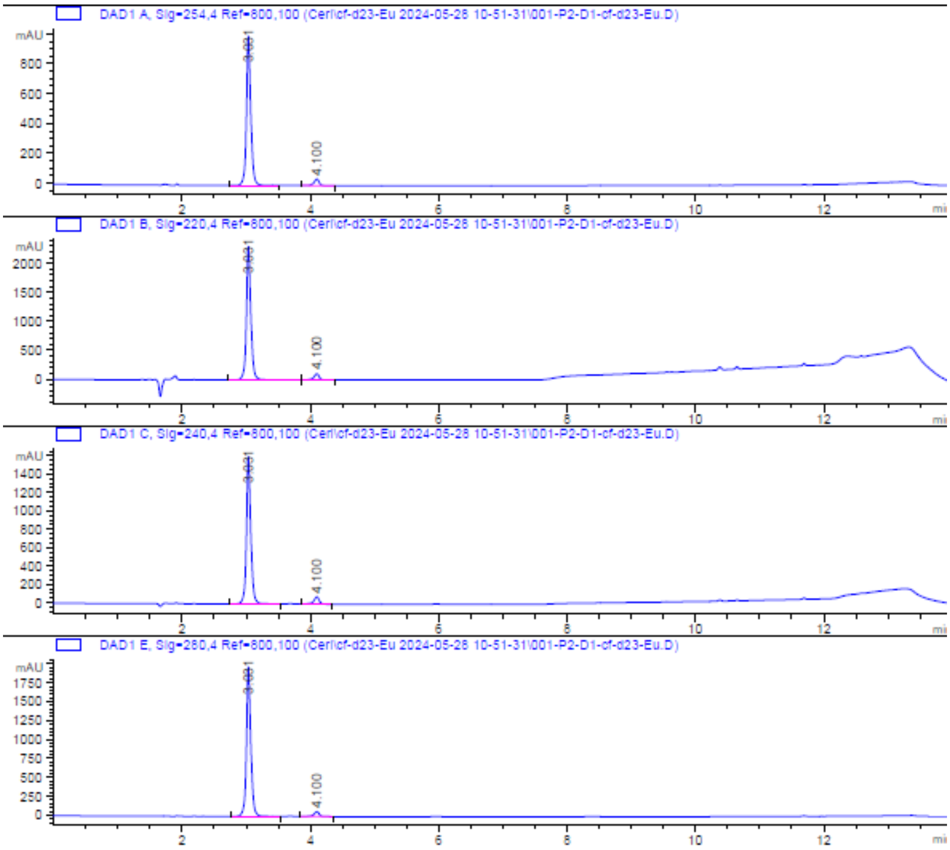

Signal 1: DAD1 A, Sig=254,4 Ref=800,100

Peak #	RetTime [min]	Type	Width [min]	Area [mAU*s]	Height [mAU]	Area %
1	3.031	BB	0.0690	4721.63525	1001.20709	94.9336
2	4.100	BB	0.0837	251.98569	44.73561	5.0664
Totals :				4973.62094	1045.94270	

Signal 2: DAD1 B, Sig=220,4 Ref=800,100

Peak #	RetTime [min]	Type	Width [min]	Area [mAU*s]	Height [mAU]	Area %
1	3.031	VV R	0.0712	1.11612e4	2308.98462	94.8664
2	4.100	BB	0.0835	603.97437	107.60862	5.1336
Totals :				1.17652e4	2416.59324	

Signal 3: DAD1 C, Sig=240,4 Ref=800,100

Peak #	RetTime [min]	Type	Width [min]	Area [mAU*s]	Height [mAU]	Area %
1	3.031	BB	0.0701	7590.58936	1606.93164	94.4154
2	4.100	BB	0.0835	448.97531	79.93547	5.5846
Totals :				8039.56467	1686.86711	

Signal 4: DAD1 E, Sig=280,4 Ref=800,100

Peak #	RetTime [min]	Type	Width [min]	Area [mAU*s]	Height [mAU]	Area %
1	3.031	BB	0.0705	9407.59473	1977.32581	96.2586
2	4.100	BB	0.0836	365.65176	65.05544	3.7414
Totals :				9773.24649	2042.38125	

Terbium nitroimidazole complex (TbNI) **2b** – LCMS method 2

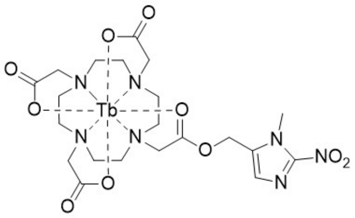

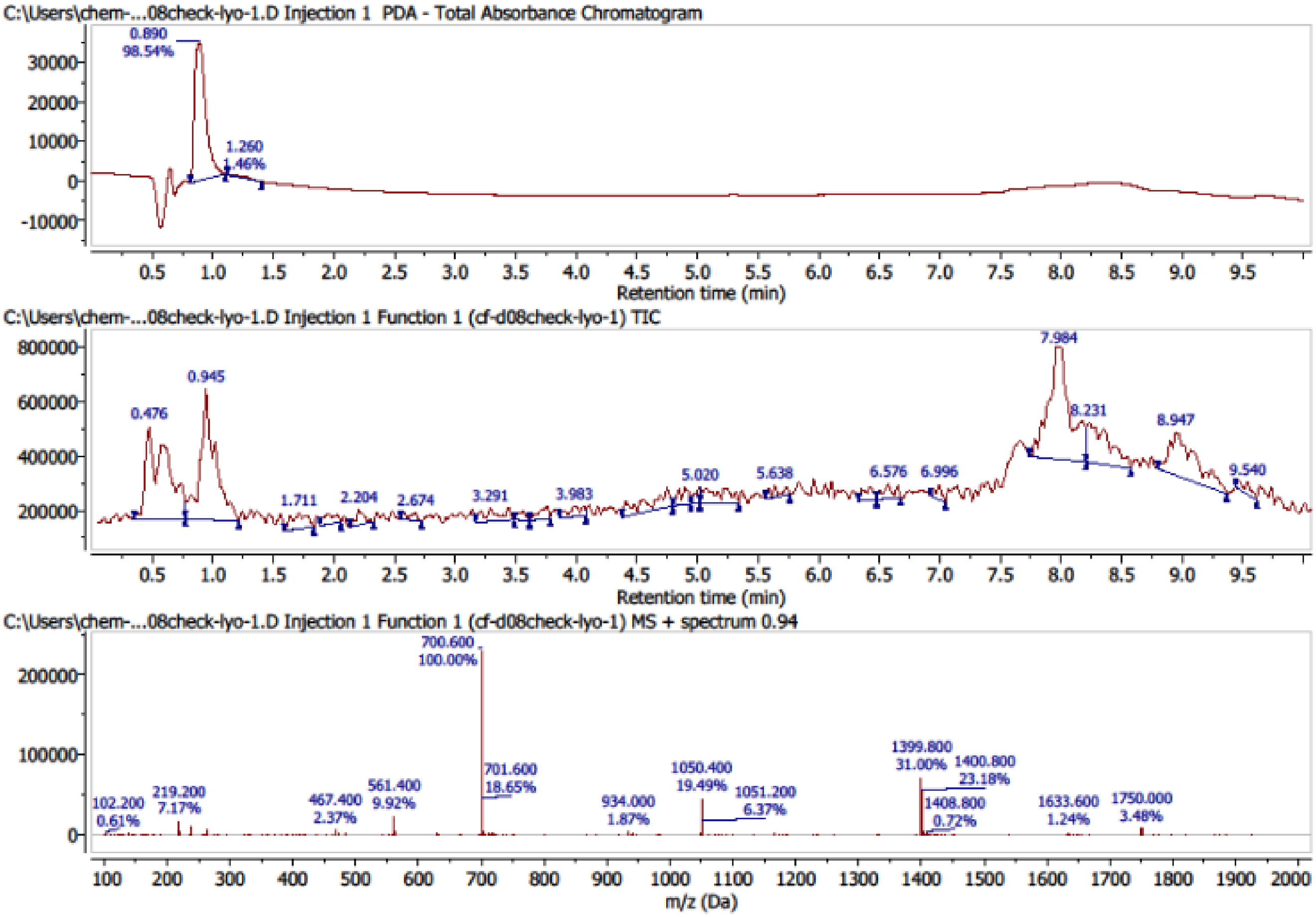

Terbium nitroimidazole complex (TbNI) **2b** – Analytical HPLC method A

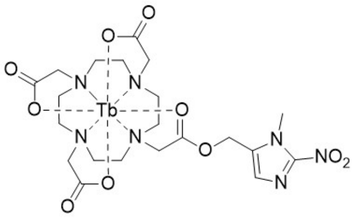

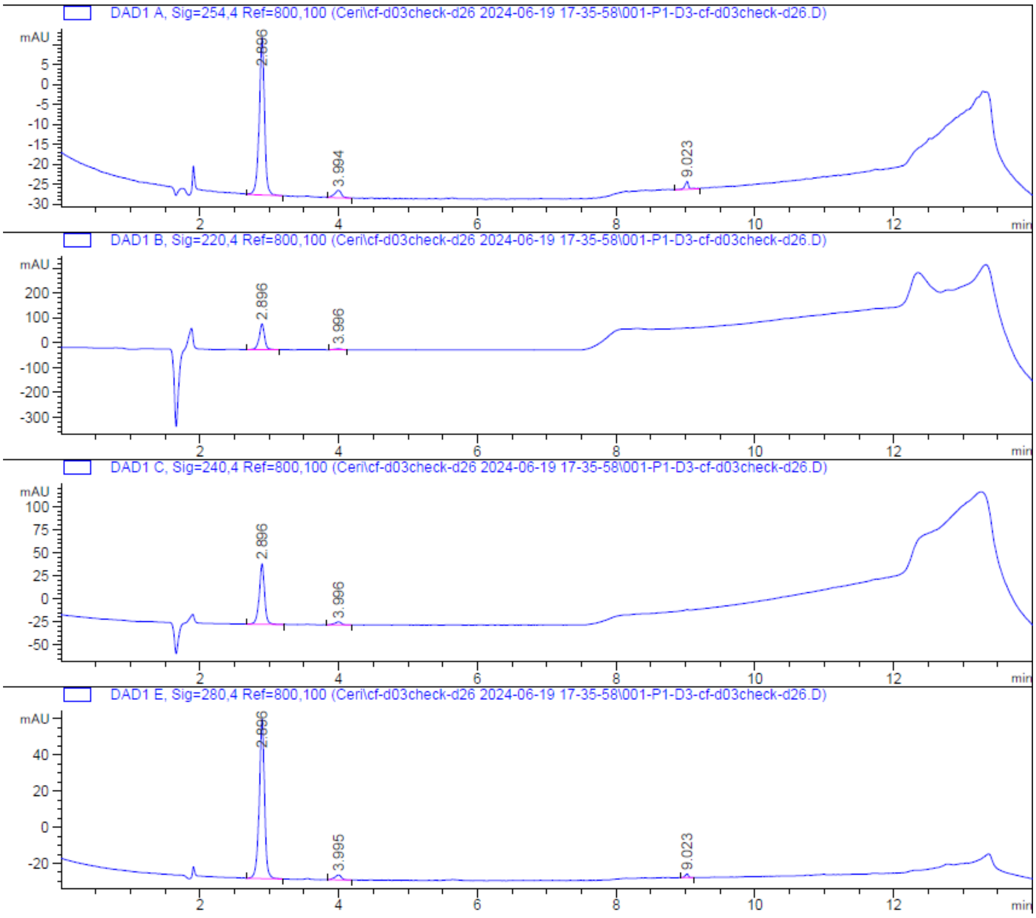

Signal 1: DAD1 A, Sig=254,4 Ref=800,100

Peak #	RetTime [min]	Type	Width [min]	Area [mAU*s]	Height [mAU]	Area %
1	2.896	BB	0.0794	211.72600	39.57319	90.7770
2	3.994	BB	0.1056	13.33914	1.86112	5.7191
3	9.023	BV R	0.0590	8.17242	1.98711	3.5039
Totals :				233.23755	43.42142	

Signal 2: DAD1 B, Sig=220,4 Ref=800,100

Peak #	RetTime [min]	Type	Width [min]	Area [mAU*s]	Height [mAU]	Area %
1	2.896	BV R	0.0790	553.90564	104.16051	95.6416
2	3.996	BB	0.0890	25.24172	4.03546	4.3584
Totals :				579.14736	108.19597	

Signal 3: DAD1 C, Sig=240,4 Ref=800,100

Peak #	RetTime [min]	Type	Width [min]	Area [mAU*s]	Height [mAU]	Area %
1	2.896	BB	0.0793	351.02808	65.72652	93.9577
2	3.996	BB	0.1041	22.57433	3.24765	6.0423
Totals :				373.60241	68.97418	

Signal 4: DAD1 E, Sig=280,4 Ref=800,100

Peak #	RetTime [min]	Type	Width [min]	Area [mAU*s]	Height [mAU]	Area %
1	2.896	BB	0.0791	470.40955	88.36967	94.9189
2	3.995	BB	0.1002	18.58730	2.67167	3.7505
3	9.023	BV R	0.0497	6.59406	2.03839	1.3305
Totals :				495.59091	93.07973	

Gadolinium nitroimidazole complex (GdNI) **2c** – LCMS method 2

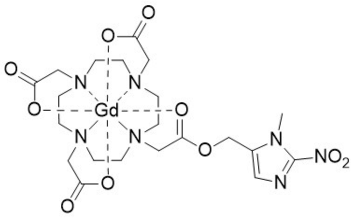

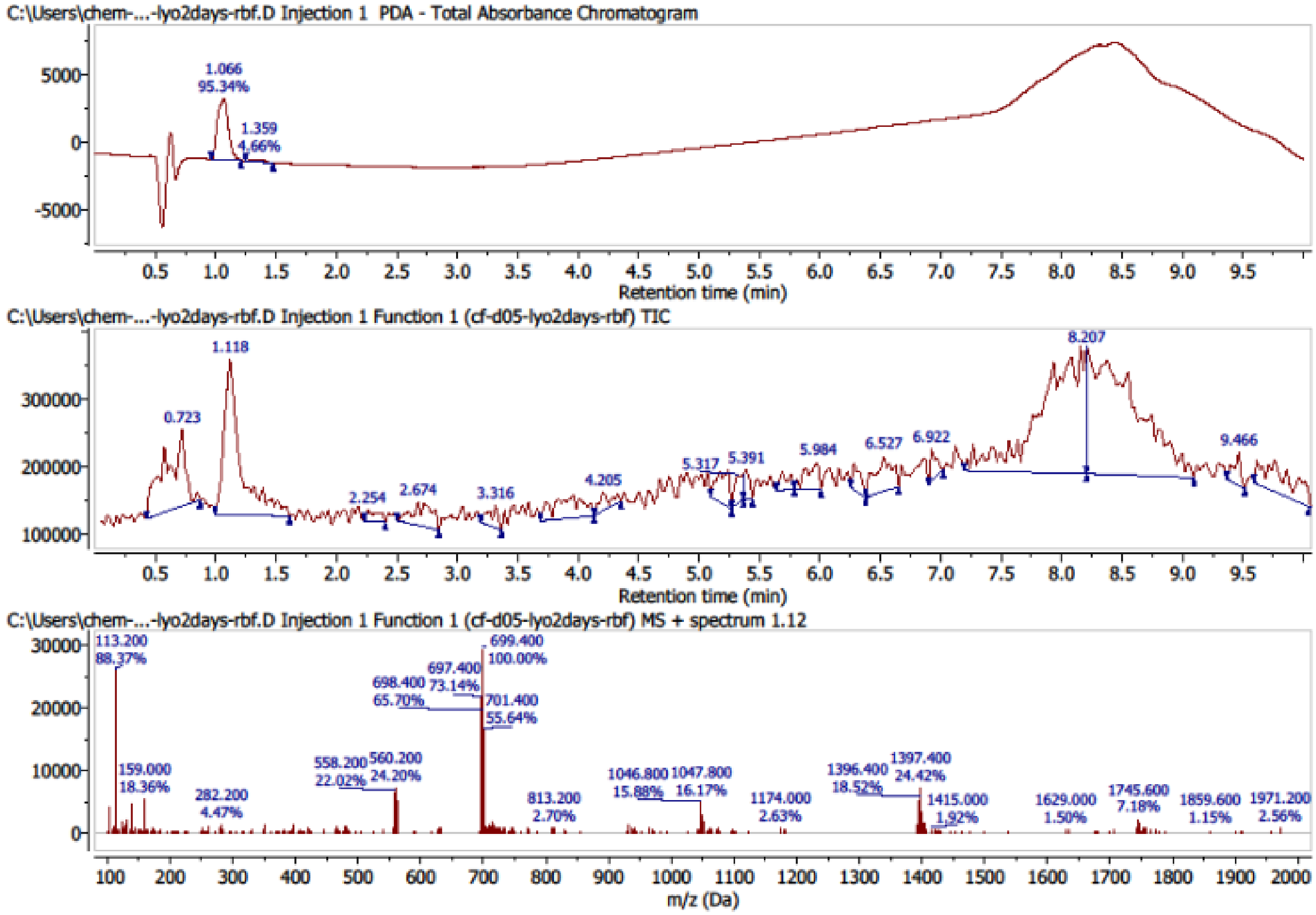

Gadolinium nitroimidazole complex (GdNI) **2c** – Analytical HPLC method A

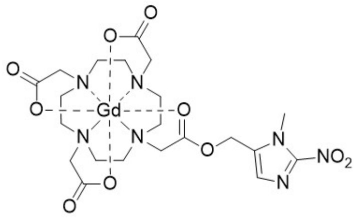

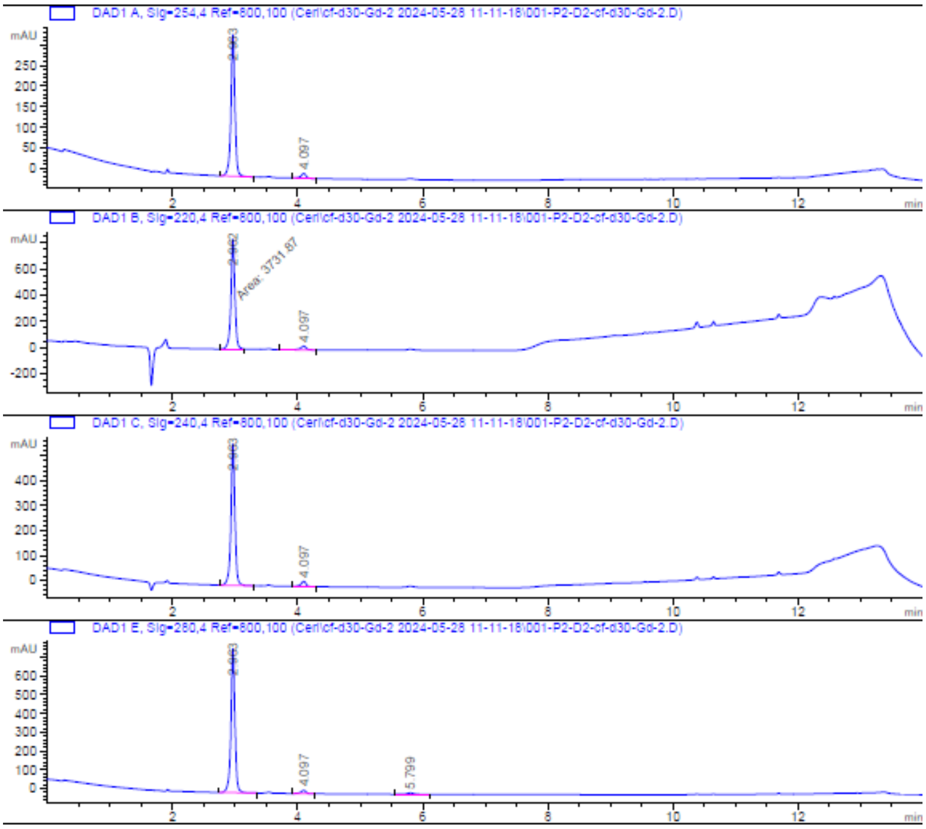

Signal 1: DAD1 A, Sig=254,4 Ref=800,100

Peak #	RetTime [min]	Type	Width [min]	Area [mAU*s]	Height [mAU]	Area %
1	2.963	BB	0.0659	1530.90210	344.14487	95.9053
2	4.097	BB	0.0817	65.36169	11.79296	4.0947
Totals :				1596.26379	355.93783	

Signal 2: DAD1 B, Sig=220,4 Ref=800,100

Peak #	RetTime [min]	Type	Width [min]	Area [mAU*s]	Height [mAU]	Area %
1	2.962	MM	0.0739	3731.86816	841.92188	95.2367
2	4.097	BB	0.0916	186.65054	29.19338	4.7633
Totals :				3918.51871	871.11526	

Signal 3: DAD1 C, Sig=240,4 Ref=800,100

Peak #	RetTime [min]	Type	Width [min]	Area [mAU*s]	Height [mAU]	Area %
1	2.963	BB	0.0659	2522.19189	566.84747	95.5290
2	4.097	BB	0.0833	118.04552	21.08798	4.4710
Totals :				2640.23741	587.93546	

Signal 4: DAD1 E, Sig=280,4 Ref=800,100

Peak #	RetTime [min]	Type	Width [min]	Area [mAU*s]	Height [mAU]	Area %
1	2.963	BB	0.0660	3416.86890	765.90320	95.6125
2	4.097	BB	0.0832	95.88809	17.14798	2.6832
3	5.799	BV R	0.1181	60.90603	7.24036	1.7043
Totals :				3573.66302	790.29154	

Europium benzyl complex (EuBn) **4a** – Analytical HPLC method A

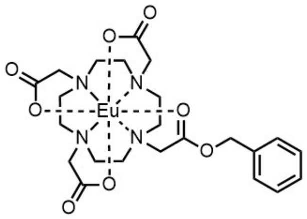

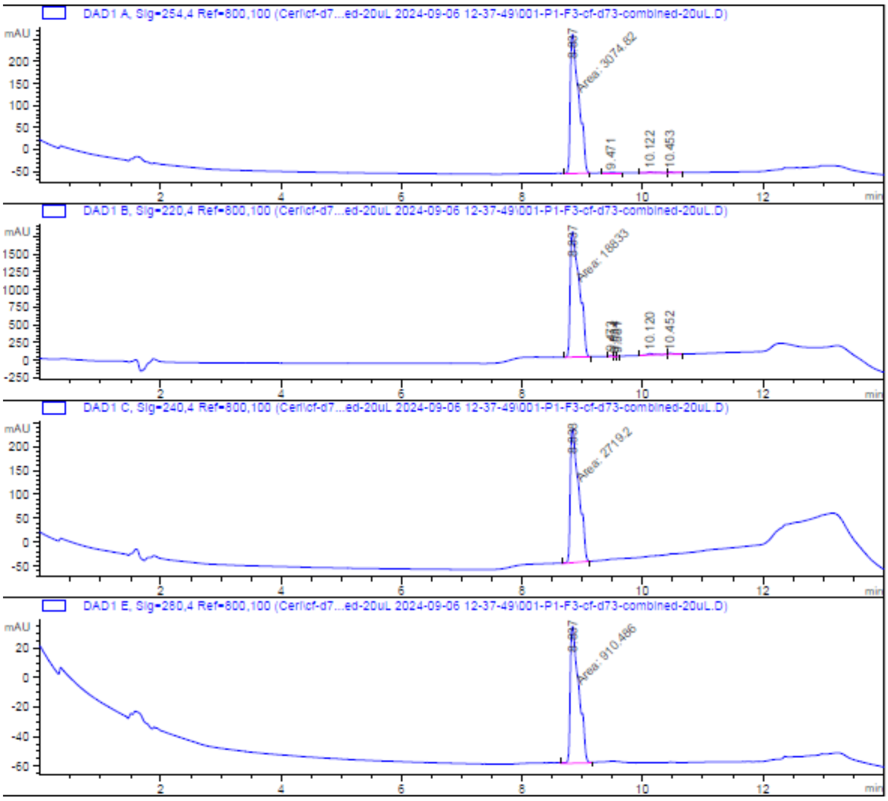

Signal 1: DAD1 A, Sig=254,4 Ref=800,100

Peak #	RetTime [min]	Type	Width [min]	Area [mAU*s]	Height [mAU]	Area %
1	8.837	MM	0.1619	3074.82300	316.51300	98.6913
2	9.471	BB	0.1046	9.25713	1.12971	0.2971
3	10.122	BV	0.1345	20.59275	2.06152	0.6610
4	10.453	VB	0.0877	10.92334	1.72970	0.3506
Totals :				3115.59621	321.43392	

Signal 2: DAD1 B, Sig=220,4 Ref=800,100

Peak #	RetTime [min]	Type	Width [min]	Area [mAU*s]	Height [mAU]	Area %
1	8.837	MM	0.1762	1.88330e4	1781.68335	98.2755
2	9.473	BV	0.0525	27.63516	7.57831	0.1442
3	9.534	VV	0.0239	5.27953	3.25249	0.0275
4	9.581	VV	0.0461	7.68636	2.23390	0.0401
5	10.120	BV R	0.1237	192.34634	21.03777	1.0037
6	10.452	BV R	0.0802	97.52488	16.69501	0.5089

Signal 3: DAD1 C, Sig=240,4 Ref=800,100

Peak #	RetTime [min]	Type	Width [min]	Area [mAU*s]	Height [mAU]	Area %
1	8.838	MM	0.1622	2719.19775	279.45468	100.0000
Totals :				2719.19775	279.45468	

Signal 4: DAD1 E, Sig=280,4 Ref=800,100

Peak #	RetTime [min]	Type	Width [min]	Area [mAU*s]	Height [mAU]	Area %
1	8.837	MM	0.1634	910.48566	92.86311	100.0000
Totals :				910.48566	92.86311	

Terbium benzyl complex (TbBn) **4b** – Analytical HPLC method A

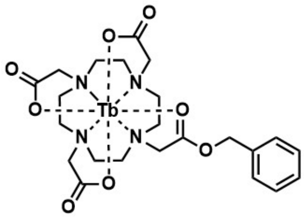

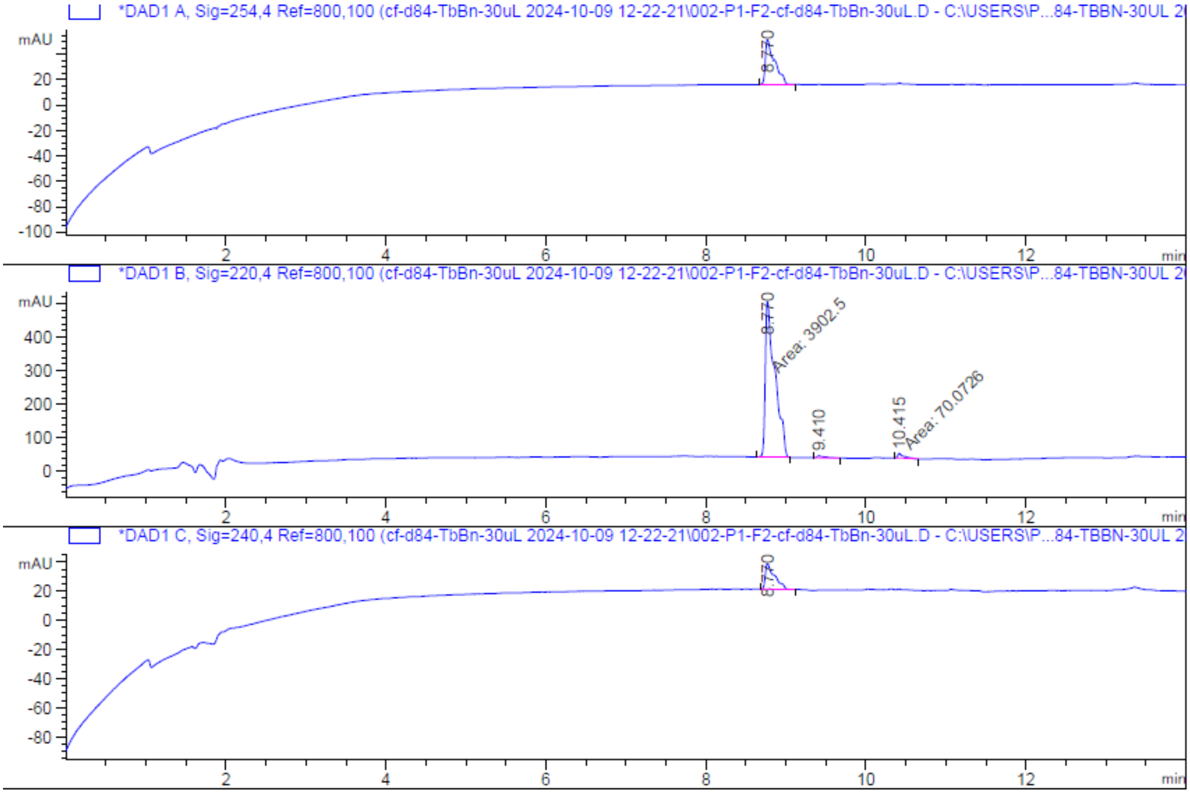

Signal 1: DAD1 A, Sig=254,4 Ref=800,100
Signal has been modified after loading from rawdata file!

Peak #	RetTime [min]	Type	Width [min]	Area [mAU*s]	Height [mAU]	Area %
1	8.770	BB	0.1085	281.87323	35.18276	100.0000
Totals :				281.87323	35.18276	

Signal 2: DAD1 B, Sig=220,4 Ref=800,100
Signal has been modified after loading from rawdata file!

Peak #	RetTime [min]	Type	Width [min]	Area [mAU*s]	Height [mAU]	Area %
1	8.770	MM	0.1398	3902.50146	465.27966	97.2110
2	9.410	BV R	0.0884	41.89270	6.31290	1.0435
3	10.415	MM	0.0865	70.07259	13.49830	1.7455
Totals :				4014.46675	485.09086	

Signal 3: DAD1 C, Sig=240,4 Ref=800,100
Signal has been modified after loading from rawdata file!

Peak #	RetTime [min]	Type	Width [min]	Area [mAU*s]	Height [mAU]	Area %
1	8.770	BB	0.1087	140.56416	17.50330	100.0000
Totals :				140.56416	17.50330	

References

(1) Calder, E. D. D.; Skwarska, A.; Sneddon, D.; Folkes, L. K.; Mistry, I. N.; Conway, S. J.; Hammond, E. M. Hypoxia-activated pro-drugs of the KDAC inhibitor vorinostat (SAHA). *Tetrahedron* **2020**, *76* (21), 131170. DOI: 10.1016/j.tet.2020.131170.
(2) O'Connor, L. J.; Cazares-Körner, C.; Saha, J.; Evans, C. N. G.; Stratford, M. R. L.; Hammond, E. M.; Conway, S. J. Efficient synthesis of 2-nitroimidazole derivatives and the bioreductive clinical candidate Evofosfamide (TH-302). *Organic Chemistry Frontiers* **2015**, *2* (9), 1026-1029, 10.1039/C5QO00211G. DOI: 10.1039/C5QO00211G.
(3) Junker, A. K. R.; Tropiano, M.; Faulkner, S.; Sørensen, T. J. Kinetically Inert Lanthanide Complexes as Reporter Groups for Binding of Potassium by 18-crown-6. *Inorganic Chemistry* **2016**, *55* (23), 12299-12308. DOI: 10.1021/acs.inorgchem.6b02063.
(4) Zhang, S.; Kovacs, Z.; Burgess, S.; Aime, S.; Terreno, E.; Sherry, A. D. {DOTA-bis(amide)}lanthanide Complexes: NMR Evidence for Differences in Water-Molecule Exchange Rates for Coordination Isomers. *Chemistry – A European Journal* **2001**, *7* (1), 288-296. DOI: 10.1002/1521-3765(20010105)7:1<288::AID-CHEM288>3.0.CO;2-6 (acccessed 2024/03/02).
(5) Binnemans, K. Interpretation of europium(III) spectra. *Coordination Chemistry Reviews* **2015**, *295*, 1-45. DOI: 10.1016/j.ccr.2015.02.015.
(6) Medina-Velazquez, D. Y.; Caldiño, U.; Morales-Ramirez, A.; Reyes-Miranda, J.; Lopez, R. E.; Escudero, R.; Ruiz-Guerrero, R.; Morales Perez, M. F. Synthesis of luminescent terbium-thenoyltriflouroacetone MOF nanorods for green laser application. *Optical Materials* **2019**, *87*, 3-10. DOI: 10.1016/j.optmat.2018.08.021.
(7) Mara, M. W.; Tatum, D. S.; March, A.-M.; Doumy, G.; Moore, E. G.; Raymond, K. N. Energy Transfer from Antenna Ligand to Europium(III) Followed Using Ultrafast Optical and X-ray Spectroscopy. *Journal of the American Chemical Society* **2019**, *141* (28), 11071-11081. DOI: 10.1021/jacs.9b02792.
(8) Kofod, N.; Nielsen, L. G.; Sørensen, T. J. Temperature Dependence of Fundamental Photophysical Properties of [Eu(MeOH-d4)9]3+ Solvates and [Eu·DOTA(MeOH-d4)]− Complexes. *The Journal of Physical Chemistry A* **2021**, *125* (38), 8347-8357. DOI: 10.1021/acs.jpca.1c04994.
(9) Horrocks, W. D., Jr.; Sudnick, D. R. Lanthanide ion probes of structure in biology. Laser-induced luminescence decay constants provide a direct measure of the number of metal-coordinated water molecules. *Journal of the American Chemical Society* **1979**, *101* (2), 334-340. DOI: 10.1021/ja00496a010.
(10) Beeby, A.; M. Clarkson, I.; S. Dickins, R.; Faulkner, S.; Parker, D.; Royle, L.; S. de Sousa, A.; A. Gareth Williams, J.; Woods, M. Non-radiative deactivation of the excited states of europium, terbium and ytterbium complexes by proximate energy-matched OH, NH and CH oscillators: an improved luminescence method for establishing solution hydration states. *Journal of the Chemical Society, Perkin Transactions 2* **1999**, (3), 493-504, 10.1039/A808692C. DOI: 10.1039/A808692C.
(11) Wahsner, J.; Gale, E. M.; Rodríguez-Rodríguez, A.; Caravan, P. Chemistry of MRI Contrast Agents: Current Challenges and New Frontiers. *Chemical Reviews* **2019**, *119* (2), 957-1057. DOI: 10.1021/acs.chemrev.8b00363.
(12) Caravan, P.; Esteban-Gómez, D.; Rodríguez-Rodríguez, A.; Platas-Iglesias, C. Water exchange in lanthanide complexes for MRI applications. Lessons learned over the last 25 years. *Dalton Transactions* **2019**, *48* (30), 11161-11180, 10.1039/C9DT01948K. DOI: 10.1039/C9DT01948K.
(13) Geraldes, C. F. G. C.; Peters, J. A. MRI Contrast Agents in Glycobiology. In *Molecules*, 2022; Vol. 27.
(14) O'Connor, L. J.; Mistry, I. N.; Collins, S. L.; Folkes, L. K.; Brown, G.; Conway, S. J.; Hammond, E. M. CYP450 Enzymes Effect Oxygen-Dependent Reduction of Azide-Based Fluorogenic Dyes. *ACS Central Science* **2017**, *3* (1), 20-30. DOI: 10.1021/acscentsci.6b00276.
(15) Sokolova, D.; Lurshay, T. C.; Rowbotham, J. S.; Stonadge, G.; Reeve, H. A.; Cleary, S. E.; Sudmeier, T.; Vincent, K. A. Selective hydrogenation of nitro compounds to amines by coupled redox reactions over a heterogeneous biocatalyst. *Nature Communications* **2024**, *15* (1), 7297. DOI: 10.1038/s41467-024-51531-2.
(16) Fulmer, G. R.; Miller, A. J. M.; Sherden, N. H.; Gottlieb, H. E.; Nudelman, A.; Stoltz, B. M.; Bercaw, J. E.; Goldberg, K. I. NMR Chemical Shifts of Trace Impurities: Common Laboratory Solvents, Organics, and Gases in Deuterated Solvents Relevant to the Organometallic Chemist. *Organometallics* **2010**, *29* (9), 2176-2179. DOI: 10.1021/om100106e.

(17) Rodriguez-Maciá, P.; Dutta, A.; Lubitz, W.; Shaw, W. J.; Rüdiger, O. Direct Comparison of the Performance of a Bio-inspired Synthetic Nickel Catalyst and a [NiFe]-Hydrogenase, Both Covalently Attached to Electrodes. *Angewandte Chemie International Edition* **2015**, *54* (42), 12303-12307. DOI: 10.1002/anie.201502364.
(18) Liu, Y.; Zhang, L.; Nazare, M.; Yao, Q.; Hu, H.-Y. A novel nitroreductase-enhanced MRI contrast agent and its potential application in bacterial imaging. *Acta Pharmaceutica Sinica B* **2018**, *8* (3), 401-408. DOI: 10.1016/j.apsb.2017.11.001.
(19) Wan, F.; Liu, M.; Zhang, J.; Li, Y.; Jiang, L. Synthesis and characterization of DOTA-mono-adamantan-1-ylamide. *Research on Chemical Intermediates* **2015**, *41* (8), 5109-5119. DOI: 10.1007/s11164-014-1615-8.
(20) Jenie, S. N. A.; Hickey, S. M.; Du, Z.; Sebben, D.; Brooks, D. A.; Voelcker, N. H.; Plush, S. E. A europium-based 'off-on' colourimetric detector of singlet oxygen. *Inorganica Chimica Acta* **2017**, *462*, 236-240. DOI: 10.1016/j.ica.2017.03.043.
(21) Jagadish, B.; Brickert-Albrecht, G. L.; Nichol, G. S.; Mash, E. A.; Raghunand, N. On the synthesis of 1,4,7-tris(tert-butoxycarbonylmethyl)-1,4,7,10-tetraazacyclododecane. *Tetrahedron Letters* **2011**, *52* (17), 2058-2061. DOI: 10.1016/j.tetlet.2010.10.074.
(22) Dadabhoy, A.; Faulkner, S.; Sammes, P. G. Long wavelength sensitizers for europium(iii) luminescence based on acridone derivatives. *Journal of the Chemical Society, Perkin Transactions 2* **2002**, (2), 348-357, 10.1039/B104541P. DOI: 10.1039/B104541P.
(23) Mizukami, S.; Tonai, K.; Kaneko, M.; Kikuchi, K. Lanthanide-Based Protease Activity Sensors for Time-Resolved Fluorescence Measurements. *Journal of the American Chemical Society* **2008**, *130* (44), 14376-14377. DOI: 10.1021/ja800322b.
(24) Rufer, C.; Kessler, H. J.; Schroeder, E. Chemotherapeutic nitroheterocycles. 6. Substituted 5-aminomethyl-3-(5-nitro-2-imidazolylmethyleneamino)-2-oxazolidinones. *Journal of Medicinal Chemistry* **1971**, *14* (2), 94-96. DOI: 10.1021/jm00284a003.
(25) Cavalleri, B.; Ballotta, R.; Arioli, V.; Lancini, G. New 5-substituted 1-alkyl-2-nitroimidazoles. *Journal of Medicinal Chemistry* **1973**, *16* (5), 557-560. DOI: 10.1021/jm00263a035.
(26) Thomsen, M. S.; Nawrocki, P. R.; Kofod, N.; Sørensen, T. J. Seven Europium(III) Complexes in Solution – The Importance of Reporting Data When Investigating Luminescence Spectra and Electronic Structure. *European Journal of Inorganic Chemistry* **2022**, *2022* (27), e202200334. DOI: 10.1002/ejic.202200334 (acccessed 2024/01/30).
(27) Aime, S.; Botta, M.; Ermondi, G. NMR study of solution structures and dynamics of lanthanide(III) complexes of DOTA. *Inorganic Chemistry* **1992**, *31* (21), 4291-4299. DOI: 10.1021/ic00047a016.
(28) Mizukami, S.; Takikawa, R.; Sugihara, F.; Hori, Y.; Tochio, H.; Wälchli, M.; Shirakawa, M.; Kikuchi, K. Paramagnetic Relaxation-Based 19F MRI Probe To Detect Protease Activity. *Journal of the American Chemical Society* **2008**, *130* (3), 794-795. DOI: 10.1021/ja077058z.
(29) Strauch, R. C.; Mastarone, D. J.; Sukerkar, P. A.; Song, Y.; Ipsaro, J. J.; Meade, T. J. Reporter Protein-Targeted Probes for Magnetic Resonance Imaging. *Journal of the American Chemical Society* **2011**, *133* (41), 16346-16349. DOI: 10.1021/ja206134b.
(30) Yamakoshi, Y.; Qiao, H.; Lowell, A. N.; Woods, M.; Paulose, B.; Nakao, Y.; Zhang, H.; Liu, T.; Lund-Katz, S.; Zhou, R. LDL-based nanoparticles for contrast enhanced MRI of atheroplaques in mouse models. *Chemical Communications* **2011**, *47* (31), 8835-8837, 10.1039/C1CC10924C. DOI: 10.1039/C1CC10924C.
(31) Wängler, C.; Wängler, B.; Eisenhut, M.; Haberkorn, U.; Mier, W. Improved syntheses and applicability of different DOTA building blocks for multiply derivatized scaffolds. *Bioorganic & Medicinal Chemistry* **2008**, *16* (5), 2606-2616. DOI: 10.1016/j.bmc.2007.11.044.
